# Distinctive Patterns of MicroRNA Expression Associated with Karyotype in Acute Myeloid Leukaemia

**DOI:** 10.1371/journal.pone.0002141

**Published:** 2008-05-14

**Authors:** Amanda Dixon-McIver, Phil East, Charles A. Mein, Jean-Baptiste Cazier, Gael Molloy, Tracy Chaplin, T. Andrew Lister, Bryan D. Young, Silvana Debernardi

**Affiliations:** 1 Institute of Cancer, Medical Oncology Centre, Barts and The London, School Of Medicine, London, United Kingdom; 2 Cancer Research UK, Bioinformatics & Biostatistics Service, London, United Kingdom; 3 Genome Centre, Barts and The London, School Of Medicine, London, United Kingdom; University of Cape Town, South Africa

## Abstract

Acute myeloid leukaemia (AML) is the most common acute leukaemia in adults; however, the genetic aetiology of the disease is not yet fully understood. A quantitative expression profile analysis of 157 mature miRNAs was performed on 100 AML patients representing the spectrum of known karyotypes common in AML. The principle observation reported here is that AMLs bearing a t(15;17) translocation had a distinctive signature throughout the whole set of genes, including the up regulation of a subset of miRNAs located in the human 14q32 imprinted domain. The set included *miR-127*, *miR-154*, miR-154*, *miR-299*, *miR-323*, *miR-368*, and *miR-370*. Furthermore, specific subsets of miRNAs were identified that provided molecular signatures characteristic of the major translocation-mediated gene fusion events in AML. Analysis of variance showed the significant deregulation of 33 miRNAs across the leukaemic set with respect to bone marrow from healthy donors. Fluorescent *in situ* hybridisation analysis using miRNA-specific locked nucleic acid (LNA) probes on cryopreserved patient cells confirmed the results obtained by real-time PCR. This study, conducted on about a fifth of the miRNAs currently reported in the Sanger database (microrna.sanger.ac.uk), demonstrates the potential for using miRNA expression to sub-classify cancer and suggests a role in the aetiology of leukaemia.

## Introduction

Acute myeloid leukaemia (AML) arises from the accumulation of myeloid precursor cells arrested at early stages of differentiation. Analysis of the karyotype of leukaemic cells has identified non-random somatically acquired translocations, inversions, and deletions, which are often associated with specific subtypes of AML [1]. The major gene fusion events are the t(8;21), t(15;17), inv(16), and the 11q23 rearrangements which together account for approximately 20% of all AMLs and result in the expression of chimeric proteins.[2]. Of the remaining AMLs, a substantial proportion, possibly as much as 40% [3], lacks any visible chromosomal abnormality and cannot be consistently associated with any known genetic lesion. Large scale clinical studies have demonstrated that cytogenetic abnormalities provide valuable information of prognostic relevance. Leukaemias fall into three broad cytogenetic prognostic risk groups, with the t(8;21), t(15;17), and inv(16) leukaemias having a more favourable outcome, whereas those with loss of chromosome 7, deletion of chromosome 5q and more complex karyotypes having an adverse outcome. All the other subtypes of AML, including those with rearrangement of 11q23 and normal karyotype, have an intermediate prognostic risk group [3]. Several studies have shown that genome-wide gene expression profiling can clearly distinguish the major cytogenetic groups, including normal karyotype samples, identifying specific sets of genes with expression patterns highly correlated with each karyotypic class [4]–[8] and so providing a better understanding of the underlying disease biology.

A new class of small non-coding RNA molecules, designated as microRNAs (miRNAs) [9], has been shown to play key roles in a number of regulatory functions, including modulation of haematopoiesis and cell differentiation in mammals [10]. MiRNAs are single stranded RNAs, typically 19–25 nucleotides in length, generated from endogenous transcripts and evolutionarily conserved. They modulate gene expression by complementarity-mediated binding to target mRNAs resulting in the repression of translation [11] or in the cleavage of the target transcript [12], [13]. There are several indications that miRNAs might be a new class of genes involved in human tumourigenesis. A proportion of human miRNA genes is reported to be located in regions involved in cancer [14] and several examples of an association between disrupted expression of specific miRNAs and cancer have been shown in a variety of tissues [15]–[18]. Lu and collaborators [19] were the first to observe distinct patterns of miRNA expression across tumour types, and miRNA profiles reflecting the developmental lineage and the differentiation state of the tumour.

The importance of miRNAs in AML has recently been emphasised by studies from this laboratory. Using a quantitative real-time PCR assay specific to the mature miRNA [20], we have demonstrated that the expression of a limited number of miRNAs in AML correlates with the AML global expression profile, and that *miR-181a* correlates with the morphological subtype and the expression of genes identified as potential targets [21]. These preliminary data illustrate the potential for using miRNA expression to subclassify cancer. To achieve greater statistical significance and to provide valuable insights into the oncogenic process we have now extended this study, and the expression levels of 157 miRNAs have been measured using the same technology in an expanded cohort of acute myeloid leukaemias. We demonstrate that miRNA expression profiles are correlated with the karyotype in primary adult AML, and that a set of miRNAs is differentially expressed with respect to normal haematopoietic tissue. We also developed a method to demonstrate the spatial localisation *in situ* of specific miRNAs identified in the quantification, to confirm their expression with relation to karyotype.

## Results

### MiRNA expression profiling discriminates karyotypes in AML

Using a quantitative real-time PCR (qRT-PCR) assay designed to prime only from the mature miRNA [20], the expression profiles of 157 miRNAs (Table S1) were determined in 100 primary AML specimens specifically chosen to exhibit the spectrum of known karyotypes common in AML (Table 1), with examples of AML French American British (FAB) morphological phenotypes [22] from M1 to M6 (Table S2). Two leukaemic cell lines, KG1 and NB4, and 2 bone marrow samples from healthy donors were also included. (The median and the normalised Ct values for each miRNA are reported in Tables S3 and S4, respectively). The data were normalised as described in the methods section and 34 miRNAs that were not expressed or did not change in level across the entire set of samples were excluded from the analysis.

**Table 1:** Distribution of the primary chromosomal aberrations across the set of 100 AMLs.

Primary chromosomal aberrations	Number of samples	
t(15;17)	*Sole aberration*	*5*
	*With additional cytogenetic changes*	*4*
t(8;21)	*Sole aberration*	*5*
	*−X/−Y*	*4*
	*With additional cytogenetic changes*	*2*
inv(16)	*Sole aberration*	*4*
	*+22*	*4*
	*With additional cytogenetic changes*	*1*
11q23 rearrangement	*t(6;11)*	*3*
	*t(9;11)*	*2*
Other structural and numerical aberrations		*30*
Normal karyotype		*36*
**TOTAL**		**100**

The total number of samples for the sole abnormality or in association with additional cytogenetic changes are given. (Full patient details are reported in Table S2 in the supporting information section).

**Figure 1:**
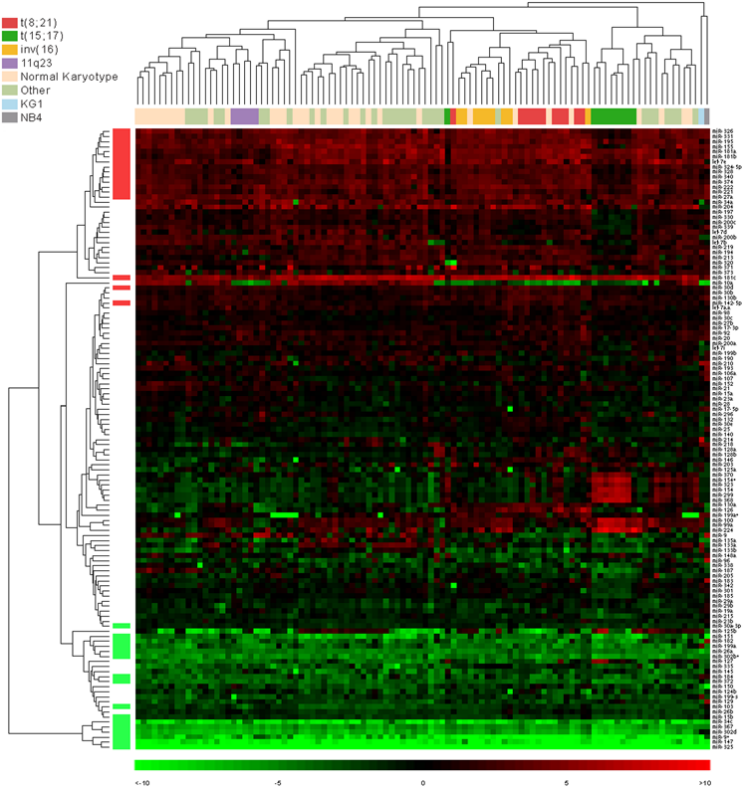
Unsupervised hierarchical cluster analysis of 123 miRNA expression profiles for 102 leukaemia samples. Each column represents a sample, and each row represents a single miRNA. The colour display encodes the logarithm of the expression changes, where varying shades of red and green indicate up and down regulation, respectively. Values ranged from log_2_ (−10) to log_2_(+10). MiRNAs passing a 5% FDR threshold from the leukaemia versus normal bone marrow comparison are highlighted in red or green on the left of the heatmap. Karyotype labels are also indicated on the top of the heatmap. The colour-key for the labelling is on the top left.

An unsupervised hierarchical cluster analysis of the remaining 123 miRNA expression profiles for 102 leukaemia samples, scaled to the median of the normal bone marrow controls, revealed molecular signatures characteristic of the major translocation-mediated gene fusion events in AML (Figure 1). The most distinctive signature throughout the whole set of miRNAs was associated with the promyelocytic leukaemias (APMLs) bearing the t(15;17) translocation. All but two APML patient samples clustered together showing high expression of a group of miRNAs. Of the two leukaemia samples that did not cluster with the other members of the t(15;17) group, one (patient n. 42) had an additional translocation t(1;10)(p32;p11.2) and standard M3 morphology, whereas the other (patient n. 94) was reclassified. At presentation patient n. 94 failed G-banded analysis but fluorescent *in situ* hybridisation (FISH) showed a positive signal for the PML/RARA rearrangement in 38% of cells. However, within 17 months, the patient was diagnosed with myelodysplastic syndrome with refractory anemia with excess blasts (MDS-RAEB). There was no more evidence of a PML/RARA fusion by FISH, but G-banded analysis revealed the presence of a t(12;17)(p13;q22) translocation. Samples bearing either the t(8;21) translocation or the inv(16) were grouped in two distinct clusters of the same major branch. The 5 AMLs with rearrangement of the MLL gene at 11q23 were clustered together within the group of monocytic leukaemias. Leukaemias with a normal karyotype or other non recurrent rearrangements were split in two major groups according to their morphological FAB subtype, myelocytic leukaemias M1 and M2 and monocytic leukaemias M4 and M5. Within each cluster the normal karyotype leukaemias were grouped together. Chromosomal numerical abnormalities did not correlate with the level of expression of the miRNAs. The 6 samples with an additional chromosome 8 (samples n. 53, 55, 65, 72, 75, and 100) did not cluster together and were rather grouped with other samples according to their morphological subtype. The two cell lines, NB4 and KG1, did not show any similarity with the patient samples and clustered separately. The unsupervised analysis also split the samples into two main groups according to their associated cytogenetic prognostic risk category [3] either favourable or intermediate. Patient samples with an adverse prognosis were a minority and were scattered across the set.

### Analysis of variance (ANOVA) reveals high expression of miRNA genes located at 14q32 in APML

To find miRNAs with statistically significant differences in expression level among the major cytogenetic groups (Table 1), an ANOVA test was applied to the 102 AML samples, including the two cell lines. Ninety-four miRNAs passed a 5% false discovery rate (FDR) filter (Table S5 in the supporting information section). APMLs bearing the t(15;17) translocation were characterised by the up regulation of 7 miRNAs transcribed from genes located at the 14q32 region. The set includes *miR-127*, *miR-154*, miR-154*, *miR-299*, *miR-323*, *miR-368*, and *miR-370*. The human panel of 157 miRNAs screened in the study included three other miRNAs located in this region, *miR-134*, *miR-337*, and *miR-342*, but their expression did not have any statistical significance associated with APML samples. The NB4 cell line, although bearing the t(15;17) translocation, did not show expression of any of the miRNAs located at 14q32. The t(15;17) group of patients was also characterised by the down regulation of 9 miRNAs (*miR-17-3p*, *miR-185*, *miR-187*, *miR-194*, *miR-200a*, *miR-200b*, *miR-200c*, *miR-330*, and *miR-339*). In this case no association with a specific genome location was observed. Samples with an inv(16) were characterised by the high level of *miR-99a*, *miR-100*, and *miR-224*, whereas AMLs carrying a t(8;21) translocation showed a high expression of *miR-146a* and a down regulation of *miR-133a*. The expression of *miR-9* and *miR-let7b* correlated with the cytogenetic prognostic risk associated to the samples, being low in the favourable groups and high in intermediate and adverse leukaemias. Two miRNAs, *miR-10a* and *miR-125b* showed high degree of variability across the set of samples. The first was not expressed in samples with 11q23 rearrangements and samples with a favourable outcome. *MiR-125b* was mainly expressed in leukaemias with a normal karyotype.

### Differential expression profiles between AMLs and normal bone marrow controls

A 5% FDR threshold ANOVA test revealed the deregulation of 33 miRNAs across the leukaemic set with respect to bone marrow from healthy donors (Table 2). Seventeen miRNAs were up regulated and 16 down regulated in the leukaemia samples. MiRNAs that are up or down regulated in AMLs are highlighted in Figure 1 by a red and green bar, respectively, on the left side of the heatmap.

**Table 2:** List of the 33 miRNAs showing different expression levels between leukaemia samples and normal bone marrow.

miRNAs up in AMLs	Acc. N. mature miRNA	miRNAs down in AMLs	Acc. N. mature miRNA
*let-7e*	MIMAT0000066	miR-9*	MIMAT0000442
*miR-27a*	MIMAT0000084	*miR-15b*	MIMAT0000417
*miR-30d*	MIMAT0000245	*miR-26a*	MIMAT0000082
*miR-142-5p*	MIMAT0000433	*miR-30a-3p*	MIMAT0000088
*miR-155*	MIMAT0000646	*miR-34c*	MIMAT0000686
*miR-181a*	MIMAT0000256	*miR-103*	MIMAT0000101
*miR-181b*	MIMAT0000257	*miR-147*	MIMAT0000251
*miR-181c*	MIMAT0000258	*miR-151*	MIMAT0000757
*miR-195*	MIMAT0000461	*miR-182*	MIMAT0000259
*miR-221*	MIMAT0000278	*miR-184*	MIMAT0000454
*miR-222*	MIMAT0000279	*miR-199a*	MIMAT0000231
*miR-324-5p*	MIMAT0000761	miR-302b*	MIMAT0000714
*miR-326*	MIMAT0000756	*miR-302d*	MIMAT0000718
*miR-328*	MIMAT0000752	*miR-325*	MIMAT0000771
*miR-331*	MIMAT0000760	*miR-367*	MIMAT0000719
*miR-340*	MIMAT0000750	*miR-372*	MIMAT0000724
*miR-374*	MIMAT0000727		

In the left and right panels are reported the 17 up regulated and the 16 down regulated miRNAs, respectively, in AMLs. (Acc. N. = accession number, Sanger miRBase database 9/04, Version 5.0, http://microrna.sanger.ac.uk/).

### Validation of pattern of expression of miR-127 and miR-154 in AML by locked nucleic acid fluorescent in situ hybridisation (LNA-FISH)

**Figure 2:**
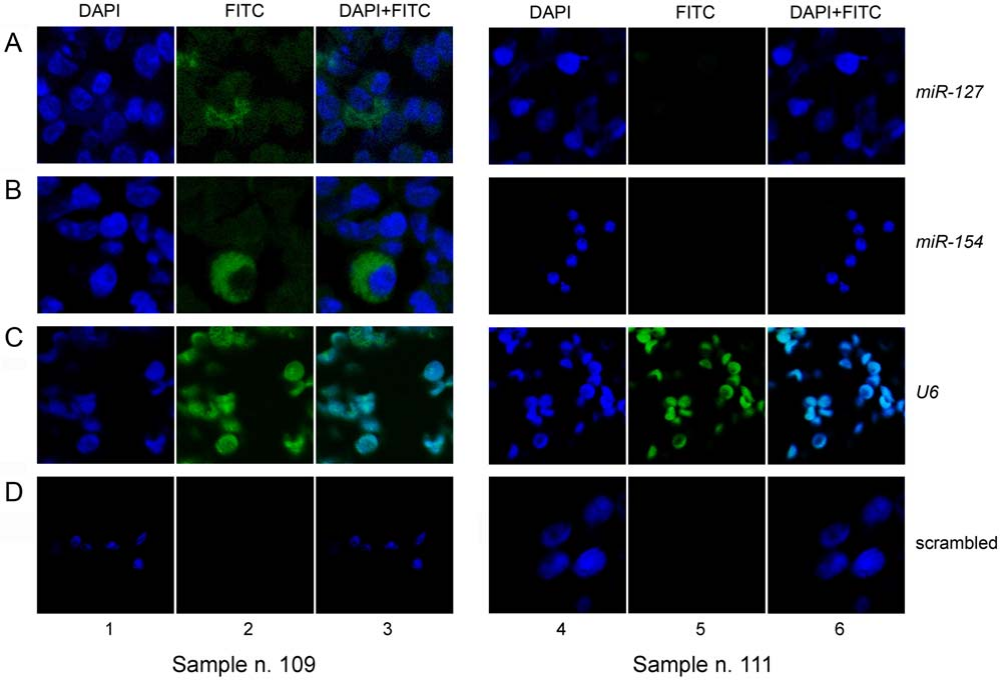
MiRNA detection in cryopreserved bone marrow cells by LNA-FISH. Patients n. 109 (lanes 1–3) and n. 111 (lanes 4–6) carrying the t(15;17) and t(9;22) translocations, respectively, are shown. All images were obtained with the confocal microscope as described in the method. The DAPI nuclear staining (blue), the fluorescent *in situ* hybridisation signals obtained with FITC-conjugated antibody (green), and the combined images are indicated. The A and B panels show the detection of *miR-127* and *miR-154*, respectively. Both miRNAs are detected in the cytoplasm of cells (lanes 2 and 3) of patient n. 109 but not in patient n. 111 (lanes 5 and 6). The C panel shows the nuclear expression of *U6*, the small RNA used as positive control, in both samples (lanes 2 and 5). No signal was detected when cells were hybridised with a scrambled oligonucleotide (negative control), as shows in lanes 2 and 5 of the D panel.

*In situ* hybridisation using locked nucleic acid (LNA)-modified probes, digoxigenin (DIG) labelled, permits the visualisation and spatial localisation of mature miRNAs within a number of tissues and cell types. These synthetic nucleic acid analogues increase the thermostability of the nucleic acid duplexes when incorporated into oligonucleotides [23]. We further developed this methodology to visualise the spatial localisation of two mature miRNAs, *miR-127* and *miR-154*, in primary AML suspension cells and thus we confirmed their high expression in APMLs as measured by real-time PCR. Seven patient samples (patients n. 104–110) carrying the t(15;17) translocations were selected based on karyotypic abnormality and sample availability. Three samples with different cytogenetics were used as negative controls. One had a normal karyotype (patient n. 112), patients n. 111 and 113 had a t(9;22) and a t(8;21) translocation, respectively (Table S2). Digoxigenin labelled LNA probes were detected using an anti-DIG fluorescein isothiocyanate (FITC) conjugated antibody and the fluorescent signals visualised with a confocal microscope which permitted the spatial localisation of the miRNAs. An example is shown in Figure 2 for patients n. 109 and n. 111. The expression of *miR-127* by LNA-FISH was seen in all samples with a t(15;17) translocation and in the sample with a t(8;21) translocation. The expression of *miR-154* was detected in all the 7 patients with a t(15;17) translocation but not in the 3 patients with other karyotypes. The detection of both miRNAs was confined to the cytoplasm (Figure 2A and 2B). Confocal imaging confirmed the nuclear expression of the small non coding RNA *U6* (M14486), chosen as positive control [24], in all the patient samples (Figure 2C). The scrambled oligonucleotide was negative in all the samples (Figure 2D). The number of positive signals for the 4 probes was counted in 100 cells in each patient and a percentage calculated (Table 3).

**Table 3:** Percentage of positive cells for each LNA probe in 10 AMLs.

	n. 104	n. 105	n. 106	n. 107	n. 108	n. 109	n. 110	n. 111	n. 112	n. 113
	*t(15;17)*	*t(15;17)*	*t(15;17)*	*t(15;17)*	*t(15;17)*	*t(15;17)*	*t(15;17)*	*t(9;22)*	*NK*	*t(8;21)*
***miR-127***	25	76	27	95	55	84	84	<5	<5	25
***miR-154***	10	38	23	33	58	55	35	<5	<5	<5
***U6*** ** (+ve ctrl)**	82	75	78	81	78	88	84	84	100	80
**−ve ctrl**	<5	<5	<5	<5	<5	<5	<5	<5	<5	<5

A simplified karyotype is reported in italics. NK = normal karyotype.

## Discussion

This study represents a detailed analysis of miRNA expression in a cohort of 100 primary AMLs. The most striking result is that AMLs bearing the t(15;17) translocation have a distinctive up regulation of seven miRNAs located on the human 14q32 imprinted domain. Specific subsets of microRNAs were identified that provide molecular signatures characteristic of the major translocation-mediated gene fusion events in AML (Figure 1). It should be noted that the genes for the seven miRNAs highly expressed in the samples carrying the t(15;17) translocation are spread over less than 200,000 bases on chromosome 14q32. They are organised in clusters together with many other miRNAs not included in the study [25]. Their imprinted expression, of maternal origin, is regulated by an intergenic differentially methylated region (DMR) located ∼200 kb upstream from the miRNA cluster [25]. Epigenetic alteration at the imprinted domain, defined by *DLK1* (AL132711) and *GTL2* (AL117190) genes [26], has been reported in human neoplasia, causing alteration in the expression of *GTL2*, although loss of imprinting for *DLK1*, has not been found [27], [28]. Interestingly, although included in the same region, three miRNAs did not show an over expression in the samples with a t(15;17) translocation when measured by real-time PCR. This might imply a more complex mechanism of regulation. It has been suggested that miRNAs in this region act as tumour repressor genes and that changes in the methylation status of their promoters could trigger cancer development [29]. For example, *miR-127* has been shown to be expressed, together with other members of its cluster, in normal tissues [29], but to be down regulated or silenced in cancer cell lines and primary tumours; the expression is correlated with the methylation and the acetylation status of its promoter. The use of inhibitors of methylation and histone deacetylation in these cancer cells causes over expression of *miR-127* and related down regulation of the target *BCL6* (NM_138931), a *bona fide* proto-oncogene [29], [30]. It is possible that the specific expression of *miR-127* and the other members of the cluster, in a subtype of leukaemia carrying the t(15;17) translocation, is due to a change in the methylation and acetylation status of the 14q32 region. The activation of the expression could be dictated by the developmental stage of the blast cells, or the chimeric product of the translocation (*PML/RAR*α) (NM_002675/X06538) could have a role in the process. Epigenetically regulated expression of miRNA genes associated with oncogenic function has now been described for some miRNAs [31], [32] and could be true for many more. It is noteworthy that to our knowledge the over expression of miRNAs at 14q32 has never been observed to be associated with a specific subtype of acute myeloid leukaemia, neither in cell lines [33], [34], nor in primary tumours [35]–[37]. In a recently published work, Mi and colleagues [36] showed distinct miRNA signatures between acute lymphoblastic leukaemias (ALLs) and AMLs. The signatures were grouping the samples according to the major translocation events in a similar fashion to the study presented here. However, the set of miRNAs analysed was only partially overlapping, and the distinct expression of the miRNAs located at 14q32 in the APML samples was not observed.

It is noticeable that the leukaemic karyotypes other than those with a t(15;17) were distinguished by the combined differential pattern of expression of just a few miRNAs. Moreover, some miRNAs showed high variability across the whole set of AMLs. For example, leukaemias with rearrangement of the *MLL* (NM_005933) gene at 11q23 region, were characterised by the absence of expression of *miR-10a*, *miR-331*, and *miR-340*. *MiR-10a* was also found down regulated in all the leukaemias carrying the major translocation events, associated with a more favourable prognosis, and in some cases of AML with a normal karyotype as we had previously observed [21]. *MiR-10a* is known to target *HOXA1* (NM_005522) gene [35]. Another miRNA with variable expression across the set of samples is *miR-125b*. *MiR-125b* was reported down regulated both in breast cancer [38] and in human prostate cancer [39]. It targets *ERBB2* and *ERBB3* in human breast cancer cell lines [40] and its depletion is critical for the proliferation of differentiated cells.

The comparison of the leukaemia samples with bone marrow from healthy donors highlighted the differential expression of a number of miRNAs potentially involved in the oncogenic process. Among the up regulated miRNAs in AMLs, a number were already known to be haematopoietic tissue-specific, i.e. *miR-142-5p*, *miR-155*, and *miR-181* [10], [41], [42], and/or reported highly expressed in a variety of haematological malignancies and solid tumours, i.e. *miR-221*, and *miR-222*. In particular, *miR-155*, a negative regulator of normal myelopoiesis and erythropoiesis [41], was first observed highly expressed in paediatric Burkitt lymphomas [17]. Later, over expression of *miR-155* was reported in other haematological malignancies [43], and in solid tumours [44]. *MiR-181a*, absent in B-cell chronic lymphocytic leukaemia (B-CLL) and mature B cells [42], known to inhibit the differentiation of all haematopoietic lineages [41], was also found associated with a specific leukaemic phenotype [21] in AMLs with a normal karyotype. *MiR-181* regulates the expression of *TCL1* (X82240) in CLL [45]. *MiR-221* and *miR-222*, involved in the negative control of human erythropoiesis by blocking the *C-Kit* (S67773) gene [46], have been reported to be highly expressed in a variety of solid tumours including colon, pancreas, and prostate cancers [44], [47]. In some of these cases, *miR-221* has been shown to block the expression of a cell cycle inhibitor and tumour suppressor gene, *p27(Kip1)* (AF480891) [48], reinforcing the hypothesis of its oncogenic role. Isken and colleagues [37] have also reported a significantly higher expression of *miR-221* and *miR-222* in AML blasts.

It is notable that, among the 16 down regulated miRNAs (with respect to the normal controls) 5 miRNAs were previously described as having an anti-oncogenic role. For example *miR-34c*, a target of p53, was shown to cooperate in suppressing proliferation and soft-agar colony formation of neoplastic epithelial ovarian cells [49]. *MiR-184* was found down regulated in paediatric neuroblastomas with *MYCN* (NM_005378) amplification and poorer prognoses [50]. Also down regulated were *miR-26a* and *miR-199a*, both described as potential tumour suppressor genes in a recent study analysing the expression of 241 human miRNAs in normal tissues and in the NCI-60 panel of human tumour-derived cell lines [34]. Other down regulated miRNAs in the leukaemia samples but with different functions include *miR-103* and *miR-372*. The first is known to act in the differentiation of granulocytic, monocytic, and B-lymphoid lineages [41], and its down regulation might have a role in the differentiation block typical of leukaemias. *MiR-372* is a potential oncogene involved in the human testicular germ cell tumour through the deactivation of the p53 pathway [51].

This is the first time, to our knowledge, that *in situ* fluorescent hybridisation using DIG labelled LNA probes has been used for the local visualisation of mature miRNAs in haematopoietic suspension cells. The LNA probe hybridisation yields highly accurate signals able to discriminate between single nucleotide differences and hence between closely related miRNA family members [23], [52]. Compared to other techniques such as northern blots, it offers the possibility to detect miRNAs in a sparse population of cells. The selected miRNAs, *miR-127* and *miR-154*, were clearly visualised and compared in different leukaemic subtypes, providing a validation of the real-time PCR measurement.

This study, conducted on about a fifth of the miRNAs currently reported in the Sanger database (microrna.sanger.ac.uk), demonstrates the potential for using miRNA expression to subclassify cancer, and suggests that miRNAs might play an important role in the molecular pathogenesis of AML interfering with pathways essential at various stages of haematopoiesis.

## Materials and Methods

### Patient samples and controls

This study was performed using archival material. One hundred and ten peripheral blood (PB) or bone marrow (BM) samples from AML patients were obtained from the St Bartholomew's Hospital tissue bank. The diagnosis of leukaemia was made based on clinical presentation and morphological analysis as previously described [5]. The average blast percentage for these samples was 63% with a median value of 83%. (Full sample information, including French American British (FAB) morphological subtype [22] and cytogenetic karyotypes are reported in Table S2). RNA was extracted from 2 BM samples from healthy donors. Two leukaemic cell lines were included KG1 (a cell line derived from a patient with erythroleukaemia in myeloblastic relapse) and NB4 (a cell line derived from a patient with acute promyelocytic leukaemia).

Written informed consent was obtained from all the patients, when possible, to store the excess diagnostic tissue for research purposes. When patient consent was not obtained because of death, patient anonymity was preserved. Ethical approval to access the stored material and perform the study described here was obtained from the East London and The City Health Authority Research Ethics Committee (P1/03/133).

### Cell culture

Fresh patient samples were either immediately processed for RNA extraction or cryopreserved following Lymphoprep™ (Axis-Shield, Norway) separation and therefore did not require culturing. Cell lines, KG1 and NB4, were cultured and maintained in RPMI 1640 medium with Glutamax (GIBCO, Invitrogen, Carlsbad, CA) supplemented with 10% heat-inactivated foetal calf serum (GIBCO) and 1% penicillin/streptomycin antibiotics at 37°C in a humidified atmosphere containing 5% CO_2_. All cultures passed mycoplasma testing.

### Quantitative real-time PCR assay

Total RNA was extracted from fresh or cryopreserved patient samples, either BM or PB, using TRIzol (Invitrogen Life Technologies) according to manufacturer's instructions. Samples were reverse transcribed using the TaqMan®MicroRNA Reverse Transcription Kit (P/N 4366597, Applied Biosystems) and mature miRNAs were quantified by real-time PCR using the TaqMan® MicroRNA Assays - Human Panel Early Access Kit (P/N 4365409, Applied Biosystems) [20] as previously described [21]. The kit contained assays for 157 miRNAs (Table S1) of the 733 currently listed in the Sanger miRBase database [53]. Reactions for patient samples n. 1 to 100, the 2 normal BM samples, and the 2 cell lines, were prepared using a Biomek®Fx Laboratory Automation Workstation (Beckman Coulter, Inc) and volumes adapted as necessary. The reaction mixtures were prepared in triplicate in two 384-well plates, A and B. For patient samples n. 104 to 113, real-time PCR reactions were manually prepared using TaqMan® individual MicroRNA Assays (Applied Biosystems) for *miR-127* (P/N 4373147), *miR-154* (P/N 4373270), and *miR-16* (P/N 4373121). Reactions were performed in triplicate as described above. All the PCR reactions were performed using an ABI 7900HT Sequence Detection System.

### Data analysis

Undetermined raw Ct values were set to 40. Median Ct replicate values were calculated and each plate scaled to the negative plate median Ct value. Prior to analysis, miRNAs displaying a coefficient of variance less than 0.15 across all samples were excluded along with miRNAs with a normalised Ct value less than 0 in all samples. After this 123 miRNAs remained. Plates A and B for each sample were merged. In the unsupervised two-dimensional cluster analysis, the 123 miRNA expression profiles for 102 leukaemia samples were scaled to the median of the normal bone marrow controls. MiRNAs and samples were clustered using complete linkage and euclidean distance. Analysis of variance (ANOVA) was performed using a 5% false discovery rate (FDR) threshold to identify differentially expressed miRNAs between leukaemia samples and normal bone marrow. MiRNAs specific to karyotype groupings were identified in the same way. The analysis was carried out using the *limma* package from Bioconductor [54].

### Locked nucleic acid fluorescent in situ hybridisation (LNA-FISH)

Locked nucleic acid (LNA)-modified probes were obtained for two miRNAs (*miR-127* and *miR-154*) and positive (*U6*) and negative controls (miRCURY-LNA detection probe, Exiqon). The probe sequences were (5′-3′): *miR-154*, CGAAGGCAACACGGATAACCTA; *miR-127*, AGCCAAGCTCAGACGGATCCGA; *U6*, CACGAATTTGCGTGTCATCCTT. A scrambled oligonucleotide was used as negative control: TTCACAATGCGTTATCGGATGT. Full detailed probe labelling and hybridisation protocols are described in Protocol S1 in the Supporting Information section. Briefly, LNA detection probes were labelled with digoxigenin (DIG) using a DIG 3′end labelling kit (P/N 03353583910, Roche). One hundred µl of 0.5 million cells/ml from thawed BM or PB was pipetted into a cytospin column and spun on poly-*l*-lysine coated glass slides. After application of 200 µl of the hybridisation mixture containing ∼30 ng/µl of labelled probe, slides were incubated for 17–18 hours at a temperature 20–22°C below the melting temperature (Tm) of the miRCURY LNA probe used in a humidified HYBrite™ (Abbott Laboratories Ltd). Slides were extensively washed and then incubated for 1 hour at 37°C with anti-DIG fluorescein isothiocynate (FITC) conjugated antibody, washed again, drained and counterstained with 4′-6′Diamidino-2-phenylindole (DAPI), before the addition of ProLong® Gold antifade reagent (Invitrogen).

### Microscopy and image analysis

LNA-FISH signals were visualised on a Zeiss 510 Meta confocal microscope equipped with a Plan-Apochromat 63×/1.4OilDIC lens, fitted with a motorised stage. One hundred cells were assessed for each probe. Image stacks of cells were captured and analysed using programs LSM510, version 3.2SP2 and Image J, version 1.39d (http://rsb.info.nih.gov/ij/index.html).

## Supporting Information

Table S1 List of the 157 human miRNAs included in the study. The miRNAs analysed in the work, reported here alongside their accession number and sequence, were selected from the Sanger miRBase database v5.0, Sept 2004, http://microrna.sanger.ac.uk/. (n/a refers to miRNAs currently removed from the database).
(0.05 MB PDF)

Table S2 AML patient details. Sex, age at diagnosis, percentage of blasts, morphological FAB subtype, and karyotype are reported. (BM = bone marrow; PB = peripheral blood; MDS = myelodysplastic syndrome; RAEB = refractory anemia with excess blasts).
(0.05 MB PDF)

Table S3 Real-time PCR results, raw data. The spreadsheet contains the median Ct values of 157 miRNAs quantified in 100 AML samples, 2 cell lines (NB4 and KG1), and 2 normal bone marrow controls (NBM4 and NBM5).
(0.25 MB PDF)

Table S4 Real-time PCR results, normalised data. The spreadsheet contains the values of 123 miRNAs after normalisation for 100 AML samples, 2 cell lines (NB4 and KG1), and 2 normal bone marrow controls (NBM4 and NBM5). Median Ct values were normalised to the negative plate median (as described in the method section of the manuscript) so negative values are the low abundant miRNAs, positive the more abundant.
(0.24 MB PDF)

Table S5 List of the 94 miRNAs that passed the 5% FDR filter in the ANOVA test. The miRNAs are reported alongside their accession number and sequence (Sanger miRBase database v5.0, Sept 2004, http://microrna.sanger.ac.uk/. n/a refers to miRNAs removed from the Registry).
(0.04 MB PDF)

Protocol S1 Locked nucleic acid fluorescent in situ hybridisation (LNA-FISH) detailed protocol.
(0.05 MB DOC)
