# Supplementary material for: Distinctive Patterns of MicroRNA Expression Associated with Karyotype in Acute Myeloid Leukaemia
Source: PLoS One. 2008 May 14;3(5):e2141. doi: 10.1371/journal.pone.0002141 (PMC2373886; doi:10.1371/journal.pone.0002141)
Supplement: Table S1 — List of the 157 human miRNAs included in the study. The miRNAs analysed in the work, reported here alongside their accession number and sequence, were selected from the Sanger miRBase database v5.0, Sept 2004, http://microrna.sanger.ac.uk/. (n/a refers to miRNAs currently removed from the database). (0.05 MB PDF) [file pone.0002141.s001.pdf]

Table S1. List of the 157 human miRNAs included in the study.

Mature miRNA	Accession number	Mature miRNA Sequence	
hsa-let-7a	MIMAT0000062	ugagguaguagguuguauaguu	
hsa-let-7b	MIMAT0000063	ugagguaguagguugugugguu	
hsa-let-7d	MIMAT0000065	agagguaguagguugcauagu	
hsa-let-7e	MIMAT0000066	ugagguaggagguuguauagu	
hsa-let-7g	MIMAT0000414	ugagguaguaguuuguacagu	
hsa-let-7i	MIMAT0000415	ugagguaguaguuugugcu	
hsa-miR-9	MIMAT0000441	ucuuugguuaucuagcuguauga	
hsa-miR-9*	MIMAT0000442	uaaagcuagauaaccgaaagu	
hsa-miR-10a	MIMAT0000253	uacccuguagauccgaauuugug	
hsa-miR-15a	MIMAT0000068	uagcagcacauaaugguuugug	
hsa-miR-15b	MIMAT0000417	uagcagcacaucaugguuuaca	
hsa-miR-16	MIMAT0000069	uagcagcacguaaauauuggcg	
hsa-miR-17-3p	MIMAT0000071	acugcagugaaggcacuugu	
hsa-miR-17-5p	MIMAT0000070	caaagugcuuacagugcagguagu	
hsa-miR-19a	MIMAT0000073	ugugcaaaucuaugcaaaacuga	
hsa-miR-20	MIMAT0000075	uaaagugcuuauagugcaggua	
hsa-miR-21	MIMAT0000076	uagcuuaucagacugauguuga	
hsa-miR-23a	MIMAT0000078	aucacauugccagggauuucc	
hsa-miR-23b	MIMAT0000418	aucacauugccagggauuaccac	
hsa-miR-25	MIMAT0000081	cauugcacuugucucggucuga	
hsa-miR-26a	MIMAT0000082	uucaaguaauccaggauaggcu	
hsa-miR-26b	MIMAT0000083	uucaaguaauucaggauaggu	
hsa-miR-27a	MIMAT0000084	uucacaguggcuaaguuccgcc	
hsa-miR-27b	MIMAT0000419	uucacaguggcuaaguucug	
hsa-miR-28	MIMAT0000085	aaggagcucacagucuauugag	
hsa-miR-29a	MIMAT0000086	cuagcaccaucugaaaucgguu	
hsa-miR-29b	MIMAT0000100	uagcaccauuugaaaucagu	
hsa-miR-29c	MIMAT0000681	uagcaccauuugaaaucgguua	
hsa-miR-30a-3p	MIMAT0000088	cuuucagucggauguuugcagc	
hsa-miR-30b	MIMAT0000420	uguaaacauccuacacucagc	
hsa-miR-30c	MIMAT0000244	uguaaacauccuacacucucagc	
hsa-miR-30d	MIMAT0000245	uguaaacauccccgacuggaag	
hsa-miR-30e	MIMAT0000692	uguaaacauccuugacugga	
hsa-miR-31	MIMAT0000089	ggcaagaugcuggcauagcug	
hsa-miR-34a	MIMAT0000255	uggcagugucuuagcugguugu	
hsa-miR-34b	MIMAT0000685	aggcagugucauuagcugauug	
hsa-miR-34c	MIMAT0000686	aggcaguguaguuagcugauug	
hsa-miR-92	MIMAT0000092	uauugcacuugucccggccugu	
hsa-miR-95	MIMAT0000094	uucaacggguauuuauugagca	
hsa-miR-96	MIMAT0000095	uuuggcacuagcacauuuuugc	
hsa-miR-98	MIMAT0000096	ugagguaguaaguuguauuguu	
hsa-miR-99a	MIMAT0000097	aacccguagauccgaucuugug	
hsa-miR-100	MIMAT0000098	aacccguagauccgaacuugug	
hsa-miR-103	MIMAT0000101	agcagcauuguacagggcuauga	
hsa-miR-104	n/a	ucaacaucagucugauaagcua	
hsa-miR-105	MIMAT0000102	ucaaaugcucagacuccugu	
hsa-miR-106a	MIMAT0000103	aaaagugcuuacagugcagguagc	
hsa-miR-107	MIMAT0000104	agcagcauuguacagggcuauca	
hsa-miR-122a	MIMAT0000421	uggagugugacaaugguguuugu	
hsa-miR-124a	MIMAT0000422	uuaaggcacgcggugaaugcca	
hsa-miR-124b	n/a	uuaaggcacgcggugaaugc	
hsa-miR-125a	MIMAT0000443	ucccugagacccuuuaaccugug	
hsa-miR-125b	MIMAT0000423	ucccugagacccuaacuuguga	
hsa-miR-126	MIMAT0000445	ucguaccgugaguaauaaugc	
hsa-miR-127	MIMAT0000446	ucggauccgucugagcuuggcu	
hsa-miR-128a	MIMAT0000424	ucacagugaaccggucucuuuu	
hsa-miR-128b	MIMAT0000676	ucacagugaaccggucucuuuc	

Table S1. List of the 157 human miRNAs included in the study.

hsa-miR-129	MIMAT0000242	cuuuuugcggucugggcuugc	
hsa-miR-130a	MIMAT0000425	cagugcaauguuaaaagggc	
hsa-miR-130b	MIMAT0000691	cagugcaaugaugaaagggcau	
hsa-miR-132	MIMAT0000426	uaacagucuacagccauggucg	
hsa-miR-133a	MIMAT0000427	uugguccccuucaaccagcugu	
hsa-miR-133b	MIMAT0000770	uugguccccuucaaccagcua	
hsa-miR-134	MIMAT0000447	ugugacugguugaccagaggg	
hsa-miR-135a	MIMAT0000428	uauggcuuuuuauuccuauguga	
hsa-miR-135b	MIMAT0000758	uauggcuuuucauuccuaugug	
hsa-miR-137	MIMAT0000429	uauugcuuaagaauacgcguag	
hsa-miR-138	MIMAT0000430	agcugguguugugaauc	
hsa-miR-139	MIMAT0000250	ucuacagugcacgugucu	
hsa-miR-140	MIMAT0000431	agugguuuuacccuaugguag	
hsa-miR-141	MIMAT0000432	aacacugucugguaaagaugg	
hsa-miR-142-3p	MIMAT0000434	uguaguguuuccuacuuuaugga	
hsa-miR-142-5p	MIMAT0000433	cauaaaguagaaagcacuac	
hsa-miR-144	MIMAT0000436	uacaguauagaugauguacuag	
hsa-miR-145	MIMAT0000437	guccaguuuucccaggaaucccuu	
hsa-miR-146	MIMAT0000449	ugagaacugaauuccaugggguu	
hsa-miR-147	MIMAT0000251	guguguggaaaugcuucugc	
hsa-miR-148a	MIMAT0000243	ucagugcacuacagaacuuugu	
hsa-miR-149	MIMAT0000450	ucuggcuccgugucuucacucc	
hsa-miR-150	MIMAT0000451	ucucccaacccuuguaccagug	
hsa-miR-151	MIMAT0000757	acuagacugaagcuccuugagg	
hsa-miR-152	MIMAT0000438	ucagugcaugacagaacuugg	
hsa-miR-154	MIMAT0000452	uagguuauccguguugccuucg	
hsa-miR-154*	MIMAT0000453	aaucauacacgguugaccuauu	
hsa-miR-155	MIMAT0000646	uuaaugcuaaucgugauagggg	
hsa-miR-181a	MIMAT0000256	aacauucaacgcugucggugagu	
hsa-miR-181b	MIMAT0000257	aacauucauugcugucgguggguu	
hsa-miR-181c	MIMAT0000258	aacauucaaccugucggugagu	
hsa-miR-182	MIMAT0000259	uuuggcaaugguagaacucaca	
hsa-miR-182*	MIMAT0000260	ugguucuagacuugccaacua	
hsa-miR-183	MIMAT0000261	uauggcacugguagaauucacug	
hsa-miR-184	MIMAT0000454	uggacggagaacugauaagggu	
hsa-miR-185	MIMAT0000455	uggagagaaaggcaguuc	
hsa-miR-186	MIMAT0000456	caaagaauucuccuuuugggcuu	
hsa-miR-187	MIMAT0000262	ucgugucuuguguugcagccg	
hsa-miR-189	MIMAT0000079	gugccuacugagcugauaucagu	
hsa-miR-190	MIMAT0000458	ugauauguuugauauauuaggu	
hsa-miR-191	MIMAT0000440	caacggaaucccaaaagcagcu	
hsa-miR-193	MIMAT0000459	aacuggccuacaaagucccag	
hsa-miR-194	MIMAT0000460	uguaacagcaacuccaugugga	
hsa-miR-195	MIMAT0000461	uagcagcacagaaauauuggc	
hsa-miR-197	MIMAT0000227	uucaccaccuucuccacccagc	
hsa-miR-198	MIMAT0000228	gguccagaggggagauagg	
hsa-miR-199a	MIMAT0000231	cccaguguucagacuaccuguuc	
hsa-miR-199a*	MIMAT0000232	uacaguagucugcacauugguu	
hsa-miR-199b	MIMAT0000263	cccaguguuuagacuaucuguuc	
hsa-miR-199-s	n/a	cccaguguucagacuaccuguu	
hsa-miR-200a	MIMAT0000682	uaacacugucugguaacgaugu	
hsa-miR-200b	MIMAT0000318	cucuaauacugccugguaaugaug	
hsa-miR-200c	MIMAT0000617	aauacugccggguaaugaugga	
hsa-miR-203	MIMAT0000264	gugaaauguuuaggaccacuag	
hsa-miR-204	MIMAT0000265	uucccuuugucauccuaugccu	
hsa-miR-205	MIMAT0000266	uccuucauuccaccggagucug	
hsa-miR-210	MIMAT0000267	cugugcgugugacagcggcug	
hsa-miR-211	MIMAT0000268	uucccuuugucauccuucgccu	

Table S1. List of the 157 human miRNAs included in the study.

hsa-miR-213	MIMAT0000270	accaucgaccguugauuguacc	
hsa-miR-214	MIMAT0000271	acagcaggcacagacaggcag	
hsa-miR-215	MIMAT0000272	augaccuaugaauugacagac	
hsa-miR-216	MIMAT0000273	uaaucucagcuggcaacugug	
hsa-miR-218	MIMAT0000275	uugugcuugaucuaaccaugu	
hsa-miR-219	MIMAT0000276	ugauuguccaaacgcaauucu	
hsa-miR-220	MIMAT0000277	ccacaccguaucugacacuuu	
hsa-miR-221	MIMAT0000278	agcuacauugucugcuggguuuc	
hsa-miR-222	MIMAT0000279	agcuacaucuggcuacugggucuc	
hsa-miR-223	MIMAT0000280	ugucaguuugucaaauacccc	
hsa-miR-224	MIMAT0000281	caagucacuagugguuccguuua	
hsa-miR-296	MIMAT0000690	agggcccccccucaauccugu	
hsa-miR-299	MIMAT0002890	ugguuuaccgucccacauacau	
hsa-miR-301	MIMAT0000688	cagugcaauaguauugucaaagc	
hsa-miR-302a	MIMAT0000684	uaagugcuuccauguuuugguga	
hsa-miR-302b	MIMAT0000715	uaagugcuuccauguuuuaguag	
hsa-miR-302b*	MIMAT0000714	acuuuaacauggaagugcuuucu	
hsa-miR-302c	MIMAT0000717	uaagugcuuccauguuucagugg	
hsa-miR-302c*	MIMAT0000716	uuuaacaugggguaccugcug	
hsa-miR-302d	MIMAT0000718	uaagugcuuccauguuugagugu	
hsa-miR-320	MIMAT0000510	aaaagcugguugagagggcgaa	
hsa-miR-323	MIMAT0000755	gcacauuacacggucgaccucu	
hsa-miR-324-5p	MIMAT0000761	cgcauccccuagggcauuggugu	
hsa-miR-325	MIMAT0000771	ccuaguagguguccaguaagu	
hsa-miR-326	MIMAT0000756	ccucugggcccuuccuccag	
hsa-miR-328	MIMAT0000752	cuggcccucucugcccuuccgu	
hsa-miR-330	MIMAT0000751	gcaaagcacacggccugcagaga	
hsa-miR-331	MIMAT0000760	gccccugggccuauccuagaa	
hsa-miR-335	MIMAT0000765	ucaagagcaauaacgaaaaaugu	
hsa-miR-337	MIMAT0000754	uccagcuccuauaugaugccuuu	
hsa-miR-338	MIMAT0000763	uccagcaucagugauuuuguuga	
hsa-miR-339	MIMAT0000764	ucccuguccuccaggagcuca	
hsa-miR-340	MIMAT0000750	uccgucucaguuacuuuauagcc	
hsa-miR-342	MIMAT0000753	ucucacacagaaaucgcacccguc	
hsa-miR-367	MIMAT0000719	aauugcacuuuagcaaugguga	
hsa-miR-368	MIMAT0000720	acauagaggaaauuccacguuu	
hsa-miR-370	MIMAT0000722	gccugcugggguggaaccugg	
hsa-miR-371	MIMAT0000723	gugccgccaucuuuugagugu	
hsa-miR-372	MIMAT0000724	aaagugcugcgacauuugagcgu	
hsa-miR-373	MIMAT0000726	gaagugcuucgauuuuggggugu	
hsa-miR-373*	MIMAT0000725	acucaaaaugggggcgcuuucc	
hsa-miR-374	MIMAT0000727	uuauaauacaaccugauaagug	
ath-miR159a	MIMAT0000177	uuuggauugaagggagcucua	*A. thaliana* negative contr
cel-lin-4	MIMAT0000002	ucccugagaccucaaguguga	*C. elegans* negative contr
cel-miR-2	MIMAT0000004	uaucacagccagcuuugaugugc	*C. elegans* negative contr
