# Supplementary material for: Distinctive Patterns of MicroRNA Expression Associated with Karyotype in Acute Myeloid Leukaemia
Source: PLoS One. 2008 May 14;3(5):e2141. doi: 10.1371/journal.pone.0002141 (PMC2373886; doi:10.1371/journal.pone.0002141)
Supplement: Table S2 — AML patient details. Sex, age at diagnosis, percentage of blasts, morphological FAB subtype, and karyotype are reported. (BM = bone marrow; PB = peripheral blood; MDS = myelodysplastic syndrome; RAEB = refractory anemia with excess blasts). (0.05 MB PDF) [file pone.0002141.s002.pdf]

Table S2. AML patient details.

Sample	Sex	Age (diagnosis)	Sample type	Percentage blasts	FAB Type	Karyotype
1	F	24	PB	98	M2	46,XX,t(1;6)(p36;p23),t(8;21)(q22;q22)/46,idem,der(22)t(1;22)(q23;p11.2)/47,idem,+8
2	F	38	PB	70	M2	46,XX,t(8;21)(q22;q22)
3	F	58	PB	nd	M3	46,XX,t(15;17),(q22;q21)
4	M	36	PB	85	M3	46,XY,t(15;17)(q22;q21)
5	F	62	PB	nd	M5	46,XX
6	F	35	PB	87	M5	47,XX,+8,der(9)del(9)(p12p22)t(9;11)(p22;q23)
7	M	47	BM	33	M2	45,XY,-7
8	M	36	BM	95	M0	47,XY,+19 [ish 9q34(ABLx1)]
9	M	67	BM	60	M2	46,XY,t(8;21)(q22;q22)
10	F	67	BM	47	M4Eo	46,XX,inv(16)(p13q22)
11	M	63	PB	84	M5	43,XY,-5,add(7)(q22),add(16)(q22),-17,-21,-22,+mar/42,idem,-18/43,idem,i(8)(q10)/42,idem,dic(11;15)(p11.2;p11.2)/43,idem,r(11)/43,idem,-18,add(20)(q1),+add(21)(q22)
12	F	76	PB	nd	M1	G-banding FAILED [BCR/ABL+ve]
13	F	70	PB	nd	M4	46,XX,i(4)(p10)x2
14	F	46	PB	nd	M2	46,XX
15	M	35	PB	70	M4	48,XY,+3,+10
16	M	60	BM	62	M2	46,XY
17	F	64	PB	80	M2	48,XX,?+X,del(7)(q22q36),+21/46,X,idic(X)(q11)/45,X,-X/46,XX
18	M	37	PB	95	M1	46,XY
19	M	51	BM	57	M2	46,XY,del(7)(q32q36),t(8;21)(q22;q22)
20	M	67	BM	97	M1	47,XY,+13
21	F	57	PB	75	M2	46,XX
22	F	64	PB	77	M4	46,XX,inv(1)(p32q32),t(11;17)(q23;q25)/52,idem,+5,+6,+9,+13,+13,+19/79,XXX,inv(1)(p32q32),+2,+3,+5,+6,-7,+8,+10,t(11;17)(q23;q25),+12,+13,+18,+19,+21/92,idemx2
23	M	45	PB	95	M4	46,XY,dir ins (6;11)
24	F	36	PB	70	M4Eo	47,XX,inv(16)(p13q22),+22
25	F	24	BM	80	M1	46,XX,t(6;9)(p22.3;q34)/46,idem,del(7)(q35)
26	F	51	BM	90	M1	46,XX,del(9)(q12q31)/46,XX
27	F	62	PB	100	M1	G-banding FAILED [BCR/ABL-ve]
28	M	52	PB	95	M1	46,XY,t(7;?),+t(7;22),+11,del(17)(p12),der(17;22)(q10;q10)/46,XY
29	M	57	BM	nd	M3	46,XY,t(15;17),(q22;q21)
30	F	nd	BM	nd	M4	46,XX
31	F	41	BM	90	M4	46,XX

Table S2. AML patient details.

32	M	23	PB	92	M2	48,XY,+8,+11,+13
33	F	55	BM	89	M5	46,XX,t(9;11)(p22;q23)
34	M	68	BM	50	M2	45,X,-Y,t(8;21)(q22;q22)
35	F	43	PB	98	M1	G-banding FAILED [BCR/ABL-ve]
36	M	76	PB	97	M1	46,XY
37	M	40	BM	nd	M2	46,XY,t(2;3)
38	M	18	PB	50	M2	45,X,-Y,t(8;21)(q22;q22)
39	F	27	BM	88	M2	45,X,-X,t(8;21)(q22;q22)
40	F	63	PB	75	M1	46,XX
41	F	67	PB	nd	M6	46,XX
42	F	11	BM	nd	M3	46,XX,t(1;10)(p32;p11.2),t(15;17)(q22;q21)
43	F	50	PB	98	M1	47,XX,dic(7;22)(q11.2;q10),+8
44	F	57	BM	72	M4	46,XX,inv(16)(p13q22)/46,idem,del(7)(q21q36)
45	F	59	PB	54	M4	46,XX
46	M	nd	PB	87	M4	46,XY
47	M	59	PB	80	M5	46,XY
48	F	49	PB	73	M5	47,XX,+11
49	F	78	BM	65	M4	46,XX
50	M	50	BM	74	M1	46,XY,t(9;22)(q34;q11.2)
51	M	56	PB	53	M2	46,XY
52	M	34	BM	52	M5	46,XY
53	M	26	BM	85	M1	47,XY,+8
54	M	41	PB	63	M5	46,XY,der(12)t(1;12)(q12;p11.2)/46,XY
55	F	65	PB	30	M1	47,XX,+8
56	F	22	BM	87	M1	46,XX,t(8;21)(q22;q22)
57	M	52	PB	61	M4	46,XY
58	M	52	PB	53	M2	46,XY,t(6;9)(p23;q34)
59	M	65	BM	nd	M4Eo	46,XY,inv(16)(p13q22)
60	M	50	BM	98	M1	46,XY
61	M	36	PB	90	M5	46,XY
62	M	46	BM	90	M4	46,XY
63	F	83	UN	nd	M4	46,XX
64	F	52	PB	90	M4	46,XX
65	M	70	PB	90	M5	47,XY,+8
66	M	61	BM	73	M2	45,X,-Y,t(8;9)(p22;p24),add(14q32)
67	M	20	PB	nd	M2	46,XY,t(8;21)(q22;q22)
68	F	68	BM	nd	M1	46,XX
69	M	18	BM	80	M2	46,XY
70	F	72	PB	75	M1	46,XX
71	F	65	BM	93	M1	46,XX
72	F	62	BM	nd	MDS	47,XX,+8

Table S2. AML patient details.

73	F	69	BM	88	M1	46,XX
74	F	40	PB	95	M4Eo	47,XX,inv(16)(p13q22),+22
75	F	88	PB	44	MDS	47,XX,+8
76	M	6	UN	nd	M4Eo	46,XX,inv(16)(p13q22)
77	M	75	PB	92	M4	46,XY
78	M	55	PB	35	M4	47,XY,inv(16)(p13q22),+22
79	F	44	PB	56	M2Eo	46,XX
80	M	27	PB	80	M3	46,XY,t(15;17)(q22;q21)/47,idem,+8
81	M	28	PB	25	M2	46,XY,del(5)(q31)/46,XY
82	M	53	BM	nd	M2	45,X,-Y,t(8;21)(q22;q22)
83	F	87	BM	nd	M1	46,XX
84	M	67	PB	30	M2Eo	46,XY,t(8;21)(q22;q22)
85	M	30	PB	95	M3	G-banding FAILED [PML/RARA+ve]
86	M	28	PB	97	M4Eo	47,XY,inv(16)(p13q22),+22
87	F	37	PB	70	M5	46,XX,der(2)t(2;10)(p23;p12)ins(10;11)(p12;q23q13),der(10)t(2;10),der(11)ins(10;11)(p12;q23q13)/47,idem,+3
88	M	34	UN	nd	M5	46,XY,t(6;11)(q27;q23)
89	F	29	BM	76	M1	46,XX
90	F	64	PB	85	M5	46,XX
91	M	51	PB	35	M2	46,XY
92	M	55	UN	nd	M4Eo	46,XY,inv(16)(p13q22)
93	M	30	PB	83	M3	46,XY,t(15;17)(q22;q21)/46,XY
94	M	55	BM	nd	M3	G-banding FAILED [PML/RARA+ve]; Reclassified as MDS-RAEB: 46,XY,t(12;17)(p13;q22)
95	F	68	BM	98	M1	46,XX
96	M	60	PB	93	M3	46,XY,t(15;17),(q22;q21)
97	F	48	PB	80	M5	46,XX
98	M	52	PB	90	M4	46,XY,t(6;11)(q27;q23)
99	F	64	PB	60	M5	46,XX
100	M	43	PB	nd	M5	47,XY,+8
104	F	17	PB	95	M3	46,XX,t(15;17)(q22;q21)
105	M	90	PB	90	M3	46,XY,t(15;17)(q22;q21)
106	M	41	BM	96	M3	46,XY,t(9;14),t(15;17)(q22;q21)
107	M	42	BM	95	M3	46,XY,t(15;17)(q22;q21),ider(17)(q10)t(15;17)
108	M	68	BM	85	M3	46,XY,t(15;17)(q22;q21)
109	M	35	BM	90	M3	46,XY,t(15;17)(q22;q21)
110	M	57	BM	nd	M3	46,XY,t(15;17)(q22;q21),add(19)(p13)
111	M	67	BM	nd	Biphenotypic	46,XY,t(9;22)(q34;q11)
112	F	66	BM	nd	M0	46,XX
113	F	57	BM	nd	M2	46,XX,t(8;21)
