# Supplementary material for: Distinctive Patterns of MicroRNA Expression Associated with Karyotype in Acute Myeloid Leukaemia
Source: PLoS One. 2008 May 14;3(5):e2141. doi: 10.1371/journal.pone.0002141 (PMC2373886; doi:10.1371/journal.pone.0002141)
Supplement: Table S3 — Real-time PCR results, raw data. The spreadsheet contains the median Ct values of 157 miRNAs quantified in 100 AML samples, 2 cell lines (NB4 and KG1), and 2 normal bone marrow controls (NBM4 and NBM5). (0.25 MB PDF) [file pone.0002141.s003.pdf]

Table S3. Median Ct values of plates A and B.

Plate	Samples	1	2	3	4	5	6	7	8	9	10
A	hsa-miR-9	34.9783	35.6797	30.1885	32.7076	29.8409	23.9239	34.8430	31.9244	33.3149	31.8323
A	hsa-miR-9*	37.2863	37.9126	34.1187	35.8100	33.5623	28.7491	38.0976	34.3691	36.4485	34.4704
A	hsa-miR-10a	35.0888	36.5180	34.8274	32.5292	31.4806	35.1300	38.1018	33.4978	34.0708	31.0636
A	hsa-miR-15a	26.2322	23.0469	26.8109	22.5980	24.1780	24.6167	25.4030	23.0434	23.1179	23.7662
A	hsa-miR-15b	24.2748	24.0699	25.0942	22.2712	22.6541	23.3785	23.6407	22.8508	21.9145	22.4384
A	hsa-miR-16	21.5723	21.9800	22.1580	20.4261	20.9607	21.3881	21.1050	20.4989	19.2264	20.2817
A	hsa-miR-17-3p	26.2369	28.0720	29.0647	26.8624	27.2803	27.7006	26.8675	26.9887	25.9249	26.9701
A	hsa-miR-17-5p	22.0231	25.8654	23.8568	21.7065	22.1830	22.5874	21.6993	22.8951	21.8267	21.9604
A	hsa-miR-19a	22.2286	22.8650	22.5163	21.0478	20.8206	21.2354	22.7928	20.5524	22.2277	21.1310
A	hsa-miR-20	24.2904	23.8392	24.5764	21.7308	21.9677	23.5979	24.6251	21.8112	22.7702	22.6220
A	hsa-miR-21	23.2294	21.4394	21.7666	19.4308	19.3438	20.3643	23.5248	19.4839	19.8227	19.9208
A	hsa-miR-23a	24.9190	23.9306	25.3272	21.8322	21.9051	22.1523	23.8551	21.7382	21.6620	21.8571
A	hsa-miR-23b	29.1471	28.3102	29.4586	26.0486	26.9645	27.1054	26.0881	26.4605	27.2674	26.9461
A	hsa-miR-25	23.7106	23.9204	24.6673	23.1665	24.0301	22.8896	24.9863	20.8574	21.7849	22.4205
A	hsa-miR-26a	21.9393	22.6200	24.1978	20.9490	21.7341	23.1196	22.3306	21.7405	20.9525	21.5355
A	hsa-miR-26b	23.3081	23.9135	25.6764	22.2706	23.1916	23.9552	24.5229	22.7603	22.7049	23.1008
A	hsa-miR-27a	24.9841	27.2863	25.4336	24.3838	23.6775	23.5255	24.3537	23.9405	23.5084	23.7514
A	hsa-miR-27b	27.9594	27.4674	28.9546	25.5560	25.4500	28.1054	25.3458	25.4622	25.3539	26.4187
A	hsa-miR-28	30.6017	27.9834	29.0849	26.9350	26.7593	28.1347	28.9080	25.9562	26.9029	26.9456
A	hsa-miR-29a	25.2950	23.7872	22.7789	21.9961	22.6664	25.1333	23.9761	22.4689	21.0094	22.5551
A	hsa-miR-29b	26.1102	25.3885	24.9764	23.6855	24.6300	27.3563	25.4032	23.9804	22.5603	24.0622
A	hsa-miR-29c	23.7003	22.8774	22.9324	20.9080	22.1051	24.4061	22.9759	20.5867	20.1733	21.4866
A	hsa-miR-30a-3p	29.8445	31.1040	29.1737	27.6237	27.2599	28.3653	29.1783	25.6522	26.7684	26.7842
A	hsa-miR-30b	23.1792	22.6980	23.7913	21.3167	21.9688	22.1867	22.5261	20.7573	21.1133	20.9758
A	hsa-miR-30c	23.3209	23.5660	24.1630	22.1312	23.4893	23.1964	23.7080	21.5822	21.9449	22.3828
A	hsa-miR-30d	25.3735	24.5094	26.4109	23.6028	24.0290	24.3709	24.5397	23.0826	23.0218	23.4317
A	hsa-miR-30e	25.4337	25.6053	26.9061	24.9495	25.6103	26.1615	25.1930	23.4805	23.3098	24.6671
A	hsa-miR-31	33.4276	34.2156	40.0000	32.6191	38.3705	34.3585	31.3304	34.1916	29.1367	33.6080
A	hsa-miR-34a	26.1585	26.8073	27.3203	24.0830	23.4981	25.5466	26.8828	24.8545	26.6098	26.6737
A	hsa-miR-34b	40.0000	37.0416	37.0600	33.9319	36.7008	40.0000	37.2319	37.6785	34.7118	37.9420
A	hsa-miR-34c	40.0000	35.7628	37.2321	34.2420	36.2999	40.0000	35.9541	37.0763	40.0000	37.3559
A	hsa-miR-92	23.8462	21.3497	23.0140	20.2381	20.8453	23.9878	21.3456	20.2665	21.2074	20.7410
A	hsa-miR-95	37.3646	34.3267	38.0960	32.3942	34.0118	37.9917	33.8525	33.0374	34.1307	34.2115
A	hsa-miR-96	37.3277	40.0000	37.3070	36.0353	34.4581	38.3464	32.9327	36.0758	40.0000	37.3136
A	hsa-miR-98	28.0509	27.6373	28.4889	24.9656	25.9987	28.0397	27.2370	27.2217	27.6244	26.1561
A	hsa-miR-99a	28.6576	33.0593	26.7188	24.9584	30.1574	33.8243	26.0206	34.3414	28.6216	26.6798
A	hsa-miR-100	28.6210	31.2487	25.8104	24.5571	29.8143	33.1565	26.0697	33.4496	27.9579	26.2868
A	hsa-miR-103	23.9504	23.6549	25.2255	22.2198	22.6414	23.1881	23.4212	22.7784	21.6274	22.2738
A	hsa-miR-104	37.6644	40.0000	36.5586	35.2741	34.9018	35.2676	37.5842	34.9049	36.0075	36.2629
A	hsa-miR-105	38.3539	40.0000	38.0861	36.5329	37.0464	38.2716	38.1506	36.3746	40.0000	38.3741
A	hsa-miR-106a	23.8755	25.7514	24.5287	22.7972	22.1662	23.7407	23.2443	22.7386	23.3043	22.8920
A	hsa-miR-107	30.0823	30.8338	32.3672	29.2581	28.9973	29.3948	31.5712	28.4703	29.9916	28.6497
A	hsa-miR-122a	38.5521	38.3305	40.0000	37.1116	39.3781	38.9177	40.0000	39.7072	37.1919	40.0000
A	hsa-miR-124a	37.8423	35.2346	36.7910	33.5736	32.8565	37.0496	34.8970	34.9810	37.0990	36.8299
A	hsa-miR-124b	32.4107	33.9217	29.9675	25.2397	28.3326	30.4596	26.5816	31.9358	28.7720	29.4465
A	hsa-miR-125a	23.0464	25.9412	27.6842	26.3750	26.8505	31.4465	26.5879	23.4207	27.6491	25.2510
A	hsa-miR-125b	36.3829	30.9786	38.5128	24.2663	30.7555	37.6317	26.4131	34.2416	40.0000	34.8683
A	hsa-miR-126	22.0839	20.8027	29.2238	25.8537	27.5043	32.9149	24.6996	28.5053	20.8797	22.7343
A	hsa-miR-127	40.0000	31.9708	34.5348	25.9405	35.0262	40.0000	38.6046	34.0568	40.0000	40.0000
A	hsa-miR-128a	32.5016	32.7443	30.4462	27.8281	29.7552	30.9860	31.5827	24.2003	26.4255	30.5971
A	hsa-miR-128b	33.9286	35.9281	30.5293	29.4242	32.1596	32.7374	32.8561	25.4145	27.3137	32.4579
A	hsa-miR-129	37.4970	36.8033	37.2459	33.3510	35.0605	35.2349	34.9872	34.1916	37.3908	36.9302
A	hsa-miR-130a	24.1148	22.6004	25.7713	22.1896	27.4265	32.6922	23.8022	22.5680	21.7884	23.0175
A	hsa-miR-130b	25.3183	25.6430	26.3251	24.8108	25.4756	25.5016	25.6008	23.5995	24.1261	25.1541
A	hsa-miR-132	29.4812	30.1738	27.5183	28.9091	30.4059	28.6017	29.7796	28.7999	26.9484	28.2085
A	hsa-miR-133a	33.4342	34.2217	34.6540	33.2106	32.1435	34.8160	33.0701	26.2692	32.7543	30.4946
A	hsa-miR-133b	32.5167	33.1929	34.6883	32.1362	32.2194	35.2563	32.4816	26.6981	33.1752	31.1743
A	hsa-miR-134	34.7740	35.7805	30.7210	28.9651	37.0585	36.2035	36.9095	35.9223	36.7620	36.7710
A	hsa-miR-135a	30.8974	35.5344	37.5788	33.7117	37.3986	40.0000	33.7156	26.6090	34.4557	32.9282
A	hsa-miR-135b	35.1714	35.0345	28.9280	34.8938	37.1295	38.5592	32.9047	31.1208	34.6161	35.1492
A	hsa-miR-137	40.0000	37.9563	7.4971	40.0000	34.8600	40.0000	38.0438	38.1197	39.7127	38.8050
A	hsa-miR-138	40.0000	39.2217	36.2108	35.2991	38.7126	37.6206	37.1789	35.9275	37.0248	39.0735
A	hsa-miR-139	34.3043	31.9057	35.5618	33.0198	33.1262	34.4865	34.3913	34.0416	29.3071	30.1052
A	hsa-miR-140	25.8738	25.4837	26.5123	24.8406	24.6422	24.8669	24.9697	23.5437	23.5638	24.1155
A	hsa-miR-141	32.0677	31.8090	33.3402	31.0918	30.5116	30.1943	31.6439	28.4261	29.8320	30.7278
A	hsa-miR-142-3p	19.0533	20.5720	20.2862	18.7251	19.2533	19.2419	19.3911	17.7628	18.4407	18.6935
A	hsa-miR-142-5p	24.1791	24.3675	25.6330	22.9950	23.6740	25.5658	23.7503	22.2495	24.4173	23.3555
A	hsa-miR-144	40.0000	40.0000	39.4042	38.3978	38.8380	40.0000	35.0792	37.0581	37.0242	32.8838

Table S3. Median Ct values of plates A and B.

A	hsa-miR-145	27.6499	29.7244	29.1390	26.8531	31.8229	26.4236	29.6612	30.2549	27.8910	28.2106
A	hsa-miR-146	21.9945	22.0847	23.4719	21.5766	23.8786	27.1463	22.4414	19.9674	19.6062	22.5574
A	hsa-miR-147	39.6481	39.0043	38.2797	36.7122	35.6944	37.7191	37.6330	36.2271	36.1823	37.5719
A	hsa-miR-148a	33.3772	31.0637	29.5442	25.1133	30.2113	28.3566	29.6046	29.9308	29.7703	29.3411
A	hsa-miR-149	34.8834	32.8958	33.2796	33.3400	31.4209	32.2236	35.0666	31.2111	32.5476	32.6189
A	hsa-miR-150	25.0512	23.4945	25.4975	22.2578	25.2593	25.9773	22.3126	21.6061	20.0961	23.6572
A	hsa-miR-151	31.9350	30.8772	35.0455	32.7722	31.4284	30.7972	31.3171	32.3597	31.3216	29.9154
A	hsa-miR-152	30.9638	32.2218	31.1434	29.0033	31.0801	28.6867	30.6045	31.1277	29.8767	29.5874
A	hsa-let-7a	22.7351	25.1946	25.2748	21.1078	22.6303	22.9378	22.7363	22.4958	22.5733	23.2532
A	cel-lin-4	40.0000	39.5286	38.8749	38.7588	40.0000	40.0000	40.0000	40.0000	40.0000	39.0540
A	ath-miR159a	37.1386	40.0000	37.4692	34.7560	36.0791	39.0106	36.9772	36.7825	36.7228	38.2137
B	hsa-miR-16	21.0637	21.9398	22.0534	20.5811	20.9100	20.3685	21.1126	20.4054	18.7875	20.3392
B	hsa-miR-154	35.5103	37.0945	29.2430	29.0288	40.0000	36.6667	39.5816	35.2375	34.0150	37.5786
B	hsa-miR-154*	33.5303	34.6797	29.0692	28.0771	37.1843	36.1800	37.8782	35.1691	33.6407	37.7436
B	hsa-miR-155	25.0017	25.9871	25.9015	23.9093	23.6681	25.8352	25.1831	25.3416	23.5800	25.0127
B	hsa-miR-181a	20.7942	21.7287	20.3715	19.2541	21.1893	22.7980	20.2335	17.5633	17.5347	20.6647
B	hsa-miR-181b	21.8735	21.3440	21.3439	19.7901	21.6942	23.4630	22.2272	18.7631	18.6443	21.6613
B	hsa-miR-181c	25.8148	28.0032	26.1147	24.2973	26.5884	28.2213	26.4582	23.5526	23.0785	26.4115
B	hsa-miR-182	33.0952	38.3283	35.7644	34.8029	33.6012	35.2522	29.3125	32.7932	31.8431	34.4103
B	hsa-miR-182*	37.0444	40.0000	36.1040	35.0798	37.0457	35.4487	35.7031	37.1262	33.5751	36.4704
B	hsa-miR-183	38.8513	40.0000	40.0000	38.7009	37.7674	39.6251	36.1915	40.0000	37.7493	40.0000
B	hsa-miR-184	38.8169	36.4807	37.3606	35.2706	38.2577	38.4915	37.7950	39.1979	38.1332	39.7931
B	hsa-miR-185	30.7593	32.3345	31.5506	29.8837	29.8231	28.9500	30.5555	29.8765	27.3146	28.9985
B	hsa-miR-186	25.2497	25.7371	25.2840	23.7668	23.9985	23.4830	25.1769	24.0462	22.1667	23.7389
B	hsa-miR-187	38.0995	35.5745	39.1577	38.0290	34.8949	32.2054	36.3554	36.2387	35.2192	34.7433
B	hsa-miR-189	37.6368	37.0553	36.7855	35.6147	39.4260	37.9879	35.9139	37.5252	35.7002	36.7047
B	hsa-miR-190	36.5068	35.2128	35.9877	35.0403	32.5984	32.1820	33.6752	32.2472	33.7785	36.2511
B	hsa-miR-191	21.6939	23.1080	23.7438	22.0822	22.5140	20.3253	23.4436	21.0541	19.7123	21.5618
B	hsa-miR-193	30.2330	28.6492	30.8630	29.2907	31.6179	30.1217	33.0733	30.1618	28.4540	28.6618
B	hsa-miR-194	25.6634	28.3029	30.0067	28.8728	29.5032	27.7118	27.5511	26.1599	25.4472	28.8310
B	hsa-miR-195	26.3797	28.6968	27.6628	25.0820	26.1189	27.1212	27.9042	26.5101	24.9946	27.1400
B	hsa-miR-197	25.1493	27.5925	27.4964	27.8324	26.0132	25.6905	25.9085	25.3440	22.8060	25.0240
B	hsa-miR-198	36.4134	39.2145	38.3916	35.7593	38.3250	37.8199	37.8349	36.3433	35.5643	39.6197
B	hsa-miR-199a	35.8413	36.5635	34.0292	32.9603	34.4241	34.0353	34.8719	32.3721	30.7540	32.6000
B	hsa-miR-199a*	25.5909	25.2151	25.0068	23.4825	40.0000	25.7436	25.2508	25.3350	21.4816	24.8908
B	hsa-miR-199b	27.8729	26.9016	25.8038	24.1523	26.1074	26.4166	26.9976	26.6354	23.2973	24.9649
B	hsa-miR-199-s	32.0252	33.6614	30.7870	29.4826	30.5106	31.3104	31.1062	30.7123	26.1039	29.3112
B	hsa-miR-200a	33.9273	32.9942	33.8621	31.7445	34.0436	30.8865	33.2927	34.7313	29.5777	32.3681
B	hsa-miR-200b	33.8986	35.7008	34.3043	32.3194	34.7756	31.5096	34.2493	34.9078	30.2049	33.3474
B	hsa-miR-200c	29.9574	32.4538	31.6175	29.9235	30.0159	28.3678	31.1034	28.5626	26.8048	29.6998
B	hsa-miR-203	32.9562	33.5242	33.3585	30.0992	33.5141	36.3476	35.0778	33.8363	33.2917	37.4431
B	hsa-miR-204	32.6220	38.6737	30.7786	31.4547	29.7740	33.6096	33.5442	32.2425	29.6370	35.9005
B	hsa-miR-205	40.0000	38.2521	40.0000	40.0000	39.7201	36.6417	37.0883	37.8080	35.2854	36.7817
B	hsa-miR-210	30.5293	30.2641	28.5634	27.5914	26.5960	29.4999	29.6660	27.0020	27.3397	28.7551
B	hsa-miR-211	36.5228	40.0000	35.1818	34.9274	38.6999	36.8282	38.2753	37.5728	37.6095	40.0000
B	hsa-miR-213	27.0443	27.4059	26.0801	24.2091	26.7006	27.8878	25.9869	22.9437	25.8092	25.8504
B	hsa-miR-214	36.6585	35.6973	34.9066	30.9636	34.0125	34.0422	33.6926	32.2913	31.2493	33.8180
B	hsa-miR-215	29.1626	33.8683	33.7297	31.4683	33.0375	31.1738	31.5410	29.6403	28.9114	33.1927
B	hsa-miR-216	39.8125	40.0000	38.7532	36.7288	38.2129	40.0000	39.0230	38.5274	39.3124	40.0000
B	hsa-miR-218	35.0800	36.7842	34.3733	34.8692	34.5207	36.7315	35.7062	33.2824	34.6595	38.2675
B	hsa-miR-219	32.7432	35.1643	34.8637	33.7830	34.3080	33.4047	32.7828	32.3155	31.7376	33.9584
B	hsa-miR-220	40.0000	40.0000	40.0000	38.3654	40.0000	40.0000	40.0000	40.0000	39.0191	40.0000
B	hsa-miR-221	23.1811	25.1959	23.2916	22.1692	23.7383	21.1358	22.6493	23.7580	21.8809	21.9359
B	hsa-miR-222	22.2242	24.9279	23.4290	22.4979	23.6171	21.5549	22.3997	24.2893	20.8718	21.6545
B	hsa-miR-223	20.2800	20.6132	22.3804	21.4038	20.1276	19.7541	20.1246	22.2528	18.3114	19.1845
B	hsa-miR-224	37.4984	39.1767	28.2668	27.3986	35.9719	37.4551	34.3599	35.3075	36.7773	30.3689
B	hsa-miR-296	31.2894	33.9114	32.5605	30.4732	29.8784	30.1391	30.0630	32.6971	29.6874	32.2560
B	hsa-miR-299	34.9814	35.6813	29.4652	28.4760	40.0000	37.6763	38.0371	36.7669	33.6722	37.7515
B	hsa-miR-301	26.9667	29.0199	28.5340	27.1825	26.8540	26.1087	28.0152	27.1843	27.0264	26.0515
B	hsa-miR-302a	37.3797	40.0000	40.0000	37.9981	38.0609	39.5667	37.7324	37.2216	37.1670	39.7984
B	hsa-miR-302b	38.3277	40.0000	40.0000	40.0000	37.5360	39.3970	40.0000	40.0000	38.6573	40.0000
B	hsa-miR-302b*	40.0000	40.0000	39.1937	40.0000	40.0000	39.9390	40.0000	40.0000	40.0000	39.8814
B	hsa-miR-302c	39.7330	40.0000	40.0000	40.0000	39.0645	40.0000	40.0000	40.0000	36.1051	37.8942
B	hsa-miR-302c*	40.0000	40.0000	38.9750	38.0056	40.0000	38.4381	39.6968	40.0000	37.9267	37.2771
B	hsa-miR-302d	40.0000	40.0000	40.0000	37.3015	38.5772	39.9701	38.9078	40.0000	36.9848	40.0000
B	hsa-miR-320	23.2956	22.9578	24.6334	23.4568	23.1834	22.7994	23.6075	22.4244	20.0071	22.3703
B	hsa-miR-323	34.3803	35.9380	30.8425	29.0479	37.3127	36.5486	38.8660	36.1986	34.5461	37.6530
B	hsa-miR-324-5p	28.0249	27.4817	28.4489	26.5773	26.7341	25.6761	27.9187	26.3294	24.7504	26.4030
B	hsa-miR-325	40.0000	40.0000	39.0611	38.2712	35.5966	38.0661	37.3844	38.9927	40.0000	40.0000
B	hsa-miR-326	30.6477	30.5347	32.7047	30.4430	29.6194	29.6778	31.1453	29.9008	28.4752	30.3441

Table S3. Median Ct values of plates A and B.

B	hsa-miR-328	26.9243	28.4814	29.5667	27.5902	27.1759	25.9353	28.3788	27.3047	24.4216	27.0684
B	hsa-miR-330	31.9298	33.3983	35.2662	33.6632	31.8282	31.2153	33.9412	32.4854	29.9361	32.2587
B	hsa-miR-331	26.2471	27.7977	28.6001	26.5875	26.5104	26.7748	27.0953	25.0254	24.8381	26.2000
B	hsa-miR-335	25.6711	26.6025	27.3148	25.6524	28.8190	26.9039	30.3647	29.0957	24.3116	24.9301
B	hsa-miR-337	39.1344	40.0000	35.2321	33.3697	40.0000	40.0000	39.3314	40.0000	38.7497	40.0000
B	hsa-miR-338	31.3715	31.3516	32.7394	30.9560	32.6935	35.1145	32.1591	32.9282	32.4650	30.9409
B	hsa-miR-339	27.3660	28.2544	28.9742	27.2457	26.1088	25.7767	29.6655	25.3483	25.8961	26.1156
B	hsa-miR-340	28.6554	30.5613	29.2682	27.4907	27.8431	29.1232	29.0658	28.3073	26.0495	27.5874
B	hsa-miR-342	25.9081	25.8061	25.7226	23.9953	25.9367	23.4129	23.5797	22.5440	22.6812	24.3156
B	hsa-miR-367	38.1998	40.0000	40.0000	39.9078	38.8258	38.5549	37.7291	40.0000	37.2996	40.0000
B	hsa-miR-368	34.4466	36.3063	28.9259	28.9798	40.0000	37.3028	37.5651	35.6153	35.7811	37.5278
B	hsa-miR-370	34.6936	37.6826	30.8708	28.5457	36.7704	35.0102	36.5615	35.2300	34.3820	35.9388
B	hsa-miR-371	37.6083	40.0000	33.7771	40.0000	39.1388	40.0000	39.1032	40.0000	32.0990	40.0000
B	hsa-miR-372	40.0000	40.0000	37.4404	40.0000	39.6457	37.9139	39.6813	38.0910	37.5834	40.0000
B	hsa-miR-373	35.1908	40.0000	34.0498	35.0680	31.5994	31.9134	34.0913	33.6492	29.9067	35.8876
B	hsa-miR-373*	40.0000	40.0000	37.3358	37.0042	35.5892	38.0234	36.5817	36.4990	32.5257	37.8567
B	hsa-miR-374	27.9766	28.4189	28.1611	25.7708	25.8985	27.3321	27.0616	26.6394	25.4704	26.0308
B	hsa-let-7a	24.5331	25.7194	23.9969	21.1156	22.2894	22.9514	22.4605	23.4339	20.7703	22.8981
B	hsa-let-7b	24.7520	25.4648	25.9052	22.6230	21.6836	21.1599	24.0464	28.9484	21.2729	23.5619
B	hsa-let-7d	24.2271	25.8970	26.4499	20.9032	22.5601	22.8747	25.4468	23.2791	23.2680	22.6803
B	hsa-let-7e	28.8315	31.3022	33.0462	29.6748	31.4440	33.7342	32.0276	29.1442	31.4446	30.6331
B	hsa-let-7g	23.3780	24.2757	24.5991	21.9755	23.2452	24.3593	22.6034	22.7586	21.8707	22.9292
B	has-let-7i	25.2599	26.1696	27.6720	23.9833	25.3892	26.1475	25.4574	25.3949	23.7185	25.1724
B	cel-miR-2	39.1707	40.0000	38.3608	37.1454	40.0000	39.6433	39.4530	40.0000	37.3056	40.0000

Table S3. Median Ct values of plates A and B.

11	12	13	14	15	16	17	18	19	20	21	22	23
31.7420	29.4452	25.3916	37.4870	33.8269	31.0030	32.9212	32.0791	38.7428	31.7684	30.9087	25.6443	29.2329
35.2443	33.4123	29.5541	39.6883	36.9573	34.7957	38.0750	35.5059	40.0000	34.6735	34.5427	30.8788	33.4407
34.9328	22.6715	22.2923	35.5135	24.2180	22.9020	26.7822	22.9880	36.0256	23.5312	20.9985	34.4380	35.0603
21.8333	23.7648	23.1313	27.5640	24.9466	23.9428	24.2129	24.4684	30.2641	25.0266	23.7826	24.5251	26.3429
22.0519	23.0657	22.4389	27.7274	24.3406	22.4115	22.8145	23.2231	28.0754	24.2065	22.3744	23.0042	24.5970
19.8514	19.8821	19.6277	23.4887	21.3162	20.5285	20.7611	21.4684	24.0192	21.1425	19.9233	21.2202	21.4163
25.0216	26.3209	26.9226	29.8155	28.1984	27.6017	25.8930	27.8834	30.1144	27.5035	25.2721	27.5955	28.9040
21.1281	22.8298	22.1051	25.6787	22.5954	22.2854	21.3033	22.6470	25.4624	22.1844	22.1573	22.6536	24.9408
20.9753	24.4187	21.3594	25.9852	25.2162	21.7120	21.8671	21.9187	24.0206	21.5281	20.8471	22.4733	22.7145
23.0804	22.8902	22.8528	25.2910	25.3569	22.8610	23.4182	22.9551	26.9716	23.3515	20.7048	24.1259	24.8850
21.7149	21.0109	18.7350	23.9969	21.1166	20.2348	21.8603	20.9923	26.5307	21.3780	20.9067	22.4344	21.9960
22.0429	22.9466	21.9993	26.6007	23.7182	22.0381	24.5659	22.2754	28.4011	23.1773	21.9400	22.9128	24.6710
24.5312	27.9602	26.5151	30.9963	28.8047	26.7633	28.9439	27.6294	31.2152	28.9171	27.6535	28.0944	29.2378
22.8203	22.7229	23.0425	25.3293	24.4843	24.4603	24.7158	24.7570	25.7002	25.1315	21.7731	24.3384	24.5686
21.2242	22.3682	21.3284	26.1978	23.9815	22.5088	22.6973	22.5117	24.4227	23.2846	20.3155	22.5164	22.9626
22.7752	23.8697	22.6860	27.6901	25.9139	23.5775	24.6960	23.4721	25.4938	25.4058	23.1055	23.9396	24.0498
23.0308	23.3614	22.9134	28.4439	23.5024	23.6994	25.1471	24.1657	27.4604	23.6330	23.2778	22.8455	25.4366
24.7743	28.2408	25.6279	31.1544	29.5254	26.0783	27.5113	27.1677	29.9814	27.4981	25.3905	28.3738	28.4036
28.2203	27.4992	25.2046	31.0446	28.7743	27.5190	28.2468	27.4534	32.6760	28.6398	27.6156	28.2872	28.5637
24.9856	25.3251	21.0648	24.9492	24.7272	23.6818	25.0693	24.0577	26.6037	23.5051	22.6476	24.0862	23.1260
25.4078	29.5435	22.2147	30.3252	29.0351	24.9972	25.9162	25.0859	29.2564	24.8603	24.6793	25.3184	24.4777
21.9894	23.6401	20.8182	25.4543	23.5044	22.1423	22.9418	22.7000	27.1228	22.6426	21.5119	23.1584	22.7448
25.7359	27.5780	26.1144	30.3455	28.6675	26.8764	28.1656	27.6565	33.9211	28.3343	26.2647	28.3323	29.2907
21.2091	21.2706	20.9190	23.4752	22.8072	22.0623	22.4076	22.1021	24.4578	22.9730	20.6932	22.5407	23.0068
21.6635	24.5572	21.7869	26.8103	25.2962	23.2192	23.4511	23.5469	25.4971	24.2781	21.6102	23.3176	24.1510
23.1838	24.9221	23.5688	27.4526	25.2213	24.2674	24.5454	24.7311	26.5477	25.9604	23.6748	24.6234	25.2742
23.0664	23.9518	23.7600	27.0948	25.6965	26.1152	25.2918	26.0683	27.0001	25.9800	23.2422	26.3630	25.5668
38.6296	40.0000	28.4884	40.0000	37.0553	38.8042	40.0000	39.9439	40.0000	40.0000	36.5008	32.3587	30.7979
28.2982	27.0866	25.8511	29.5057	29.9789	23.6546	23.9457	25.5411	29.5228	24.9160	25.9772	28.2761	29.7573
33.2840	38.0193	37.9239	37.3264	40.0000	33.9738	36.8598	36.3350	37.8219	37.5055	40.0000	37.7008	35.9390
32.5093	40.0000	37.3555	40.0000	40.0000	34.2216	36.1315	36.3201	38.1059	40.0000	40.0000	40.0000	36.5617
19.6394	22.1456	20.1512	25.6270	24.0387	19.7434	19.8013	21.2266	24.0449	20.2555	18.9841	24.4394	22.5964
33.6800	36.6500	30.9232	38.1990	36.3187	35.4430	35.4648	34.9315	38.3492	36.1672	34.4023	34.8598	32.9132
35.6715	37.5025	37.6944	39.4063	37.7368	36.5495	34.0365	37.5520	39.2453	37.4178	37.3501	35.4609	34.8951
25.7906	28.0475	26.8729	30.5551	29.1012	26.2176	26.5696	27.3560	30.5092	28.7585	27.9508	27.9831	28.5387
22.9011	27.4394	28.0494	32.6652	29.7584	26.4705	22.5505	27.8616	32.5754	27.9587	26.0512	33.4531	29.8115
23.3255	26.4329	28.2746	31.8982	29.8295	27.2148	22.4996	27.8150	31.7082	27.9438	26.2668	32.8514	29.7226
22.6007	23.7253	22.1319	26.2248	24.5871	23.0266	22.5120	24.4762	25.9610	25.3589	23.9113	22.3628	24.5033
34.5462	40.0000	33.9743	40.0000	39.0047	37.7022	35.9079	36.3815	39.4037	36.9656	38.6778	36.9988	34.6108
37.0458	40.0000	37.7686	40.0000	40.0000	36.9096	37.8087	38.3735	38.3173	37.0438	38.1432	38.2208	38.3308
20.9335	24.4372	23.4242	27.8678	24.9208	23.0916	21.9536	23.2553	27.6709	23.3239	22.4156	24.1565	25.3354
27.7497	30.3186	28.9412	34.7838	32.0678	29.5729	29.3457	30.0493	36.2481	31.8252	30.1593	29.6481	30.7576
37.4010	40.0000	40.0000	40.0000	39.9996	39.9215	37.7642	40.0000	40.0000	40.0000	40.0000	37.8349	40.0000
33.7391	34.5463	35.9355	38.3070	37.8173	35.8795	33.8066	36.5557	38.0806	33.5356	35.0820	35.8105	37.3699
28.3382	32.6937	29.1427	32.4861	30.6624	28.0030	29.2071	30.1852	30.4138	30.2215	28.8323	28.4644	30.9760
22.1745	24.2843	26.8131	30.6983	31.3015	24.2264	24.5828	25.0875	30.0928	28.4890	25.8775	25.0472	26.2139
23.2618	36.5672	37.5468	36.7124	38.9654	28.1778	23.8662	28.3693	40.0000	28.2509	26.3518	40.0000	38.8505
22.8565	27.7781	25.7472	25.4807	31.0079	28.2110	26.1161	28.4683	24.9966	27.2782	27.1810	31.4124	26.8580
31.1540	40.0000	40.0000	40.0000	40.0000	35.8439	35.3319	38.8227	40.0000	37.4123	34.6409	40.0000	40.0000
29.0590	31.0509	29.9676	34.2811	33.4887	29.4432	30.8193	30.7958	34.5215	31.8950	29.9994	31.4844	32.7146
30.6259	32.8290	31.6179	35.4850	35.9676	30.9322	31.9448	31.4505	35.2320	33.6250	31.7069	32.7193	33.7919
33.6471	39.8505	35.4492	39.0598	38.8090	35.9917	35.7858	37.0014	38.7212	36.5311	36.0856	36.8991	39.7661
21.6077	23.4486	25.2988	25.0288	31.9225	25.4783	23.6912	25.6270	25.0824	26.2328	26.9409	29.5742	25.4458
24.6023	25.7260	25.6249	27.7282	27.3054	25.0913	24.8608	25.1806	27.6860	27.4293	26.3730	26.2077	26.4940
28.2624	29.2997	28.1551	31.8101	27.9889	30.7333	30.5628	30.9321	31.8673	30.3332	29.5941	31.0504	30.4712
30.4180	28.2904	30.0664	36.0332	31.3748	33.7599	31.1245	33.6976	35.9215	28.4220	27.1926	32.5459	30.2536
29.5134	28.9701	31.0212	36.3147	32.8063	33.4798	30.2369	33.9835	35.4149	29.3515	27.6644	31.4686	30.2339
32.1799	38.6834	36.3994	38.5352	38.0072	36.3687	35.9284	37.4671	37.5222	38.6249	35.8276	37.4719	37.2956
29.7330	27.0187	32.0002	38.5441	35.3396	33.2676	36.1712	32.9129	35.9762	34.1736	32.6635	34.9670	33.1802
34.2807	32.7519	32.0776	37.0814	36.2253	35.7169	34.6976	36.2953	35.9909	35.0990	36.3533	36.4678	34.3474
37.3892	29.7132	37.0545	40.0000	40.0000	37.3596	37.4289	37.4772	40.0000	38.4540	38.0153	40.0000	40.0000
36.7677	40.0000	37.4527	40.0000	39.1290	40.0000	36.9417	36.6135	37.7167	35.8101	37.7023	37.0282	37.0170
33.2160	37.6387	33.0368	35.5161	36.8509	33.7975	34.4691	34.9234	34.0231	34.6880	33.8896	34.9819	35.3239
24.1040	24.5774	23.0371	26.9549	24.7555	24.7588	25.4655	24.9093	27.8755	26.0463	23.4883	24.4414	25.1930
30.0437	29.9022	29.5905	35.1473	31.2836	29.4679	30.9312	30.3617	34.9100	31.1767	29.7534	30.9229	31.0348
19.1887	18.5366	18.2188	21.0685	19.1806	19.3147	20.0154	19.6206	21.7764	20.6324	18.8759	18.6509	20.0714
23.2747	23.2235	23.0531	26.6526	24.0824	23.2228	24.0007	23.7096	27.1603	24.5012	23.5686	24.5166	25.5170
36.9924	40.0000	40.0000	40.0000	40.0000	37.0728	37.9486	37.0277	40.0000	36.4993	37.9494	35.4815	33.1186

Table S3. Median Ct values of plates A and B.

30.8116	32.4448	28.9495	31.5081	30.3748	29.0175	27.7214	30.6250	31.1441	29.5471	29.1066	27.4393	30.9557
22.2384	21.4801	22.3681	23.4365	22.6787	24.3442	24.5954	25.4405	24.0889	24.9583	21.5498	25.1438	24.1469
36.0692	39.0772	39.2434	38.2231	38.1207	33.9311	36.6804	38.4971	38.8378	37.8008	38.5478	37.2862	36.9787
28.9797	27.2373	26.5334	31.4397	30.5054	25.2782	28.7181	25.7952	33.2686	26.7756	28.1592	29.3011	29.7345
30.9909	35.4762	34.9675	38.4216	37.0176	31.1512	31.9940	32.5575	38.1214	33.5512	33.9604	33.5022	35.2916
24.0886	26.4513	19.6776	27.9507	25.2463	24.5294	22.5975	25.5133	27.1280	25.0853	24.1452	23.2554	21.1831
27.4289	33.7553	28.0441	33.4627	32.6285	27.1073	27.9703	27.9744	34.2361	33.0926	32.6702	32.7387	31.0816
29.9603	31.4533	26.6236	33.9019	29.7280	26.8940	30.1356	27.6102	33.6727	29.3544	29.0493	31.3047	30.4905
20.7167	23.7721	21.9877	26.7164	23.4577	21.8102	22.1045	22.1102	26.3242	22.9546	22.0258	23.4241	25.2051
37.1154	40.0000	40.0000	40.0000	40.0000	40.0000	36.8112	39.4627	40.0000	33.9523	39.1261	40.0000	40.0000
38.3288	38.8687	36.9576	39.4557	37.2106	36.7860	39.4602	38.6379	37.2601	36.5323	36.7292	38.2208	39.1496
20.4345	18.6198	19.2318	22.4066	20.0068	21.4075	20.6610	21.6457	23.6341	20.8714	19.8970	20.5940	21.2926
32.1272	40.0000	37.2000	37.7852	37.7618	40.0000	35.4508	38.8109	40.0000	38.0201	35.2963	37.3500	38.1526
31.4722	37.5068	36.3568	40.0000	37.5517	37.7261	34.6902	37.7550	39.0635	37.3510	34.1855	37.5847	38.9779
27.2504	25.0902	24.9557	27.8855	27.2245	26.3913	25.4184	25.7237	30.0335	26.2542	24.8970	27.2189	27.0811
19.8836	18.0461	21.5805	21.8223	22.0451	22.7073	20.2824	22.4701	24.2620	21.4465	19.2603	23.3811	23.7629
20.7908	19.8389	23.2756	22.8861	23.6799	23.1647	21.3366	23.5580	24.9782	22.8214	21.2621	24.5716	24.6645
25.8254	23.4224	27.0158	27.2171	26.8365	28.0422	26.2980	27.6223	29.2976	26.7074	24.7650	28.0296	29.1823
34.6455	34.7767	33.9837	36.3286	34.0438	34.5308	31.8503	35.4505	37.6065	36.1341	35.2234	31.3238	30.9135
36.5046	36.9733	36.3900	37.3109	36.7183	39.2533	35.4520	37.6352	36.9358	35.7916	36.9831	35.6113	37.2035
39.2076	39.8237	40.0000	40.0000	39.2470	40.0000	39.7079	40.0000	40.0000	40.0000	40.0000	37.1069	36.6786
38.2051	38.0572	38.7560	40.0000	38.3949	40.0000	40.0000	38.6693	39.0656	38.3725	37.9114	38.5707	38.3012
30.9309	29.1699	28.5389	31.0456	29.7204	29.9704	31.4917	30.2429	32.9633	30.8814	29.9754	29.8629	28.1846
24.2799	23.3663	23.2830	25.4426	24.4215	24.3766	25.1618	24.7433	28.3140	25.4310	24.3771	24.1351	25.2077
36.9200	36.8698	31.8508	36.7145	36.1150	33.8090	35.5361	34.9093	40.0000	34.5493	37.0485	31.7415	38.8152
35.5684	35.9556	35.6935	36.4502	37.3586	38.0695	37.7941	36.8898	40.0000	37.7449	37.5505	38.3113	37.5230
33.0490	29.7406	35.0945	35.3642	36.2139	31.4084	37.6127	30.8953	37.8825	35.1872	34.9926	31.6210	33.4323
21.7054	21.2103	20.6373	23.7212	20.3988	21.4430	23.2647	22.2895	25.5994	23.6689	21.2022	21.6279	23.3446
28.8443	31.3483	29.6880	31.8175	33.8375	29.0404	30.5897	29.5131	33.7117	30.5705	33.1259	32.4980	31.8125
28.5849	27.2058	27.2699	30.2684	29.1560	29.2806	27.3283	29.0602	30.3982	29.1533	27.2110	27.3290	26.7915
26.6741	25.0958	27.2945	27.9478	26.3948	28.4156	26.8911	27.7131	28.7784	28.3834	25.8681	26.0304	27.6494
24.6576	24.9227	23.9699	27.2976	24.6191	26.2411	26.0632	27.2732	27.9635	26.9326	24.6953	24.8813	27.1585
40.0000	38.6189	38.2360	39.5300	35.9769	40.0000	38.9398	40.0000	39.5333	37.0345	37.1201	37.6355	39.9590
32.5825	32.4931	28.5245	31.3145	34.0986	35.4991	34.2596	35.1913	35.2290	35.0105	32.0357	33.1510	31.9681
25.6505	23.6706	26.2447	25.7134	25.8983	25.2323	26.3885	40.0000	29.4682	27.6301	40.0000	24.8879	26.7945
26.7450	25.0005	27.0640	28.4216	27.4459	25.9665	28.6252	26.7705	32.2389	27.4049	27.2973	25.4301	28.1862
30.5677	28.2300	28.0971	28.9569	31.4274	30.9829	31.9789	31.3435	32.4817	31.2814	30.1683	30.6633	31.1781
33.0317	29.7598	30.6060	34.7901	33.8374	31.3287	33.3745	31.9477	36.8501	34.0680	32.4085	30.9266	31.8938
33.6674	30.2603	30.9091	34.9655	33.8338	32.6155	34.2517	32.0567	36.4067	34.1266	32.1338	30.9922	32.5078
30.5024	29.7917	28.5666	33.0118	29.8521	28.8104	30.0972	29.2520	34.4000	31.0942	30.0978	28.7144	30.5518
34.1016	28.2270	35.2342	36.9514	37.0926	38.3176	30.8516	38.7603	38.9035	36.4646	36.5901	33.5197	37.1120
35.3849	30.4699	34.7033	30.9460	30.8546	34.4621	32.6319	31.7695	32.8084	36.3380	30.8327	32.3568	31.3757
30.7214	40.0000	36.1228	40.0000	40.0000	39.1252	37.6366	40.0000	38.4010	38.2075	40.0000	36.2355	38.0182
29.0278	27.1743	27.5657	31.7752	29.4559	26.5069	28.8355	27.4647	33.7935	29.3875	27.3643	30.0691	28.9986
38.4737	37.2498	37.3124	40.0000	37.5935	36.6758	37.5814	40.0000	40.0000	40.0000	38.5278	37.7494	40.0000
25.3816	24.5868	26.6972	27.9756	27.0922	28.6645	25.5426	27.7690	30.4078	26.3095	25.8026	29.1851	30.0735
32.9678	32.9996	32.7437	31.0438	34.5037	35.5832	33.1511	35.3368	33.8368	33.5315	34.1391	34.5214	34.9132
32.6604	29.4725	30.8462	32.6766	32.2130	31.9408	31.0488	32.1364	34.6631	31.6707	31.1478	31.3120	30.9016
38.5284	32.5597	38.0603	39.3352	37.6835	38.8198	38.8369	39.0539	40.0000	38.3301	40.0000	37.9287	39.7467
36.8698	29.1414	32.4525	36.0006	35.4978	38.5503	33.8432	36.5338	36.7116	34.1370	35.2963	36.6605	37.7352
32.3443	31.6116	34.2876	33.7058	33.9286	33.8744	32.7947	34.8744	34.4352	34.8929	34.0833	33.1045	34.3041
40.0000	40.0000	40.0000	40.0000	40.0000	40.0000	40.0000	40.0000	40.0000	40.0000	40.0000	40.0000	37.1856
21.4833	24.5051	22.1260	25.7793	21.6353	22.3032	21.0376	22.7389	25.9419	23.5843	23.0744	22.2790	25.2072
21.5082	24.0209	22.5311	24.2205	22.0813	22.4753	20.3234	22.9286	25.0904	23.8091	22.5182	22.6037	26.1093
20.0110	19.1122	18.9941	21.0814	19.5717	19.6596	19.4568	20.4327	22.6424	21.4316	19.7000	18.0541	20.6378
36.9929	30.9441	36.6971	35.8990	37.7955	38.1890	36.1314	37.2081	38.0099	33.1597	38.0618	30.1164	40.0000
34.0981	30.9149	29.8720	32.4647	32.9534	33.5207	26.5970	34.0056	33.9691	30.2258	30.9708	29.6864	32.0516
32.3791	38.9142	37.8824	38.5010	40.0000	40.0000	36.0094	39.2300	39.9371	40.0000	36.2556	38.2122	39.0940
28.5041	27.1806	26.5312	28.8018	27.0261	27.5089	26.4588	27.2665	30.5147	29.3305	28.8196	25.6161	27.3082
38.6196	38.1022	39.3919	40.0000	38.3497	40.0000	38.0788	40.0000	40.0000	38.3342	38.8834	39.2749	39.2073
40.0000	40.0000	40.0000	40.0000	40.0000	40.0000	37.6681	40.0000	40.0000	40.0000	37.3546	40.0000	40.0000
40.0000	40.0000	40.0000	40.0000	37.2253	40.0000	40.0000	40.0000	37.5525	38.4954	37.8225	38.2003	40.0000
39.7443	40.0000	37.2565	40.0000	36.8997	40.0000	40.0000	40.0000	40.0000	39.9893	40.0000	38.7550	40.0000
40.0000	38.4614	39.5799	39.9344	38.6198	40.0000	39.0657	40.0000	39.2296	38.6924	39.4727	40.0000	40.0000
40.0000	40.0000	38.7949	37.5486	39.8699	37.8991	37.2728	40.0000	39.2938	38.2008	37.9766	35.6516	38.4016
22.1464	21.7417	22.3565	23.3127	24.0443	23.5720	23.4522	23.7189	25.3459	23.5025	22.0461	24.0400	24.4081
34.5365	40.0000	37.6581	40.0000	37.2736	38.0903	35.8468	39.8560	40.0000	40.0000	36.1915	37.5341	40.0000
26.4342	26.3074	25.6959	28.4553	26.6553	27.8214	26.9931	27.5800	31.2779	28.5761	28.0823	26.5857	27.6506
39.1103	38.6000	38.4269	38.7387	38.1445	39.5174	39.2044	39.7475	35.7378	38.0339	39.1912	39.2238	39.7805
29.5256	29.8165	29.0484	31.9225	28.7298	31.3009	29.8567	32.6298	32.9543	32.0876	30.6876	29.5497	31.7767

Table S3. Median Ct values of plates A and B.

25.8728	27.0862	26.5981	28.0444	27.0845	27.4489	28.1218	27.9815	29.3383	28.3941	26.9386	26.4556	29.5465
33.5489	31.8102	31.2812	34.4455	32.3181	33.4179	33.3575	33.7653	36.0423	34.2000	31.9665	31.9579	33.4487
26.1821	25.3086	25.9256	28.3427	25.9370	28.8824	26.0568	28.3291	28.6084	28.3681	26.4931	26.0180	27.6876
25.2437	23.6145	26.1728	28.8347	30.9582	27.9745	31.2960	28.0983	28.7243	29.5248	25.8353	28.1830	28.3308
38.5426	40.0000	40.0000	40.0000	40.0000	40.0000	37.1291	39.5741	37.8583	40.0000	37.2742	40.0000	37.4255
33.4932	31.2642	30.6444	34.0422	31.4217	29.9601	30.8064	29.8752	36.4259	31.5598	29.8637	28.6903	31.7676
27.6116	26.0823	23.7862	29.0508	25.1151	25.8618	28.8872	25.8361	31.2088	27.1150	24.8794	26.1922	26.8002
29.3352	27.3681	27.2711	30.7332	28.7305	29.3789	29.1893	29.0846	32.1962	31.1113	29.2320	28.2448	29.6037
23.6899	24.1319	21.6533	26.9521	24.2974	26.3609	23.0258	26.4277	28.6870	26.5645	25.3548	24.2376	24.3913
39.3882	40.0000	38.0477	38.4346	40.0000	38.0675	40.0000	39.2937	40.0000	40.0000	38.4538	37.8401	38.7724
32.2116	38.7579	38.1550	37.4665	40.0000	40.0000	34.7224	40.0000	40.0000	40.0000	36.5772	40.0000	36.8057
32.8019	40.0000	35.0596	39.3780	36.9180	38.0205	35.7302	37.4981	40.0000	38.6441	35.6524	34.9701	37.0022
36.6019	39.3145	40.0000	40.0000	40.0000	34.1259	37.7713	40.0000	40.0000	38.3200	40.0000	37.1419	40.0000
35.3682	39.7478	40.0000	40.0000	40.0000	38.8648	36.2481	40.0000	40.0000	40.0000	39.2993	40.0000	40.0000
32.8495	36.7314	35.9484	38.2763	35.2908	32.7439	33.3825	32.6716	35.9816	32.6868	32.0606	33.1936	34.4586
37.8386	40.0000	34.9311	37.7204	36.0277	36.4776	37.0899	37.7860	38.4407	34.8968	36.2783	37.1081	38.3513
26.4909	24.9560	25.9418	27.9127	26.7348	27.0951	27.0544	26.9191	29.7147	28.0929	25.8835	27.1630	26.7946
21.4634	21.8993	21.3382	25.3333	23.1527	22.0711	22.1321	21.8017	27.5225	22.3407	21.7159	24.3976	24.6730
23.7527	29.1073	20.3820	25.6499	21.6570	21.1629	22.9437	20.5804	27.9688	21.3736	21.3462	24.2938	26.0183
24.2321	24.4988	21.2008	27.4065	22.9266	23.0376	25.6563	22.5037	29.1313	26.1872	22.2417	23.8025	26.7251
26.8894	27.3818	32.3674	33.1100	33.3621	30.2798	29.0563	30.1642	35.4363	32.5532	31.0815	32.3651	31.1572
21.8929	21.5164	21.9211	24.5906	22.5709	24.2303	22.8113	23.7774	25.3993	23.9281	22.5667	23.6397	23.2507
25.7197	23.6950	25.6326	26.0716	24.8808	26.3729	25.2470	25.8190	28.4160	25.9116	25.1842	25.2285	26.3451
40.0000	38.1448	40.0000	39.0133	38.3401	40.0000	37.8981	40.0000	40.0000	39.8788	40.0000	40.0000	40.0000

Table S3. Median Ct values of plates A and B.

24	25	26	27	28	29	30	31	32	33	34	35	36
34.9814	26.8398	30.5481	28.7539	30.5956	38.8466	25.3369	29.5882	25.6628	27.4993	37.8773	27.3017	32.8080
37.7156	30.9367	33.6624	32.6742	34.5057	38.3827	29.0506	32.6112	29.6948	30.2913	38.5359	31.4606	36.5613
35.2677	21.9427	22.6442	23.1548	23.9742	33.9561	22.3832	22.3495	24.1831	34.2011	34.3690	22.1660	23.9106
27.4491	23.3082	21.8287	24.9232	24.3692	26.7860	23.2087	22.8331	24.9672	24.8305	29.2736	23.1193	24.6721
26.1440	21.3212	20.7887	23.8538	22.8903	25.4068	23.7810	21.8235	23.4883	23.7025	25.2879	21.0681	22.3765
23.1765	19.5369	18.6773	21.1962	21.2774	22.8577	21.2689	19.4888	21.2125	20.5222	23.2300	19.1948	20.1465
29.6943	25.1162	25.2625	27.3692	26.9988	29.8037	27.9750	26.8628	27.9384	26.1200	27.2208	26.3562	26.7952
23.9423	21.4083	20.6392	23.5170	22.7595	25.1282	23.3436	23.1365	22.5181	21.6540	26.6782	21.7344	22.5290
27.0596	19.7482	19.4092	22.0810	21.8739	23.8235	21.5597	21.0753	21.8993	19.9704	27.9148	20.3054	20.5570
26.9429	20.9388	20.7169	23.9088	22.5590	27.0291	23.5901	22.5926	23.1507	20.3890	24.2254	21.5650	21.3210
23.8083	19.7568	18.6692	22.2379	20.6927	25.6217	19.8983	19.1499	22.2410	21.0655	24.7926	20.1724	22.5036
25.3024	20.7958	21.8829	23.7632	21.7827	25.6772	22.4695	22.1492	23.7748	22.1731	28.0215	21.0972	23.8389
30.0255	26.6123	25.7714	28.2095	27.1590	29.7136	27.6114	27.2171	28.4084	28.0009	28.4884	26.0654	27.7941
25.2215	22.2905	21.8858	24.2863	23.4657	25.4764	23.7469	24.0024	24.6525	23.4291	23.7068	21.3711	23.0349
25.9499	20.2914	19.5543	23.1126	21.7672	23.0222	22.6167	21.3374	23.7386	21.1708	24.4663	20.9798	22.3680
26.7371	22.6048	21.5036	25.3463	23.9125	25.1821	24.4547	22.9946	25.5267	22.8278	25.9989	22.7967	24.3835
25.3223	21.5437	22.9145	23.1681	23.3939	26.0133	23.5933	22.4315	24.5550	23.1740	32.0938	21.8107	24.9148
30.8820	25.5750	25.0010	26.9788	26.8721	30.3567	26.9799	26.3071	27.7774	27.5132	30.1389	25.8485	26.8670
29.7958	26.6809	26.0447	28.5759	27.8673	31.3662	26.7732	26.3925	28.5795	27.4870	29.5720	27.0330	28.5874
26.0669	21.9553	20.7157	23.5605	24.5879	25.7221	24.0354	22.7564	23.5606	22.0256	25.9330	24.2942	24.5595
30.3146	23.8727	22.5578	26.2105	25.9467	27.7693	25.5772	23.8732	24.9962	23.6219	26.7853	25.5029	25.6839
25.3819	20.9017	20.1938	23.2287	22.8999	24.9974	22.7032	21.3131	22.7289	21.5617	25.9759	21.7795	22.5575
29.2076	26.4556	25.4933	28.8135	27.4195	31.0036	26.8324	26.9589	27.8288	27.3812	33.0820	26.4654	27.4920
23.5277	20.8427	19.5747	22.3340	22.4348	24.7372	22.5925	21.7370	22.4784	21.9037	24.1588	20.6180	21.8182
26.4028	21.7217	20.9797	23.8209	22.5663	25.3342	23.4648	22.7523	23.4552	22.7580	24.2456	22.2173	22.8638
27.0536	23.7638	21.8687	25.0342	24.4046	26.6777	25.3848	24.2561	24.8027	24.9816	27.4182	22.7347	24.1062
27.4751	23.2798	22.6966	24.9756	25.2486	27.4239	25.5456	24.3682	26.0782	24.2591	31.7867	23.8075	25.0977
40.0000	36.6043	34.4016	40.0000	40.0000	37.7701	39.1558	39.0157	39.8538	30.7553	39.8736	36.9389	40.0000
29.8102	25.1098	23.3086	25.9859	25.7074	28.1139	25.2796	25.5293	25.3199	28.6016	31.8779	25.4333	24.0202
37.9862	30.5964	34.4471	40.0000	33.9038	38.2785	35.9581	36.8952	36.7394	36.1096	40.0000	38.1523	37.1226
40.0000	30.6658	34.5487	40.0000	33.6695	39.8116	37.2243	36.9733	36.2400	38.4744	40.0000	40.0000	40.0000
25.0544	19.8240	18.7432	21.7852	20.7811	25.5837	21.2352	20.0925	20.3636	19.0579	22.2368	20.9390	19.3108
38.3419	35.1952	32.7751	35.7614	34.5646	36.4313	33.9799	32.8151	35.8908	32.5860	35.3451	35.2313	35.3790
40.0000	37.3687	34.7662	40.0000	36.5452	38.9930	37.1982	36.5433	37.3463	36.0637	40.0000	38.9889	37.2077
30.1602	27.2290	23.9649	29.5504	26.7561	30.3676	27.1072	27.0326	27.9269	28.3147	32.6972	27.1871	28.1666
27.9225	30.9834	24.0148	29.1477	25.9888	24.2314	25.7348	28.4995	28.4553	27.7780	34.2307	25.9139	25.3420
28.3344	27.9150	23.5292	28.4554	26.1857	23.5755	25.5738	28.1373	28.8121	28.4556	28.2513	25.1607	25.9481
25.9312	23.3357	21.1872	25.6687	23.2376	25.8213	23.9857	22.6124	23.9243	25.4338	24.6844	22.7250	24.9086
40.0000	35.4161	32.6482	37.5697	34.9857	39.0058	35.9736	35.2563	40.0000	35.7459	39.3782	35.4118	37.3390
40.0000	36.9717	37.7272	38.8653	38.8787	40.0000	38.3968	38.0146	37.0582	35.5884	40.0000	37.7998	37.8473
25.7997	22.2765	21.2246	24.5784	23.3100	27.4506	22.8602	23.3706	23.4987	22.8456	32.2623	22.3133	22.3723
31.7623	29.4733	26.6116	32.2745	29.8943	33.1563	29.0507	29.3942	30.5981	31.8409	37.3152	28.4468	30.2368
40.0000	37.1299	40.0000	40.0000	38.2495	40.0000	38.1488	39.3428	40.0000	40.0000	38.0323	39.9154	38.4211
38.2116	33.5857	33.1443	35.7369	35.9262	36.8618	31.7442	35.0956	33.4506	34.3553	40.0000	33.9536	35.4309
31.8072	29.8866	28.7284	32.2827	31.0375	28.2734	28.0990	27.4786	28.5281	29.4734	37.4396	29.7559	29.6944
27.6157	23.4871	22.9545	27.0641	25.1957	24.8888	27.5927	29.1326	26.2682	24.1772	27.9803	25.1806	26.0380
36.1600	31.8744	23.7651	37.4938	27.1128	34.3562	26.6011	28.7303	38.7498	37.0608	37.7956	36.6351	25.6460
26.5195	23.1026	23.2257	29.7391	25.6998	29.0764	27.6604	27.1525	28.5528	26.4435	21.7133	28.9373	26.4126
40.0000	37.7324	35.6049	40.0000	37.4363	34.1864	36.6364	35.1963	40.0000	40.0000	40.0000	40.0000	36.4396
34.1138	29.2835	27.2203	32.2572	29.4936	32.7745	30.0432	30.3983	31.4631	31.5405	32.5579	29.2673	30.5585
34.6134	30.8130	27.1965	33.6961	31.6102	33.0759	31.5584	32.5598	32.4616	33.1149	32.3218	29.8466	32.2652
39.2393	34.6977	33.4244	37.5298	35.8243	34.4271	35.5163	34.6276	36.3958	34.7615	36.6639	35.9528	35.3825
25.8123	21.8105	22.6455	28.0969	24.9870	25.7207	28.0177	26.1655	26.6921	24.7652	27.6306	26.9413	27.5431
27.1867	24.2624	24.8092	27.0242	25.4832	26.4068	26.4769	25.0454	28.3111	26.2970	28.6546	25.1905	24.9674
31.7752	29.8393	28.1035	31.9947	30.1685	31.8270	30.3353	29.0953	30.4879	29.7207	29.7289	28.7864	30.4949
31.5276	25.3331	27.0185	28.8130	27.8773	35.7762	34.2634	30.0140	27.9654	31.3734	34.9757	25.5066	27.1892
32.8473	26.0583	27.1245	29.3580	28.2112	34.9435	33.3932	30.1854	28.3288	30.9269	37.0639	26.4293	27.8212
39.0602	36.6836	35.7512	38.1093	38.7915	30.4918	37.5580	37.1899	38.0066	36.1215	37.8726	37.1514	37.0628
36.2206	31.0084	29.5222	30.3698	34.0680	34.2977	30.5571	34.2690	32.6812	34.3387	37.1181	24.6867	30.2204
37.8899	34.0604	32.5203	35.2442	35.4685	38.0209	32.5569	36.3591	37.1526	34.4919	36.9631	29.4398	34.9258
40.0000	38.4052	37.2337	38.1867	39.4901	40.0000	35.4891	38.4996	40.0000	37.9184	37.5560	40.0000	39.0883
40.0000	38.1156	37.8537	39.5957	38.9390	37.4299	36.3700	36.2893	37.1758	37.1388	40.0000	37.2473	37.9417
34.0435	33.9405	31.7899	33.8053	35.5358	34.7145	32.0274	33.5606	34.1282	33.2881	37.8536	34.3563	33.2303
26.6522	24.1620	23.4128	25.4671	24.9908	27.2811	25.0018	24.1767	25.5302	24.6444	27.2595	23.9039	25.2385
33.3803	30.8930	30.3310	31.6468	30.9785	33.6770	30.2421	29.5201	31.1825	31.2457	34.2249	31.0181	31.2551
20.9113	18.3409	17.6830	19.8212	19.4357	19.7700	19.9623	19.0738	19.9652	18.9327	23.9546	18.7670	20.0309
26.6564	22.6223	21.9129	24.5729	23.8238	25.8725	23.9781	22.6689	25.2062	24.0984	31.2656	23.2078	24.3462
40.0000	38.3076	34.9435	37.8489	36.6333	37.0074	37.8528	40.0000	38.4332	37.0298	40.0000	39.0670	40.0000

Table S3. Median Ct values of plates A and B.

30.9782	25.8589	26.3442	33.2385	30.2435	27.8722	27.1217	29.1299	29.5225	29.2170	32.2648	30.9527	29.2267
24.5984	21.7545	20.5994	24.2385	22.0082	22.6563	22.2641	23.7690	23.8023	21.6505	21.8605	20.9099	22.4840
40.0000	39.0116	35.7973	37.0138	36.5788	37.2865	36.1906	36.8789	36.7850	38.4700	34.3179	38.2155	37.1731
33.6046	28.5915	26.3540	27.4239	31.2490	26.0648	24.0353	25.8991	28.0272	28.6677	32.0315	25.7309	26.7721
35.1064	31.5691	33.5836	35.5191	35.3145	36.3574	32.5412	33.4882	35.3640	33.9907	37.5313	34.4257	35.3416
27.6551	23.6034	23.4784	26.9185	24.6867	26.0415	25.5469	23.8913	25.8361	21.2248	25.1516	24.5020	26.1876
32.3439	32.7707	29.6095	35.3052	34.5340	34.3687	30.9518	31.3234	34.8862	30.8302	32.5036	34.4815	33.6316
33.5033	26.9289	26.9507	30.2351	30.7937	31.4089	28.4242	27.2170	29.2569	30.6288	31.0423	27.6567	28.6703
25.3042	21.2390	20.5845	24.0039	22.2135	24.4757	21.5214	21.9382	23.8398	22.1022	29.7328	22.3554	23.2091
40.0000	39.0490	38.7508	38.7345	40.0000	39.8506	39.0253	40.0000	40.0000	39.8821	40.0000	40.0000	39.9865
36.3739	37.0248	36.0641	38.7268	37.9068	38.1284	39.3480	36.7079	38.4719	35.1261	37.1417	34.9892	35.6829
21.9190	19.1109	18.6484	21.8928	21.0272	22.2010	21.3685	19.6488	21.3956	20.2818	22.6445	19.0175	19.8654
40.0000	38.7464	36.2453	40.0000	40.0000	28.3046	37.5903	36.3819	40.0000	37.4468	40.0000	40.0000	39.9352
37.9467	40.0000	35.6858	40.0000	40.0000	28.3280	39.1619	36.9700	40.0000	36.4056	40.0000	40.0000	40.0000
28.2035	25.2727	23.1752	26.9512	25.0545	30.8778	25.1866	26.0102	25.3663	26.7199	25.2726	23.4347	24.9816
22.1201	18.9515	18.3524	20.3454	20.8141	19.9938	21.2970	21.8334	21.8664	22.3722	25.1993	18.9310	20.3117
23.2119	21.6195	19.4019	22.5036	21.6671	21.9140	23.2217	23.0620	24.9716	24.5497	20.8853	19.9460	21.9799
27.4992	24.3854	23.2118	26.9805	26.6626	25.3523	26.2630	27.2488	27.8271	27.3017	29.1888	23.9476	25.6058
36.0712	34.1991	31.5029	36.6090	33.5438	36.8556	34.7977	35.9938	36.1232	34.2017	35.5251	35.0930	36.5251
37.5976	36.9254	35.9789	38.5560	38.3352	34.7613	38.2155	36.1107	36.3102	34.1769	38.2321	35.4022	37.8540
40.0000	40.0000	38.8253	40.0000	40.0000	40.0000	40.0000	39.3437	40.0000	40.0000	40.0000	40.0000	40.0000
37.1699	33.8470	37.1254	39.4542	36.0288	37.5079	38.2061	37.3450	36.4796	37.0142	40.0000	36.3218	35.1533
31.5294	28.9531	28.1076	31.4108	30.2433	31.7827	29.9173	29.6011	30.7307	30.0604	32.6732	29.4541	30.3637
25.9135	22.6948	21.6955	25.6946	23.9746	25.8833	24.4124	24.0772	25.1745	25.0138	26.5448	23.0921	24.1230
36.0487	35.6859	35.8227	39.4810	36.2354	39.0618	32.5240	32.3057	36.7838	35.2378	35.3107	36.9001	39.4725
38.6535	35.4649	36.7604	37.4620	38.4833	37.0088	37.6169	37.2426	36.7001	37.7167	36.7426	36.8573	37.7721
37.3857	30.9352	32.7351	35.8014	35.2134	35.6992	33.2506	35.0889	37.2782	33.8912	36.5614	33.2182	34.9837
23.7535	21.1806	20.2159	25.6840	22.0349	22.9725	22.6422	22.0145	23.0908	21.4976	21.7316	20.9186	22.9158
31.1427	32.2617	30.4884	33.0187	32.7345	28.8919	29.5343	29.8103	32.6554	30.6220	33.9910	32.5738	32.5470
31.1797	27.9147	27.2869	29.6116	28.3560	30.9576	28.7477	29.4070	28.1986	27.8702	29.8160	27.4749	27.5228
28.0280	24.9385	24.4302	29.0481	26.8073	26.8503	27.0375	25.6025	28.7441	26.0546	26.9392	24.9259	26.3004
27.0466	24.6248	23.7334	27.8080	25.4579	27.5369	26.1625	25.4971	26.2321	26.0202	25.7276	25.0882	25.8526
40.0000	38.1972	39.1812	40.0000	40.0000	39.2143	37.2575	39.3082	38.3520	36.0941	40.0000	37.9475	37.7951
35.1715	31.9924	27.6924	33.9769	33.9118	33.5257	30.1700	31.3185	34.8997	31.8501	34.1585	32.0961	31.4415
25.5068	24.1063	23.1472	27.5672	25.6021	25.4280	25.9259	26.0140	26.6243	25.7148	25.0866	23.0662	25.5531
26.5150	25.0809	25.0611	27.9925	26.7649	27.0920	27.6332	27.7618	26.0206	27.2921	30.6625	24.0525	27.2423
30.4427	29.7037	25.8969	32.5421	31.3882	30.8999	28.2950	30.4816	31.6977	30.2424	32.3868	29.0048	29.6964
33.8077	34.7089	30.8126	33.5015	33.1334	34.2868	32.2638	31.2977	31.4336	31.3560	33.9587	32.8069	35.4691
35.4548	34.7345	31.8393	34.2511	33.7324	34.3260	32.2445	31.7362	32.0762	32.4917	34.3024	34.4563	34.9917
31.8481	30.1741	29.8249	31.3925	30.9706	32.0238	29.5615	29.7809	30.2584	30.0509	30.9259	30.3878	30.5852
35.0698	29.9373	34.7025	35.1674	38.6875	34.1967	34.0401	36.1061	38.4901	36.3899	40.0000	35.3148	34.1512
30.2639	32.3023	32.1913	38.0995	33.9383	31.8335	32.0861	31.2585	33.6176	30.6217	32.9588	30.7838	32.5431
38.6517	33.9920	37.2924	40.0000	38.6693	38.6004	37.8595	38.6542	40.0000	35.1153	40.0000	38.6611	37.3225
30.5618	30.5083	24.8556	28.5974	28.4492	30.2389	28.2341	28.8105	29.0376	30.4290	33.4304	26.5333	28.7622
37.9895	36.1264	36.3123	35.9738	40.0000	39.7099	37.0347	38.4226	24.2284	35.4548	40.0000	38.5510	36.3713
27.6943	25.3944	24.2848	27.1255	26.8841	26.3370	26.6308	26.9191	25.7574	28.1705	28.0092	23.9907	25.4688
35.8800	32.6750	27.6886	36.9240	34.1133	33.3890	30.3325	32.5193	33.7983	33.2334	30.6430	33.2156	31.2377
34.0123	31.6506	31.5382	32.4482	32.3533	34.1260	32.3258	32.3810	31.8510	30.5879	36.2329	31.0822	31.2455
38.6683	39.6753	38.6891	40.0000	40.0000	36.5350	38.2566	39.1492	38.2161	38.3358	40.0000	37.9311	38.3110
39.2686	36.6416	30.0866	37.2950	36.0342	34.5381	33.0038	37.1639	37.4639	35.5674	35.7675	36.8603	34.6187
33.9920	31.2814	31.0372	35.9400	34.0085	33.9509	35.0174	34.1370	36.9751	32.8228	35.7752	32.3481	33.7893
40.0000	40.0000	40.0000	40.0000	40.0000	40.0000	40.0000	40.0000	40.0000	40.0000	40.0000	40.0000	40.0000
23.2622	21.1495	21.2185	25.5668	22.1710	23.2463	23.8803	22.4030	24.9983	23.6079	27.8107	22.9619	23.9175
22.8988	21.5485	21.0821	30.2403	22.7858	23.3666	24.1624	22.9630	25.0975	24.6016	24.9895	23.0076	23.2337
20.4384	18.9892	18.1650	23.1184	20.0425	22.4763	19.9820	18.5496	21.6447	20.7746	19.7042	19.6487	20.5503
34.1296	38.3157	32.9766	32.8102	39.7547	27.9917	29.1213	36.4098	36.6873	36.9177	36.2104	32.3875	36.0325
34.2326	30.2306	29.3306	34.9750	33.6541	33.1732	31.4472	31.4791	34.1645	28.9899	32.7917	31.3535	31.7160
40.0000	40.0000	37.3245	40.0000	40.0000	29.2299	39.5824	38.7696	40.0000	38.2075	40.0000	38.7780	39.7087
28.7145	26.5919	24.6153	29.3921	26.9081	29.5084	27.6037	26.6957	28.6129	30.1534	35.1987	26.5091	27.6937
34.6416	38.3102	39.0669	39.8766	40.0000	40.0000	39.5522	38.6887	37.8094	39.2090	37.1973	39.2681	39.6507
40.0000	39.6827	39.1846	38.3010	39.3987	40.0000	40.0000	39.2143	40.0000	38.5948	40.0000	40.0000	40.0000
40.0000	37.7239	40.0000	38.4932	40.0000	37.6821	39.5420	38.8580	38.5070	39.1756	40.0000	39.0369	38.3015
40.0000	39.6323	39.0181	38.2168	36.2466	40.0000	40.0000	40.0000	40.0000	40.0000	40.0000	40.0000	40.0000
39.5192	40.0000	38.8221	40.0000	40.0000	38.5025	38.1306	40.0000	40.0000	39.1662	40.0000	40.0000	40.0000
38.4967	39.5190	40.0000	40.0000	40.0000	40.0000	40.0000	37.8701	40.0000	38.4203	40.0000	40.0000	39.2525
24.1350	20.8584	20.8001	25.6851	23.1145	25.1331	23.4783	23.2131	24.1830	22.6222	24.9957	22.8472	23.5223
38.2385	37.8279	37.4336	39.6314	39.5577	30.1201	40.0000	37.6794	38.3456	37.2234	40.0000	40.0000	40.0000
28.4939	26.2230	24.7698	29.9447	27.4198	28.4323	27.4663	27.3042	27.4247	28.5870	27.8683	26.1638	28.0317
38.3359	37.6789	37.2468	40.0000	39.0401	39.3179	38.0284	39.1511	38.8770	34.9410	40.0000	39.8738	39.1359
30.7455	29.9303	28.9558	36.0202	29.5518	32.8749	29.9947	30.5584	30.4805	32.1076	34.8717	29.9032	31.9914

Table S3. Median Ct values of plates A and B.

28.8326	26.7503	24.9676	29.0438	26.8157	28.2543	27.9072	26.9510	28.8012	27.3364	27.2297	27.1295	28.4740
34.3681	32.2829	31.1155	35.7392	33.1433	33.9957	33.7141	32.8532	34.8747	32.3734	33.6021	32.2466	32.7337
27.4655	26.5360	24.6024	29.1022	26.6098	29.0874	27.5512	27.0997	29.1885	28.2982	28.9607	25.3647	27.6152
26.0545	30.9085	25.4244	30.8200	28.2459	26.0587	26.7422	30.2254	29.2558	24.6527	29.2736	28.4339	27.1648
40.0000	40.0000	39.2564	38.8207	40.0000	34.2464	40.0000	39.9252	40.0000	39.7038	40.0000	37.7227	39.1200
32.9674	28.8944	31.6882	36.5160	33.5362	32.3043	31.7885	29.2088	33.1775	29.9201	38.1049	32.3540	32.1181
28.9789	24.6053	25.0273	27.5003	27.4556	29.0892	26.7525	24.8884	26.8089	26.9249	28.9817	25.4677	25.5062
29.6129	28.8754	26.4623	27.7280	30.4136	30.3158	29.2278	28.4430	29.3566	30.3342	30.0239	27.6964	29.5295
26.3044	24.9586	22.5775	27.4226	24.9767	25.9944	25.2927	25.7864	25.7870	23.9965	27.4079	24.4621	26.9568
40.0000	38.1792	37.8958	38.6286	40.0000	40.0000	40.0000	39.2548	40.0000	38.8732	40.0000	37.6075	40.0000
37.1452	40.0000	37.1264	38.6732	40.0000	27.9257	37.9482	37.4886	40.0000	37.3622	40.0000	40.0000	40.0000
38.5698	38.0555	35.9870	37.6811	39.6084	30.0311	36.2091	36.1415	38.8071	33.9227	40.0000	36.8915	36.3745
40.0000	36.5883	37.6403	38.8786	38.3799	39.2434	40.0000	40.0000	39.6602	33.2061	40.0000	33.1237	40.0000
40.0000	37.6872	38.3989	39.5684	39.4759	39.5797	39.7348	38.3369	40.0000	38.8084	40.0000	36.3058	39.9558
34.8197	30.7178	33.1056	34.7865	34.9438	33.1634	31.9898	31.8891	36.1574	30.0574	36.7722	31.5112	31.5501
36.6652	35.4969	35.0509	38.8211	36.3888	37.2568	35.2464	35.2420	36.6467	34.0835	40.0000	35.4301	35.5614
28.0524	25.0450	24.3018	29.9403	27.2699	28.8930	26.3470	25.4393	27.6947	26.7107	28.2866	25.6259	26.8675
24.0830	20.5296	21.0377	26.2440	22.9601	24.0686	21.6095	21.9990	23.8398	21.1370	30.2044	22.3461	23.0895
24.4770	19.8911	21.2372	23.2312	22.7190	25.2074	21.0186	20.5329	22.2497	20.9526	27.1733	22.1698	22.1928
26.9864	23.8891	20.9465	27.3510	22.3417	26.8377	26.1869	22.3009	23.1991	25.1830	27.6836	21.9041	23.0567
31.5381	28.7302	28.1599	33.1949	29.8611	29.8973	31.4951	31.7973	31.8548	29.9804	35.3701	30.8691	30.8565
25.0553	21.8152	20.7265	24.9186	23.5304	24.7555	23.3337	23.0694	23.7948	22.4240	26.3713	22.2564	23.2040
26.8365	25.0359	23.8100	27.9378	25.6738	27.0770	25.6843	24.9296	27.2524	26.2420	27.8527	25.6075	25.4143
40.0000	38.4969	37.7489	40.0000	40.0000	38.8247	38.1102	40.0000	37.2758	39.0883	40.0000	37.8016	38.0274

Table S3. Median Ct values of plates A and B.

37	38	39	40	41	42	43	44	45	46	47	48	49
35.9695	35.3604	37.1932	33.2006	29.5799	28.9642	34.2386	32.1106	31.2870	29.0540	29.9959	32.6950	29.7254
38.8628	39.1574	38.8447	36.1895	32.5118	32.9482	37.5157	36.4581	36.2309	32.4070	33.3228	38.0639	34.2094
28.1170	34.6560	37.5957	22.2438	24.0500	34.3940	26.9814	27.8960	25.8423	22.8294	23.8167	25.8478	23.8182
26.5225	22.8670	25.0215	23.2076	24.9197	25.1609	26.0858	24.1811	26.3398	24.5215	25.3335	26.2088	25.0142
25.0254	24.1162	24.3566	21.2811	23.9924	24.5370	23.7302	22.8402	23.2947	21.5606	24.0020	25.4514	22.5833
21.5278	21.5801	22.1037	19.7350	20.6284	21.8301	21.4228	20.2499	20.3871	20.3744	21.4361	22.3838	20.6247
27.9987	26.6715	27.9806	26.5359	27.9750	28.9098	27.2058	26.7728	26.6097	26.8521	28.2937	28.6572	26.7274
22.9484	24.3838	23.4308	21.6189	23.1229	23.7309	22.4803	21.7835	21.9423	21.7009	23.5841	23.3808	21.9393
28.2836	25.8874	23.5341	20.9443	23.1043	21.8393	22.5231	21.1965	22.7803	20.5285	22.0763	25.4129	21.0702
28.0673	25.2215	23.4440	21.3738	24.4269	24.5514	23.9077	23.4784	25.4154	21.8437	24.0461	26.7609	22.9805
25.3462	20.6603	22.4033	22.6338	21.3658	20.0704	22.9652	22.4427	25.0315	23.9906	20.3922	23.7087	21.8749
26.9607	24.5892	22.1993	22.1312	23.5239	23.3871	24.2974	24.5533	27.2398	22.8332	22.9105	24.9683	24.0845
28.6053	28.6583	28.2244	27.1117	27.6605	28.1129	28.8556	28.4971	28.9847	27.0748	28.5889	29.3027	28.8701
24.8403	23.5964	24.2345	22.6562	24.0562	24.6905	25.1011	22.7702	23.6755	23.7006	24.3099	25.5934	24.2105
24.4375	24.9397	22.7362	21.3273	21.8995	22.6993	23.4527	21.8684	22.2097	22.8821	23.7288	24.9583	22.9699
25.8036	25.1825	24.1528	23.0667	23.1845	25.0949	24.3529	22.2732	23.4057	24.3317	24.3266	27.1883	24.6198
26.2692	26.3493	24.5907	23.8809	24.1713	24.4797	24.3147	23.7548	25.4112	23.6319	24.2758	24.0753	24.2461
28.9662	28.5967	26.7524	25.6020	28.4438	27.2667	28.9450	25.6873	30.1524	26.9063	28.3385	30.9706	28.5932
31.6250	27.4179	28.9206	26.2716	28.8040	28.2329	31.6991	27.6510	29.6992	28.0396	27.8339	30.2846	28.4749
25.7196	22.5368	24.7030	22.1709	23.5119	22.7329	27.3411	23.9054	26.3321	22.3671	23.4999	25.0373	23.9699
32.6109	25.8063	25.7368	23.1226	24.5472	25.3685	28.0384	25.7397	27.2546	23.8764	24.6167	30.7365	25.7710
25.0920	22.8225	23.1495	20.4876	22.8438	22.4180	24.4571	23.9125	25.0252	21.4376	22.8601	24.4749	22.8009
29.6512	30.2250	28.8812	28.2999	28.3667	28.6672	29.1056	29.9662	29.9246	27.6595	28.2843	29.7461	28.2868
22.9934	21.7365	22.6987	21.5070	22.1740	23.3320	22.5704	22.3254	23.1447	22.9151	23.5786	22.5467	22.9688
26.6680	24.6149	23.4637	22.9905	23.1084	24.0055	24.1586	23.3003	23.9914	23.2741	24.1976	25.8499	23.6600
25.9191	24.9013	24.7760	24.3878	24.8546	26.0716	24.4459	23.6689	24.5274	25.2259	25.6482	25.8390	25.1178
26.4098	24.3456	26.1474	24.7791	25.5667	25.6905	26.0368	24.6683	25.6150	25.8907	26.4607	26.5236	26.4202
39.2630	30.1517	38.2030	33.2804	31.3499	40.0000	40.0000	31.6037	32.6839	38.4929	35.0410	40.0000	38.6725
32.6360	28.8100	28.2006	24.5729	28.1957	26.5196	28.1644	29.3641	29.3509	24.0655	26.5677	29.9198	27.9062
40.0000	35.8343	33.9516	38.0050	40.0000	37.1507	35.4557	40.0000	40.0000	38.2203	35.7591	37.4237	40.0000
40.0000	38.8215	34.1853	36.5336	39.1508	38.9110	35.7438	40.0000	40.0000	37.0703	39.0332	40.0000	40.0000
25.5627	23.0176	22.1060	19.7191	22.2455	22.5815	21.7226	23.1950	23.5298	20.5286	23.0589	25.2642	22.6847
36.1102	33.7556	36.0592	33.8398	32.7249	36.7161	38.2883	34.5394	34.9417	35.4368	35.1241	38.0492	37.1510
35.5491	36.2781	40.0000	35.9693	36.0490	38.1019	40.0000	40.0000	38.6653	35.7427	37.8958	38.3898	38.2886
29.9580	28.3851	27.8126	26.1570	28.4226	27.4421	27.3799	27.6323	27.7934	27.2987	28.3953	30.5470	28.8238
26.9804	30.1713	30.6427	26.0403	29.5978	24.5220	27.0900	26.0163	28.0806	26.5296	31.0848	27.5505	27.3091
26.9303	28.6448	31.0522	25.7190	28.1100	24.3123	27.2827	25.8665	28.1551	25.5987	29.3773	27.8311	26.8225
24.6467	23.2302	23.0957	23.3052	22.6318	24.2866	24.7066	22.1124	22.8733	23.9923	24.1213	25.5776	24.5416
38.4481	40.0000	37.2378	38.5677	36.1724	35.8903	35.9096	37.0403	37.9331	37.4995	37.1155	39.9525	38.5379
40.0000	40.0000	38.4319	38.6611	37.0749	37.0646	38.2409	37.9538	38.1412	38.5349	37.2407	40.0000	37.4572
25.1911	26.0769	23.8922	22.6422	23.9761	24.3210	23.2716	22.2700	23.9271	22.5862	24.2731	25.6822	23.6213
32.3434	33.1065	30.9942	29.3644	30.6691	30.5523	30.9099	28.4989	30.0650	28.9002	30.8691	33.8264	30.8633
39.5786	40.0000	40.0000	40.0000	40.0000	40.0000	37.5707	40.0000	37.6145	40.0000	40.0000	40.0000	38.7734
39.4543	37.7045	36.3141	33.9968	36.3324	34.8488	36.6041	33.4098	35.9438	35.0003	32.2450	38.3524	34.7694
31.2324	34.8279	32.4807	29.8528	29.9761	29.7361	33.2510	28.7619	28.2010	30.0630	31.3606	33.5363	29.5723
26.2156	27.9822	31.8567	25.2363	28.9525	27.1364	24.9614	24.6277	27.4516	30.6615	28.3660	28.5796	30.7600
35.0945	38.5680	31.9927	25.2034	38.3332	25.7464	27.4355	34.2234	36.5217	26.9193	40.0000	38.9770	37.1871
26.7958	23.9734	23.2596	26.1284	27.3382	29.2619	26.7829	20.9624	26.9961	26.0458	28.8181	28.4971	26.9038
39.1863	40.0000	38.5846	32.6910	40.0000	35.6198	40.0000	40.0000	40.0000	38.3977	40.0000	40.0000	40.0000
35.3706	30.4497	27.6453	30.1401	32.0912	29.4178	30.9738	32.7232	33.9961	30.2040	31.5008	33.9696	31.7620
37.5153	32.4424	29.2322	32.5545	33.6773	29.8578	31.9559	33.8075	35.4222	31.5472	32.4880	35.7874	32.7599
39.3153	39.4361	35.0637	36.2937	36.0637	35.4159	38.4176	35.5250	37.9240	35.1738	36.7471	38.1436	37.9096
25.3684	23.9582	25.8877	25.3732	27.5684	25.1757	23.7686	24.0821	27.3672	24.0225	28.9387	25.8616	25.4890
26.2672	26.5341	25.8830	24.9728	26.3836	25.8366	26.2805	25.8731	25.5806	25.2714	26.8597	27.3261	25.7017
31.1875	28.9925	27.8937	30.1317	29.2016	30.3472	32.5791	28.8932	29.7622	29.7037	29.5503	32.2305	29.9063
33.2423	33.0798	37.8732	26.4131	31.7943	34.2896	27.9103	30.4769	33.1264	28.3557	35.3879	29.0964	28.6248
33.7061	32.8137	38.8584	26.5272	32.2833	33.3446	27.5508	29.4781	32.3165	28.8266	33.7694	30.5494	28.4422
38.1836	38.5444	39.2221	33.4730	36.2448	39.1716	37.2279	35.2843	36.2211	36.9322	38.0423	38.4101	37.0886
36.1191	32.3177	35.7240	26.5813	34.2109	36.9605	32.9811	31.9127	35.9028	31.3542	36.7444	36.1491	31.1853
37.5210	32.9380	36.5600	30.7451	33.9005	37.7183	37.8299	34.9534	34.3535	35.5121	37.6562	38.8158	33.9961
40.0000	40.0000	38.9416	40.0000	40.0000	38.0786	40.0000	37.7636	39.9392	39.0052	36.5614	39.1492	40.0000
40.0000	39.0363	40.0000	36.7777	37.7051	37.9628	39.4684	36.3808	38.0657	38.5112	36.2006	39.0533	37.3171
34.4776	34.4347	31.0513	35.5435	33.9564	35.2417	35.2135	32.0204	33.3525	34.3702	35.2482	37.3721	34.3132
25.9626	25.0993	24.8486	24.9949	24.7732	25.6473	26.0792	24.5382	25.2815	25.6576	25.0062	26.1374	25.4859
32.1658	29.5723	31.3262	31.0604	30.9674	31.1594	32.2579	30.7007	31.7268	31.9840	31.8575	32.4423	31.8634
20.4506	18.8717	20.8307	20.1002	20.0706	19.8455	20.0595	18.7647	19.3785	20.2000	20.1362	20.4704	19.6763
25.7646	24.7229	25.3227	23.7038	24.5205	23.9951	24.2964	23.4611	24.7577	24.2208	25.2695	25.3899	25.2879
40.0000	40.0000	40.0000	39.8607	40.0000	40.0000	34.7164	37.7199	37.3749	40.0000	40.0000	40.0000	37.7087

Table S3. Median Ct values of plates A and B.

29.9526	32.5622	28.7636	29.3843	31.0812	27.9331	33.2030	28.3630	29.5119	26.3287	28.2682	32.6104	29.1085
23.6760	21.6815	22.4723	21.9075	24.1985	23.0677	24.1099	22.0331	24.7474	24.3052	25.6025	25.4527	23.6184
37.8536	40.0000	38.2836	36.1387	38.3401	40.0000	40.0000	34.4316	38.5195	39.0017	37.3394	36.7662	37.4508
31.7955	27.9332	32.2591	26.0936	29.9950	27.1097	33.2667	31.8974	29.5248	29.7730	26.1664	31.5954	27.8666
35.9770	36.6576	40.0000	33.2840	37.0728	32.6516	36.3176	33.9703	33.7728	33.8356	33.8024	38.9177	35.4609
26.0454	22.9198	25.7077	23.0079	22.0463	25.3816	26.4006	22.8806	24.9683	24.8899	23.2647	27.7755	27.3317
27.9068	29.5550	32.9806	33.3213	31.3648	35.8338	35.9649	31.3648	29.4178	34.1258	30.8248	34.0038	32.6154
32.1764	31.5447	33.8676	30.6541	27.8229	30.6624	31.0076	29.7725	29.5039	30.3374	27.0627	30.9487	28.4163
24.2718	24.1744	23.7148	20.0460	22.7317	23.6159	22.7996	21.9105	20.7357	21.8315	23.5043	25.6380	22.5755
40.0000	40.0000	40.0000	38.2818	40.0000	39.2870	40.0000	38.8749	40.0000	39.7071	40.0000	40.0000	38.9470
37.8349	37.1410	39.2437	36.2295	38.0302	39.1370	36.6399	33.8038	37.0609	37.1208	38.4722	36.0661	37.9321
20.2529	20.4903	21.6640	19.4118	20.1983	21.4251	21.1067	19.6737	19.9549	20.1950	21.5474	21.3699	20.4770
38.5625	38.4977	38.9154	35.0554	37.5483	35.0259	40.0000	38.2254	40.0000	40.0000	37.2212	38.5052	37.1373
36.9661	40.0000	40.0000	34.3826	36.9358	37.1260	40.0000	37.9834	37.6820	37.6814	36.7066	38.2271	36.8774
25.9612	24.3314	26.4209	23.6631	26.2495	26.1189	26.8300	25.9204	25.5925	23.7658	26.5501	26.3023	25.2260
22.2463	19.8864	21.2524	20.5526	22.9723	19.8492	21.3391	20.4883	22.8158	21.6184	24.4613	21.0312	21.9241
23.4075	20.5405	22.2123	21.0400	24.6936	21.5458	22.6101	21.4132	23.6050	22.4261	25.5743	22.9474	23.3167
27.0051	25.6276	27.2399	24.6069	28.2078	25.9938	26.5955	25.4498	27.3125	26.6404	29.9048	25.9878	26.7438
31.3505	34.4658	38.1775	33.4377	33.1117	37.7359	37.7241	36.2472	33.8296	33.7105	35.3550	34.3056	33.8428
34.4237	38.7651	39.0132	36.1841	37.1910	37.8674	38.9068	35.1043	34.6686	35.7867	38.3390	37.2326	35.1850
37.1733	40.0000	40.0000	38.7658	40.0000	40.0000	40.0000	40.0000	39.5416	40.0000	40.0000	40.0000	39.9282
37.2108	39.6356	39.1112	37.6368	38.4348	35.8824	40.0000	36.9883	38.4320	38.2000	38.0706	36.7425	38.3029
30.3668	29.5982	30.9846	29.6321	31.2981	30.4125	31.5021	28.7505	32.2364	29.8925	30.0013	30.6878	30.3345
25.6934	24.5692	24.8379	24.1921	25.4058	24.1222	25.5247	24.2845	25.9300	24.1777	25.2692	25.1026	23.8517
35.8063	37.2945	37.4089	38.2791	32.5057	38.0798	38.6865	38.0307	34.2264	37.8842	33.8961	37.4060	33.7384
35.8411	36.4765	37.9892	35.9438	40.0000	37.8359	40.0000	40.0000	38.5430	36.1602	38.2579	37.6825	38.0523
35.6285	35.6234	35.2644	32.5046	36.8202	36.0276	35.1365	36.5432	33.6738	33.6914	37.4850	36.8061	34.1283
23.4889	22.6084	22.3478	22.4493	21.8432	22.8223	23.3110	22.4501	21.9301	22.5736	23.2943	23.8624	22.4359
30.9256	30.4574	31.6568	30.0950	30.9187	30.2801	32.4616	29.3032	29.5143	33.1814	30.0099	31.4609	29.9079
27.7449	27.2776	28.5696	27.2350	28.7238	30.2232	29.9721	28.8904	29.1511	28.9153	29.9887	29.7474	28.7634
26.4075	26.8865	28.0273	23.9071	27.4153	26.8748	27.6979	25.9431	25.0903	26.6594	28.5193	27.4403	25.8881
26.3336	24.7802	26.6609	26.2185	26.0454	26.1302	25.9389	24.5388	26.0844	26.6653	26.7452	26.3302	25.8522
36.5352	40.0000	40.0000	36.7212	38.2522	37.5878	33.6699	37.3526	37.6183	40.0000	36.8227	37.8228	37.6661
30.2720	35.3717	34.0655	33.7178	33.8647	33.4417	35.6992	34.0815	30.9883	33.9908	32.8887	33.4015	31.2866
25.5921	24.0051	23.5079	25.8625	29.4155	23.5553	28.2146	24.8903	26.7574	25.2345	27.0226	27.1024	25.5418
27.6719	27.0902	24.9664	26.8097	29.2340	24.4777	28.2770	27.7384	28.4875	26.1272	28.1016	28.1722	26.6254
28.1525	30.7331	28.8736	30.6320	31.1356	29.5253	32.5569	32.6512	28.9279	30.8968	32.4819	31.9527	29.6358
31.3728	31.0244	33.6078	30.9701	33.2976	31.1119	35.3303	35.7295	32.4292	32.7913	33.2510	31.4819	32.5315
31.9283	32.3291	34.2091	31.8263	33.9242	32.6349	37.8190	34.6268	31.8150	33.4332	33.7641	32.5466	32.2626
29.4308	28.8912	30.6125	30.2631	30.1649	30.4508	31.8207	30.5245	29.1362	31.5753	30.9668	31.0642	30.5863
36.3914	35.0519	38.0605	36.6021	36.2770	32.8103	36.2502	35.1571	36.4280	37.1385	38.5314	37.5100	35.9120
31.4597	33.4934	31.7178	32.6954	36.0589	35.1304	33.7110	30.8298	31.0102	33.3383	33.8791	30.3380	31.2878
38.4715	37.9113	39.4044	40.0000	38.6316	40.0000	40.0000	37.8456	39.0166	36.4514	40.0000	37.9449	37.7745
30.6393	27.3063	31.5850	25.9144	30.8013	27.7555	28.7897	29.4754	28.9938	30.8468	28.9445	27.8139	30.5312
35.3133	37.6920	37.5031	38.0293	40.0000	37.4890	40.0000	38.4278	37.2158	38.4643	40.0000	39.1627	36.2518
28.9779	26.1734	27.5238	26.1323	28.5947	25.1367	26.3409	28.1559	28.4045	27.6756	29.5412	26.2250	27.8274
30.8657	34.4060	35.2546	32.7098	34.0089	33.2203	35.1226	35.8341	30.0915	33.7388	35.2953	34.7357	31.4229
31.2073	30.1900	32.8455	31.5654	33.1566	33.0344	33.7288	35.0668	32.9302	32.6446	33.0582	33.3248	32.3008
37.6878	40.0000	38.6544	36.9019	40.0000	38.4599	40.0000	39.3125	39.2426	39.1091	38.1929	38.0826	39.1214
33.4859	35.2844	36.8778	36.8005	37.8531	36.2145	35.7904	36.8668	32.1200	35.7975	37.7877	39.3084	33.6867
33.9706	32.9775	34.7816	33.7096	34.9498	36.2980	33.2874	32.7790	33.1390	33.1931	34.9586	34.6141	33.0675
40.0000	39.0674	40.0000	40.0000	38.5247	40.0000	33.4572	40.0000	40.0000	40.0000	40.0000	40.0000	40.0000
22.9497	24.8878	24.1784	24.8313	22.8248	22.3900	22.7126	22.6247	23.5484	23.4637	23.7792	22.8999	22.8432
22.1408	23.1250	24.3940	23.7463	23.5518	23.7396	22.6709	22.2832	22.0949	23.3019	24.5737	23.0912	22.9769
20.1720	20.1790	21.2454	20.6749	20.2138	21.6447	21.7618	18.6342	19.1508	20.5683	20.8669	20.1836	19.7010
34.2864	40.0000	40.0000	33.3230	40.0000	25.4514	38.7151	32.1363	32.2980	40.0000	37.5656	36.6581	35.5594
31.3989	30.3221	33.6379	30.8436	31.1141	32.0570	32.9630	30.4907	29.5319	32.9154	32.4640	33.3195	30.4656
39.2512	39.4494	38.9957	35.0253	37.4919	37.1930	40.0000	38.4818	37.4859	39.9427	38.7784	37.5920	38.3751
27.2778	27.9851	28.2759	27.2169	28.2026	27.2827	27.2655	26.2914	27.6624	26.6674	27.5047	27.1086	27.0583
38.2577	37.9167	40.0000	38.8254	38.1168	38.8557	37.9771	37.1167	37.8015	36.8422	40.0000	37.1112	38.7264
40.0000	37.3604	40.0000	39.2475	37.7717	40.0000	40.0000	39.3841	37.8687	40.0000	40.0000	40.0000	38.8675
39.2347	40.0000	38.3039	39.1144	40.0000	31.7144	40.0000	39.6814	36.9304	40.0000	40.0000	40.0000	38.8638
40.0000	40.0000	40.0000	40.0000	39.2444	40.0000	26.9699	38.7627	40.0000	40.0000	40.0000	40.0000	40.0000
37.7410	40.0000	40.0000	38.4973	39.4007	39.3912	30.1451	37.2364	38.9383	40.0000	39.8652	38.5668	36.7888
39.5783	40.0000	39.0090	39.9700	40.0000	39.3790	39.2063	40.0000	39.1007	39.8579	40.0000	39.7693	37.0422
23.9234	22.8690	22.4692	22.7334	24.3698	23.2592	22.7660	22.1934	23.7701	23.8013	24.8302	24.1884	24.8327
40.0000	38.8545	40.0000	35.7576	38.4808	37.4832	37.9291	38.8083	37.0416	37.9741	38.9604	40.0000	39.1119
27.3726	26.6187	26.5702	27.4699	27.7812	26.8969	27.9123	28.0103	28.0895	27.3438	28.2045	28.0266	27.6211
38.0060	34.9339	38.9331	38.6241	39.0244	38.0006	38.4448	38.2042	38.3805	39.2713	38.8863	39.1706	40.0000
29.7045	32.2222	29.6983	30.7626	30.2550	30.1576	31.7990	30.9166	30.6173	30.6965	31.0079	30.1728	30.7572

Table S3. Median Ct values of plates A and B.

27.6474	27.6672	27.5348	27.0992	27.8033	27.9472	28.0644	27.6469	26.6807	27.7348	28.4407	27.9607	27.8854
32.4005	32.9300	33.2132	32.2159	31.7049	34.9550	33.6012	32.8263	32.8129	32.5942	34.0470	33.0054	33.7509
27.4947	26.4926	26.8950	27.2306	26.7132	27.4597	27.2883	26.7211	26.5302	27.1090	28.7809	27.6247	27.7460
25.6038	27.1249	26.5661	28.0304	30.9013	26.6589	33.3003	24.5149	26.8496	31.7243	30.0830	26.3650	27.9638
38.2080	39.8840	40.0000	40.0000	39.3427	40.0000	40.0000	39.6784	40.0000	40.0000	38.1277	40.0000	40.0000
29.6178	32.6126	35.9565	31.1677	31.3966	31.2293	31.2279	29.8459	32.1491	31.8205	29.6528	29.9356	30.9281
27.0764	27.3376	29.1179	26.1224	25.5494	27.9679	27.1327	27.3328	25.1966	25.8263	27.5419	27.4617	25.4185
28.5003	27.8084	29.6699	29.1126	29.5088	28.4700	29.2396	28.4786	29.4860	30.8035	30.0503	28.8801	29.1448
23.8397	22.3157	26.5710	24.9260	24.0587	24.6160	26.6403	24.9338	25.6183	26.5307	26.0701	25.4586	25.9526
39.9478	40.0000	40.0000	40.0000	40.0000	40.0000	37.1525	40.0000	40.0000	40.0000	40.0000	37.7173	40.0000
37.3363	40.0000	40.0000	36.1399	38.7437	36.1799	40.0000	40.0000	36.5049	40.0000	38.1204	38.9995	40.0000
35.0609	40.0000	39.6631	35.7747	36.5238	38.5410	39.4825	35.9687	34.1637	38.0074	36.8456	37.2789	35.9660
39.2557	40.0000	40.0000	40.0000	38.9768	40.0000	39.3634	34.2346	40.0000	39.9819	34.4637	38.7296	38.9440
37.1420	40.0000	40.0000	38.0898	40.0000	39.4328	40.0000	39.3108	35.8207	39.7579	39.8622	40.0000	38.8735
35.9905	36.6934	35.0090	37.2290	36.1550	31.9409	33.6781	31.4597	32.5444	32.4072	32.7884	32.1198	33.4038
37.2957	40.0000	38.7074	39.6716	36.3477	35.6703	36.3084	34.5738	36.6301	35.6019	36.8038	35.0186	37.4501
28.0610	26.0602	27.9013	26.5319	28.1872	26.0046	27.9303	28.9950	27.6629	26.1444	28.6028	27.5089	27.9572
23.7923	23.1880	24.3201	20.3159	23.4793	22.9325	22.9962	24.6264	22.1431	22.3387	23.2402	23.4467	23.1445
24.5719	24.1085	24.5023	19.3152	23.2804	24.1693	23.7907	27.0952	20.9691	20.8519	22.6864	23.0491	21.8511
26.5099	22.8372	24.0449	20.4903	22.9550	24.6509	25.6077	25.7752	26.5892	22.4118	26.8683	25.4801	23.4225
30.9230	32.6106	36.1818	28.4707	33.8878	30.5707	28.4549	33.0627	31.3907	33.1709	33.6548	31.5066	33.8365
23.0717	21.7969	24.7417	21.7774	23.3776	23.2319	23.3825	23.5086	23.5238	23.5130	24.4653	23.9509	24.2923
25.4273	23.8026	25.6462	23.1892	26.3439	25.4600	26.0983	24.9120	24.2021	25.9517	26.6041	26.1737	25.7843
38.4391	39.1995	40.0000	38.1291	40.0000	38.3167	40.0000	40.0000	37.4265	38.9523	38.3052	40.0000	39.4230

Table S3. Median Ct values of plates A and B.

50	51	52	53	54	55	56	57	58	59	60	61	62
35.2185	33.8384	29.4702	29.3373	28.2897	33.5581	38.5287	23.9057	28.7874	33.3868	28.4107	30.4965	34.5513
40.0000	36.6917	34.3580	33.4644	31.7729	36.7628	40.0000	27.7003	33.3325	37.5338	32.5815	32.8150	38.2516
24.8412	23.1780	23.5770	23.6735	34.8471	26.6757	38.6239	20.9254	24.4747	33.7286	23.1337	23.6803	36.8568
25.3643	24.4394	23.8800	25.5401	22.9999	26.5140	27.6786	20.9167	26.4844	26.7748	25.0425	25.4021	25.0163
23.0751	21.6349	23.6272	22.5616	23.8070	23.1000	26.6656	20.8893	23.3499	23.5858	22.7774	23.6124	23.8934
21.6367	20.2651	21.0088	21.8845	21.4565	22.0658	22.6849	18.2789	21.4207	20.8683	20.8387	20.8444	20.5871
27.2625	26.7089	27.2804	28.0976	29.4282	28.0733	28.2962	25.7142	27.3262	18.7170	27.8075	28.7289	27.6687
22.1293	22.4583	21.2321	22.8464	25.8759	22.6723	22.7035	21.2087	22.9293	20.3837	23.3648	24.3487	23.0213
21.5281	21.7054	24.4500	21.8449	23.5714	23.5351	27.2936	20.2190	22.0587	23.5454	21.5659	22.7778	21.6635
22.2558	21.6037	24.5593	23.4513	25.0250	24.4006	26.7079	20.3734	23.8945	25.7908	22.9658	24.4907	23.2741
22.7217	21.4583	19.2966	23.6437	19.4644	24.4709	24.1778	17.3984	23.1033	23.9534	22.5456	20.7420	21.4072
22.9976	22.8483	22.4260	24.5662	22.8362	25.3427	25.9151	21.1629	24.4517	23.7858	23.8834	23.6265	25.2681
27.8578	27.2871	26.9624	28.2116	28.3266	28.1812	30.8972	25.4413	25.5071	28.9738	28.5063	27.8646	28.1520
23.5808	23.7622	24.0528	24.2721	24.5321	25.0005	24.6435	22.8910	25.0604	24.5332	23.2252	24.6907	23.4975
22.9248	21.5889	23.9689	23.1753	23.7870	23.2779	24.5790	19.8534	23.0101	22.0012	22.8785	22.1039	21.9483
24.5703	23.3475	25.8216	25.1533	23.9026	25.3214	25.8916	20.7335	24.8154	23.1142	24.9324	24.1885	24.2395
24.7740	23.8832	22.5933	26.0891	24.9929	25.9715	25.0906	22.3993	24.9133	24.1403	24.0953	24.3721	26.4845
26.5441	26.3607	28.2935	27.5281	27.4467	28.1634	30.3469	24.8025	25.7443	30.1489	26.9531	27.1081	27.3444
27.4111	27.7461	27.4494	28.7722	26.4070	29.2306	32.4033	24.5689	29.4407	30.9227	28.2289	27.0865	28.1381
23.0848	22.4664	23.6572	23.4721	23.6658	25.1588	25.3950	20.6753	23.6152	25.8079	24.0154	22.6347	22.2569
24.4035	23.3921	27.5778	25.2463	24.6458	27.0008	30.4099	21.9417	25.0681	27.2673	25.3732	24.5778	24.6972
21.9755	21.8510	22.8953	22.5479	22.3389	24.2809	25.2284	19.9638	22.5265	25.0879	22.8959	22.1690	21.7093
28.1446	28.0858	27.0947	28.4622	29.6513	29.5379	30.4020	26.0373	28.8875	28.8912	28.8228	28.6649	28.5970
23.0117	22.4494	22.4142	22.5523	22.6873	22.6717	22.9656	20.2109	23.3771	22.9396	23.2902	22.4236	21.5239
23.5720	22.9045	24.4756	23.7053	23.3371	23.9211	25.9693	21.2060	24.6338	23.4941	23.7826	22.8030	22.9274
25.1506	24.5363	25.5204	24.4657	25.2323	25.3664	25.1580	22.5334	25.4298	24.6212	25.3066	24.2756	24.3095
25.7987	25.3768	25.6437	26.1810	26.7406	27.4394	26.9504	23.6052	26.2816	26.3618	26.5419	26.3680	24.8350
40.0000	30.7349	36.4347	37.2268	38.2856	38.4149	39.1492	31.0691	32.3966	32.4097	40.0000	37.6428	37.3025
25.8866	25.9841	25.9651	25.9236	24.1262	26.5858	29.7740	22.3007	28.0654	29.7414	27.0593	25.0125	25.3704
40.0000	35.0629	40.0000	36.9459	36.6090	36.1438	37.5760	37.4204	40.0000	40.0000	40.0000	39.7295	37.2913
40.0000	35.5691	40.0000	38.3087	36.5493	37.3953	39.1772	36.6225	40.0000	40.0000	39.0342	40.0000	37.6670
20.3328	19.8394	23.1674	20.9465	22.3482	21.4813	25.6332	20.1833	24.1325	24.7178	20.4207	21.4852	21.1962
35.4585	32.6873	35.4001	34.4363	34.6918	35.1135	38.5641	31.8687	34.7529	35.3470	34.2473	33.4681	34.2701
37.3469	31.7313	37.8216	31.9816	40.0000	37.6484	39.2541	35.1591	36.7610	38.4775	37.7288	38.3573	37.1484
27.1015	27.3115	27.9609	28.5446	27.0499	28.7075	28.3898	24.5977	30.2850	27.7507	29.2146	28.5810	28.3853
26.6797	25.4336	31.9260	27.0100	33.1061	26.8696	33.6118	29.2652	28.2956	26.9877	25.6699	29.8510	27.0378
26.9966	26.4512	29.2362	27.0240	31.7698	26.9389	33.0848	27.5364	28.3366	27.1194	25.1210	29.4719	27.4733
23.4465	24.3108	23.3291	24.8180	22.7898	23.9507	24.4801	20.8224	25.5623	22.5194	25.1896	23.7649	23.9537
35.9444	36.9771	36.9030	39.6972	40.0000	37.3213	40.0000	36.4175	38.1010	36.9633	39.4588	34.9505	37.4111
37.5052	36.9118	40.0000	36.7053	40.0000	39.3338	40.0000	36.8643	37.9510	37.5015	38.9954	36.3633	39.1797
22.2922	23.2342	23.6848	23.3259	26.5054	24.3661	25.7521	21.9851	24.6330	24.2419	23.7558	25.0260	23.8888
29.7672	30.1600	30.3215	30.4531	29.4096	30.9906	31.6010	26.9605	32.1965	29.0516	31.2023	30.3475	30.7626
36.3130	37.6746	39.2379	40.0000	39.2039	37.1726	40.0000	40.0000	38.4805	40.0000	38.2468	36.9821	40.0000
35.2010	33.4683	31.4641	34.1138	37.3633	35.3317	36.4374	31.6058	34.1876	36.2005	34.9600	33.9630	38.2436
28.0507	29.7037	29.4797	26.3718	34.7127	31.6715	31.1228	27.7732	28.3455	12.1546	28.9948	28.3929	30.4021
25.4215	27.8907	30.8831	26.9607	25.3406	37.2288	26.4813	25.5751	26.8906	36.6521	25.0666	29.4477	25.2495
28.0813	26.3364	32.2046	27.0582	37.8258	36.9961	40.0000	28.0367	36.4111	30.8826	26.1351	30.1787	36.2748
23.3390	23.5843	31.6829	25.4750	31.7882	26.5365	26.7095	23.2426	26.5023	25.0547	29.2009	30.7913	25.6432
33.3359	32.4729	37.7630	37.8448	40.0000	40.0000	40.0000	34.4436	40.0000	40.0000	34.9909	38.0585	40.0000
28.9134	30.4892	31.3224	31.1009	32.3865	32.1792	34.2780	29.0732	33.1281	33.8821	32.0251	32.2076	28.4369
29.2315	31.8143	33.9107	32.9624	34.7915	33.6974	35.5644	31.0757	34.2721	34.2961	33.2592	33.0894	28.8936
34.9579	36.2718	39.3860	36.3268	38.2089	36.4873	38.8442	36.4194	37.3550	38.4546	36.6243	35.9452	36.0811
24.6175	26.7682	30.6636	24.1658	30.5133	24.8132	25.4455	25.7273	25.3436	25.0396	25.4610	29.6816	23.5555
25.7118	25.1808	25.4707	26.3719	26.3680	26.2662	26.1982	25.1759	27.0988	26.0564	26.0114	25.5101	24.8019
30.7575	29.2617	28.9647	30.4403	27.7837	31.2632	30.9736	27.9184	30.5379	29.7852	31.2455	27.5318	30.1888
29.0006	27.3810	33.8249	27.6521	37.4359	27.9947	34.9290	29.0153	29.9428	38.0601	28.0416	31.7481	34.7642
29.1099	27.3471	34.9150	27.7739	37.1410	27.5354	34.9144	29.1078	30.6172	36.4786	28.8401	32.5599	35.3929
35.1109	33.8609	36.6038	36.2699	39.1155	39.7017	37.1343	35.4418	36.0112	36.9309	36.7142	36.3285	37.1420
32.6986	32.0796	36.4842	31.1492	38.0409	31.1229	36.7670	31.1081	32.3543	33.8401	26.3125	34.9634	34.4261
36.4100	32.6182	36.5854	33.9634	37.5194	36.2195	38.6198	29.0068	34.6698	34.9414	30.9770	34.5696	37.4038
38.8341	37.4381	37.6203	36.7086	38.8609	40.0000	40.0000	37.4434	37.8359	40.0000	35.8387	37.0027	36.9325
35.9419	37.7293	38.5472	36.8399	40.0000	37.4401	40.0000	34.7390	35.8769	39.0076	39.2327	37.7765	39.2811
33.8618	31.8736	35.9382	31.8167	37.8017	36.0162	35.3030	31.9706	34.2384	33.2611	35.1126	32.6093	34.8879
25.5835	25.0531	24.4769	26.4697	24.9289	26.7071	26.5475	22.7628	26.0322	25.5629	26.0650	24.5280	25.1816
31.8546	32.1096	29.9040	33.4103	29.8322	34.8635	33.1120	29.6647	32.0390	32.7156	32.2954	31.7274	31.3016
19.7222	19.6449	18.8715	20.9537	20.3585	21.4637	19.9667	17.5792	20.2100	19.5618	21.1856	20.7622	19.5068
24.8728	24.3810	23.5280	25.0682	24.9319	25.4816	25.8794	22.1252	25.7053	24.4977	26.1284	25.6628	24.3403
36.3776	34.5324	40.0000	34.4041	40.0000	40.0000	40.0000	37.4030	36.9481	40.0000	39.8250	38.3378	40.0000

Table S3. Median Ct values of plates A and B.

29.9806	27.2375	27.3679	26.8258	28.7853	30.0047	30.1138	26.9336	29.1941	30.7222	30.7678	27.8308	30.9634
23.5583	22.2800	24.5449	23.3574	26.3587	24.1460	24.3091	23.0389	23.7252	23.7769	22.0961	23.7947	21.3965
32.8309	37.8634	38.1037	39.6014	36.9706	38.1350	37.6333	36.3028	36.0918	38.1196	36.6463	38.3507	38.3332
31.8034	27.5335	24.2434	28.8953	28.0166	32.0777	33.5406	24.6555	30.4025	34.2834	25.5475	27.9861	30.8514
35.1023	33.7841	33.8188	35.4542	32.6474	36.8700	33.3368	33.9676	34.5157	35.3696	32.7285	30.9132	33.0196
26.7079	21.0660	25.4689	24.9902	25.3871	24.8784	27.8250	21.7467	23.1761	24.2251	24.4675	23.8134	23.8500
28.4797	28.2795	30.2972	31.3340	32.0408	33.8560	33.8916	28.6381	31.4438	32.5894	32.6732	30.4532	32.1890
30.9242	29.7309	26.9867	30.9290	27.7504	33.0651	33.4097	25.7316	29.9823	30.3000	31.5570	28.5104	31.1303
22.7577	22.5091	21.8656	23.4234	23.1869	24.5800	23.9789	19.4784	23.8329	22.5849	23.0110	23.5994	24.3939
39.6809	39.2583	40.0000	40.0000	33.6474	40.0000	40.0000	40.0000	40.0000	40.0000	40.0000	39.6693	39.5363
33.7626	37.4255	37.0986	38.1709	37.5475	37.4111	39.6240	37.8280	36.8317	39.6748	36.6591	34.8494	40.0000
21.7608	19.9975	19.6933	21.5146	21.1398	24.3137	21.4799	18.7160	20.7456	20.5534	20.4975	20.6463	20.7978
34.8954	34.0623	36.6581	37.8107	38.7441	40.0000	38.4572	37.7064	35.5527	37.6260	35.5875	37.9261	36.1485
34.7923	34.4029	36.4798	36.3819	37.8573	40.0000	40.0000	38.8033	35.3147	40.0000	33.8858	40.0000	36.6925
23.8532	25.4947	26.0317	25.5513	27.3940	25.6852	26.4259	24.2355	25.4965	27.1406	23.9058	25.5935	25.4988
25.8711	21.5768	22.5833	22.0314	23.3576	22.3741	20.5511	20.9190	21.3071	22.0415	20.4501	22.6478	19.7874
25.9174	23.3419	24.3396	23.8074	23.5949	22.6430	21.3750	21.8817	23.1237	22.9112	21.5963	23.6724	21.2580
29.8923	27.1585	28.3192	27.3418	29.5130	27.5791	25.4861	24.8486	26.6732	26.5307	25.5642	27.2840	24.9428
34.9327	30.3047	36.0325	30.1881	37.3193	34.5532	35.5967	32.1715	32.6396	35.8602	35.0211	34.4818	34.8883
35.8504	35.7952	35.2602	35.2224	38.3885	36.2778	35.3852	35.9311	34.6447	34.8935	36.1296	36.0206	37.1455
40.0000	36.1108	38.7705	35.7311	40.0000	40.0000	40.0000	37.3608	39.4500	40.0000	39.2480	40.0000	40.0000
37.4397	36.7845	35.4522	36.2165	38.6419	35.0553	36.4499	37.9061	36.9514	37.1513	37.3731	37.4648	38.5348
30.6121	29.1911	28.2697	30.0751	29.9137	31.8324	31.6872	28.6201	30.1842	31.5026	30.3419	29.3568	29.2312
24.4928	24.2769	23.7777	24.8580	25.4404	26.0607	25.7962	22.8434	25.4406	25.6771	24.4427	24.4929	24.4219
40.0000	36.2051	31.4309	38.0217	31.5012	37.6033	39.5166	34.4945	36.6070	36.9596	36.5987	34.4536	37.1067
38.4235	37.7485	36.0891	37.6445	35.4524	37.1644	37.7840	34.3839	34.9046	37.3845	37.9853	38.6793	37.4017
33.8139	34.1767	34.7907	34.5441	36.1411	35.6347	35.6165	34.3202	35.1958	36.1498	33.6009	36.8472	35.9958
23.4074	22.4830	21.4586	23.4762	22.4641	23.4222	23.1765	21.1257	23.0158	22.0832	23.1022	22.4192	22.5631
34.1845	33.5683	28.0101	32.1737	29.0037	32.8826	31.4339	29.3407	30.6824	29.0356	32.4993	31.5739	32.1897
29.4343	27.2367	28.9682	29.1779	28.4288	30.5345	29.6443	28.0568	28.7500	29.5748	28.2074	29.2020	28.4859
28.1159	26.1441	26.6317	27.3231	28.5354	29.6884	26.7749	23.0500	26.9152	25.8270	26.6760	27.1337	27.6637
26.2125	26.9648	24.5187	27.1624	24.9017	28.1693	26.3271	23.6105	26.9814	25.9379	26.4541	24.8538	26.1284
37.3095	38.1321	36.5372	36.2852	40.0000	38.3158	37.4692	36.4609	36.8971	37.0598	36.9702	37.4750	38.3092
36.4858	29.0006	31.7245	29.2729	36.9869	33.7786	34.6288	27.8697	29.5928	35.1720	32.2044	34.1512	32.3060
25.9832	40.0000	26.5036	24.8177	28.4287	25.5100	25.6471	26.4575	25.1966	24.6859	24.8315	40.0000	29.7671
27.2162	27.1582	27.6746	25.7119	28.1791	26.1941	27.7404	26.5590	26.3216	25.8768	27.0727	28.1405	28.8878
32.0683	28.1419	30.9101	27.3278	34.4005	30.7776	29.9167	27.6900	28.5850	30.4480	30.3654	32.0334	30.9439
33.8280	32.9312	30.2187	35.1466	32.0071	33.5800	33.8930	30.6473	32.5511	33.3922	32.3423	33.6347	32.7806
36.0186	32.9795	31.2966	34.4553	33.3964	33.8422	34.4366	30.2350	32.7435	33.3291	31.7324	32.0680	33.4046
31.3685	30.9764	28.6634	32.6855	29.0786	32.4147	31.1107	28.5023	30.5267	30.1376	31.1543	28.9047	30.9890
37.4377	37.1105	35.7457	37.5191	38.3887	36.3642	36.1758	36.1092	34.4213	34.6746	34.0220	36.3955	38.4393
33.7654	31.2127	30.3382	33.7392	36.1590	32.2933	30.8999	32.9446	31.3261	31.4310	35.9689	29.4170	36.0902
40.0000	39.5030	35.6908	38.8574	40.0000	40.0000	37.4258	33.3043	37.3216	38.2863	38.5727	34.5463	38.6182
29.4363	28.4172	26.0161	30.1622	26.7572	30.9169	30.5049	25.6205	30.7843	31.4361	28.2329	27.1577	27.6860
35.7251	35.9470	39.9069	37.0322	40.0000	36.8663	36.5193	37.7020	38.2347	35.1135	37.8865	38.1878	40.0000
31.5726	26.7505	27.4239	27.6734	28.4382	28.1313	26.5214	27.0100	27.1205	27.5763	25.5981	27.5998	25.5522
32.9580	32.1572	32.1935	29.1545	34.5915	36.8495	32.2612	32.8576	34.5182	34.2071	33.6819	33.1074	33.5421
31.9976	30.8970	30.7951	32.2619	31.4221	33.7662	31.7551	32.3214	31.9966	32.4423	31.5480	32.8348	32.1836
39.3667	38.6241	38.7892	38.6276	40.0000	40.0000	36.1947	39.8082	37.3918	37.7320	34.1088	39.4199	40.0000
39.9686	34.4952	34.2165	31.0091	40.0000	35.5366	36.0026	36.7763	38.3600	37.1728	34.8865	37.1064	37.3411
33.6593	34.1650	33.3689	33.9794	37.3177	35.5295	32.5177	35.0942	33.7706	33.3192	33.7632	36.1281	32.9148
40.0000	38.0801	40.0000	40.0000	40.0000	40.0000	39.6384	40.0000	40.0000	40.0000	40.0000	40.0000	40.0000
24.1719	24.0776	21.5357	23.8831	23.0680	24.8257	23.1144	21.4914	23.4146	21.9804	25.6507	23.6720	22.6916
24.4580	23.5710	21.9124	24.4975	23.3347	25.0462	22.1740	21.7176	23.6247	22.4428	24.9564	23.4210	22.3070
21.2867	20.3691	18.5246	21.1128	20.9260	21.6178	19.8705	18.5839	20.9387	18.9497	21.2913	20.4159	21.7649
36.9237	34.0599	40.0000	33.2823	38.8142	38.3680	40.0000	36.1839	36.5314	30.2610	31.3685	40.0000	37.7757
30.9152	29.3834	31.0277	31.9341	32.1703	34.7112	32.9752	31.6037	30.7765	32.2329	30.4552	31.0734	32.8932
35.2229	34.8090	38.7078	37.1686	39.9941	40.0000	40.0000	38.1090	37.4799	39.2463	35.3115	38.4029	37.2554
27.7809	27.8836	26.0645	28.4523	27.9316	29.9703	28.0958	25.8327	28.3396	26.8378	28.6892	27.9848	27.9164
36.3497	37.7846	36.3728	39.1917	40.0000	39.3417	38.9248	38.5263	37.8094	39.5418	38.1302	38.0217	38.9151
40.0000	40.0000	40.0000	39.1933	40.0000	40.0000	39.9659	39.1908	40.0000	36.0173	40.0000	39.0552	40.0000
38.9238	40.0000	40.0000	37.1045	40.0000	40.0000	37.0607	37.9581	36.3694	36.7435	37.2512	39.1684	39.7669
38.4083	40.0000	38.8993	40.0000	38.8919	40.0000	37.6358	40.0000	39.3189	38.3496	39.7500	39.8858	39.0163
38.5670	39.2748	39.3735	38.4998	40.0000	40.0000	37.8736	40.0000	38.3838	39.9855	39.4511	38.6012	39.6010
37.7505	38.1479	39.7618	39.6203	40.0000	38.2827	38.4593	40.0000	38.3757	38.3805	37.3107	39.4007	38.2055
23.6588	23.9362	23.1988	22.9174	24.4428	24.4316	23.2751	22.6376	24.4706	24.3234	24.0831	23.7637	22.6419
36.6604	34.6882	37.3439	39.6851	40.0000	38.1011	37.4216	37.7787	36.8109	39.4854	35.8178	40.0000	37.6965
27.4715	27.9165	26.5056	27.9366	27.7257	28.6554	27.4677	26.2947	28.8075	27.5841	28.2596	27.3713	27.4499
37.8748	36.6536	37.2467	37.9049	40.0000	38.8191	36.1231	39.0386	38.5577	37.0007	38.0382	37.3183	39.8780
31.3147	31.7909	28.9921	31.3851	30.8069	31.6797	30.2082	28.9880	31.3061	29.6769	31.6842	29.2681	31.1746

Table S3. Median Ct values of plates A and B.

28.2107	28.2645	26.8665	28.2229	27.5825	28.7161	26.5913	25.6490	27.9080	28.1068	28.1541	26.6730	28.0199
33.7643	32.3015	31.4336	34.7955	32.1048	33.3685	32.3766	31.4563	33.2733	33.1286	33.8484	30.9716	33.3255
27.1450	27.4887	26.3263	27.6113	27.5711	27.6876	26.3755	25.6605	27.8326	26.9715	27.6442	26.9269	26.1742
26.5384	28.7610	28.5472	27.9091	29.6384	35.5318	26.0160	28.4714	26.9912	24.5725	28.1610	32.5228	24.7800
40.0000	37.6806	40.0000	39.8416	37.7951	40.0000	40.0000	40.0000	37.2429	39.0410	38.7855	38.1729	38.0987
31.7716	31.0840	27.8782	31.4402	30.1947	33.4901	34.8798	27.3789	30.8878	32.0540	32.7959	31.3188	32.9342
26.4378	26.7782	24.0869	26.2108	26.6129	28.9919	29.1122	24.0290	26.8311	28.9712	26.5864	24.4538	28.5688
31.7340	29.7379	27.2097	30.5261	29.6095	29.9945	29.5225	26.6824	30.2432	29.1891	28.7236	28.8153	28.5834
26.0677	23.6639	24.2155	25.1734	26.6250	25.5132	26.3916	23.0669	24.4923	26.0907	25.4615	24.3157	24.3655
40.0000	37.8512	36.9974	39.0340	40.0000	40.0000	40.0000	40.0000	40.0000	38.4307	40.0000	39.9495	40.0000
35.8763	36.0693	35.8188	40.0000	40.0000	37.5032	40.0000	37.8623	36.7553	37.9123	35.3497	40.0000	36.2153
34.3821	33.2423	35.2052	36.3657	40.0000	39.2963	32.7850	38.5935	34.8108	34.3496	35.3700	35.7937	35.9689
40.0000	38.2983	39.3325	39.0437	38.2221	30.8990	38.3953	40.0000	39.3170	39.8631	40.0000	40.0000	39.7649
40.0000	40.0000	39.2062	39.1416	40.0000	40.0000	39.0861	40.0000	40.0000	40.0000	40.0000	37.7634	40.0000
33.9781	32.4455	30.0947	32.6838	39.1308	34.7730	34.7750	35.7549	31.2136	33.3352	34.1973	31.2347	33.4004
34.9050	35.1167	34.4748	34.9787	40.0000	37.7414	39.0779	37.9314	36.8248	38.0822	36.3468	33.5814	36.5727
27.2013	27.0597	25.8215	27.8526	27.1895	29.7206	28.3626	25.6760	27.6589	27.6674	25.8969	26.9035	26.8168
23.4031	22.2199	20.9091	22.7155	22.3953	24.5604	23.7241	20.0464	22.8947	23.6098	21.9946	23.1138	22.7802
22.4185	21.0355	19.9307	22.3351	21.6433	24.6246	24.8076	19.5151	22.2259	25.7397	21.0164	21.9159	29.2387
23.0164	22.0359	25.2235	25.9848	22.7147	26.3306	25.8935	20.2087	26.4460	26.3445	22.7034	23.1692	24.8919
30.5968	31.8443	32.2744	31.0582	29.1576	35.1007	30.9794	28.7236	31.0208	30.5607	29.4976	32.1882	29.4140
23.7573	22.5999	22.6060	23.9171	23.0728	25.1527	23.7411	21.4586	23.8162	23.5388	23.0576	23.8610	22.5962
25.2564	25.3869	24.7763	27.0786	25.7524	27.9058	24.9062	22.5333	26.4317	24.9930	25.5106	25.8777	26.5094
40.0000	37.5720	38.6527	40.0000	39.6537	39.5060	38.2806	38.7251	38.6829	38.3553	39.1925	37.4952	38.7790

Table S3. Median Ct values of plates A and B.

63	64	65	66	67	68	69	70	71	72	73	74	75
26.2266	27.4308	27.3556	34.1304	37.1823	28.7571	32.6845	33.4479	29.9902	31.1517	28.7763	33.0066	31.7326
30.4483	30.5376	31.8543	39.1493	39.4406	32.4627	35.4052	36.0760	33.2625	35.3445	32.7715	36.4302	35.6287
22.0996	20.9589	26.2550	35.5763	35.4255	22.9770	22.8998	34.0497	24.0411	27.4484	21.5940	33.7369	27.2866
23.5478	22.2172	23.2218	25.9951	25.9270	25.0125	23.9368	21.8726	25.1196	25.3574	23.0053	24.1262	25.5712
22.0022	20.7505	23.3670	23.9359	24.3893	23.2976	22.9770	21.4113	24.1495	23.8495	21.3948	22.9779	23.6712
20.1216	19.1168	21.4469	21.4414	21.8275	21.0342	20.1221	19.7149	22.0151	22.5003	19.5796	21.2118	22.0918
26.1798	25.9334	28.5236	27.2284	28.6616	28.2567	26.2851	26.1660	28.7110	29.1688	26.0953	28.1572	28.4700
21.8221	21.1494	23.3926	23.0050	23.5326	23.2365	22.2341	21.3028	24.4173	24.4297	21.1855	23.5848	24.4058
20.9553	21.0953	22.7990	21.9641	23.1942	21.9329	21.4485	20.3631	22.4651	22.5815	20.8276	22.9057	23.3821
22.5519	20.8684	24.6545	23.3588	23.7563	22.8527	21.8221	20.2594	23.7288	23.5389	21.8132	23.7555	24.1221
19.7249	19.5664	21.3771	23.0593	23.3649	22.2446	21.7053	20.1104	22.8974	20.3171	20.6136	19.7298	22.3295
22.0262	21.7883	23.9093	25.0695	24.4974	23.3908	22.3344	22.0584	24.0123	22.1970	21.7001	21.1202	23.3295
26.9237	26.2639	28.1915	28.8187	29.0566	28.4934	27.1610	25.3429	29.3685	27.6975	26.1247	27.3339	27.8547
23.4760	23.0181	25.6317	23.7229	24.5457	23.4985	23.3398	21.7390	24.6444	24.2899	22.6000	23.5732	25.0150
22.5835	21.0347	24.0112	24.7934	23.0474	21.9654	22.3890	20.2761	22.8944	23.3347	21.0756	22.6610	23.6852
23.5743	22.6220	24.4586	25.3791	24.4920	24.0471	23.2334	20.5494	24.5514	24.8138	23.0953	24.0348	25.1352
22.5626	23.7143	24.0996	27.1895	25.5298	24.0174	23.1247	23.1595	24.8461	24.1642	22.1201	23.2940	24.9132
25.9048	25.7891	26.7611	27.9752	28.0713	27.4846	26.6688	25.1538	28.6745	27.2091	25.2285	26.5637	26.1878
26.9964	25.7805	26.9744	28.8947	29.4458	27.9590	27.6462	26.2253	29.7282	28.7396	26.6912	27.0666	27.9177
22.9856	21.8051	24.3347	23.5459	23.5859	24.3887	22.7982	22.3498	25.5031	23.5119	22.0094	23.6843	22.9464
24.1899	23.7404	26.2825	26.0286	24.9876	26.5968	24.4169	23.7732	26.3002	25.0089	23.2923	25.5935	24.6853
21.4970	21.1460	23.7349	23.7205	23.2498	23.4108	21.9504	20.6102	24.1334	22.6118	20.8451	22.7495	22.5719
27.6950	26.6023	28.4985	28.4295	28.8667	28.0848	27.7876	26.0759	29.2537	28.2618	26.4014	26.9207	28.2190
22.1324	21.5484	22.6794	23.2954	23.2540	22.3108	21.6722	19.4205	23.7708	22.5977	20.8306	21.9317	22.8266
23.1079	21.9215	23.4831	23.9658	24.7643	23.1608	22.6270	20.9434	24.8380	23.3933	21.9900	22.5003	23.5809
24.1233	23.4436	24.8373	26.3977	25.1051	24.6151	24.1984	21.6364	26.7022	24.3356	23.5900	24.2178	25.0074
24.8135	24.8706	26.5453	26.4252	26.0845	25.4613	24.6623	23.1972	25.3389	25.1473	24.1438	24.9956	25.9424
39.6990	34.4994	37.6620	39.4937	34.4960	39.4021	35.0093	38.5426	38.6596	37.7245	34.5864	33.9945	31.5831
27.0618	23.5124	24.6200	25.9951	27.0762	27.7154	27.5684	26.3829	27.6551	26.6830	24.8208	27.2324	26.6409
40.0000	38.0952	37.3542	36.8555	37.7593	40.0000	38.2510	35.3289	38.4745	40.0000	35.3463	37.7877	34.0164
40.0000	36.9477	38.5803	37.9385	38.1054	39.3185	39.8510	35.4627	40.0000	40.0000	36.0904	37.8311	34.4246
21.2951	19.1861	22.2160	22.5336	22.6774	21.5393	20.9846	19.4725	22.9643	22.6173	19.4103	22.3389	21.8179
35.1634	33.0433	33.5153	34.8702	35.5932	35.5268	35.4231	33.8464	35.7209	34.5358	33.8969	35.4327	33.1054
35.2806	35.8887	37.1916	32.3244	40.0000	39.4118	37.9542	30.6121	39.5112	32.7995	37.6178	39.1403	35.2832
27.3201	26.3245	27.1887	28.1988	29.3978	28.1375	27.3415	24.9629	29.7694	28.2890	26.6881	27.4044	28.3902
28.4165	26.9604	33.4000	32.7712	33.5890	27.3023	25.8025	25.7846	27.1069	27.3078	25.9161	26.7114	27.0896
27.3603	26.5129	32.6289	32.6162	32.7932	27.9700	25.9136	25.8649	25.8759	27.8092	25.5831	26.6132	26.9032
22.9857	22.6305	22.7037	24.8520	24.5355	24.9731	23.2974	21.1377	26.1327	23.4151	23.0274	23.0132	23.5361
35.1570	36.7627	37.4262	38.3273	36.8439	39.4471	36.1732	38.5532	38.1940	36.3866	35.7172	36.1561	36.8784
36.1249	36.4383	38.1631	40.0000	38.4625	39.2144	37.9003	35.9756	36.1142	38.0331	35.4660	37.2865	37.9661
22.8944	22.3233	24.4754	23.4291	24.1890	24.3912	22.7511	21.6992	25.0867	24.1035	21.9272	23.9755	24.1874
29.1523	28.8828	28.9111	31.1957	31.4260	32.0998	29.7659	27.3323	32.3970	29.5692	29.4406	28.8255	29.5791
40.0000	37.6585	40.0000	40.0000	39.7943	40.0000	40.0000	37.7707	37.1278	40.0000	37.7645	38.2835	40.0000
32.7010	33.2986	32.7002	37.7431	36.4695	33.9186	35.8089	34.4955	34.2885	35.2498	32.7065	35.2559	29.2685
27.3262	28.0121	30.3026	31.4736	27.8601	29.7951	28.7509	30.2490	29.0277	27.4583	30.8530	29.1728	28.0909
29.7653	25.9468	26.5671	25.9499	26.7688	27.4851	28.0606	26.2933	26.7507	26.4320	23.2576	25.9020	25.0836
35.9813	26.4731	40.0000	40.0000	40.0000	28.7057	37.0607	24.7818	26.8778	27.1459	25.5757	27.4343	27.8763
27.2444	23.8114	30.7445	32.2208	22.8386	29.0950	25.6390	21.6778	28.3980	25.6785	27.0416	23.3805	26.4280
40.0000	35.4242	40.0000	40.0000	37.7393	38.5152	40.0000	33.0989	37.5716	33.0506	36.4094	35.5672	32.1928
30.1382	29.1444	31.2326	25.9794	31.8957	31.5313	30.3065	24.6411	32.0037	30.6410	29.2989	29.9997	31.5210
31.5958	31.4437	32.9710	26.7742	32.9546	32.8325	31.7922	24.4729	33.2409	32.5615	30.8147	31.7071	33.6856
35.0040	36.2908	36.6418	36.7269	37.1392	36.1792	36.7179	35.8121	36.7101	36.3722	34.4949	36.1502	36.6409
26.0969	24.6575	30.4658	23.8009	23.3342	27.3322	28.5881	21.0013	26.4902	25.4071	25.9546	25.0063	25.0197
26.3827	25.3421	26.4488	24.1209	25.3721	26.4337	26.5047	23.8235	28.0322	26.3437	25.1195	25.8570	25.7867
28.3512	27.6101	30.2962	30.7088	30.5067	30.8521	29.2487	26.6592	32.0050	32.6430	29.2470	30.6058	31.2111
31.6011	28.3351	34.5559	35.0995	34.4992	30.8657	30.3123	30.2988	29.7264	29.2771	26.9095	31.9899	31.5288
31.1678	27.9003	33.9438	35.4308	35.5217	31.2571	30.5466	30.1049	30.3880	29.5157	27.2600	31.9208	31.8722
36.2148	34.3847	38.5478	35.7587	34.3645	36.7109	36.6084	33.7883	37.9523	36.8967	35.8188	38.9376	33.5733
33.3803	31.5275	38.2776	34.7763	35.2742	29.9675	32.6516	26.7628	26.5809	34.1724	25.8914	33.5158	33.1923
34.9727	33.9884	36.2815	38.0369	35.6991	32.7642	33.8758	30.8280	30.6705	38.0234	31.5530	38.1438	34.4364
37.1348	37.1670	37.5490	37.4738	39.0668	39.1464	39.5828	37.5224	37.3282	35.0979	37.8612	36.0977	39.6437
34.0162	35.8395	38.1865	36.2996	36.6735	37.3250	36.7867	36.5377	37.4088	35.8607	35.6766	37.5019	35.3312
32.7056	35.1463	36.3679	35.4788	31.4124	34.0521	35.3255	33.4083	34.8330	33.9497	33.5143	31.6924	34.6401
24.0266	24.4946	25.3567	25.9501	25.6874	25.1349	24.9335	23.4458	26.6856	25.1845	23.8367	24.6935	25.4322
30.4795	31.9688	31.3434	31.4484	32.8721	31.6833	30.6804	29.7758	32.3543	30.9163	31.1019	31.2468	31.4263
19.0520	20.1448	20.7472	19.7402	20.4417	20.0718	19.2075	17.2670	20.9783	19.8046	18.5182	19.9700	20.4500
23.0717	24.8636	25.3832	25.0078	25.8521	24.0873	23.4275	21.7810	25.6542	24.1943	23.0011	24.1637	25.0031
37.5729	39.7597	40.0000	39.3058	40.0000	40.0000	40.0000	32.9512	40.0000	36.5211	40.0000	40.0000	38.6201

Table S3. Median Ct values of plates A and B.

27.8049	27.3187	31.8630	30.6871	28.8030	32.9431	27.8085	29.4261	32.9589	32.3335	29.6561	30.3332	29.3689
24.2338	24.0143	28.2542	25.1418	22.1071	22.6685	23.1886	20.0665	23.4430	24.5781	21.4159	22.8552	24.5151
36.2188	37.6618	36.7118	37.2153	39.1657	35.1346	36.9720	37.0452	38.2277	35.3462	37.6369	36.3856	37.4472
27.3749	27.4313	26.4954	23.8296	31.2745	26.8978	30.9764	29.5088	28.4888	29.1998	27.4703	30.3938	25.7213
33.2234	32.0662	31.8162	33.1818	38.1111	35.9363	35.1930	35.6691	36.4451	35.3280	33.4966	36.4213	32.5707
24.5304	22.9735	26.0241	24.5338	23.1441	25.6476	23.1665	23.6407	24.2257	24.2402	24.0455	23.8658	22.0642
29.6905	30.8080	31.6600	27.6313	32.2508	35.6340	29.5977	31.6407	33.8636	28.6058	33.7401	31.3545	29.8849
27.9845	29.6110	28.6109	31.6680	32.4987	29.3882	28.6681	30.2844	30.4138	29.9652	28.9983	30.7272	28.4373
22.1639	20.1680	23.4132	24.7552	25.1034	22.9583	23.0374	20.2517	23.7036	24.9954	20.9280	23.0780	24.2796
39.3385	38.7896	40.0000	40.0000	39.4523	40.0000	40.0000	38.9920	39.7884	40.0000	38.6887	40.0000	40.0000
35.1790	37.2126	39.1482	34.0818	36.8045	37.7957	35.9781	37.4686	37.7390	38.7825	37.3071	38.5791	37.9354
20.1798	19.3342	20.2719	20.8302	20.8284	21.1081	19.7997	19.7541	21.5130	21.9716	19.3178	20.9890	22.0844
36.9863	38.3720	37.5892	37.9015	32.7760	38.9636	37.5053	35.6004	40.0000	36.0118	36.8407	38.0993	32.9547
36.3383	36.4383	37.1493	37.5586	32.7790	38.5015	36.9660	36.7970	40.0000	34.8164	36.3448	36.5655	32.5132
25.5809	23.0007	25.9059	23.0249	24.9834	26.1394	26.7224	23.2744	27.1960	27.3991	22.0474	27.5435	27.6247
21.4224	21.0201	22.0130	20.6095	20.4542	20.5584	20.8440	17.1769	20.8160	23.0854	19.5662	21.7148	23.3897
23.1798	21.7140	22.6625	20.7527	21.2921	21.3222	21.9144	18.5467	23.0887	23.9855	20.5457	22.6726	23.8362
26.9599	26.2973	27.3525	25.9747	26.4714	25.8265	25.2191	21.2914	25.9360	28.8693	25.5065	27.8371	28.7584
34.5678	32.8075	33.4520	28.9589	35.4483	35.7039	34.1003	27.5625	37.7342	30.8429	34.6539	35.5737	32.6688
34.4408	35.4413	35.7859	35.3850	35.2276	36.1911	36.5674	34.4306	37.0016	38.4285	35.1432	38.3874	37.4152
40.0000	37.5976	38.0776	35.1378	40.0000	40.0000	40.0000	33.0265	40.0000	39.2622	38.7565	40.0000	40.0000
37.9446	37.9834	36.6341	38.4858	39.4055	38.6372	37.2528	38.1301	38.8911	35.8549	37.7034	39.4082	36.5503
28.9081	29.1089	29.3384	30.2306	29.5474	31.3940	29.6857	28.7840	31.8629	30.2842	29.7615	29.9506	30.7030
24.4661	24.0979	24.3362	24.5467	23.9937	25.0106	24.2283	22.6467	25.9809	25.0031	23.3321	25.0375	25.3437
35.0228	33.3310	30.4164	35.3118	36.9839	36.2650	38.5190	34.1082	38.0430	33.5103	36.1306	35.7310	34.9224
37.3585	35.0049	35.7970	37.6484	37.0149	36.9077	36.2370	35.2630	37.3981	38.2490	35.5190	38.5544	38.0751
35.3535	35.3154	34.4453	33.9484	36.0719	33.8290	32.9467	32.5702	35.5273	35.8602	34.2400	35.8020	35.4602
23.1801	21.4845	22.0325	22.0616	23.0643	23.0683	21.7783	20.4098	24.4872	23.1183	21.3067	22.7476	24.0690
31.2358	32.1023	28.4537	29.1416	30.5639	33.5233	33.7220	30.7134	33.9841	32.8489	32.2386	31.4213	31.2544
28.7838	28.8622	28.6966	28.4663	29.1134	28.7038	28.8178	26.4632	29.3094	29.3809	27.6371	30.6652	29.2271
26.6383	25.7408	26.3496	27.4923	27.8620	26.2104	25.9108	23.8989	27.3814	28.8468	26.1939	27.1422	28.8553
24.8861	24.7298	25.5767	26.6414	25.5922	26.0071	25.7186	25.0500	28.0222	26.0032	25.0798	25.9416	26.9843
33.0475	35.6858	37.1082	38.1301	36.1983	36.6502	39.3957	36.2889	38.3265	37.9880	35.6371	39.3584	40.0000
32.7000	30.8771	33.9275	34.8444	30.7602	33.8462	29.9760	31.5444	33.7450	32.4980	33.1804	34.1841	35.8427
26.2248	26.0080	25.9252	26.3552	24.0402	32.4396	25.0950	24.2296	28.0549	26.1674	23.6917	24.0915	25.8939
26.7575	26.4987	26.4554	28.8632	25.2619	26.9823	26.3703	24.8301	28.9805	27.1218	25.1071	25.2912	26.9642
30.7725	30.1687	31.4865	31.3097	29.1707	30.6755	29.1280	28.4351	32.3869	30.8531	29.5007	30.3828	32.2170
31.9252	32.9081	31.1642	30.8625	32.4850	32.2075	33.5430	30.2205	34.7335	32.6510	30.8517	33.0556	32.5417
32.4712	31.7135	31.6499	32.5445	33.3750	32.1654	34.1117	30.7710	35.2623	34.5752	31.5994	34.7390	33.1049
29.1659	29.4647	29.4595	30.5517	30.8653	30.4417	30.0592	28.6060	32.8100	31.0402	29.8680	30.8710	31.1475
38.7934	36.0967	36.4784	40.0000	35.6254	34.3184	37.9099	36.2842	35.5054	35.1665	35.9681	34.5166	34.5421
31.9099	30.8464	29.9839	30.9552	29.5338	31.1861	29.4866	31.0362	31.5784	31.3817	31.0899	32.7310	32.8251
38.2547	38.0539	40.0000	36.8240	36.7306	40.0000	40.0000	40.0000	40.0000	38.2324	37.1103	36.6490	40.0000
25.5826	28.5965	28.6914	28.8654	30.1816	28.9961	28.9791	28.5522	30.9164	30.4863	26.4246	30.9207	29.2522
34.9714	35.9160	37.5738	37.8839	37.6118	40.0000	32.6841	38.0922	38.4020	38.8798	38.1855	36.3664	39.3012
26.6627	26.6397	26.8636	25.9286	26.1289	26.3565	26.7629	23.9580	27.4447	27.9028	25.2883	26.6194	28.3146
33.4295	31.6342	33.0054	35.2277	33.1324	34.0855	34.0020	31.5067	34.2527	34.2570	31.4054	34.5917	34.3813
31.5935	31.6112	32.0298	31.6400	31.2260	32.0337	32.1422	30.2879	32.3262	32.9703	31.2211	33.7445	32.2634
37.6887	37.7269	40.0000	40.0000	36.2300	38.2488	37.9369	38.3449	37.6293	39.5222	37.4237	38.6180	39.2139
35.1843	37.0423	37.5335	30.1703	35.6665	37.3987	36.5473	36.9861	37.5076	37.8763	35.6158	40.0000	35.2513
34.2149	34.8961	34.2467	32.4825	32.8027	35.1965	33.0720	32.8390	34.2376	34.3367	32.7901	35.8559	35.9333
40.0000	38.8554	39.9221	40.0000	40.0000	40.0000	40.0000	38.5667	40.0000	40.0000	40.0000	40.0000	40.0000
22.5565	23.3849	24.0948	25.5669	22.6145	23.8344	22.1077	21.1504	26.2340	24.6570	23.6269	23.0218	25.0481
23.4776	22.6515	24.8876	24.3416	22.6858	23.9778	22.5305	21.3295	26.0007	25.6733	23.2582	24.2580	25.6498
18.2304	19.0751	19.4980	22.8059	20.2273	20.8725	19.8479	18.3553	22.1817	21.0272	19.2725	19.6290	20.5337
38.3500	38.1968	40.0000	37.9831	35.7000	33.9977	36.9252	39.0971	35.9595	39.4576	34.0266	37.6785	33.3850
31.7779	31.4856	29.2762	33.7463	31.9532	32.3528	31.8002	31.4300	32.2184	33.5037	29.9261	32.5373	31.6372
39.1847	37.7756	38.0269	39.6900	33.7411	40.0000	38.6746	36.5052	40.0000	36.4244	38.1507	36.8132	33.7738
26.8967	27.6227	26.4530	27.6436	27.5536	28.0148	26.5100	26.2108	30.2174	27.0291	26.3321	28.3226	27.9477
37.7154	37.4420	40.0000	40.0000	38.0469	38.4205	37.1247	37.7256	40.0000	38.1579	38.7049	37.6611	39.6483
39.5286	38.0598	40.0000	37.1550	40.0000	39.4158	39.6393	39.0914	40.0000	40.0000	40.0000	39.7187	37.5387
37.5317	38.8108	37.4945	38.8567	39.4844	38.0210	36.6031	40.0000	36.9880	37.3706	38.2064	39.0993	40.0000
40.0000	40.0000	40.0000	40.0000	40.0000	40.0000	40.0000	38.3217	39.1362	40.0000	37.4460	40.0000	40.0000
37.7921	39.2713	39.8052	39.1968	37.6100	40.0000	38.5187	39.4467	38.0507	39.0917	37.9767	37.8704	40.0000
38.9539	37.7860	40.0000	36.6123	39.4999	37.0418	38.9995	40.0000	38.3085	40.0000	40.0000	40.0000	40.0000
24.0102	22.6000	23.0177	24.6146	21.4802	23.9463	23.1307	21.5519	24.7398	23.4802	22.5374	23.7615	23.8529
38.2375	38.3189	39.2470	40.0000	39.5367	38.0085	40.0000	37.2992	38.4238	36.6384	37.4234	38.2905	34.8960
27.9244	27.5013	26.7895	27.3409	26.1138	28.2070	26.9792	25.7583	29.4347	27.0000	26.5799	28.0626	27.9715
36.3553	38.9872	37.7642	38.1101	40.0000	38.0676	39.5432	38.4017	38.2846	40.0000	38.7154	39.6018	39.9404
29.8632	29.9732	29.3518	34.2131	30.3909	31.9129	30.3340	30.0596	32.6996	29.9027	30.3941	30.8845	31.3224

Table S3. Median Ct values of plates A and B.

27.8500	27.9754	26.8228	29.6807	26.5415	28.0744	27.4205	26.2188	29.4829	28.4855	27.2858	28.2583	29.1921
33.2769	32.2388	33.2874	33.2587	31.6628	33.7177	33.1288	31.5073	35.0294	33.4582	32.1141	34.6640	34.3458
27.8143	26.1623	27.8215	26.2049	26.1382	27.4206	27.6335	25.3647	29.0158	27.3717	26.0143	27.0234	27.9891
27.8190	27.0261	28.3362	29.3511	25.7006	26.0399	27.1619	22.5790	29.7028	29.0224	26.1257	26.7798	29.3575
37.1013	40.0000	39.1605	40.0000	40.0000	40.0000	34.7815	38.4152	39.2666	40.0000	40.0000	40.0000	38.0197
29.5476	29.7627	30.5383	34.1126	31.5205	33.9385	33.0536	32.1382	36.0927	31.5280	32.0738	31.9975	30.7284
25.4156	24.2506	25.8431	27.9821	26.8548	26.2467	26.5093	25.9738	28.2364	26.3985	24.8808	27.0955	26.9302
28.8173	28.8335	28.9678	29.6692	28.0831	28.5024	29.5378	26.4099	30.5337	28.9850	27.5403	29.1796	29.8858
25.0212	24.6438	25.8486	25.3426	23.7138	25.6690	25.3551	22.6112	26.5364	25.0830	24.9075	25.8730	24.3500
39.0825	38.1034	40.0000	38.0472	38.5868	39.6930	40.0000	36.7265	40.0000	40.0000	37.3496	40.0000	40.0000
38.4024	37.3815	36.4815	36.4973	33.1257	40.0000	40.0000	36.9228	38.2660	36.9588	36.8474	38.2384	34.8613
34.9940	34.6309	36.2856	39.1056	33.0940	38.2507	37.1193	36.4782	38.0036	36.3514	36.4609	38.6826	32.9807
38.6039	38.9068	33.0250	33.0779	32.1950	40.0000	32.9285	39.6634	40.0000	39.3061	39.4363	40.0000	40.0000
37.2076	40.0000	38.2204	40.0000	38.0853	40.0000	39.2150	40.0000	38.5540	40.0000	39.1146	39.3714	40.0000
31.0131	35.5380	33.0262	32.2418	30.6182	33.9177	32.1276	36.4552	34.1621	35.7947	33.0752	33.3794	36.5661
33.2801	38.2304	38.8250	35.4360	33.5132	36.1787	36.0083	38.9550	36.9357	36.2168	35.1746	37.6979	36.6952
26.9665	26.6831	27.9050	28.3307	27.1090	26.8463	26.7799	25.3188	28.0897	28.5068	25.4730	27.7396	28.5248
21.4179	20.1666	22.1295	23.5216	22.7212	22.9723	22.1755	20.4218	23.1894	25.3593	21.3143	23.7506	24.9084
20.5734	19.5553	21.5170	28.2957	23.5020	22.4186	21.7440	21.9738	22.6905	24.8604	20.2063	23.3657	23.8416
25.3184	20.2391	25.6885	25.5475	25.3155	23.2325	24.5271	20.9623	27.0256	24.5009	21.4255	23.4526	24.1469
32.4404	29.4463	31.2996	31.2019	30.9513	31.8091	32.4587	29.4992	31.5510	31.8398	28.5539	31.2206	30.3236
23.0560	22.4963	23.4139	22.9473	23.7278	23.6731	22.6822	21.0038	24.6174	25.2087	22.1576	24.1410	24.3374
25.2710	24.1554	25.2061	25.7428	25.6890	26.2068	25.2268	23.7093	27.4524	27.0670	24.7475	26.4114	26.6654
40.0000	36.5515	37.6220	40.0000	36.8847	38.0179	38.3222	38.4457	40.0000	40.0000	40.0000	40.0000	38.2457

Table S3. Median Ct values of plates A and B.

76	77	78	79	80	81	82	83	84	85	86	87	88
35.3697	32.1680	34.9835	31.9972	35.5575	33.9659	37.7465	23.4070	33.5931	35.1560	32.6438	25.6900	31.0348
38.6435	36.0368	36.9471	34.3562	37.1442	38.5622	38.7326	28.2435	35.7410	37.8223	36.5288	29.7864	35.6938
34.8852	34.8946	34.8906	23.6348	31.4250	30.3856	38.5161	24.0772	35.6118	31.2571	30.9374	35.9873	34.8720
26.2913	25.0091	27.3231	26.1420	25.3123	26.6164	29.0758	23.1890	22.4846	24.9124	23.1317	24.9866	24.5977
23.9824	23.1700	24.7464	24.1569	24.4309	24.4491	28.5331	20.8006	22.4622	24.6256	22.6162	23.3780	22.4847
22.9516	21.2646	22.8294	22.4473	21.9316	21.3363	25.0284	18.8271	19.8843	21.4120	21.1674	21.0661	21.7334
29.3323	27.3713	30.0986	28.0776	28.9822	27.1734	31.3927	24.9630	26.1950	27.9815	27.3726	26.3733	26.3380
24.2888	23.1078	27.0776	23.9922	23.3922	21.6841	26.7811	20.3990	22.1488	23.2958	22.2016	21.9295	21.3085
23.7926	22.6025	27.1188	22.2720	23.8572	21.3771	26.4584	19.3722	21.1885	26.6304	21.5401	20.6760	20.8928
24.4791	23.8138	25.2723	22.9433	27.4961	23.7173	27.1952	20.9276	21.0503	24.2629	23.8364	23.7231	23.2379
23.0545	21.3389	23.3295	22.9977	24.6410	24.6948	25.7254	19.4294	19.7485	20.8633	20.0166	22.9917	21.8892
23.1792	23.1781	27.7981	23.5835	24.3992	25.0981	27.7011	23.0475	22.0099	23.7577	21.9871	24.6280	21.9418
28.3477	28.1142	29.2735	28.4231	29.0698	27.7175	33.4241	25.7509	26.7982	27.6957	27.6231	28.5623	26.9576
24.3835	23.9814	24.4802	24.1929	25.4985	24.4067	27.3596	21.3347	22.4895	25.0700	23.2126	24.1908	23.1309
23.3915	23.2176	24.7509	23.4560	22.2560	21.6710	26.3915	19.8049	20.6326	22.5531	22.3147	24.7134	22.9568
25.1457	24.6582	26.1925	24.4987	24.2790	23.9942	27.4079	21.8827	22.2325	24.4912	24.6335	24.5309	25.2625
24.4320	24.2485	33.1528	24.3965	23.6525	24.0611	29.7367	24.2133	23.3033	24.1375	22.9443	23.4803	24.0984
27.6573	27.3958	32.7952	27.5398	27.9519	26.6266	30.6904	24.8710	24.8418	27.3138	26.3335	27.1090	26.9510
28.7381	27.7793	29.0882	28.2229	32.1358	30.0490	32.7352	26.4494	26.5121	29.8074	27.2401	28.4588	27.0967
25.0558	23.6023	26.6592	22.9188	24.8337	25.5166	26.5833	21.4218	22.4700	22.6768	23.5339	25.5861	24.0458
27.0966	24.8409	27.3503	24.3089	26.1160	27.4101	28.1547	23.1919	23.5142	24.5421	24.8631	27.9459	25.9421
23.9091	22.2997	27.2597	22.4780	23.4275	24.5830	26.6571	20.0342	21.3777	22.3715	22.2769	24.4849	23.3660
27.7276	27.2724	33.9786	28.4514	29.6944	29.9845	32.6867	25.8985	27.5687	28.5182	26.9316	29.5107	27.4300
22.8184	22.1445	24.0251	22.6212	23.6785	23.1323	26.3341	19.3799	21.1588	22.7313	22.0295	23.9102	23.2336
23.7260	22.9274	24.2015	23.4901	23.9977	23.9349	28.0872	20.9924	21.9243	23.8763	22.9871	24.2943	23.5615
24.4091	24.7440	27.3930	24.9087	26.0627	24.7752	28.6550	21.3908	23.1034	25.6201	24.3288	25.8435	25.2961
26.7113	26.3028	31.3687	25.5172	25.7120	25.2436	28.7646	22.8717	24.0711	25.5035	25.1429	26.6631	27.2983
38.0488	36.5025	36.5058	36.3972	36.2553	36.2643	40.0000	38.2441	32.7408	40.0000	32.7106	40.0000	34.8693
27.7174	28.0276	33.3978	30.4774	27.6893	27.9982	33.1314	23.2653	24.9019	30.5514	26.3541	25.9155	24.6773
38.3250	38.6146	40.0000	36.5200	39.3767	39.0192	37.1259	38.1915	35.8210	36.7031	37.0101	37.7793	33.9288
37.5718	39.7049	40.0000	38.1125	37.8711	40.0000	40.0000	36.1691	36.3245	36.7544	37.4035	37.3230	33.7687
22.2364	21.2386	24.1597	21.7409	23.0270	19.9612	26.1708	18.0853	19.6356	21.3768	20.9555	22.4126	19.7802
36.8427	33.5416	35.9310	35.8998	34.9558	35.1539	38.4559	32.4099	32.2435	38.6567	35.0365	37.2363	36.3400
38.2606	37.1385	38.5641	36.5681	36.3489	39.9361	40.0000	35.5576	38.0018	37.0903	39.0751	38.2815	40.0000
28.6091	28.3008	30.2952	28.7426	27.8877	27.7392	32.5006	25.6617	25.1100	28.0205	26.5356	28.9431	28.1725
27.2649	26.6384	28.3884	27.0857	21.8522	25.9550	31.8255	24.0133	28.9981	22.5973	25.8759	35.6144	31.0165
27.1854	27.1251	30.9500	27.8344	21.7656	24.9481	31.0359	24.7679	27.7147	21.1252	26.0575	34.6723	31.0721
24.4077	23.3479	25.6369	24.9588	23.4388	23.1824	28.0762	22.5864	22.2714	23.9987	23.0641	23.7825	26.8366
36.6385	35.3412	40.0000	37.2776	37.8266	35.6805	40.0000	34.7088	35.7315	36.6266	36.3860	37.7899	37.1479
38.4762	37.9962	38.8104	38.1730	38.4923	38.1684	40.0000	36.7846	36.9784	37.4860	36.6101	37.4933	38.0859
24.4786	24.0011	29.5050	24.3962	24.7999	22.0645	28.2972	21.4838	22.2927	24.1961	22.8918	21.7992	21.9370
29.6533	29.5326	33.9908	31.3326	30.2707	29.2066	35.2965	28.7648	28.8257	31.1425	28.7252	29.4474	30.0354
40.0000	40.0000	37.7925	40.0000	37.8651	40.0000	40.0000	40.0000	37.1011	40.0000	37.6942	38.1581	40.0000
37.3199	36.3340	40.0000	35.8349	35.5514	35.9980	40.0000	33.2410	33.7724	35.8087	33.8126	34.8798	35.9328
31.3681	30.6925	40.0000	31.5688	27.7482	29.9181	33.7829	27.0967	27.2853	31.2806	27.3515	29.1069	29.5424
27.2702	26.0773	30.7722	27.5239	26.1564	23.2150	29.3566	27.3130	22.6573	25.8238	25.6864	30.3033	24.6587
27.8664	26.8002	37.1223	27.5347	21.9679	25.1935	40.0000	24.4963	28.1206	22.4280	26.5033	40.0000	30.7566
24.4202	24.9363	24.0038	27.0996	27.8088	21.8120	26.3009	22.6830	20.2785	29.1217	22.5912	33.2426	29.2328
36.2913	38.3953	40.0000	31.7177	28.9208	40.0000	40.0000	35.5688	33.2740	27.5764	36.4189	40.0000	37.6794
30.9877	31.8663	36.2432	32.9166	32.0926	32.0824	32.3472	29.2527	26.4458	30.4401	27.9857	33.6079	29.8093
32.5289	32.9466	34.0826	33.8745	32.5388	33.7260	33.9755	30.3441	27.3236	30.3802	28.5169	34.2072	31.0869
35.3093	36.4068	40.0000	36.3519	37.0565	36.4644	39.7843	33.9851	35.4505	34.5458	34.1614	35.8842	35.9552
24.4637	24.8361	25.8271	24.4103	29.2076	23.6006	26.9535	23.0853	21.4808	25.4161	23.2463	31.9295	26.5461
26.2430	24.8966	27.9736	26.9427	26.9021	26.4550	29.0259	24.3023	24.1667	25.7397	25.5283	26.3477	24.7780
30.3485	30.3956	31.5518	28.6591	31.2389	29.9481	32.1008	28.5964	28.4026	30.7368	29.6052	27.9784	29.4825
33.2701	34.4034	34.4545	32.4814	36.7674	33.2778	40.0000	25.6891	34.1417	33.9970	29.9535	36.3994	33.4831
33.1248	34.6625	34.4246	32.9605	35.3071	32.9948	40.0000	25.7397	32.2049	33.7681	29.6611	33.3668	33.6425
37.9335	36.8956	37.5323	33.9811	31.1282	38.6070	40.0000	35.2694	35.5800	30.9626	36.1938	35.9804	36.8816
33.3829	32.6167	36.1124	32.9658	36.9105	30.6009	35.9115	30.9363	30.5956	37.2410	30.0883	37.7425	36.2937
36.2040	35.6634	37.2430	36.9927	36.1533	34.4714	37.1084	29.6029	33.5164	37.5810	33.9432	37.3952	36.1521
38.2016	38.3157	40.0000	39.8484	37.7148	38.3394	40.0000	38.1440	40.0000	40.0000	37.1823	40.0000	38.6881
37.3793	38.2962	38.2332	37.4679	37.3066	38.5040	38.7729	36.9740	36.3984	36.8124	37.4671	37.7856	37.1292
33.3094	33.5748	40.0000	33.2039	33.9283	33.9933	34.0067	33.3844	32.3484	35.1600	32.7719	34.5124	34.8326
25.8477	24.8058	26.9580	25.6687	26.1677	25.8128	28.9939	23.3022	24.4536	25.9566	25.2111	25.7233	25.1529
32.9122	32.5193	34.2640	32.0685	31.8269	33.0606	37.3954	30.6685	31.3213	31.4591	30.3807	31.0068	30.6954
21.0413	20.8672	22.3419	19.9788	19.6823	20.4044	23.8825	17.8360	18.4750	19.5595	19.1354	19.8599	20.7662
25.0566	25.3310	29.8237	24.5093	23.9893	25.4964	29.8563	22.0301	22.6886	24.0824	23.3685	24.4565	25.0036
40.0000	40.0000	39.2607	37.3248	36.2846	40.0000	39.1155	34.7614	40.0000	28.0262	37.7979	40.0000	39.7672

Table S3. Median Ct values of plates A and B.

28.1363	31.3282	32.0592	30.0311	26.4670	29.7190	35.9873	27.9546	27.1452	28.3501	29.3362	29.0498	24.8088
23.5018	23.8032	23.5319	24.1148	24.1253	22.8822	24.4684	21.0188	19.7148	22.4084	22.0835	30.0188	26.0700
32.5084	37.9919	39.2335	35.7927	39.2123	38.5728	40.0000	36.9449	36.6178	37.6944	37.1419	37.8256	38.6956
30.6621	30.4997	31.1746	29.1968	28.6603	32.9385	35.1794	26.9796	27.7016	27.0463	29.4534	29.8474	26.2607
33.8567	32.2493	36.0219	34.7044	35.2012	37.8748	40.0000	31.8498	32.5907	33.0979	33.3982	32.0863	30.8554
25.3217	22.9253	24.5772	25.0753	24.5912	23.1248	26.0676	23.8752	21.9566	25.9684	23.3070	27.3850	23.8843
32.5525	27.0853	32.5357	31.0801	32.8790	28.1034	35.7259	27.6949	31.4706	35.8050	31.4262	34.6111	32.7321
32.5185	29.4360	31.9943	31.3870	29.4727	31.8576	35.3972	29.1750	29.8198	31.9739	32.4903	30.8082	28.8370
24.4262	23.7044	29.3350	23.6798	22.1459	22.2024	28.2909	19.9792	20.8762	22.9425	22.3011	22.6903	22.4167
38.8759	40.0000	40.0000	40.0000	37.3402	38.4648	40.0000	38.9983	38.8146	36.9755	37.5658	39.1630	40.0000
36.2561	38.5267	38.0932	37.0662	39.3223	38.2907	40.0000	36.5976	37.4381	37.0519	37.6008	36.6171	39.3033
23.3573	21.2089	22.6786	22.3959	21.4151	21.0155	23.2005	18.8191	20.2202	21.9733	21.2202	20.8848	21.4273
38.0890	36.9283	40.0000	32.9298	28.2859	38.1994	40.0000	38.0581	36.8677	27.9474	37.4954	37.7341	40.0000
38.4019	37.5482	37.2399	33.2917	30.6208	35.7930	40.0000	37.1916	35.6302	28.4695	37.6414	39.1873	37.7678
28.5463	26.4201	26.7575	26.1722	28.8609	27.7821	28.2674	22.6625	24.1267	29.3800	26.6324	27.2995	26.3967
22.6246	21.9925	26.0300	22.4971	20.4750	22.2210	22.4232	19.3513	19.2376	20.3712	20.5946	25.4434	23.2304
23.9156	22.8166	23.6998	23.4826	22.4814	23.3294	23.4066	20.4415	20.2559	21.3344	21.8449	25.6890	23.9143
28.6969	27.2852	31.5795	27.6228	25.8979	26.7752	28.2053	24.4251	24.4200	26.4026	25.2335	30.9987	28.8274
36.2626	34.5067	34.7620	35.4279	34.0130	37.7498	40.0000	32.2643	34.4348	34.8503	35.7290	33.6317	35.7625
38.6067	37.4240	40.0000	37.3076	35.5155	37.4411	39.0851	35.2748	37.1433	38.4554	35.6560	33.6120	36.3698
40.0000	40.0000	39.9371	40.0000	39.3867	40.0000	40.0000	38.5516	40.0000	40.0000	40.0000	39.7656	40.0000
38.3665	38.9325	40.0000	36.8366	38.5150	39.0949	40.0000	37.2375	35.4566	35.1442	35.4176	38.3717	37.5300
31.6172	29.8039	33.5710	31.5104	31.5057	30.9778	32.2659	28.9274	29.5372	31.0636	29.0366	30.4646	30.0504
26.1996	24.5041	26.2936	25.1498	25.9526	25.3348	26.8826	23.1013	23.7309	25.2230	24.8413	25.4424	24.0979
38.4587	36.9281	35.3137	39.5138	40.0000	40.0000	39.2596	34.7308	36.2825	39.4509	35.3814	35.6652	35.4279
38.5246	37.9301	37.7354	36.6705	36.9658	37.3349	36.5978	35.4756	35.4870	37.6490	37.4812	38.3509	36.9709
37.4177	38.3191	39.3800	33.5785	37.6350	38.7263	37.1176	30.4103	33.6168	35.7419	35.4430	35.0046	34.4783
23.1456	23.2191	22.9581	23.9633	23.0238	23.8290	24.3511	20.9913	20.9682	22.9404	22.8634	21.4982	22.0360
31.1227	31.8115	33.8376	34.4754	29.0767	32.6629	33.8390	30.5039	29.7166	30.0210	30.2272	30.8098	29.7331
31.4578	29.3467	32.4502	30.2097	29.0870	29.1862	29.3482	27.1057	27.6146	30.8147	29.0981	27.7680	34.6624
28.9550	27.3370	29.2181	27.4498	24.2256	27.6415	29.8036	24.7124	26.2033	28.1297	26.4474	27.0038	27.4954
26.6639	25.4503	26.3342	27.9420	26.2310	26.6078	28.2262	24.6386	25.0740	26.7593	26.0591	25.1265	25.3128
37.2485	38.7772	40.0000	37.7412	39.4745	37.9485	37.7829	39.0643	36.5195	38.9758	38.9308	37.8907	38.5667
34.9274	34.6691	35.5550	30.1748	32.8432	32.8594	33.1024	30.0351	33.5084	35.4899	32.7645	36.5188	34.0150
24.6990	26.0563	25.5393	40.0000	25.9123	28.6953	27.0396	40.0000	23.7604	24.7355	24.0756	27.1376	24.6893
25.6102	28.2954	28.1631	26.7829	27.2183	29.7152	29.7711	24.3877	24.4425	25.6067	25.4272	27.9960	25.5225
30.7744	31.1468	36.1214	29.5428	28.3815	31.8059	32.4611	28.2404	28.5132	30.8695	29.2916	33.0831	31.2197
34.2330	33.0829	34.7184	33.5569	33.8969	33.5709	34.3580	31.4055	32.2073	33.5004	33.0283	33.2054	30.8264
34.0598	33.0091	34.1609	33.7970	34.3670	33.4313	34.4480	31.0931	32.9894	34.6155	34.1208	33.0122	31.7919
31.8213	30.1090	31.6992	31.4400	30.9968	31.8665	33.2516	29.1608	29.9593	30.8293	30.5398	29.5778	28.8812
37.1036	36.6489	40.0000	35.5117	34.2553	37.5568	32.5870	34.1526	34.9322	33.1132	29.0780	36.6840	37.6819
34.6685	33.6847	31.1795	29.6652	36.3407	33.7506	29.8620	37.8175	33.4307	34.3404	32.1008	31.4900	35.4248
38.7627	38.4930	40.0000	40.0000	40.0000	38.1300	40.0000	37.5770	40.0000	35.5931	37.3066	39.9857	38.1758
32.4706	29.6640	31.0224	32.4894	32.1161	32.3266	29.7457	27.6514	28.9724	29.1078	29.8192	30.3798	28.9617
37.7466	37.7068	40.0000	40.0000	38.4056	40.0000	39.5649	36.3117	38.2813	38.6291	37.0290	37.2277	36.1688
28.2435	27.1285	28.8317	27.3672	27.0480	26.7858	31.0017	24.2571	24.9858	25.6214	25.6171	31.6483	27.7006
37.0795	33.1927	35.7614	33.1784	30.9671	35.2744	35.1625	29.9447	32.6024	36.0840	31.6360	35.6695	31.8650
35.3214	33.3276	37.4862	34.1325	33.1811	33.2016	34.0834	30.7109	30.4934	35.0510	32.3919	33.4677	31.0246
39.1923	39.1378	40.0000	38.4790	36.1948	39.3322	40.0000	36.8695	38.6155	38.2286	38.3709	39.4815	39.3644
37.5038	38.1791	36.8856	36.1942	32.1390	37.8800	36.7383	31.4774	36.3939	35.8946	35.9885	36.0081	38.9817
35.2349	34.7582	37.3855	34.8520	33.9495	33.9221	35.3867	31.4146	32.9647	34.4893	33.3734	35.0785	36.7111
40.0000	40.0000	40.0000	40.0000	40.0000	40.0000	40.0000	40.0000	40.0000	40.0000	40.0000	39.1130	40.0000
24.5042	23.9708	25.9440	23.6182	22.7384	23.2513	27.2502	22.3000	22.5040	21.9088	23.2622	21.2451	22.1461
24.6912	23.4474	25.1538	23.9669	23.0822	22.6020	26.2646	21.8063	21.9590	22.3440	22.8413	21.3304	22.6719
21.8896	20.4922	20.5210	20.9510	22.3777	21.5162	23.1124	19.0973	19.2870	22.5164	20.4895	18.9916	19.6450
35.9405	37.8168	40.0000	34.4140	25.0004	30.6281	40.0000	35.5278	39.2457	25.7708	32.3965	36.4716	38.2981
33.0899	31.8312	33.3730	32.2217	32.7878	33.5786	34.8401	29.2001	29.5294	33.6783	32.3389	27.4227	31.6896
39.0885	40.0000	37.2115	34.3824	30.7272	39.5055	40.0000	38.1059	37.0548	29.5350	37.1272	37.5970	39.4618
29.8505	27.1667	32.0926	29.0652	28.1072	28.2244	32.2406	27.1440	26.6601	27.5426	28.2035	27.5218	27.9122
37.7009	39.0215	40.0000	39.3878	38.3350	39.9299	40.0000	37.5723	37.3308	40.0000	38.5773	37.9446	40.0000
40.0000	40.0000	40.0000	39.5521	40.0000	40.0000	40.0000	36.8185	37.1562	40.0000	40.0000	39.3666	40.0000
39.3956	39.1056	40.0000	39.3888	39.1843	37.3734	40.0000	40.0000	40.0000	40.0000	37.7704	39.3487	39.0865
40.0000	40.0000	40.0000	37.9362	40.0000	40.0000	40.0000	40.0000	40.0000	40.0000	40.0000	38.3899	40.0000
40.0000	40.0000	40.0000	37.3428	37.7264	39.3075	40.0000	39.5757	37.4210	39.1612	39.1912	38.3464	38.6705
37.0899	38.4986	40.0000	37.4024	39.0361	38.7216	24.1542	37.1929	40.0000	40.0000	39.5791	39.7938	39.4535
23.6709	23.6321	27.3974	24.5345	23.8064	22.7998	40.0000	20.9432	21.3078	23.8899	22.4665	23.8101	22.5825
37.2046	39.5993	37.3219	34.4557	32.3974	40.0000	37.5280	36.8655	36.6965	30.1433	38.1358	40.0000	39.5951
28.9453	26.8316	28.8733	28.6083	27.8408	28.4413	29.0746	26.2219	26.3822	27.3100	27.8440	28.5776	26.6064
40.0000	38.2401	40.0000	37.2750	37.8603	38.6047	27.8045	35.1945	39.1454	39.2723	37.4324	39.0580	38.0634
32.4320	30.8112	35.8456	32.1552	32.3782	31.7319	33.8752	29.7595	30.9534	32.3105	30.4817	31.7036	30.9561

Table S3. Median Ct values of plates A and B.

28.7116	27.9972	29.7452	27.8268	28.0164	28.6065	29.2300	25.8303	26.5257	28.3384	28.5168	26.8841	26.9742
34.9846	33.6033	35.3987	33.8784	33.6764	33.6897	34.3393	31.8134	31.9738	34.5231	34.2617	33.0682	33.7460
28.1335	27.2174	30.0315	28.2069	28.3034	27.6785	29.7400	26.3711	25.9503	28.2523	27.3677	27.7431	27.1141
27.5343	28.1958	28.5310	25.4055	26.8725	25.3078	28.5895	24.5920	25.0276	27.8061	24.7627	27.2421	28.9243
40.0000	40.0000	40.0000	40.0000	36.2080	40.0000	40.0000	40.0000	40.0000	34.5233	39.8050	38.8783	39.0629
33.2109	30.3847	37.1114	31.5825	29.5070	30.0174	35.0885	31.5179	30.9249	35.3823	31.1376	30.5490	30.6342
28.4860	26.0069	29.3905	29.1055	29.0847	28.0448	30.1614	24.9698	27.8314	28.5701	28.3661	25.8566	25.4639
30.3684	28.2993	29.7997	29.9801	29.4153	30.4754	30.9356	27.8943	27.1966	29.8970	28.2473	30.2364	29.5528
26.1170	24.2611	26.2927	25.9297	25.9884	24.9306	27.0170	23.3394	23.6547	25.6778	25.1518	25.1098	24.7558
40.0000	40.0000	40.0000	37.7710	40.0000	39.9461	40.0000	40.0000	40.0000	40.0000	39.3129	39.2126	40.0000
38.1979	39.0650	40.0000	33.1864	28.0913	37.7216	40.0000	38.1846	38.2318	28.8900	36.4243	40.0000	40.0000
38.8911	37.4025	36.9583	34.6257	31.7340	39.4907	40.0000	34.3403	35.4977	29.7863	35.5080	36.2306	37.4275
40.0000	40.0000	40.0000	38.2615	39.0003	38.4685	31.2392	38.6432	40.0000	40.0000	38.6855	32.6397	38.5812
40.0000	38.7265	40.0000	40.0000	40.0000	38.0152	40.0000	38.4231	38.4410	40.0000	38.4614	39.5768	39.5763
34.1887	34.6652	38.2098	33.7248	32.9397	33.5173	34.8922	32.1899	36.1918	34.6948	32.4298	33.4350	32.9714
38.1997	37.3766	40.0000	37.0423	36.7684	35.7731	36.1727	34.3844	37.7683	36.2842	36.3761	35.7913	36.7559
28.8204	27.3524	28.8792	27.8511	28.4531	27.4472	30.3831	25.4367	25.1047	27.8982	26.4989	29.0437	27.4351
25.7842	23.8098	29.7830	23.4838	22.7887	22.4562	26.2312	19.8833	20.8452	23.7558	22.6376	23.6579	21.9945
26.2676	23.3179	28.8544	22.7922	23.4736	24.6754	26.7332	19.6370	21.5763	24.9089	23.6173	24.2497	21.6175
25.5447	23.5944	27.1093	23.2820	25.3012	26.8098	27.7637	19.9099	20.8781	23.8880	24.8463	26.0852	24.9856
33.0964	30.9994	37.1372	31.9143	30.8473	30.4531	33.1929	29.8608	27.5925	32.0152	29.9065	36.1331	29.3219
26.0822	23.6980	26.1913	24.7397	23.2378	23.5231	26.3676	21.6773	21.7027	24.7380	23.3394	23.8515	23.7584
28.8266	26.7480	28.3269	27.0451	25.8877	26.9207	28.4629	23.7967	23.8468	26.5372	25.5934	26.7896	26.1317
40.0000	39.2591	40.0000	38.9080	38.7374	40.0000	40.0000	38.1604	38.6383	40.0000	37.7353	39.1363	37.3929

Table S3. Median Ct values of plates A and B.

89	90	91	92	93	94	95	96	97	98	99	100	NBM4
30.7473	32.5505	31.0601	35.7191	32.7904	33.9290	31.5577	34.4492	26.9790	31.5409	28.3775	26.7895	32.6432
34.2600	37.8275	33.5306	38.6621	36.0412	36.4603	35.1718	36.8002	31.4155	34.5035	32.1727	31.0525	10.9616
24.4418	24.2648	25.4983	33.2697	31.3078	33.7080	32.1320	32.3596	22.7018	37.1019	23.9787	24.0042	29.0072
24.8270	25.9624	22.8051	25.6544	24.9773	27.4171	23.9874	26.0705	23.7247	25.9743	24.1237	25.3200	26.2187
23.6795	24.1669	21.2815	24.8009	24.0811	25.4552	22.3849	24.8269	22.7963	24.7373	22.9650	23.8244	20.4997
21.4006	22.2891	19.3947	23.0779	21.5618	24.0231	20.7060	22.0554	20.6997	22.8121	20.5974	21.6762	23.2045
27.6820	29.5876	26.5932	29.3871	28.6775	31.3205	27.6677	28.7697	27.5354	26.8683	27.8571	28.7959	29.2193
22.3530	25.3461	21.9923	23.7061	24.4240	25.9640	22.5440	24.5090	22.8810	22.9139	22.9068	22.8578	22.8102
22.3467	26.4388	22.1186	26.0743	22.2011	24.1678	20.9632	22.5418	21.8385	20.9015	21.9423	23.9570	21.3877
24.5354	27.2828	21.9380	26.7225	24.2879	26.4007	22.3604	25.1178	23.0205	22.2856	23.4789	24.8897	23.8693
22.6811	22.7110	21.6666	21.9938	19.7986	25.5108	22.0407	22.2733	18.8793	22.0467	19.8383	23.8890	25.0000
22.9014	24.6590	22.3794	24.1606	23.8392	25.0864	21.9294	25.6550	21.8712	22.9656	22.2799	27.4289	24.6810
27.4926	29.2668	26.6752	28.6107	28.3369	29.7599	26.7768	28.4899	26.8415	28.7801	27.3648	29.9346	27.0000
24.2670	26.4169	22.7233	24.1364	24.0326	25.1685	22.8787	24.2725	24.3431	24.3923	23.9796	25.7382	25.4642
22.5893	24.2253	20.7148	24.3052	22.4015	26.7304	21.6321	22.9660	22.6401	22.7977	22.2544	25.0234	14.6284
24.1764	26.1092	22.2504	26.1309	23.7989	26.9760	23.0989	25.0908	23.6532	24.3874	23.6231	25.5991	20.7256
23.2722	25.6047	24.3280	24.0783	24.5777	26.9073	22.4014	26.0008	23.2449	23.6579	23.8425	25.0666	31.7630
27.8379	29.1228	26.1313	30.2948	27.4701	28.9833	26.4317	28.2832	26.6007	27.2441	27.1556	27.7892	30.5397
28.9611	28.2161	26.4903	29.9477	28.6976	30.0585	27.0730	29.2819	26.4887	28.9202	26.8619	28.9170	30.0949
24.5491	25.3318	22.8301	25.5742	22.4494	26.9019	24.0128	23.3702	22.7764	24.0826	24.2531	23.7939	25.0000
25.9145	27.2200	22.9417	29.9797	23.8608	27.3154	25.5403	25.0556	24.0375	25.2046	25.0978	26.7249	26.0792
22.8951	24.3063	21.3618	24.9857	21.8607	26.0624	21.8497	22.6216	21.9761	22.8375	22.5319	23.8999	22.0000
27.9917	29.3184	26.9516	29.0392	28.0818	30.1502	26.6295	28.9795	27.1220	28.5770	27.4134	30.9787	26.4505
22.5596	23.7851	21.8450	23.4681	22.5624	25.5625	20.9994	22.8876	22.3791	22.4362	22.5238	24.0989	26.0896
23.9117	24.5679	21.6969	25.9754	23.8457	26.0947	22.2465	23.6950	22.8594	23.6283	23.2797	25.9302	23.8536
24.4991	25.8273	23.6259	26.2699	25.6634	27.4810	23.4691	25.9831	24.5831	25.0860	24.7829	25.4529	28.9290
26.0521	28.6113	25.4702	26.6564	25.1159	28.9226	25.0576	26.0523	26.7992	24.3735	25.8866	29.0396	26.3974
37.3058	40.0000	35.2129	38.2777	32.7036	40.0000	39.6914	40.0000	36.1742	40.0000	38.5164	37.4542	32.4973
29.7199	40.0000	26.4627	28.1470	28.7130	33.4358	26.1795	26.6623	23.5087	29.2905	25.7553	25.9116	31.9144
40.0000	40.0000	37.5126	38.1961	40.0000	36.5115	39.3131	34.6209	37.2707	35.2709	33.7227	39.8428	33.3523
40.0000	40.0000	37.6388	40.0000	40.0000	36.4634	39.0496	35.1469	38.1031	34.6043	34.3596	40.0000	21.5386
21.5374	22.9499	19.8889	25.8404	22.3688	24.9238	20.9463	22.2124	21.6216	20.8541	21.8852	22.7408	25.5662
34.8362	37.7962	32.8962	38.3186	33.6397	35.9049	35.9250	34.9714	32.8510	37.1262	35.3794	36.0677	35.0000
39.1263	38.8389	35.8525	39.1949	36.5591	36.6067	38.5709	36.8205	37.2196	38.2481	38.2376	39.6705	40.0000
26.2405	30.0063	26.3595	29.4183	27.4996	29.9758	26.7206	28.6074	26.6604	28.5963	27.3201	30.9736	30.0000
26.9703	32.8003	25.9713	28.8485	21.9765	31.2193	23.0419	23.3358	30.1767	29.2285	29.3568	29.0245	31.6939
26.7700	33.3153	26.4155	29.0241	21.3587	30.9868	22.1600	22.7411	28.6136	29.1047	28.6380	28.0851	31.7794
23.3517	24.9756	21.9418	24.9630	23.9661	26.5895	23.0979	24.7442	22.0009	25.1307	23.1824	24.6090	20.0000
36.3997	40.0000	40.0000	40.0000	37.0586	37.7153	36.2267	37.3273	34.0707	37.4778	36.7716	39.6047	33.0000
38.1456	40.0000	37.6868	40.0000	37.2018	37.5475	36.3814	38.8820	37.0964	36.5234	37.7076	40.0000	35.0000
23.1698	26.4147	22.6160	26.0348	24.8267	26.5104	23.1518	25.3578	23.2014	23.4540	23.9112	26.0946	25.3537
28.6480	31.2525	28.3698	31.2417	31.1548	32.0521	28.5670	31.8245	28.5564	30.6977	29.7697	33.4595	31.7296
40.0000	38.5191	36.9753	38.5752	39.4570	40.0000	40.0000	40.0000	40.0000	36.8427	40.0000	37.3424	18.7375
35.9240	36.9328	33.2201	36.9575	36.0879	37.0201	35.8430	38.0706	29.2995	37.1512	35.8948	36.9462	34.0000
28.0409	30.6007	26.6789	31.0382	28.9148	29.8283	31.2411	32.7791	29.2219	29.6598	29.3998	29.5793	30.7006
29.5149	30.0232	23.0985	29.3609	25.7301	31.5008	24.6515	25.8233	27.4209	24.7932	29.2635	30.4910	26.3498
27.2918	34.0971	25.2927	36.0963	33.4112	32.0102	23.3757	32.9077	29.8523	28.6938	28.9625	40.0000	31.0000
25.7703	29.3595	23.4276	26.0986	27.8629	28.8448	33.3514	27.0775	28.2224	25.6451	27.3597	27.5203	28.8051
39.0421	38.0298	29.4039	40.0000	33.0450	32.7910	37.8761	34.0504	37.1094	37.8108	37.8390	40.0000	32.7144
30.9907	32.8579	29.4938	33.0579	28.1913	32.9952	30.2003	29.8232	30.3759	31.6953	30.5986	32.8526	31.9858
33.1320	34.3030	31.7916	35.1888	28.2917	33.6299	31.0829	30.0718	31.5577	33.1926	32.2529	34.2161	32.2262
35.4739	38.2317	35.6373	37.0275	34.3937	36.2896	35.4179	33.8608	35.7629	35.7100	36.1301	37.4045	32.3224
26.6924	30.4881	23.4325	25.4596	25.0039	27.0775	25.2234	24.4554	28.3697	23.5852	26.8329	28.7092	27.7873
26.7132	27.8775	24.2216	26.8626	26.0855	28.8910	26.3499	26.2878	25.2770	26.2406	25.6963	27.8046	30.5409
29.1135	30.0750	30.3971	30.4732	30.3263	32.4228	30.5218	30.2813	28.0617	31.3914	29.0962	31.7904	32.5585
31.6716	36.2443	30.9747	31.4120	34.7978	35.6419	36.4350	33.8842	31.3579	28.6906	33.4345	33.4258	34.1070
31.3204	35.8918	31.0174	32.0453	33.2697	36.1956	35.1009	34.3487	31.7863	28.1893	33.5586	33.7118	32.3355
38.5155	36.6056	32.0014	38.1656	29.4123	34.5503	36.7183	29.9465	36.3710	37.1443	37.6019	36.7216	34.2520
37.3057	38.9220	31.0933	36.4122	35.3064	36.0646	34.9999	34.7176	35.2512	34.2500	33.4558	36.2296	31.4146
37.7740	40.0000	33.6270	35.8646	34.5310	36.7975	36.7708	36.1824	35.4337	39.0650	36.1235	40.0000	35.0222
39.2522	38.6791	37.8487	40.0000	40.0000	37.0517	40.0000	40.0000	35.6985	37.4493	37.1233	39.0567	28.3272
37.6396	38.7799	36.4865	39.4681	34.8519	38.0954	36.0906	38.1714	37.2150	37.5334	37.2583	39.3400	33.4882
34.0295	35.1889	33.2779	34.2616	34.0586	34.8073	34.8261	34.8636	35.0223	33.9648	34.2867	34.3217	33.6475
25.5752	26.3988	24.1837	26.0811	25.4464	26.8778	24.7150	25.9224	24.0862	25.7242	24.6614	26.7666	25.7265
31.5396	33.9966	31.0534	31.7797	31.7170	34.1640	30.6776	32.8762	31.6233	30.5393	31.5024	36.2918	29.6266
19.9006	22.2709	18.7951	20.3065	19.2750	22.5432	19.0052	19.9543	20.0735	19.9951	19.5002	22.5250	22.2756
24.0892	27.0654	23.0160	25.1074	23.8311	26.2100	23.3421	24.5508	24.3628	24.3318	23.8034	27.2158	28.3595
38.2971	40.0000	32.5062	40.0000	33.6068	36.3335	39.0686	36.6270	39.1034	40.0000	37.7092	39.6147	35.2379

Table S3. Median Ct values of plates A and B.

30.1994	40.0000	28.9779	29.7797	27.6827	26.9799	28.5571	27.7296	28.7771	28.4717	30.0947	30.6696	26.9966
23.9423	40.0000	22.9819	23.9730	22.8583	27.9723	22.7902	23.0216	23.3247	25.1581	25.2241	25.8649	24.7138
38.2452	38.8613	37.4288	37.4881	38.0483	37.8243	37.6874	37.0613	38.1146	37.2817	38.3836	40.0000	20.4951
31.9615	30.2229	25.3860	31.0314	23.8748	26.3942	27.5364	26.4395	25.3217	28.6646	28.5807	34.9905	25.7116
36.9824	34.7596	33.1030	35.5239	35.0618	37.4611	35.4491	35.4217	32.5547	34.1168	32.7737	39.4909	34.5685
24.9832	26.4556	21.5200	25.8717	22.2648	26.2395	26.6613	25.6265	24.5269	26.0112	24.9606	26.0000	25.1849
31.5763	32.5266	27.1521	29.0206	31.3555	32.5053	35.7844	33.3896	28.1577	33.0951	29.3271	34.1086	28.2624
32.3990	28.6235	27.9228	32.2193	28.9382	33.1492	31.4837	29.6376	26.6359	31.4164	29.6569	32.1866	32.5000
21.8920	24.3294	20.6490	24.5732	23.6214	26.4860	22.2410	24.4130	22.4113	23.5973	22.4490	24.3867	25.8717
40.0000	40.0000	39.7943	40.0000	36.0314	40.0000	38.5983	38.2192	40.0000	38.4614	40.0000	40.0000	22.6060
38.9927	38.6607	38.0661	37.9254	36.9870	38.3691	36.9266	39.1444	38.6980	37.9732	36.7930	36.7511	33.3997
21.5094	22.3022	19.3395	21.7975	21.3085	24.3080	20.7427	21.9063	20.5073	22.3815	20.4873	20.3298	21.6339
38.4683	38.1371	31.6321	38.7208	27.7835	34.7058	39.9324	27.8157	38.4201	37.4815	40.0000	38.6784	21.1657
40.0000	37.4047	31.0768	37.8934	28.0310	34.3509	40.0000	27.8868	37.6651	37.8778	37.7111	40.0000	35.0696
25.4815	26.8373	24.2461	27.8611	28.6407	31.0132	26.4202	27.8334	25.8771	28.3887	25.2115	22.7881	30.5184
21.9745	24.7380	23.4656	22.5762	20.1195	27.3679	19.0949	19.9678	23.0104	22.6144	23.3310	21.6226	24.5498
22.8807	25.2653	24.0450	23.8792	22.0077	27.4692	20.4463	21.0634	23.9248	23.8406	24.6900	21.6514	25.5551
26.5958	30.8326	27.5721	27.9802	25.5810	32.9557	24.8150	25.7316	28.5331	27.3369	28.5950	27.2429	33.3519
36.3391	34.3351	30.9907	35.3565	32.4886	34.5411	34.9108	34.7294	34.4776	36.4605	36.2212	35.2655	28.5000
37.0224	39.2623	34.9557	36.4093	36.2995	38.0464	36.5677	39.3615	36.2610	37.6281	36.8243	34.3627	24.8599
40.0000	40.0000	38.0367	40.0000	38.6539	40.0000	40.0000	39.8935	39.7406	40.0000	40.0000	39.0294	34.1246
39.1465	40.0000	36.4826	35.9133	37.1466	36.5480	38.1682	37.2223	40.0000	39.1093	37.4301	34.2451	33.0495
30.9183	31.8830	26.9372	30.9742	29.6040	32.2644	29.5344	31.5856	29.1445	30.8363	29.2749	29.7978	28.5095
24.6845	26.4684	23.7780	25.3530	24.9640	27.6786	23.8147	24.9472	23.6995	25.3012	23.9643	24.6150	25.5484
38.9251	35.5941	33.4204	36.7069	37.1977	35.8448	37.6380	40.0000	30.1230	36.4640	32.1687	37.5870	33.3028
38.3451	38.9959	35.1163	39.0456	38.8970	40.0000	37.2931	39.3505	37.8193	38.9831	35.8580	38.8991	35.0000
37.3421	40.0000	30.3247	37.1974	34.4700	38.3436	34.4789	35.1677	37.3017	33.1239	34.0535	37.9244	34.2610
22.8408	24.1214	21.8583	23.0675	23.5130	25.8816	21.9210	22.9797	21.9021	23.2049	22.4483	23.2747	24.1239
32.1728	33.1670	28.9220	30.0383	29.1650	33.8023	31.8853	29.7349	29.6731	31.1781	29.4535	33.0305	31.0000
30.2092	31.5449	26.2452	30.3329	28.6433	32.6675	28.4201	29.6963	28.9695	28.7179	30.3895	31.1479	32.8129
28.3564	29.5011	24.4678	27.9283	27.4052	31.0840	27.3268	27.1754	26.8889	28.5268	26.6711	27.2774	30.6113
27.0083	26.8395	24.9730	26.1645	26.5446	27.6008	25.2479	26.7808	24.8126	27.0885	25.8551	25.3802	27.0662
38.6871	37.9356	35.8996	40.0000	38.2946	38.1789	38.5640	38.8336	39.2506	38.5525	38.7747	35.2036	33.2516
34.9307	31.9877	32.8398	34.6747	32.5664	34.1202	33.7858	31.3248	33.1027	35.4920	34.1283	32.1895	26.6972
27.3158	40.0000	25.4354	25.3527	23.9535	26.8038	25.8176	24.2476	26.8977	26.0748	25.8347	26.1279	25.5863
26.5624	29.2666	26.3502	26.6366	25.0622	27.9658	26.5944	25.8123	27.7549	27.7096	26.4086	26.5626	24.4868
32.2017	32.0991	30.0199	31.0506	29.6526	31.6082	31.5682	28.6573	31.8316	31.4358	31.5479	32.8498	25.7346
33.8955	33.8939	30.9297	34.0045	33.0145	35.6627	33.5564	33.0082	33.3717	32.2643	31.9057	35.4943	32.1057
34.1973	33.7494	30.9770	34.4326	33.4291	36.1673	34.0301	33.5103	33.7012	32.3085	32.6315	33.7810	34.9398
30.6372	31.3929	28.8377	30.6841	31.0972	33.1937	29.3787	32.4464	29.9030	30.8704	30.1959	31.7955	30.8674
38.7914	37.7617	37.5440	37.7013	32.3309	39.5326	40.0000	31.8499	36.3675	37.3196	38.4216	37.7485	34.1748
33.3989	37.2396	29.5058	34.0849	32.8184	34.3029	32.9609	35.5167	34.3492	31.4794	31.9335	31.0320	27.4535
39.1534	38.4162	40.0000	40.0000	37.3299	39.7898	39.5969	40.0000	37.8354	37.4449	38.8373	39.5327	34.2029
31.1146	40.0000	27.5127	29.8344	28.4753	31.2644	31.5284	29.3547	25.1256	31.8872	30.6341	29.9814	29.0000
40.0000	38.8274	37.1480	38.5922	34.8856	38.8609	37.2435	39.8629	40.0000	38.1306	39.7576	35.8696	34.7127
26.3142	30.3726	29.8009	26.7763	25.2230	31.9233	24.5351	25.5065	27.9206	28.3119	29.2249	26.5856	26.4069
33.9708	34.8713	32.3602	34.3036	34.4020	33.9692	33.4188	31.6273	32.4102	34.1168	32.2616	32.9058	34.0068
33.5408	36.0054	29.5031	33.3606	31.7521	33.6869	32.9856	33.7404	32.7573	32.8164	33.6995	35.2625	30.0000
38.1178	39.0568	38.9263	39.5245	38.5932	39.4726	38.1278	37.6195	38.2742	40.0000	38.1177	39.7013	36.3883
38.3262	37.3719	35.7866	36.6958	34.8585	37.8040	37.4064	33.3616	34.0706	38.2493	35.8921	37.2160	34.0663
33.4990	36.8852	33.9615	34.5001	33.3469	37.1003	34.0974	34.3197	36.1765	33.4830	34.6583	36.8878	34.4364
40.0000	40.0000	40.0000	40.0000	40.0000	40.0000	40.0000	40.0000	40.0000	40.0000	40.0000	36.7615	36.5756
22.1970	24.8055	23.7248	23.1691	23.4186	27.2060	21.3077	23.7742	21.8159	25.4224	23.5673	21.9084	24.0000
22.6194	24.9958	23.3848	23.8475	23.9834	28.0357	21.7049	23.7420	22.5697	24.5499	24.2160	22.9293	25.4469
20.9209	21.1249	19.4838	20.8920	21.3171	22.2303	18.8849	22.4674	18.8508	21.4015	19.2785	19.9558	23.7375
36.5810	38.1231	28.5899	38.1948	26.0467	37.2609	29.8419	26.5472	35.3077	40.0000	40.0000	37.4294	33.1608
32.9822	33.7334	31.3355	33.8986	32.0708	32.6524	34.0079	31.9938	31.6291	33.6374	31.4699	30.6579	31.5259
40.0000	37.7682	31.6398	39.0394	28.4351	37.2447	39.7176	28.8148	38.8590	36.9070	37.1514	40.0000	34.0186
27.5191	29.7649	26.5821	27.4265	28.0496	30.0965	27.1035	28.7672	26.2803	29.2298	26.8328	29.2676	23.3273
39.3064	40.0000	37.9508	40.0000	38.7994	40.0000	40.0000	36.8357	37.3351	37.1016	37.0139	40.0000	31.6307
40.0000	40.0000	39.1926	40.0000	40.0000	37.6194	40.0000	37.2082	40.0000	38.2913	39.7041	30.4850	32.8681
40.0000	40.0000	40.0000	38.1674	38.3078	38.1211	40.0000	39.6596	40.0000	38.4990	38.5685	39.2349	26.6798
40.0000	40.0000	38.1928	39.9460	40.0000	40.0000	40.0000	40.0000	40.0000	40.0000	37.7309	40.0000	33.8353
38.3762	39.8046	39.4196	40.0000	39.9333	40.0000	38.5578	40.0000	37.2739	39.6906	38.8075	39.1535	35.2355
39.9503	40.0000	40.0000	40.0000	40.0000	40.0000	38.7204	39.6733	37.9729	39.0591	39.1975	38.7053	29.5193
23.5510	25.6596	22.4348	23.7440	24.0635	31.2658	23.2380	24.4142	23.4415	22.7980	23.2691	21.2546	25.3525
40.0000	39.1046	34.8849	38.4682	29.6454	33.9010	38.9903	29.3263	38.8306	39.7545	38.0599	38.1439	34.7650
27.6636	29.3890	25.9082	27.6373	28.1455	29.2613	26.8226	27.4942	26.2640	27.8918	26.4455	27.5976	30.6341
40.0000	37.2221	37.8083	35.7376	39.3747	37.5387	39.4568	38.2678	38.2853	37.5152	37.1965	40.0000	5.6537
31.0437	32.5266	30.2466	30.1200	32.2200	34.1191	30.9058	32.0093	28.8203	31.6625	29.2302	30.4464	36.6170

Table S3. Median Ct values of plates A and B.

27.5898	29.1058	27.4641	28.4556	28.1820	30.8793	26.9514	27.7761	26.4368	27.9924	26.7055	27.3287	30.0000
33.4168	35.2005	32.2254	33.1813	34.8401	35.2326	32.0530	35.2670	35.0774	34.1157	33.1048	31.8230	33.0000
27.0268	29.5347	26.5773	27.3444	28.9673	29.8329	26.7653	28.1049	26.2676	28.6455	26.5288	27.3435	30.9747
25.0116	32.6000	26.5514	26.1138	26.8483	31.1475	23.6052	27.0365	31.4031	27.1405	28.4306	29.6897	18.4566
40.0000	40.0000	36.4007	40.0000	34.2442	40.0000	38.5312	34.4258	40.0000	40.0000	39.8368	38.7014	29.7352
32.2806	32.4374	29.0113	30.8917	32.7404	33.7981	29.4764	33.0063	29.1362	32.6568	30.6972	31.1396	29.1522
26.7236	26.2614	24.9713	27.5524	28.5941	28.3656	25.9729	28.6578	25.3898	27.7183	25.1784	25.2960	28.0000
28.8588	30.1517	27.2525	29.1409	29.0354	31.3620	28.3962	29.1026	28.3890	30.8248	28.0632	29.5748	31.9141
24.4895	27.0297	22.8918	25.6312	23.9884	28.1287	24.4110	25.9116	24.5629	26.3097	25.4323	25.4245	24.0000
36.6535	40.0000	39.1589	39.5099	40.0000	40.0000	40.0000	40.0000	40.0000	37.4893	40.0000	40.0000	26.2567
40.0000	37.7235	31.8127	37.4673	27.3182	35.7747	40.0000	28.6493	40.0000	40.0000	38.4039	40.0000	32.5193
38.1648	37.6848	31.9585	37.3649	28.9925	35.2691	37.8381	29.2303	36.8586	37.1810	36.8782	34.1405	32.7274
39.4972	40.0000	39.2378	40.0000	34.0492	36.5195	40.0000	38.7615	40.0000	39.3562	39.7434	31.8042	35.9741
40.0000	40.0000	37.2180	40.0000	40.0000	40.0000	38.8919	37.0562	38.3571	39.1089	38.1461	38.3109	30.0000
34.4219	40.0000	35.8841	33.8083	34.9370	33.8037	33.4322	35.6643	34.0512	33.0325	32.4359	30.1392	35.7984
36.4643	36.4186	37.4804	36.3982	37.0537	37.7955	36.2980	38.6487	36.6203	37.0632	36.1587	33.5392	39.8442
27.1942	29.9807	25.8444	27.5262	27.2945	30.8513	26.5674	28.2619	26.9623	27.8523	26.2386	29.2230	30.6392
22.5010	24.1841	20.5593	23.9324	22.9393	27.2993	22.7682	24.7490	22.7631	23.3079	21.3146	22.9699	25.3234
23.1175	23.4727	20.6749	23.9984	24.6966	27.4214	23.3204	24.6694	21.7993	22.7998	21.3407	24.8002	23.0000
26.0881	24.4214	20.4662	23.8430	25.4748	26.8850	22.5600	24.1626	23.0235	26.0449	25.3149	27.6561	24.5988
33.8884	33.8583	27.1921	33.6036	31.4991	37.9284	30.4446	32.4557	32.8734	29.9817	32.2034	35.4547	34.2723
23.1984	25.5616	21.5626	24.5757	23.5917	27.2492	22.9307	24.7235	23.3909	24.0112	23.1988	25.4154	24.0000
26.4551	27.9968	23.5605	26.4388	26.1964	28.4943	26.0975	26.8353	25.8473	26.8501	25.4358	26.8868	24.0000
40.0000	39.4463	39.9589	38.5642	38.7366	40.0000	38.0768	40.0000	40.0000	39.3514	39.1384	38.5593	40.0000

Table S3. Median Ct values of plates A and B.

NBM5	KG1	NB4
33.2648	36.1460	26.4927
35.5773	40.0000	30.6387
31.5887	38.8029	40.0000
26.3803	27.4486	25.7926
24.6633	24.5511	23.0871
21.6394	23.6427	22.5505
30.6145	27.6460	28.3004
25.1812	21.5303	21.2318
23.4305	21.5317	21.5808
25.6222	23.3154	23.3828
22.7126	23.7810	24.0940
24.8634	24.2073	23.5379
29.3713	28.1987	27.8835
24.1621	27.1201	22.5892
23.1716	27.9014	26.2554
24.6897	27.5334	25.6357
26.7358	25.7594	25.1972
28.9454	29.1492	28.4594
28.3945	31.7535	31.2668
23.0602	25.9742	24.1525
24.3429	25.7510	24.3457
22.9207	24.3093	23.0088
29.4948	31.3138	29.2112
24.2642	24.4141	24.1072
24.8953	25.6063	24.9824
26.1848	26.7980	26.4893
27.3692	30.3671	29.3174
30.9513	40.0000	40.0000
31.6191	37.7568	31.3139
37.6342	40.0000	37.2673
40.0000	40.0000	39.7709
23.7095	23.1172	22.4464
32.1240	38.0184	34.3922
33.9734	40.0000	29.7595
29.9159	28.5834	29.2549
30.9614	26.9944	34.4944
30.7408	26.8136	33.9147
24.9775	24.3072	22.5172
35.9550	38.7974	38.5892
38.0659	40.0000	39.5530
25.6884	22.9548	22.4214
31.5727	30.8838	27.7866
38.3122	40.0000	40.0000
36.0508	35.3069	32.9243
27.5435	34.6456	31.2741
28.8512	25.9997	30.7930
30.5511	28.0862	36.2848
26.7197	28.2768	32.8540
38.4203	40.0000	40.0000
32.7616	32.6814	30.3634
33.9407	33.1698	32.2299
37.7235	40.0000	29.7459
26.7530	24.6487	33.2332
27.5666	26.4843	25.9613
29.8990	34.1255	32.9340
32.9247	28.0706	37.5330
29.7734	26.6384	33.1401
36.5916	40.0000	37.5915
35.6543	38.7906	40.0000
33.6920	40.0000	25.4168
38.1835	37.5165	40.0000
37.6676	39.4363	40.0000
32.5394	36.4341	36.2854
25.9477	27.6175	26.2906
33.5180	35.6066	33.6082
22.3744	21.8729	19.8209
26.8879	27.8160	26.2414
36.6087	40.0000	39.0858

Table S3. Median Ct values of plates A and B.

27.5670	32.7175	27.8977
24.1920	25.5749	25.2791
35.7912	39.5059	39.6458
27.1017	30.1714	26.8804
36.6633	40.0000	40.0000
21.6087	30.4407	36.8188
26.6691	28.7450	38.8550
28.9320	31.6866	32.8767
25.6948	26.2739	26.0581
40.0000	40.0000	40.0000
36.4711	38.6403	40.0000
22.2440	23.3987	22.4771
37.2916	40.0000	40.0000
36.8988	40.0000	40.0000
28.7037	27.7028	27.1623
25.7022	24.8462	23.1277
26.1538	24.5725	22.9628
30.9441	32.2022	29.9827
30.0018	38.8773	25.3481
36.0166	40.0000	34.4411
36.4381	40.0000	32.4800
33.2825	33.1103	34.0310
29.3279	32.9016	31.6263
25.2829	26.1208	25.2791
36.4836	36.0657	40.0000
39.3445	37.0994	38.1502
35.6578	37.1883	34.5618
24.2474	24.8686	23.6246
31.6861	32.9398	27.9329
27.9347	26.9637	28.7148
28.3392	31.8236	29.9644
27.0448	28.4379	26.9119
38.0989	40.0000	38.9070
29.6393	25.7869	35.5092
26.7388	24.0308	24.4718
29.2893	31.4892	26.2852
29.2388	25.0679	32.2628
33.6563	38.1109	35.8392
35.3946	40.0000	40.0000
31.4968	34.0849	32.6020
37.4866	35.1585	40.0000
36.8067	40.0000	40.0000
40.0000	40.0000	40.0000
29.8596	24.5711	25.0453
39.7086	38.2580	39.2132
30.2663	29.4698	27.4426
31.7884	25.7979	36.4032
31.1132	33.3884	32.4632
38.5884	40.0000	40.0000
36.3814	32.4165	33.0384
36.9683	35.4536	35.5082
38.9568	40.0000	40.0000
26.9488	24.4753	22.4251
27.6217	25.6283	23.0883
20.4195	24.0196	21.9154
38.6430	40.0000	40.0000
31.8282	35.9088	35.2488
40.0000	40.0000	40.0000
30.1552	27.5478	26.7727
39.0887	37.0604	40.0000
40.0000	40.0000	40.0000
38.2012	40.0000	40.0000
40.0000	40.0000	40.0000
39.8877	40.0000	37.4680
33.5718	33.8664	34.3527
25.3199	24.6953	25.5632
38.1931	40.0000	40.0000
29.1595	28.9892	26.8955
38.3281	40.0000	40.0000
32.0318	31.7551	31.9400

Table S3. Median Ct values of plates A and B.

29.8063	30.3276	31.2487
35.4684	34.0381	33.3541
29.0387	27.9578	27.5464
29.3105	30.2487	28.5340
40.0000	40.0000	40.0000
30.5322	33.9174	35.8134
28.1859	28.1376	25.4564
31.1299	33.1283	31.9670
24.2160	23.7099	25.0146
38.8127	40.0000	40.0000
37.6899	40.0000	39.0076
38.7146	40.0000	40.0000
39.7021	40.0000	40.0000
39.9400	36.8274	40.0000
32.8136	34.8752	33.6923
36.9554	38.5320	37.6513
28.9199	30.3532	29.4278
25.5388	25.6543	24.8364
25.9876	25.1384	27.0496
25.7262	25.2569	25.6758
34.9937	31.4685	37.7048
24.5314	26.6050	26.9047
27.8988	30.5411	29.9661
38.5545	40.0000	40.0000
