# Supplementary material for: Distinctive Patterns of MicroRNA Expression Associated with Karyotype in Acute Myeloid Leukaemia
Source: PLoS One. 2008 May 14;3(5):e2141. doi: 10.1371/journal.pone.0002141 (PMC2373886; doi:10.1371/journal.pone.0002141)
Supplement: Table S4 — Real-time PCR results, normalised data. The spreadsheet contains the values of 123 miRNAs after normalisation for 100 AML samples, 2 cell lines (NB4 and KG1), and 2 normal bone marrow controls (NBM4 and NBM5). Median Ct values were normalised to the negative plate median (as described in the method section of the manuscript) so negative values are the low abundant miRNAs, positive the more abundant. (0.24 MB PDF) [file pone.0002141.s004.pdf]

Table S4. Normalised Ct values for plates A and B.

Samples	1	2	3	4	5	6	7	8	9
hsa-miR-9	-5.8263	-5.7306	-1.0322	-6.7131	-1.9224	4.7203	-7.7831	-5.3127	-5.8459
hsa-miR-9*	-8.1343	-7.9635	-4.9623	-9.8155	-5.6439	-0.1049	-11.0377	-7.7575	-8.9795
hsa-miR-10a	-5.9368	-7.0553	-5.6711	-6.5346	-3.5621	-6.4859	-11.0419	-6.8861	-6.6018
hsa-miR-15a	2.9198	6.9021	2.3454	3.3965	3.7404	4.0275	1.6569	3.5683	4.3511
hsa-miR-15b	4.8772	5.8792	4.0621	3.7234	5.2644	5.2657	3.4192	3.7609	5.5545
hsa-miR-17-3p	2.9151	1.8771	0.0916	-0.8678	0.6381	0.9436	0.1924	-0.3771	1.5441
hsa-miR-17-5p	7.1289	4.0837	5.2995	4.2881	5.7354	6.0567	5.3606	3.7166	5.6423
hsa-miR-19a	6.9234	7.0841	6.6400	4.9467	7.0978	7.4088	4.2671	6.0593	5.2413
hsa-miR-20	4.8615	6.1099	4.5799	4.2638	5.9507	5.0463	2.4348	4.8005	4.6988
hsa-miR-21	5.9226	8.5097	7.3898	6.5638	8.5746	8.2799	3.5351	7.1278	7.6464
hsa-miR-23a	4.2330	6.0185	3.8292	4.1623	6.0133	6.4919	3.2048	4.8734	5.8070
hsa-miR-23b	0.0049	1.6389	-0.3023	-0.0540	0.9539	1.5388	0.9718	0.1511	0.2016
hsa-miR-25	5.4414	6.0287	4.4890	2.8280	3.8884	5.7546	2.0736	5.7543	5.6841
hsa-miR-26a	7.2126	7.3291	4.9585	normalised	6.1843	5.5246	4.7293	4.8712	6.5165
hsa-miR-26b	5.8439	6.0356	3.4799	3.7239	4.7268	4.6890	2.5370	3.8514	4.7641
hsa-miR-27a	4.1679	2.6628	3.7228	1.6107	4.2409	5.1186	2.7062	2.6712	3.9606
hsa-miR-27b	1.1926	2.4817	0.2017	0.4386	2.4684	0.5387	1.7141	1.1495	2.1151
hsa-miR-28	-1.4497	1.9656	0.0714	-0.9404	1.1591	0.5095	-1.8481	0.6555	0.5661
hsa-miR-29a	3.8570	6.1619	6.3775	3.9984	5.2520	3.5108	3.0838	4.1428	6.4596
hsa-miR-29b	3.0418	4.5606	4.1799	2.3091	3.2885	1.2878	1.6567	2.6313	4.9087
hsa-miR-30a-3p	-0.6925	-1.1549	-0.0174	-1.6292	0.6586	0.2789	-2.1184	0.9595	0.7006
hsa-miR-30b	5.9728	7.2511	5.3651	4.6779	5.9496	6.4575	4.5338	5.8544	6.3557
hsa-miR-30c	5.8310	6.3831	4.9933	3.8634	4.4292	5.4477	3.3519	5.0295	5.5241
hsa-miR-30d	3.7785	5.4397	2.7454	2.3917	3.8895	4.2732	2.5202	3.5291	4.4472
hsa-miR-30e	3.7183	4.3438	2.2502	0.6384	2.3081	2.4826	1.8669	3.1312	4.1592
hsa-miR-34a	2.9935	3.1418	1.8360	1.9115	4.4203	3.0976	0.1771	1.7572	0.8592
hsa-miR-34c	-10.8480	-5.8137	-8.0757	-8.2474	-8.3814	-11.3558	-8.8942	-13.3883	-12.5310
hsa-miR-92	5.3058	8.5993	6.1423	5.7564	7.0731	4.6564	5.7143	6.3452	6.2616
hsa-miR-96	-8.1757	-10.0509	-8.1506	-10.0408	-6.5397	-9.8173	-5.8728	-9.4641	-12.5310
hsa-miR-98	1.1011	2.3118	0.6675	1.0290	1.9197	0.6045	-0.1771	-0.6100	-0.1554
hsa-miR-99a	0.4944	-3.1102	2.4376	1.0362	-2.2389	-5.1801	1.0393	-7.7297	-1.1526
hsa-miR-100	0.5310	-1.2996	3.3459	1.4375	-1.8959	-4.5123	0.9902	-6.8380	-0.4889
hsa-miR-103	5.2016	6.2942	3.9309	3.7748	5.2770	5.4561	3.6387	3.8333	5.8416
hsa-miR-106a	5.2765	4.1977	4.6276	3.1974	5.7523	4.9034	3.8156	3.8731	4.1648
hsa-miR-107	-0.9303	-0.8847	-3.2108	-3.2635	-1.0789	-0.7507	-4.5113	-1.8586	-2.5226
hsa-miR-124b	-3.2587	-3.9726	-0.8112	0.7548	-0.4141	-1.8154	0.4783	-5.3241	-1.3030
hsa-miR-125a	6.1056	4.0079	1.4722	-0.3804	1.0679	-2.8024	0.4720	3.1909	-0.1801
hsa-miR-125b	-7.2309	-1.0295	-9.9386	1.7283	-2.8371	-11.3558	0.6467	-7.6299	-12.5310
hsa-miR-126	7.0681	9.1464	-0.0675	0.1408	0.4141	-4.2707	2.3603	-1.8936	6.5893
hsa-miR-127	-10.8480	-2.0218	-5.3785	0.0540	-7.1077	-11.3558	-11.5447	-7.4452	-12.5310
hsa-miR-128a	-3.3496	-2.7952	-1.2899	-1.8336	-1.8367	-2.3418	-4.5228	2.4113	1.0435
hsa-miR-128b	-4.7767	-5.9790	-1.3729	-3.4297	-4.2411	-4.0933	-5.7962	1.1972	0.1554
hsa-miR-129	-9.3008	-10.0509	-8.0896	-7.3564	-7.1421	-6.5907	-7.9273	-7.5799	-9.9218
hsa-miR-130a	5.0372	7.3487	3.3851	3.8050	0.4919	-4.0480	3.2577	4.0437	5.6806
hsa-miR-130b	3.8337	4.3061	2.8312	1.1838	2.4429	3.1426	1.4591	3.0122	3.3430
hsa-miR-132	-0.3292	-0.2247	1.6380	-2.9146	-2.4875	0.0425	-2.7197	-2.1882	0.5206
hsa-miR-133a	-4.2822	-4.2727	-5.4976	-7.2161	-4.2251	-6.1718	-6.0102	0.3425	-5.2853
hsa-miR-133b	-3.3647	-3.2438	-5.5320	-6.1417	-4.3009	-6.6121	-5.4217	-0.0864	-5.7062
hsa-miR-135a	-1.7454	-5.5853	-8.4225	-7.7172	-10.6400	-11.3558	-6.6557	0.0027	-6.9867
hsa-miR-140	3.2782	4.4654	2.6441	1.1539	3.2762	3.7773	2.0902	3.0680	3.9052
hsa-miR-142-5p	4.9728	5.5816	3.5234	2.9995	4.2445	3.0783	3.3096	4.3621	3.0517
hsa-miR-145	1.5021	0.2247	0.0174	-0.8585	-3.9045	2.2206	-2.6013	-3.6432	-0.4220
hsa-miR-146	7.1575	7.8644	5.6845	4.4179	4.0398	1.4979	4.6185	6.6443	7.8628
hsa-miR-147	-10.7480	-10.0509	-9.1233	-10.7177	-7.7759	-11.3558	-10.5731	-9.6154	-8.7133
hsa-miR-148a	-4.2252	-1.1146	-0.3879	0.8813	-2.2929	0.2876	-2.5447	-3.3192	-2.3013
hsa-miR-150	4.1008	6.4546	3.6588	3.7367	2.6591	2.6669	4.7473	5.0056	7.3730
hsa-miR-151	-2.7830	-0.9281	-5.8891	-6.7777	-3.5099	-2.1530	-4.2572	-5.7480	-3.8526
hsa-miR-152	-1.8118	-2.2727	-1.9871	-3.0088	-3.1617	-0.0425	-3.5446	-4.5161	-2.4077
hsa-miR-154	-3.1868	-3.5017	1.9677	0.7505	-7.3541	-4.9552	-7.0720	-2.9682	-4.0936
hsa-miR-154*	-1.2067	-1.0869	2.1415	1.7022	-7.3541	-4.4685	-7.0720	-2.8998	-3.7193
hsa-miR-155	7.3218	7.6057	5.3092	5.8699	8.9779	5.8763	7.7449	6.9276	6.3414
hsa-miR-181a	11.5293	11.8640	10.8392	10.5251	11.4567	8.9135	12.6945	14.7060	12.3867
hsa-miR-181b	10.4500	12.2488	9.8668	9.9891	10.9518	8.2485	10.7008	13.5061	11.2771
hsa-miR-181c	6.5088	5.5896	5.0960	5.4820	6.0576	3.4902	6.4698	8.7167	6.8429
hsa-miR-182	-0.7716	-5.6453	-4.5537	-5.0237	-0.9553	-3.5407	3.6155	-0.5239	-1.9217
hsa-miR-183	-6.5277	-6.4072	-8.7893	-10.2208	-5.1215	-7.9136	-3.2634	-7.7307	-7.8279
hsa-miR-184	-7.6764	-3.2288	-6.4491	-5.4914	-5.6117	-6.7800	-7.0720	-7.7307	-8.2118

Table S4. Normalised Ct values for plates A and B.

hsa-miR-185	1.5642	1.2583	-0.3399	-0.1044	2.8229	2.7615	2.3725	2.3928	2.6068
hsa-miR-187	-7.1883	-1.9817	-8.7893	-8.2498	-2.2490	-0.4938	-3.4274	-4.5591	-5.2978
hsa-miR-190	-4.1833	-1.6200	-4.7770	-5.2611	0.0475	-0.4705	-0.7471	0.0221	-3.8571
hsa-miR-193	2.0905	4.9435	0.3477	0.4885	1.0280	1.5898	-0.1452	2.1074	1.4674
hsa-miR-194	6.6601	5.2899	1.2040	0.9064	3.1427	3.9997	5.3769	6.1094	4.4742
hsa-miR-195	5.9439	4.8959	3.5479	4.6972	6.5270	4.5903	5.0238	5.7592	4.9268
hsa-miR-197	7.1743	6.0003	3.7143	1.9469	6.6328	6.0210	7.0195	6.9253	7.1154
hsa-miR-199a	-3.5178	-2.9707	-2.8185	-3.1811	-1.7782	-2.3238	-1.9439	-0.1029	-0.8326
hsa-miR-199a*	6.7326	8.3777	6.2039	6.2967	-7.3541	5.9679	7.6773	6.9343	8.4398
hsa-miR-199b	4.4506	6.6912	5.4069	5.6269	6.5385	5.2950	5.9305	5.6339	6.6241
hsa-miR-199-s	0.2984	-0.0686	0.4237	0.2966	2.1354	0.4011	1.8219	1.5570	3.8175
hsa-miR-200a	-1.6037	0.5986	-2.6514	-1.9652	-1.3977	0.8250	-0.3647	-2.4621	0.3437
hsa-miR-200b	-1.5750	-2.1080	-3.0936	-2.5401	-2.1297	0.2019	-1.3213	-2.6385	-0.2835
hsa-miR-200c	2.3661	1.1390	-0.4068	-0.1443	2.6301	3.3437	1.8246	3.7066	3.1166
hsa-miR-203	-0.6327	0.0686	-2.1478	-0.3200	-0.8682	-8.2885	-2.1497	-1.5670	-3.3703
hsa-miR-204	-0.2984	-5.0809	0.4321	-1.6755	2.8720	-1.8980	-0.6161	0.0268	0.2844
hsa-miR-205	-7.6764	-6.4072	-8.7893	-10.2208	-7.3541	-5.5647	-4.1603	-7.7307	-5.3640
hsa-miR-210	1.7942	3.3286	2.6473	2.1879	6.0499	2.2116	3.2621	5.2673	2.5817
hsa-miR-213	5.2792	6.1869	5.1306	5.5701	5.9453	3.8237	6.9411	9.3256	4.1122
hsa-miR-214	-4.3350	-2.1045	-3.6959	-1.1844	-1.3665	-2.3307	-0.7645	-0.0221	-1.3279
hsa-miR-215	3.1609	-0.2756	-2.5190	-1.6891	-0.3915	0.5377	1.3871	2.6290	1.0100
hsa-miR-218	-2.7565	-3.6961	-3.1626	-5.0900	-5.3881	-5.1140	-2.7782	-1.0131	-4.7381
hsa-miR-219	-0.4196	-1.5715	-3.6530	-4.0038	-1.6620	-1.6931	0.1452	-0.0463	-1.8162
hsa-miR-221	9.1425	8.3969	7.9191	7.6100	8.9077	10.5757	10.2787	8.5113	8.0405
hsa-miR-222	10.0994	8.6649	7.7817	7.2813	9.0288	10.1566	10.5283	7.9800	9.0496
hsa-miR-224	-5.7203	-6.4072	2.9439	2.3806	-3.3260	-5.7436	-1.4319	-3.0382	-10.0786
hsa-miR-296	1.0342	-0.3186	-1.3498	-0.6940	2.7675	1.5724	2.8650	-0.4278	0.2340
hsa-miR-299	-2.6579	-2.0885	1.7455	1.3032	-7.3541	-5.9648	-5.4713	-4.4976	-3.7508
hsa-miR-301	5.3569	4.5729	2.6767	2.5967	5.7919	5.6029	4.9129	5.0850	2.8950
hsa-miR-302b*	-7.6764	-6.4072	-8.1127	-10.2208	-7.3541	-8.2885	-7.0720	-7.7307	-10.0786
hsa-miR-302d	-7.6764	-6.4072	-8.7893	-10.2208	-6.4009	-8.2885	-7.0720	-7.7307	-7.0634
hsa-miR-320	9.0280	10.6350	6.5773	6.3225	9.4626	8.9121	9.3205	9.8449	9.9144
hsa-miR-323	-2.0567	-2.3452	0.3682	0.7313	-7.3541	-5.5031	-7.0720	-3.9293	-4.6247
hsa-miR-324-5p	4.2986	6.1111	2.7618	3.2019	5.9118	6.0354	5.0093	5.9399	5.1710
hsa-miR-325	-7.6764	-6.4072	-8.1586	-8.4920	-2.9507	-7.2339	-4.4564	-6.8912	-10.0786
hsa-miR-326	1.6759	3.0581	-1.4940	-0.6638	3.0265	2.0337	1.7827	2.3685	1.4463
hsa-miR-328	5.3993	5.1114	1.6440	2.1890	5.4700	5.7762	4.5493	4.9646	5.4998
hsa-miR-330	0.3938	0.1945	-4.0555	-3.8840	0.8177	0.4962	-1.0132	-0.2161	-0.0147
hsa-miR-331	6.0765	5.7951	2.6106	3.1917	6.1356	4.9367	5.8327	7.2439	5.0833
hsa-miR-335	6.6525	6.9903	3.8959	4.1269	3.8270	4.8076	2.5633	3.1735	5.6098
hsa-miR-338	0.9520	2.2412	-1.5287	-1.1768	-0.0475	-3.4030	0.7689	-0.6589	-2.5436
hsa-miR-339	4.9575	5.3384	2.2365	2.5335	6.5372	5.9348	3.2626	6.9210	4.0253
hsa-miR-340	3.6681	3.0315	1.9425	2.2885	4.8028	2.5883	3.8622	3.9620	3.8719
hsa-miR-342	6.4154	7.7867	5.4881	5.7839	6.7092	8.2986	9.3484	9.7253	7.2402
hsa-miR-367	-7.6764	-6.4072	-8.7893	-10.2208	-6.7683	-8.2885	-4.9754	-7.7307	-7.5080
hsa-miR-368	-2.1231	-3.0060	2.2848	0.7994	-7.3541	-5.5913	-7.0720	-3.3460	-5.8597
hsa-miR-370	-2.3701	-4.0899	0.3399	1.2336	-4.1245	-3.2987	-3.6334	-2.9607	-4.4606
hsa-miR-371	-7.6764	-6.4072	-2.5664	-10.2208	-7.3541	-8.2885	-7.0720	-7.7307	-2.1776
hsa-miR-372	-7.6764	-6.4072	-8.7893	-10.2208	-7.0747	-8.2885	-7.0348	-7.7307	-7.6620
hsa-miR-373	-2.8672	-6.4072	-2.8391	-5.2887	1.0466	-0.2019	-1.1633	-1.3800	0.0147
hsa-miR-374	4.3470	5.1739	3.0496	4.0085	6.7474	4.3794	5.8665	5.6299	4.4510
hsa-let-7a	6.4168	4.7545	3.8816	4.8867	5.2882	5.7064	4.3236	4.1159	4.8957
hsa-let-7b	7.5716	8.1280	5.3054	7.1562	10.9623	10.5516	8.8816	3.3209	8.6485
hsa-let-7d	8.0965	7.6958	4.7608	8.8761	10.0858	8.8368	7.4813	8.9902	6.6534
hsa-let-7e	3.4921	2.2906	-1.8355	0.1044	1.2020	-2.0227	0.9004	3.1251	-1.5232
has-let-7i	7.0637	7.4232	3.5387	5.7960	7.2568	5.5641	7.4706	6.8743	6.2029

Table S4. Normalised Ct values for plates A and B.

10	11	12	13	14	15	16	17	18	19	20
-4.8864	-5.8478	-1.5761	1.1869	-6.0131	-3.9227	-4.1178	-5.5047	-4.4361	-7.5562	-3.3902
-7.5246	-9.3501	-5.5432	-2.9756	-8.4127	-7.0531	-7.9105	-11.0348	-7.8630	-8.2122	-6.2954
-4.1178	-9.0386	5.1976	4.2862	-4.0396	5.6862	3.9832	0.6342	4.6549	-4.2379	4.8470
3.1796	4.0609	4.1043	3.4472	3.9099	4.9576	2.9424	3.1682	3.1745	1.5237	3.3516
4.5074	3.8423	4.8034	4.1396	3.7465	5.5636	4.4737	4.6020	4.4199	3.7124	4.1716
-0.0242	0.8727	1.5483	-0.3441	1.6584	1.7058	-0.7165	1.5234	-0.2405	1.6734	0.8746
4.9854	4.7662	5.0393	4.4734	5.7952	7.3088	4.5998	6.1132	4.9959	6.3254	6.1938
5.8149	4.9189	3.4504	5.2191	5.4887	4.6880	5.1732	5.5494	5.7242	7.7671	6.8500
4.3239	2.8139	4.9789	3.7257	6.1829	4.5473	4.0242	3.9983	4.6878	4.8162	5.0267
7.0250	4.1794	6.8582	7.8435	7.4770	8.7875	6.6504	5.5561	6.6506	5.2571	7.0002
5.0888	3.8513	4.9225	4.5792	4.8732	6.1859	4.8471	2.8505	5.3675	3.3867	5.2009
-0.0002	1.3631	-0.0910	0.0634	0.4776	1.0994	0.1219	-1.5274	0.0135	0.5726	-0.5390
4.5254	3.0739	5.1463	3.5360	6.1446	5.4199	2.4249	2.7006	2.8860	6.0875	3.2467
5.4104	4.6701	5.5010	5.2501	5.2761	5.9227	4.3764	4.7191	5.1312	7.3651	5.0936
3.8450	3.1191	3.9994	3.8925	3.7838	3.9903	3.3077	2.7204	4.1709	6.2940	2.9724
3.1945	2.8635	4.5077	3.6651	3.0300	6.4018	3.1858	2.2693	3.4772	4.3274	4.7452
0.5272	1.1200	-0.3717	0.9506	0.3195	0.3788	0.8069	-0.0949	0.4752	1.8064	0.8800
0.0002	-2.3260	0.3699	1.3739	0.4293	1.1299	-0.6338	-0.8303	0.1895	-0.8882	-0.3856
4.3907	0.9086	2.5441	5.5137	6.5247	5.1769	3.2034	2.3471	3.5852	5.1841	4.8731
2.8837	0.4864	-1.6744	4.3638	1.1487	0.8690	1.8880	1.5003	2.5570	2.5314	3.5179
0.1617	0.1584	0.2911	0.4641	1.1284	1.2366	0.0088	-0.7491	-0.0135	-2.1333	0.0439
5.9700	4.6852	6.5985	5.6595	7.9987	7.0970	4.8229	5.0089	5.5408	7.3300	5.4052
4.5630	4.2308	3.3119	4.7916	4.6636	4.6080	3.6660	3.9654	4.0961	6.2907	4.1000
3.5141	2.7105	2.9470	3.0097	4.0213	4.6829	2.6178	2.8711	2.9118	5.2401	2.4178
2.2788	2.8278	3.9173	2.8185	4.3791	4.2077	0.7700	2.1247	1.4843	4.7877	2.3981
0.2722	-2.4040	0.7825	0.7274	1.9682	-0.0747	3.2306	3.4708	2.1019	2.2650	3.4622
-10.7486	-6.6150	-12.1309	-13.4215	-8.5261	-10.0958	-7.3364	-8.7151	-8.6772	-6.3181	-11.6218
6.2048	6.2548	5.7235	6.4273	5.8469	5.8654	7.1418	7.6152	6.4164	7.7429	8.1227
-10.3678	-9.8938	-10.7768	-11.1159	-8.5261	-7.8327	-9.6643	-6.6200	-10.5980	-8.2122	-9.6808
0.7898	0.1037	-0.1784	-0.2944	0.9188	0.8030	0.6676	0.8468	0.2869	1.2785	-0.3804
0.2660	2.9931	0.4298	-1.4709	-1.1913	0.1458	0.4147	4.8659	-0.2186	-0.7876	0.4195
0.6590	2.5688	1.4362	-1.6961	-0.4243	0.0747	-0.3296	4.9169	-0.1721	0.0796	0.4344
4.6720	3.2935	4.1439	4.4466	5.2491	5.3171	3.8586	4.9044	3.1668	5.8268	3.0192
4.0539	4.9607	3.4320	3.1543	3.6061	4.9833	3.7936	5.4628	4.3877	4.1169	5.0542
-1.7038	-1.8554	-2.4495	-2.3627	-3.3098	-2.1636	-2.6877	-1.9292	-2.4064	-4.4603	-3.4471
-2.5007	-2.4439	-4.8246	-2.5642	-1.0122	-0.7582	-1.9225	-1.7907	-2.5423	1.3740	-1.8433
1.6949	3.7198	3.5849	-0.2346	0.7756	-1.3973	2.6588	2.8337	2.5554	1.6950	-0.1108
-7.9225	2.6325	-8.6981	-10.9683	-5.2385	-10.0958	-1.2926	3.5502	-0.7264	-8.2122	0.1273
4.2116	3.0377	0.0910	0.8313	5.9932	-1.1037	-1.3258	1.3003	-0.8253	6.7912	1.1000
-13.0541	-5.2597	-12.1309	-13.4215	-8.5261	-10.0958	-8.9587	-7.9155	-12.1610	-8.2122	-9.0341
-3.6512	-3.1647	-3.1818	-3.3891	-2.8072	-3.5846	-2.5580	-3.4029	-3.1528	-2.7337	-3.5168
-5.5121	-4.7316	-4.9598	-5.0394	-4.0111	-6.0634	-4.0470	-4.5283	-3.8076	-3.4442	-5.2468
-9.9843	-7.7528	-11.9814	-8.8707	-8.5261	-10.0958	-9.1065	-8.3694	-9.3584	-7.0155	-8.1530
3.9283	4.2865	4.4206	1.2797	6.4451	-2.0183	1.4069	3.7253	2.0159	6.7054	2.1454
1.7918	1.2920	2.1431	0.9536	3.7457	2.5988	1.7939	2.5557	2.4623	4.1018	0.9489
-1.2627	-2.3681	-1.4305	-1.5766	-0.3362	1.9153	-3.8481	-3.1464	-3.2891	-0.0796	-1.9550
-3.5488	-4.5237	-0.4213	-3.4879	-4.5593	-1.4706	-6.8747	-3.7081	-6.0547	-4.1337	-0.0439
-4.2284	-3.6192	-1.1010	-4.4427	-4.8408	-2.9021	-6.5946	-2.8205	-6.3406	-3.6271	-0.9733
-5.9824	-3.8387	0.8505	-5.4217	-8.0363	-5.4354	-6.3824	-8.7547	-5.2700	-4.6626	-5.7954
2.8304	1.7902	3.2917	3.5414	4.5190	5.1487	2.1264	1.9509	2.7336	3.9123	2.3319
3.5904	2.6196	4.6456	3.5254	4.8213	5.8218	3.6624	3.4157	3.9333	4.6275	3.8769
-1.2647	-4.9173	-4.5757	-2.3710	-0.0342	-0.4706	-2.1323	-0.3050	-2.9820	0.6437	-1.1689
4.3884	3.6559	6.3890	4.2104	8.0374	7.2255	2.5410	2.8210	2.2024	7.6989	3.4199
-11.0441	-10.1749	-11.2081	-12.6649	-7.8651	-8.2165	-7.0459	-10.7511	-12.3571	-8.2122	-9.4226
-2.3952	-3.0855	0.6318	0.0451	0.0342	-0.6012	1.6070	-1.3016	1.8477	-1.4808	1.6025
3.2886	1.8056	1.4178	6.9009	3.5232	4.6579	2.3558	4.8190	2.1296	4.6598	3.2928
-2.9696	-1.5347	-5.8862	-1.4656	-1.9888	-2.7243	-0.2221	-0.5538	-0.3314	-2.4483	-4.7144
-2.6416	-4.0660	-3.5842	-0.0451	-2.4280	0.1762	-0.0088	-2.7191	0.0327	-1.8849	-0.9762
-5.4690	0.1507	-9.9616	-6.1048	-6.9391	-6.4021	-7.7219	-3.5362	-7.3325	-5.1518	-5.4667
-7.6866	0.8057	-9.9616	-8.9048	-6.9391	-6.4021	-7.7219	-2.7756	-5.5318	-5.1518	-7.0767
7.3007	5.0275	4.9482	6.1395	5.1754	6.3734	5.8869	6.4963	6.5934	4.8147	6.6691
11.6487	12.3943	11.9923	9.5146	11.2386	11.5528	9.5709	11.6322	9.9130	10.5862	11.4768
10.6521	11.4871	10.1996	7.8195	10.1748	9.9181	9.1135	10.5781	8.7580	9.8699	10.1019
5.9019	6.4526	6.6160	4.0793	5.8438	6.7614	4.2360	5.6167	4.7609	5.5505	6.2158
-2.0969	-2.3676	-4.7382	-2.8886	-3.2677	-0.4458	-2.2527	0.0643	-3.0674	-2.7584	-3.2109
-7.6866	-6.9297	-9.9616	-8.9048	-6.9391	-6.3218	-7.7219	-8.0854	-7.6169	-5.1518	-7.0767
-7.6866	-7.7221	-8.7570	-8.9048	-6.9391	-6.4021	-7.7219	-8.0854	-6.3198	-4.2175	-5.5890

Table S4. Normalised Ct values for plates A and B.

3.3149	1.3470	0.8685	2.5562	2.0153	3.8775	2.3078	0.4229	2.1402	1.8849	2.0419
-2.4299	-4.6421	-6.8314	-0.7556	-3.7629	-2.5170	-1.5308	-3.6215	-2.5262	-5.1518	-1.6260
-3.9377	-0.7710	0.2978	-3.9994	-2.3033	-2.6159	0.8698	-5.6981	1.4878	-3.0343	-2.2639
3.6516	3.4336	-1.3099	1.4072	1.2434	-0.2395	3.2378	1.3249	2.8700	1.1365	2.3528
3.4824	3.6930	2.8326	3.8253	2.7925	4.4419	2.9976	4.5863	3.3230	4.4500	3.7699
5.1735	5.6038	4.9426	3.8006	5.1131	7.2032	3.8626	5.0235	4.6700	6.0698	4.5399
7.2894	7.6204	5.1157	7.1252	5.7633	8.9789	6.0370	5.8514	5.1099	6.8847	5.9907
-0.2866	-0.3046	-2.4546	2.5706	1.7464	-0.5007	-3.2210	-2.3450	-2.8082	-0.3808	-2.0872
7.4184	6.6275	6.3679	4.7702	7.3475	7.6996	7.0459	5.5261	-7.6169	5.2341	5.2502
7.3485	5.5329	5.0379	4.0311	4.6393	6.1520	6.3117	3.2894	5.5113	2.3660	5.4682
3.0022	1.5539	1.8084	2.9980	4.1040	2.1706	1.2952	-0.0643	0.8480	2.3153	1.6419
-0.0547	-0.7538	0.2787	0.4891	-1.7292	-0.2395	0.9495	-1.4599	0.4236	-2.0019	-1.1447
-1.0340	-1.3894	-0.2219	0.1860	-1.9046	-0.2359	-0.3374	-2.3371	0.3264	-2.5206	-1.2033
2.6136	1.7755	0.2467	2.5285	0.0491	3.7458	3.4677	1.8174	3.1311	0.4482	1.8290
-5.1297	-1.8236	1.8114	-4.1391	-3.8905	-3.4946	-6.0395	1.0630	-7.6169	-4.0553	-3.5413
-3.5871	-3.1070	-0.4315	-3.6082	2.1149	2.7434	-2.1840	-0.7173	0.6136	2.0398	-3.4147
-4.4683	1.2677	-9.9616	-5.0277	-6.9391	-6.4021	-6.8470	-5.7220	-7.6169	-3.5528	-7.0767
3.5583	3.2501	2.8641	3.5294	1.2857	4.1421	5.7713	3.0791	4.9184	1.0547	3.5358
6.4630	6.8963	5.4517	4.3979	5.0853	6.5057	3.6137	6.3721	4.5843	4.1754	6.6138
-1.5046	-0.6899	-2.9612	-1.6485	2.0171	-0.9057	-3.3050	-1.2365	-2.9910	1.0114	-0.6083
-0.8793	-0.3825	0.5659	0.2489	0.3843	1.3850	0.3374	0.8659	0.2467	0.1851	1.2525
-5.9541	-4.5919	0.8971	-1.3573	-2.9397	-1.8998	-6.5551	-1.9286	-7.6169	-1.8634	-4.3533
-1.6450	-0.0663	-1.5731	-3.1924	-0.6449	-0.3307	-1.5963	-0.8801	-2.4912	0.4130	-1.9697
10.3775	10.7946	5.5333	8.9692	7.2816	11.9626	9.9749	10.8770	9.6442	8.9062	9.3390
10.6589	10.7697	6.0175	8.5640	8.8404	11.5166	9.8029	11.5912	9.4545	9.7578	9.1142
1.9445	-4.7150	-0.9056	-5.6019	-2.8381	-4.5473	-5.9109	-4.4529	-7.6169	-3.1617	-0.2365
0.0574	-1.8201	-0.8764	1.2232	0.5962	0.6445	-1.2426	5.3176	-1.7487	0.8790	2.6975
-6.4441	-0.1011	-9.8381	-7.5608	-6.9391	-6.4021	-7.7219	-4.0948	-7.6169	-5.1518	-7.0767
6.2619	3.7738	2.8578	4.5639	4.2591	6.5718	4.7693	5.4558	5.1167	4.3334	3.5928
-7.6866	-7.7221	-9.9616	-8.9048	-6.9391	-3.6274	-7.7219	-8.0854	-7.6169	-5.1518	-6.2345
-7.6866	-7.7221	-9.9616	-8.6200	-6.9391	-6.4021	-7.7219	-8.0854	-7.6169	-5.1518	-7.0767
9.9431	10.1315	8.2967	8.7387	9.7482	9.5536	8.7061	8.4624	8.6643	9.5023	9.4208
-7.6866	-2.2586	-9.9616	-6.5630	-6.9391	-6.4021	-7.7219	-3.9322	-7.6169	-5.1518	-7.0767
5.9104	5.8437	3.7310	5.3993	4.6056	6.9426	4.4567	4.9215	4.7777	3.5703	4.3472
-7.6866	-6.8323	-9.9616	-7.3317	-6.9391	-4.5466	-7.5669	-7.2898	-7.6169	-5.1518	-5.4824
1.9693	2.7523	0.2219	2.0468	1.1384	4.8682	0.9773	2.0580	-0.2467	1.8938	0.8357
5.2450	6.4051	2.9522	4.4971	5.0165	6.5135	4.8293	3.7928	4.4016	5.5098	4.5292
0.0547	-1.2710	-1.7718	-0.1860	-1.3846	1.2798	-1.1398	-1.4629	-1.3822	-1.1941	-1.2767
6.1134	6.0959	4.7298	5.1696	4.7182	7.6610	3.3957	5.8579	4.0540	6.2398	4.5551
7.3833	7.0342	6.4239	4.9224	4.2262	2.6398	4.3037	0.6187	4.2848	6.1239	3.3985
0.9907	-1.2153	-1.2258	-8.9048	-0.9813	2.1762	2.3181	1.1082	2.5079	-5.1518	1.0374
6.1978	4.6664	3.9561	7.3090	4.0101	8.4828	6.4163	3.0274	6.5471	3.6393	5.8083
4.7261	2.9427	2.6703	3.8240	2.3277	4.8674	2.8992	2.7253	3.2986	2.6519	1.8119
7.9978	8.5880	5.9065	9.4418	6.1088	9.3006	5.9172	8.8888	5.7903	6.1612	6.3588
-7.6866	-7.3839	-9.9616	-8.9048	-6.9391	-6.4021	-7.7219	-8.0854	-7.3223	-5.1518	-7.0767
-7.6866	0.0663	-9.3734	-7.1705	-6.9391	-6.4021	-7.7219	-2.8078	-7.6169	-5.1518	-7.0767
-3.6254	-0.5240	-9.9616	-3.9645	-6.3171	-3.3200	-6.2540	-3.8156	-5.1150	-5.1518	-5.7208
-7.6866	-4.3239	-9.9406	-8.9048	-6.9391	-6.4021	-1.8478	-5.8567	-7.6169	-5.1518	-7.0767
-7.6866	-3.0903	-9.9536	-8.9048	-6.9391	-6.4021	-6.9163	-8.0854	-7.6169	-5.1518	-7.0767
-3.5741	-0.5716	-6.6930	-4.8533	-6.2875	-1.6929	-0.4657	-1.4679	-0.2885	-1.1335	0.2365
6.2826	5.7871	5.0824	5.1534	5.1482	6.8632	5.1830	4.8602	5.4641	5.1335	4.8304
3.6927	5.1775	4.0970	4.5908	4.7575	6.4465	5.0750	5.3120	5.5328	5.4233	5.4236
8.7515	8.5252	0.9311	10.7132	7.4110	11.9409	11.1152	8.9709	11.6845	6.8794	11.5497
9.6331	8.0458	5.5396	9.8943	5.6544	10.6713	9.2406	6.2583	9.8795	5.7168	6.7361
1.6803	5.3885	2.6566	-1.2723	-0.0491	0.2359	1.9983	2.8583	2.2189	-0.5881	0.3701
7.1410	6.5583	6.3434	5.4625	6.9893	8.7171	5.9053	6.6676	6.5641	6.4321	7.0117

Table S4. Normalised Ct values for plates A and B.

21	22	23	24	25	26	27	28	29	30	31
-3.7219	3.2385	0.2737	-4.3831	-0.5829	-5.4163	-0.0890	-3.4597	-9.0274	1.7067	-2.4035
-7.3559	-1.9961	-3.9341	-7.1173	-4.6797	-8.5307	-4.0093	-7.3698	-8.9045	-2.0070	-5.9478
6.1883	-5.5552	-5.5536	-4.6694	4.3142	2.4875	5.5101	3.1617	-3.8759	4.6604	4.8352
3.4042	4.3577	3.1638	3.1491	2.9487	3.3031	3.7417	2.7667	3.2942	3.8348	4.3517
4.8124	5.8786	4.9096	4.4543	4.9357	4.3431	4.8111	4.2456	4.6735	3.2626	5.3612
1.9147	1.2873	0.6027	0.9040	1.1407	-0.1307	1.2957	0.1371	0.2765	-0.9314	0.3219
5.0295	6.2292	4.5658	6.6560	4.8486	4.4926	5.1479	4.3764	4.9520	3.7000	4.0483
6.3397	6.4095	6.7921	3.5387	6.5087	5.7225	6.5839	5.2620	6.2568	5.4839	6.1095
6.4820	4.7569	4.6217	3.6554	5.3181	4.4148	4.7561	4.5769	3.0511	3.4535	4.3400
6.2801	6.4484	7.5106	6.7900	6.5002	6.4625	6.4270	6.4432	4.4586	7.1453	8.0349
5.2467	5.9700	4.8357	5.2959	5.4612	3.2488	4.9017	5.3532	4.4031	4.5741	5.0355
-0.4667	0.7883	0.2688	0.5728	-0.3554	-0.6396	0.4554	-0.0231	0.3666	-0.5679	-0.0323
5.4137	4.5444	4.9380	5.3768	3.9665	3.2459	4.3787	3.6702	4.6039	3.2966	3.1823
6.8712	6.3664	6.5440	4.6484	5.9655	5.5775	5.5523	5.3687	7.0580	4.4269	5.8473
4.0813	4.9432	5.4569	3.8612	3.6521	3.6282	3.3187	3.2234	4.8981	2.5889	4.1901
3.9090	6.0373	4.0701	5.2760	4.7132	2.2172	5.4968	3.7420	4.0669	3.4503	4.7532
1.7963	0.5090	1.1030	-0.2837	0.6819	0.1307	1.6861	0.2638	-0.2765	0.0636	0.8776
-0.4288	0.5955	0.9429	0.8025	-0.4240	-0.9130	0.0890	-0.7314	-1.2860	0.2704	0.7923
4.5392	4.7966	6.3806	4.5314	4.3016	4.4160	5.1045	2.5480	4.3581	3.0082	4.4283
2.5075	3.5644	5.0289	0.2837	2.3842	2.5740	2.4544	1.1892	2.3110	1.4664	3.3115
0.9221	0.5505	0.2160	1.3907	-0.1987	-0.3615	-0.1486	-0.2836	-0.9234	0.2111	0.2258
6.4936	6.3420	6.4998	7.0706	5.4143	5.5571	6.3309	4.7011	5.3431	4.4511	5.4478
5.5766	5.5652	5.3557	4.1955	4.5352	4.1520	4.8440	4.5696	4.7461	3.5788	4.4325
3.5120	4.2594	4.2325	3.5447	2.4932	3.2630	3.6307	2.7313	3.4026	1.6588	2.9287
3.9446	2.5198	3.9398	3.1232	2.9771	2.4352	3.6893	1.8873	2.6563	1.4980	2.8165
1.2096	0.6066	-0.2506	0.7881	1.1471	1.8232	2.6790	1.4285	1.9663	1.7639	1.6555
-12.8132	-11.1172	-7.0550	-9.4017	-4.4089	-9.4169	-11.3351	-6.5336	-9.9198	-10.3582	-12.8153
8.2027	4.4434	6.9102	5.5439	6.4329	6.3886	6.8797	6.3548	4.4966	5.8084	7.0922
-10.3063	-7.0924	-5.3884	-9.4017	-12.4851	-9.6344	-11.3351	-9.4093	-9.9198	-10.1546	-9.8027
-0.7640	0.8997	0.9679	0.4381	-0.9721	1.1668	-0.8855	0.3798	-0.2874	-0.0636	0.1521
1.1356	-4.5703	-0.3048	2.6758	-4.7265	1.1169	-0.4828	1.1471	5.8489	1.3088	-1.3148
0.9200	-3.9686	-0.2160	2.2639	-1.6580	1.6026	0.2095	0.9502	6.5048	1.4698	-0.9526
3.2755	6.5200	5.0034	4.6671	2.9213	3.9445	2.9962	3.8982	4.2590	3.0579	4.5723
4.7712	4.7263	4.1713	4.7986	3.9804	3.9071	4.0865	3.8259	2.6296	4.1834	3.8141
-2.9725	-0.7653	-1.2509	-1.1640	-3.2164	-1.4798	-3.6096	-2.7584	-3.0761	-2.0071	-2.2094
-1.6455	0.4184	-1.4693	-1.2089	-3.6297	-3.5967	-3.6178	-3.9016	1.8069	-1.0554	-0.2939
1.3092	3.8355	3.2927	2.9826	2.7699	2.1772	1.6008	1.9402	5.1914	-0.5491	-1.9479
0.8349	-11.1172	-10.4933	-5.5618	-5.6175	1.3666	-11.3351	0.0231	-4.2760	0.4424	-1.5456
0.0058	-2.5297	2.6487	4.0788	3.1544	1.9060	-1.0742	1.4361	1.0038	-0.6168	0.0322
-7.4541	-11.1172	-10.4933	-9.4017	-11.4754	-10.4731	-11.3351	-11.0638	-4.1062	-9.5928	-8.0115
-2.8126	-2.6017	-3.2079	-3.5155	-3.0265	-2.0886	-3.5923	-2.3577	-2.6942	-2.9996	-3.2136
-4.5201	-3.8366	-4.2852	-4.0151	-4.5561	-2.0647	-5.0312	-4.4743	-2.9957	-4.5148	-5.3751
-8.8988	-8.0163	-10.4770	-8.9920	-8.4408	-8.2926	-8.8648	-8.6884	-4.3468	-8.4727	-7.4429
0.2459	-0.6915	4.0608	4.7860	4.4465	2.4863	0.5680	2.1489	4.3596	-0.9742	1.0192
0.8137	2.6751	3.0127	3.4116	1.9945	0.3225	1.6407	1.6527	3.6735	0.5667	2.1393
-2.4073	-2.1677	-0.9645	-1.1769	-3.5823	-2.9717	-3.3298	-3.0326	-1.7467	-3.2917	-1.9105
-0.0058	-3.6632	-0.7469	-0.9293	0.9239	-1.8867	-0.1480	-0.7414	-5.6959	-7.2199	-2.8293
-0.4776	-2.5858	-0.7272	-2.2491	0.1987	-1.9928	-0.6931	-1.0753	-4.8632	-6.3496	-3.0007
-5.4767	-6.0843	-3.6735	-5.6223	-4.7514	-4.3904	-1.7049	-6.9321	-4.2175	-3.5135	-7.0842
3.6985	4.4414	4.3136	3.9461	2.0949	1.7190	3.1978	2.1451	2.7992	2.0418	3.0081
3.6182	4.3662	3.9897	3.9419	3.6347	3.2188	4.0920	3.3121	4.2077	3.0655	4.5158
-1.9198	1.4434	-1.4491	-0.3799	0.3980	-1.2124	-4.5736	-3.1076	2.2081	-0.0782	-1.9452
5.6370	3.7390	5.3597	5.9999	4.5025	4.5324	4.4264	5.1277	7.4240	4.7795	3.4157
-12.1776	-8.6073	-10.4933	-9.4017	-12.7547	-10.6655	-11.3351	-10.1247	-7.2393	-9.1470	-9.6941
-0.9724	-0.4184	-0.2279	-3.0063	-2.3345	-1.2222	1.2410	-4.1131	4.0155	3.0082	1.2857
3.0416	5.6274	8.3235	2.9432	2.6536	1.6534	1.7464	2.4492	4.0387	1.4967	3.2934
-5.4834	-3.8559	-1.5749	-1.7457	-6.5138	-4.4778	-6.6403	-7.3981	-4.2884	-3.9082	-4.1387
-1.8625	-2.4219	-0.9838	-2.9050	-0.6720	-1.8190	-1.5702	-3.6578	-1.3286	-1.3806	-0.0322
-3.2481	-6.2765	-6.2216	-5.9978	-7.9900	-5.5947	-5.3870	-6.4600	3.0656	-5.9485	-8.3924
-2.1374	-6.2668	-7.3164	-5.9978	-8.8917	-5.0353	-5.3870	-6.4600	3.0422	-8.3582	-5.3624
7.1511	4.0990	4.8499	5.7987	5.8355	7.4753	7.6618	8.4855	0.4923	6.4551	5.5975
12.7878	7.9368	8.1681	11.8821	12.1568	12.2981	14.2676	12.7259	11.3763	10.3448	9.7742
10.7860	6.7463	7.2575	10.7903	9.4887	11.2486	12.1094	11.8729	9.4562	8.4201	8.5456
7.2831	3.2883	2.7486	6.5029	6.7229	7.4387	7.6325	6.8774	6.0178	5.3788	4.3589
-3.1752	-0.0059	1.0174	-2.0690	-3.0909	-0.8524	-1.9960	-0.0038	-5.4854	-3.1559	-4.3861
-7.9519	-5.7890	-4.7477	-5.9978	-8.8917	-8.1748	-5.3870	-6.4600	-8.6298	-8.3582	-8.3924
-5.8633	-7.7928	-6.3703	-3.1678	-2.7388	-7.7269	-5.3870	-2.6793	-6.1378	-8.1530	-5.9973

Table S4. Normalised Ct values for plates A and B.

2.0727	1.4550	3.7463	2.4727	2.1552	2.5429	3.2022	3.2967	-0.4126	1.7245	2.0066
-5.0004	-0.4236	-7.2702	-2.0465	-4.5776	-5.1722	-4.8679	-2.7185	-7.6916	-0.8822	-0.6980
-2.9445	-0.3031	-1.5623	-3.3835	0.1731	-2.0846	-1.1883	-1.6734	-4.3290	-1.6088	-3.4812
-1.0778	-1.1801	0.1185	2.8595	-1.1535	0.1621	1.5943	0.8055	2.4782	2.1074	1.7974
4.8371	3.9889	5.1394	2.8224	3.1936	3.3636	5.0014	5.1840	0.4126	2.8941	2.2006
6.1800	5.2876	4.2816	5.9742	6.1698	6.2203	5.5650	6.7327	4.5198	4.6043	6.0052
7.3529	6.4366	4.7724	6.9556	6.4834	6.9172	6.8050	8.0822	3.8333	5.4793	6.1105
0.0125	-1.8331	-0.0371	-1.1694	-0.8841	2.9581	0.6361	-0.3718	-2.1555	1.4718	0.2891
-7.9519	6.4300	5.1365	8.4954	6.9936	7.5033	7.0458	7.9379	5.9422	5.6367	5.5936
4.6224	5.8878	3.3331	7.4871	5.6753	5.1724	6.6205	6.7751	4.2782	4.0086	3.8458
1.8798	0.6546	-8.0691	3.5594	1.3855	4.7075	2.0709	2.1518	0.4702	3.3467	1.1260
-0.3603	0.3913	0.0371	0.1944	-3.6006	-0.1621	1.1116	0.4066	-2.9166	-0.6220	0.3100
-0.0857	0.3257	-0.5769	-1.4527	-3.6262	-1.1888	0.3620	-0.1924	-2.9559	-0.6027	-0.1285
1.9504	2.6035	1.2463	2.1540	0.9341	0.8256	3.2205	2.5694	-0.6537	2.0803	1.8267
-4.5420	-2.2018	-5.5133	-1.0676	1.1710	-4.0520	-0.5544	-5.5147	-2.8266	-2.3983	-4.4985
1.2155	-1.0389	0.5552	3.7383	-1.1940	-1.5408	-3.9759	-0.3983	-0.4633	-0.4443	0.3492
-7.9519	-4.9176	-8.0691	-4.6496	-2.8837	-7.4344	-5.3870	-5.1293	-7.4032	-8.3582	-8.3415
4.6839	1.2489	2.9104	3.4403	0.5999	5.7949	6.0156	5.0908	1.1312	3.4077	2.7972
6.2455	2.1329	1.6022	6.3078	5.6262	6.3657	7.4875	6.6559	5.0332	5.0110	4.6886
-2.0910	-3.2035	-3.3578	-1.8779	-1.5668	2.9292	-2.3110	-0.5733	-2.0188	1.3093	-0.9117
0.9004	0.0059	1.0294	-0.0102	-0.5423	-0.8877	2.1648	1.1867	-2.7558	-0.6840	-0.7733
-3.2482	-5.3426	-8.0691	-5.9978	-5.5333	0.5639	-2.7087	-2.4941	-3.1679	-1.3620	-5.5562
-2.0351	-1.7866	-2.3732	0.0102	-0.1731	-0.3866	-1.3270	-0.4685	-2.5807	-3.3756	-2.5294
8.9738	9.0389	6.7237	10.7399	9.9587	9.4321	9.0462	11.3690	8.1239	7.7615	9.2046
9.5299	8.7142	5.8216	11.1034	9.5598	9.5355	4.3727	10.7542	8.0035	7.4794	8.6446
-7.9519	1.2015	-8.0691	-0.1274	-8.8917	-2.3261	1.8028	-6.4600	3.3785	2.5205	-4.8021
1.0773	1.6315	-0.2245	-0.2304	0.8777	1.2323	-0.3620	-0.1141	-1.8030	0.1946	0.1285
-4.2075	-6.8943	-8.0691	-5.9978	-8.8917	-6.6739	-5.3870	-6.4600	2.1402	-8.3582	-7.1619
3.2285	5.7018	4.5750	5.2876	4.5164	5.9982	5.2209	6.6319	1.8618	4.0381	4.9119
-5.9607	-7.0976	-8.0691	-5.9978	-7.5982	-9.3495	-5.3870	-6.4600	-6.8486	-8.2428	-8.3924
-7.9519	-8.6821	-8.0691	-4.4945	-8.4107	-9.3495	-5.3870	-6.4600	-8.6298	-8.3582	-8.3924
10.0020	7.2779	7.5228	9.8671	10.2499	9.8504	8.9279	10.4255	6.2370	8.1635	8.3945
-4.1433	-6.4689	-8.0691	-4.3955	-6.7196	-6.7831	-5.3870	-6.4600	1.2501	-8.3582	-6.9038
3.9659	4.7322	4.2804	5.5083	4.8853	5.8807	4.6684	6.1202	2.9379	4.1755	4.3035
-7.7510	-8.6821	-8.0691	-4.3338	-6.5707	-6.5963	-5.3870	-6.4600	-8.5696	-6.3867	-7.5435
1.3605	1.7682	0.1222	3.2567	1.1780	1.5882	-1.4072	3.9882	-1.5048	1.6471	1.0492
5.1095	4.8623	1.9977	5.1695	4.3579	5.6830	5.5692	6.7243	3.1159	3.7346	4.6567
0.0816	-0.6399	-1.5177	-0.3659	-1.1746	-0.4650	-1.1262	0.3968	-2.6255	-2.0723	-1.2456
5.5550	5.2999	4.2433	6.5366	4.5723	6.0481	5.5108	6.9302	2.2828	4.0906	4.5079
6.2128	3.1350	3.6001	7.9477	0.1998	5.2261	3.7931	5.2941	5.3115	4.8996	1.3823
2.1844	2.6276	0.1566	1.0347	1.8720	-1.0376	-2.2511	0.0038	-0.9342	-0.1467	2.3989
7.1687	5.1257	5.1307	5.0233	6.5030	5.6232	7.1127	6.0844	2.2809	4.8893	6.7192
2.8161	3.0731	2.3272	4.3893	2.2329	4.1882	6.8850	3.1264	1.0543	2.4140	3.1646
6.6933	7.0803	7.5397	7.6978	6.1497	8.0730	7.1904	8.5633	5.3757	6.3491	5.8213
-7.0997	-8.6821	-8.0691	-5.9978	-8.8917	-7.2702	-5.3870	-6.4600	-8.6298	-8.3582	-8.3924
-4.5291	-8.6821	-8.0691	-5.9978	-8.8917	-7.1419	-5.3870	-6.4600	3.4445	-8.3582	-8.3924
-3.6043	-3.6522	-5.0713	-4.5677	-8.0577	-5.3365	-3.0681	-6.4600	1.3390	-4.5673	-4.5339
-7.9519	-8.6821	-8.0691	-5.9978	-8.8917	-7.4559	-5.3870	-6.4600	-7.9187	-8.3582	-8.3924
-7.9519	-8.6821	-8.0691	-5.9978	-6.5790	-9.3495	-5.2221	-6.4600	-8.6298	-8.3582	-7.0434
-0.0125	-1.8756	-2.5276	-0.8175	0.3905	-2.4551	-1.8975	-1.4038	-1.7933	-0.3480	-0.2814
6.1646	4.1549	5.1364	5.9498	6.0633	6.3487	4.6727	6.2701	2.4772	5.2948	6.1683
5.1610	5.4587	4.3016	5.2941	5.0180	4.5472	4.6610	4.9224	5.6045	5.5221	5.2466
10.7019	7.0241	5.9126	9.5251	11.2172	9.4133	11.3818	10.8210	6.1628	10.6232	11.0748
9.8064	7.5154	5.2058	7.0157	7.2192	9.7040	7.2620	11.1983	4.5324	5.4549	9.3068
0.9666	-1.0472	0.7737	2.4640	2.3781	2.4906	1.4181	3.6789	1.4729	0.1467	-0.1896
6.8639	6.0894	5.5859	7.1656	6.0724	6.8405	6.6752	7.8662	4.2931	5.9575	6.6781

Table S4. Normalised Ct values for plates A and B.

32	33	34	35	36	37	38	39	40	41	42
2.6571	0.8859	-6.7138	-0.9090	-5.4674	-6.0142	-6.4592	-8.3431	-6.9863	-1.1467	-0.9132
-1.3749	-1.9061	-6.3578	-5.0679	-9.2207	-9.3799	-11.0988	-11.1500	-9.9752	-4.0786	-4.8973
4.1368	-5.8159	-2.1910	4.2267	3.4300	1.8383	-5.7548	-8.7457	3.9705	4.3833	-6.3431
3.3527	3.5547	2.9044	3.2735	2.6684	3.4328	6.0342	3.8286	3.0067	3.5135	2.8901
4.8316	4.6827	6.8901	5.3246	4.9640	4.9299	4.7850	4.4934	4.9332	4.4408	3.5139
0.3815	2.2651	4.9573	0.0366	0.5453	1.9566	2.2297	0.8694	-0.3216	0.4582	-0.8588
5.8018	6.7312	5.4999	4.6584	4.8116	7.0069	4.5175	5.4192	4.5954	5.3104	4.3200
6.4206	8.4148	4.2632	6.0873	6.7836	1.6717	3.0138	5.3160	5.2700	5.3289	6.2117
5.1693	7.9961	7.9526	4.8277	6.0196	1.8880	3.6797	5.4060	4.8405	4.0063	3.4995
6.0789	7.3197	7.3855	6.2204	4.8370	4.6091	8.2409	6.4468	3.5805	7.0675	7.9805
4.5451	6.2121	4.1565	5.2956	3.5017	2.9946	4.3120	6.6508	4.0831	4.9093	4.6638
-0.0885	0.3843	3.6897	0.3273	-0.4536	1.3500	0.2430	0.6256	-0.8974	0.7728	-0.0620
3.6674	4.9561	8.4713	5.0216	4.3057	5.1150	5.3048	4.6156	3.5581	4.3770	3.3605
4.5813	7.2144	7.7118	5.4129	4.9725	5.5178	3.9615	6.1138	4.8870	6.5337	5.3516
2.7932	5.5574	6.1792	3.5960	2.9571	4.1517	3.7187	4.6972	3.1476	5.2488	2.9560
3.7649	5.2112	0.0842	4.5820	2.4257	3.6861	2.5520	4.2594	2.3334	4.2620	3.5713
0.5425	0.8720	2.0392	0.5442	0.4736	0.9891	0.3046	2.0976	0.6123	-0.0106	0.7842
-0.2596	0.8982	2.6060	-0.6403	-1.2468	-1.6697	1.4834	-0.0706	-0.0573	-0.3708	-0.1820
4.7593	6.3596	6.2450	2.0985	2.7810	4.2357	6.3644	4.1470	4.0434	4.9213	5.3181
3.3237	4.7633	5.3927	0.8898	1.6566	-2.6556	3.0949	3.1133	3.0918	3.8860	2.6825
0.4912	1.0040	-0.9040	-0.0727	-0.1514	0.3041	-1.3238	-0.0312	-2.0856	0.0665	-0.6163
5.8416	6.4815	8.0193	5.7747	5.5224	6.9619	7.1647	6.1513	4.7073	6.2592	4.7189
4.8647	5.6272	7.9325	4.1754	4.4768	3.2874	4.2864	5.3863	3.2238	5.3248	4.0454
3.5172	3.4036	4.7598	3.6581	3.2343	4.0363	3.9999	4.0741	1.8265	3.5786	1.9793
2.2417	4.1260	0.3914	2.5853	2.2429	3.5455	4.5556	2.7026	1.4352	2.8665	2.3604
3.0000	-0.2165	0.3001	0.9594	3.3203	-2.6807	0.0912	0.6494	1.6414	0.2376	1.5313
-8.7588	-11.6148	-7.8220	-13.6073	-12.6594	-10.0447	-11.0988	-5.3353	-10.3193	-11.5668	-10.9013
7.9563	9.3273	9.9412	5.4537	8.0298	4.3926	5.8837	6.7440	6.4952	6.1877	5.4694
-9.4995	-8.3188	-7.8220	-12.5962	-12.6594	-6.2952	-7.3769	-11.1500	-9.7550	-7.6158	-10.0729
0.3930	0.0705	-0.5191	-0.7943	-0.8261	-0.0027	0.5161	1.0374	0.0573	0.0106	0.6089
-0.1354	0.6072	-2.0526	0.4788	1.9986	2.9749	-1.2700	-1.7926	0.1740	-1.1646	3.5289
-0.4921	-0.0705	3.9268	1.2320	1.3925	3.0251	0.2565	-2.2022	0.4953	0.3232	3.7386
4.3956	2.9514	7.4937	3.6677	2.4319	5.3086	5.6710	5.7543	2.9091	5.8014	3.7644
4.8213	5.5395	-0.0842	4.0794	4.9683	4.7642	2.8243	4.9578	3.5721	4.4571	3.7300
-2.2782	-3.4557	-5.1371	-2.0540	-2.8963	-2.3881	-4.2053	-2.1441	-3.1501	-2.2359	-2.5014
-0.2082	-1.0882	-5.3392	-3.3631	-2.3538	-1.2771	-5.9267	-3.6307	-3.6385	-1.5429	-1.6851
2.0518	4.2079	4.1978	1.2121	1.3026	3.7398	0.9190	-3.0067	0.9780	-0.5193	0.9145
-11.0124	-8.9074	-7.8220	-10.2424	1.6946	-5.1392	-9.6668	-3.1427	1.0109	-9.8999	2.3045
-0.2328	1.9417	10.4647	-2.5446	0.9280	3.1595	4.9278	5.5905	0.0859	1.0951	-1.2109
-11.6801	-11.6148	-7.8220	-13.6073	-10.5575	-10.0447	-11.0988	-10.3555	-6.4767	-11.5668	-7.5689
-3.1432	-3.1554	-0.3798	-2.8746	-3.2179	-5.4153	-1.5485	1.2047	-3.9258	-3.6580	-1.3668
-4.1416	-4.7297	-0.1438	-3.4539	-4.9246	-7.5599	-3.5412	-0.3822	-6.3402	-5.2441	-1.8069
-8.0759	-6.3763	-7.8220	-9.5601	-8.0419	-10.0447	-10.6642	-6.2137	-10.0794	-7.6305	-7.3650
1.6278	3.6200	4.5474	-0.5486	-0.2026	4.5869	4.9430	2.9623	0.8412	0.8648	2.8752
0.0089	2.0882	3.5235	1.2022	2.3732	3.6882	2.3671	2.9670	1.2415	2.0496	2.2143
-2.1680	-1.3355	2.4491	-2.3937	-3.1543	-1.2322	-0.0912	0.9563	-3.9174	-0.7684	-2.2963
0.3545	-2.9882	-2.7976	0.8861	0.1514	-3.2870	-4.1786	-9.0231	-0.1988	-3.3611	-6.2386
-0.0088	-2.5417	-4.9139	-0.0366	-0.4806	-3.7507	-3.9124	-11.1500	-0.3129	-3.8501	-5.2937
-4.3613	-5.9535	-4.9401	1.7060	-2.8799	-6.1637	-3.4165	-6.8740	-0.3670	-5.7776	-8.9096
2.7897	3.7408	4.9186	2.4888	2.1021	3.9928	3.8019	4.0014	1.2194	3.6601	2.4036
3.1138	4.2868	0.9124	3.1850	2.9944	4.1907	4.1783	3.5274	2.5105	3.9127	4.0558
-1.2026	-0.8319	-0.0868	-4.5599	-1.8861	0.0027	-3.6610	0.0865	-3.1700	-2.6479	0.1178
4.5176	6.7347	10.3175	5.4828	4.8566	6.2793	7.2197	6.3777	4.3068	4.2347	4.9832
-8.4651	-10.5846	-7.8220	-11.8227	-10.4787	-10.0447	-11.0988	-11.1500	-9.9244	-10.9699	-11.9491
0.2927	-0.2825	0.1466	0.6618	0.5684	-1.8401	0.9681	-3.4091	0.1207	-1.5618	0.9412
2.4839	7.1604	7.0264	1.8907	1.1529	3.9099	5.9814	3.1424	2.6454	6.3870	2.6694
-6.5663	-2.4450	-0.3256	-8.0887	-6.2910	2.0486	-0.6538	-4.1305	-7.1069	-2.9315	-7.7828
-0.9370	-2.2436	1.1357	-1.2640	-1.3297	-2.2211	-2.6434	-5.0176	-4.4398	0.6103	-2.6115
-6.4505	-6.4578	-5.4130	-7.8286	-7.9453	-7.5647	-7.3884	-6.3772	-3.3596	-4.4142	-3.0270
-6.4505	-5.6842	-5.4130	-7.8286	-7.9453	-8.7211	-7.3884	-6.3772	-2.6867	-4.1041	-5.1271
8.1832	4.2691	9.3144	8.7367	7.0731	5.3177	8.2802	7.2020	8.0328	6.8847	5.8800
11.0382	8.6168	9.3878	13.2403	11.7430	9.0326	12.7252	12.3704	11.1432	10.1619	12.1497
8.5780	6.4394	13.7018	12.2254	10.0748	7.8714	12.0710	11.4105	10.6559	8.4406	10.4531
5.7224	3.6873	5.3982	8.2237	6.4489	4.2738	6.9840	6.3830	7.0890	4.9264	6.0051
-2.5736	-3.2126	-0.9380	-2.9217	-4.4704	-0.0716	-1.8542	-4.5546	-1.7418	0.0225	-5.7369
-6.4505	-9.0110	-5.4130	-7.8286	-7.9453	-5.8944	-7.3884	-6.3772	-8.3041	-6.8658	-8.0011
-6.4505	-6.0252	-5.4130	-4.1504	-3.0985	-5.9319	-7.3884	-6.3772	-5.9691	-6.8658	-3.8835

Table S4. Normalised Ct values for plates A and B.

2.8188	0.9287	1.9138	2.7173	1.6910	0.9121	3.0134	2.6383	2.0638	1.8361	1.5865
-3.5793	-4.2488	-0.7237	-5.1822	-7.4178	-4.5274	-4.6829	-4.5581	-6.5833	0.6285	-7.0503
-4.0152	-3.4678	-1.9744	-1.0468	-2.9290	-4.3496	-3.0118	-1.6416	-0.8087	-3.6860	-4.0287
0.6827	0.3670	0.5960	-0.4024	-0.4923	0.3533	2.1542	1.9660	1.6008	2.2154	1.7189
5.3510	3.1188	4.7711	4.6964	4.5319	3.5340	5.3339	5.0532	4.4608	4.4103	1.5306
4.8055	4.9344	7.6478	7.2454	5.7543	4.8714	5.7251	5.5955	7.7887	5.7189	5.1241
7.3174	4.9688	8.8595	7.0831	6.2021	4.9453	7.8314	6.9619	5.4773	7.0888	5.8687
-1.3502	-0.8611	0.4286	0.0752	0.6132	1.0069	-2.7601	-0.4426	-2.0219	-0.7306	-1.4427
6.5507	5.2743	9.5004	9.1052	6.5016	5.6868	8.6065	10.1149	5.8334	3.5802	8.4437
7.4466	3.6969	3.9245	8.1189	4.8124	3.6070	5.5214	8.6564	4.8862	3.6128	7.5212
1.6742	0.7466	2.2002	3.1666	2.3583	3.1264	1.8784	4.7492	1.0638	1.9928	2.4736
1.5939	-0.3670	0.6283	-0.6355	-3.4144	-0.0939	1.5872	0.0151	0.7258	-0.1634	0.8870
1.2161	-1.5027	0.2846	-2.2849	-2.9370	-0.6494	0.2824	-0.5862	-0.1304	-0.7900	-0.6359
3.1031	0.9381	3.6611	1.7835	1.4695	1.8481	3.7204	2.7874	1.4327	2.9692	1.5481
-6.4505	-5.4008	-5.4130	-3.1434	-2.0965	-5.1125	-2.4403	-4.5927	-4.9063	-4.0259	-0.8113
-0.3489	0.3673	1.6282	1.3876	-0.4884	-0.1808	-0.8818	1.9050	-0.9995	-2.9247	-8.0011
-6.4505	-4.3966	-5.4130	-7.0673	-5.3610	-8.7211	-7.3884	-6.1173	-8.3041	-6.8658	-8.0011
4.4835	0.5600	1.1566	5.6381	3.2925	0.6396	5.3053	2.0378	5.7815	2.3329	4.2434
7.7617	2.8185	6.5778	8.1807	6.5859	2.3010	6.4382	6.0990	5.5635	4.4716	6.8623
-6.4505	-2.2443	3.9441	-1.0442	0.8170	0.4132	-1.7945	-1.6318	-1.0140	-0.8747	-1.2214
1.6114	0.4011	-1.6459	1.0892	0.8092	0.0716	2.4216	0.7774	0.1304	-0.0225	-1.0354
-6.4505	-9.0110	-1.1805	-4.6889	-2.5640	-2.2070	-2.6728	-3.5913	-5.1046	-4.7189	-8.0011
-3.4256	-1.8337	-1.1881	-0.1767	-1.7346	-2.6917	-0.3659	-1.1587	-2.0137	-1.8156	-4.2991
8.5513	7.3812	6.7764	9.2095	8.1372	8.3292	7.7237	9.4445	6.8645	10.3094	9.6090
8.4521	6.3874	9.5976	9.1637	8.8210	9.1381	9.4865	9.2289	7.9496	9.5824	8.2594
-3.2131	-5.9425	-1.6234	-0.2161	-3.9778	-3.0075	-7.3884	-6.3772	-1.6271	-6.8658	6.5476
-0.7583	1.9991	1.7954	0.8178	0.3387	-0.1200	2.2895	-0.0151	0.8523	2.0201	-0.0580
-6.4505	-7.2637	-5.4130	-7.8286	-7.6753	-8.0927	-7.3884	-6.3772	-3.3294	-6.8658	-5.1941
4.9366	0.8356	-0.6116	5.6623	4.3610	4.0011	4.6264	5.3469	4.4790	4.9316	4.7162
-5.1325	-9.0110	-5.4130	-7.2857	-7.9453	-8.6589	-7.3884	-5.6679	-8.3041	-6.8658	-8.0011
-6.4505	-9.0110	-5.4130	-7.8286	-7.8486	-8.6032	-7.3884	-6.3772	-8.3041	-6.8658	-7.6017
9.3665	8.3668	9.5913	9.1047	8.5324	7.3555	9.7426	11.1537	8.9625	8.7644	8.7397
-6.4505	-6.2343	-5.4130	-7.8286	-7.9453	-8.7211	-7.1462	-6.3772	-4.0617	-5.3574	-5.4843
6.0070	2.4020	6.7187	6.0076	4.0230	3.9063	5.9929	7.0527	4.2260	5.3529	5.1021
-6.4505	-3.9520	-5.4130	-7.7066	-7.0812	-6.7271	-7.3884	-5.3102	-6.9282	-6.1214	-6.0017
3.0058	-1.1186	-0.2846	2.2682	0.0633	1.5744	0.3894	3.9245	0.9332	2.8792	1.8413
4.7349	3.6526	7.3573	5.0418	3.5807	3.6315	4.9443	6.0881	4.5967	5.3309	4.0518
-1.3252	-1.3844	0.9850	-0.0752	-0.6790	-1.1216	-0.3184	0.4097	-0.5201	1.4293	-2.9561
4.3611	2.6908	5.6264	6.8067	4.4395	3.7842	6.1190	6.7279	4.4652	6.4210	4.3163
4.2938	6.3363	5.3135	3.7374	4.8899	5.6751	5.4867	7.0568	3.6655	2.2329	5.3400
0.3489	1.0689	-5.4130	-0.1827	-0.0633	1.6611	-0.0010	-2.3337	0.5282	1.1339	0.7697
6.7407	4.0641	5.6053	6.7036	6.5486	4.2025	5.2739	4.5049	5.5735	7.5848	4.0311
4.1930	0.6548	4.5631	4.4750	2.5252	2.7786	4.8032	3.9529	2.5832	3.6253	3.5289
7.7626	6.9926	7.1792	7.7093	5.0979	7.4392	10.2959	7.0518	6.7698	9.0754	7.3829
-6.4505	-9.0110	-5.4130	-6.1138	-7.9453	-8.7211	-7.3884	-6.3772	-8.3041	-6.8658	-8.0011
-6.4505	-7.0908	-5.4130	-7.8286	-7.9453	-8.7211	-7.3884	-6.3772	-4.4441	-6.8658	-4.1810
-6.4505	-2.9337	-5.4130	-4.7201	-4.3198	-3.7820	-7.3884	-6.3772	-4.0789	-3.3896	-6.5420
-6.1491	-2.2171	-5.4130	-0.9523	-7.9453	-8.1177	-7.3884	-6.3772	-8.3041	-6.8658	-8.0011
-6.4505	-8.5138	-5.4130	-7.0095	-7.9453	-5.8819	-7.3884	-6.3772	-8.3041	-6.8658	-7.4338
-2.6079	0.9316	-2.1852	0.6602	0.5046	-4.7116	-4.0819	-1.3861	-5.5331	-3.0209	0.0580
5.8548	4.2783	6.3004	6.5454	5.1872	3.2179	6.5514	5.7215	5.1639	4.9470	5.9943
4.4801	6.2830	2.4453	4.0373	4.1315	5.6835	4.7268	5.1352	6.1683	5.7016	4.4351
11.2998	10.0364	7.4137	10.0016	9.8619	6.7070	8.5031	9.1205	12.3806	9.8538	7.8296
10.3504	5.8060	6.9035	10.2673	8.9980	4.7690	9.7744	9.5779	11.2056	10.1792	7.3480
1.6948	1.0086	-0.7830	1.3022	1.1982	0.3559	0.0010	-2.5590	3.2252	-0.7536	1.4282
6.2972	4.7470	6.7344	6.5638	6.6404	5.8516	8.8090	7.9767	8.5066	6.7903	6.5389

Table S4. Normalised Ct values for plates A and B.

43	44	45	46	47	48	49	50	51	52	53
-6.1372	-3.6806	-1.9384	-1.9495	-1.5038	-2.2792	-1.2668	-7.6579	-6.5761	-0.8411	-1.7472
-9.4143	-8.0281	-6.8823	-5.3026	-4.8307	-7.6482	-5.7508	-12.4394	-9.4294	-5.7289	-5.8743
1.1200	0.5341	3.5063	4.2750	4.6755	4.5680	4.6404	2.7194	4.0843	5.0521	3.9166
2.0156	4.2490	3.0088	2.5830	3.1586	4.2069	3.4443	2.1963	2.8229	4.7491	2.0500
4.3712	5.5898	6.0539	5.5438	4.4901	4.9644	5.8753	4.4855	5.6274	5.0019	5.0285
0.8956	1.6572	2.7388	0.2524	0.1984	1.7586	1.7311	0.2981	0.5534	1.3487	-0.5075
5.6211	6.6466	7.4063	5.4035	4.9080	7.0350	6.5193	5.4313	4.8040	7.3970	4.7436
5.5783	7.2336	6.5682	6.5760	6.4158	5.0028	7.3883	6.0325	5.5569	4.1791	5.7452
3.7373	4.9517	3.9332	5.2607	4.4460	3.6549	5.4781	5.3048	5.6585	4.0698	4.1388
5.1362	5.9873	4.3171	3.1139	8.0999	6.7071	6.5837	4.8389	5.8040	9.3325	3.9464
3.8040	3.8767	2.1087	4.2712	5.5816	5.4475	4.3741	4.5630	4.4140	6.2031	3.0239
-0.7542	-0.0671	0.3639	0.0296	-0.0968	1.1130	-0.4116	-0.2972	-0.0248	1.6667	-0.6215
3.0003	5.6599	5.6731	3.4038	4.1822	4.8224	4.2481	3.9798	3.5001	4.5763	3.3180
4.6487	6.5617	7.1389	4.2223	4.7633	5.4575	5.4887	4.6358	5.6734	4.6602	4.4148
3.7485	6.1569	5.9429	2.7727	4.1655	3.2274	3.8388	2.9903	3.9148	2.8075	2.4367
3.7867	4.6753	3.9374	3.4726	4.2163	6.3405	4.2124	2.7866	3.3791	6.0358	1.5010
-0.8436	2.7428	-0.8038	0.1981	0.1537	-0.5548	-0.1347	1.0165	0.9016	0.3356	0.0620
-3.5977	0.7791	-0.3506	-0.9352	0.6582	0.1312	-0.0164	0.1495	-0.4838	1.1797	-1.1821
0.7603	4.5247	3.0164	4.7373	4.9922	5.3785	4.4886	4.4758	4.7959	4.9720	4.1180
0.0630	2.6903	2.0940	3.2280	3.8754	-0.3207	2.6876	3.1571	3.8702	1.0513	2.3438
-1.0042	-1.5362	-0.5760	-0.5551	0.2078	0.6697	0.1718	-0.5840	-0.8235	1.5344	-0.8721
5.5310	6.1046	6.2039	4.1893	4.9136	7.8690	5.4898	4.5489	4.8129	6.2149	5.0378
3.9428	5.1298	5.3572	3.8304	4.2945	4.5659	4.7986	3.9886	4.3578	4.1535	3.8848
3.6555	4.7612	4.8211	1.8785	2.8440	4.5768	3.3407	2.4100	2.7260	3.1087	3.1243
2.0646	3.7618	3.7336	1.2137	2.0314	3.8922	2.0384	1.7619	1.8855	2.9854	1.4091
-0.0630	-0.9340	-0.0023	3.0389	1.9244	0.4960	0.5523	1.6740	1.2782	2.6641	1.6665
-7.6424	-11.5700	-10.6514	-12.8956	-11.5079	-9.5842	-11.5414	-12.4394	-8.3068	-11.3709	-10.7186
6.3788	5.2350	5.8187	6.5758	5.4332	5.1516	5.7739	7.2278	7.4228	5.4617	6.6435
-11.8986	-11.5700	-10.2200	-8.6383	-9.4037	-8.7039	-9.8301	-9.7863	-4.4690	-9.1925	-4.3915
0.7215	0.7978	1.5552	-0.1943	0.0968	-0.1312	-0.3652	0.4591	-0.0492	0.6682	-0.9545
1.0114	2.4138	1.2680	0.5748	-2.5927	2.8653	1.1494	0.8695	1.8287	-3.2968	0.5801
0.8187	2.5636	1.1935	1.5057	-0.8852	2.5847	1.6361	0.5640	0.8110	-0.6071	0.5661
3.3948	6.3176	6.4752	3.1122	4.3708	4.8381	3.9170	4.1141	2.9515	5.3000	2.7720
4.8298	6.1600	5.4214	4.5183	4.2191	4.7336	4.8373	5.2684	4.0281	4.9443	4.2642
-2.8085	-0.0689	-0.7164	-1.7958	-2.3769	-3.4106	-2.4048	-2.2066	-2.8977	-1.6924	-2.8630
-5.1496	-0.3319	1.1476	-2.9586	-2.8685	-3.1205	-1.1137	-0.4901	-2.4414	-0.8505	1.2182
3.1400	3.8023	1.8969	-3.5570	0.1261	1.8361	-2.3014	2.1391	-0.6284	-2.2540	0.6293
0.6659	-5.7934	-7.1732	0.1852	-11.5079	-8.5612	-11.5414	-0.5207	0.9259	-3.5755	0.5319
1.3185	7.4676	2.3525	1.0586	-0.3260	1.9187	1.5547	4.2216	3.6780	-3.0538	2.1151
-11.8986	-11.5700	-10.6514	-11.3993	-11.5079	-9.5842	-11.5414	-5.7753	-5.2106	-9.5207	-10.2547
-2.8724	-4.2932	-4.6475	-3.0996	-3.0087	-3.5538	-3.3035	-1.3528	-3.2269	-2.6933	-3.5108
-3.8545	-5.3775	-6.0736	-4.4428	-3.9959	-5.3716	-4.3014	-1.6709	-4.5520	-5.2816	-5.3723
-10.3162	-7.0949	-8.5754	-8.0694	-8.2550	-8.1597	-9.8245	-7.3973	-9.0095	-11.3709	-8.7368
4.3328	4.3479	1.9813	3.0819	-0.4466	4.5542	2.9696	2.9431	0.4941	-2.0345	3.4243
1.8209	2.5569	3.7679	1.8330	1.6325	3.0897	2.7568	1.8488	2.0815	3.1584	1.2182
-4.4777	-0.4632	-0.4136	-2.5993	-1.0582	-1.8147	-1.4478	-3.1969	-1.9994	-0.3356	-2.8502
0.1911	-2.0469	-3.7779	-1.2513	-6.8958	1.3194	-0.1663	-1.4400	-0.1188	-5.1958	-0.0620
0.5506	-1.0480	-2.9679	-1.7221	-5.2773	-0.1336	0.0164	-1.5493	-0.0848	-6.2859	-0.1839
-4.8797	-3.4827	-6.5542	-4.2498	-8.2522	-5.7333	-2.7268	-5.1380	-4.8173	-7.8551	-3.5591
2.0222	3.8919	4.0670	1.4468	3.4859	4.2784	2.9726	1.9771	2.2092	4.1522	1.1204
3.8050	4.9689	4.5909	2.8837	3.2226	5.0259	3.1707	2.6878	2.8813	5.1011	2.5219
-5.1016	0.0671	-0.1634	0.7757	0.2239	-2.1946	-0.6499	-2.4200	0.0248	1.2613	0.7643
3.9915	6.3969	4.6012	2.7993	2.8896	4.9630	4.8402	4.0023	4.9822	4.0842	4.2326
-11.8986	-6.0015	-9.6773	-12.7323	-8.9109	-6.3504	-9.3988	-5.2703	-10.6011	-9.4746	-12.0114
-5.1652	-3.4674	-0.1762	-2.6685	2.3257	-1.1796	0.5919	-4.2428	-0.2712	4.3857	-1.3053
1.7008	5.5494	4.3803	2.2145	5.2274	2.6403	1.1269	0.8527	6.1963	3.1602	2.5999
-7.8635	-2.9347	-0.0692	-7.0214	-2.3326	-3.5880	-4.1568	-0.9191	-1.0172	-1.6681	-3.7439
-2.9062	-1.3424	-0.1554	-3.2329	1.4294	-0.5329	0.0422	-3.3636	-2.4686	1.6424	-3.3389
-6.3603	-5.9892	-8.0325	-6.8239	-4.0666	-6.3397	-8.1573	-1.2818	-2.0616	-5.6892	-5.7613
-6.3603	-7.2849	-7.2546	-4.5231	-3.7829	-6.1053	-5.0347	-1.1787	-2.4022	-5.5109	-4.3279
6.8097	6.7947	6.3750	9.4103	6.6045	6.0309	6.6167	9.7604	6.5060	4.9372	6.5026
12.3006	12.2268	9.1517	11.5577	8.6933	11.3020	9.9186	7.7425	10.4240	8.3856	10.0226
11.0295	11.3019	8.3625	10.7501	7.5803	9.3858	8.5261	7.6962	8.6588	6.6293	8.2465
7.0442	7.2653	4.6550	6.5357	3.2498	6.3454	5.0990	3.7213	4.8423	2.6497	4.7122
-5.7426	-3.5321	-1.8621	-0.5344	-2.2004	-1.9723	-2.0001	-1.3191	1.6961	-5.0636	1.8658
-6.3603	-7.2849	-8.0325	-6.8239	-6.8454	-7.6668	-8.0855	-6.3864	-4.1100	-9.0311	-3.6772
-6.3603	-4.2731	-7.1104	-5.5355	-6.8426	-4.4092	-6.6328	-3.8262	-4.7838	-4.4833	-4.1625

Table S4. Normalised Ct values for plates A and B.

2.1376	3.9646	-0.2689	3.2836	3.1533	1.6454	1.5082	3.0015	2.8097	2.6992	1.9788
-6.3603	-6.7218	-2.2589	-4.7081	-0.7415	-5.0728	-1.8957	-6.3864	-4.3107	-0.4621	-5.9678
-1.4969	-3.8281	-1.9655	-0.5153	-4.3304	-4.4729	-2.2855	-0.2003	-2.1759	-3.8218	-2.4902
1.1781	3.4120	2.4532	-0.0052	3.1447	0.8723	1.9348	-0.5709	-1.5675	2.9587	-0.1198
3.6676	3.8247	2.8164	4.2608	3.1659	2.5858	3.0793	4.1792	4.7641	2.0007	2.8760
5.9417	6.7720	6.8772	6.5167	4.6353	4.8929	5.9546	5.4977	5.8567	4.3371	4.7308
7.7007	8.1763	5.8831	6.5109	6.4094	6.0030	5.9905	7.4011	5.0359	6.4502	4.8915
-2.0596	-1.3664	0.9792	-0.8146	0.2659	-1.0683	0.5561	-2.8722	3.0001	-0.7556	2.7810
5.2084	7.8248	5.2101	7.9417	6.1320	5.2308	6.3009	7.6304	-7.9993	4.4653	7.2362
5.2898	4.9767	3.4800	7.0489	5.0530	4.1610	5.2174	6.3974	4.8426	3.2943	6.3420
1.0828	0.0639	3.0396	2.2794	0.6727	0.3806	2.2069	1.5453	3.8589	0.0588	4.7261
-1.6907	-3.0144	-0.4617	0.3848	-0.0964	0.8513	-0.6888	-0.2144	-0.9305	0.7502	-3.0927
-5.3732	-1.9117	0.1525	-0.2571	-0.6095	-0.2134	-0.4198	-2.4050	-0.9788	-0.3277	-2.4014
1.8189	2.1906	2.8313	1.6008	2.1878	1.2690	1.2564	2.2450	1.0243	2.3055	-0.6316
-2.6106	-2.4420	-4.4605	-3.9623	-5.9967	-6.1953	-8.1573	-3.8241	-6.4576	-4.7769	-7.9461
-0.0713	1.8853	0.9573	-0.1621	-0.7245	1.9952	0.5549	-0.1519	0.7880	0.6306	-1.6852
-6.3603	-7.2849	-8.0325	-3.2752	-6.8454	-5.6117	-8.1573	-6.3864	-7.9993	-4.7219	-7.9461
4.8499	3.2397	2.9737	2.3294	4.2101	4.5193	1.3116	4.1773	3.5835	4.9528	1.8917
7.2987	4.5592	3.5631	5.5005	3.6134	6.1082	4.0153	2.0410	5.2503	3.5449	4.3805
-1.4830	-3.1189	1.8760	-0.5627	-2.1407	-2.4025	0.4198	0.6556	-0.1564	-1.2246	2.8994
-0.0891	-2.3517	-0.9627	0.5315	0.0964	-0.9916	-0.4581	1.6160	1.1037	0.1737	-0.2080
-3.9365	-4.1517	-0.1525	-3.3613	-4.6523	-7.0668	-1.8440	-6.3864	-2.4945	-3.2476	1.0448
0.3523	-0.0639	-1.1715	-0.0169	-1.8040	-2.2809	-1.2248	-0.0458	-2.1642	-2.4001	-1.9254
10.9270	10.0904	8.4191	9.7124	9.3754	9.4333	8.9996	9.4417	7.9231	9.4331	8.1708
10.9687	10.4319	9.8726	9.8742	8.5809	9.2420	8.8659	9.1556	8.4297	9.0565	7.5564
-6.3603	0.5788	-0.3305	-6.8239	-4.6863	-4.6081	-3.7167	-3.9782	-2.0592	-9.0311	-1.2284
0.6767	2.2244	2.4356	-0.1396	0.6906	-0.9863	1.3771	2.6984	2.6174	-0.0588	0.1198
-6.3603	-5.7714	-5.5184	-6.8239	-6.5042	-5.4957	-7.5534	-1.6093	-2.8082	-7.7389	-5.1146
6.3741	6.4237	4.3051	6.5088	5.6499	5.2246	4.7845	5.8326	4.1172	4.9044	3.6016
-6.3603	-7.2849	-5.1397	-6.8239	-6.8454	-7.6668	-7.0211	-5.4664	-7.9993	-9.0311	-6.6445
-6.3473	-7.2849	-8.0325	-6.8239	-6.8454	-7.6028	-8.1573	-5.3506	-7.9993	-9.0001	-7.9461
10.8736	10.5217	8.1974	9.3749	8.3244	8.1448	7.0101	9.9547	8.0645	7.7701	9.1366
-6.3603	-7.2849	-5.0740	-6.8239	-5.9398	-7.6668	-7.7876	-3.0468	-2.6874	-6.4702	-7.9461
5.7274	4.7048	3.8781	5.8323	4.9501	4.3066	4.2217	6.1421	4.0843	4.4633	4.1173
-6.3603	-5.8010	-6.4130	-6.0952	-5.7317	-6.9242	-8.1573	-6.3864	-4.6529	-6.2778	-5.8509
1.8407	1.7985	1.3502	2.4796	2.1467	2.1604	1.0855	2.2989	0.2099	1.9767	0.6688
5.5752	5.0682	5.2868	5.4413	4.7139	4.3725	3.9574	5.4029	3.7363	4.1024	3.8310
0.0384	-0.1112	-0.8454	0.5819	-0.8924	-0.6722	-1.9081	-0.1507	-0.3008	-0.4648	-2.7416
6.3513	5.9940	5.4373	6.0671	4.3737	4.7085	4.0968	6.4686	4.5120	4.6426	4.4426
0.3393	8.2002	5.1179	1.4518	3.0716	5.9682	3.8789	7.0752	3.2398	2.4217	4.1449
2.0095	2.8692	-0.1816	1.3557	3.5018	2.3977	0.9147	1.8420	0.9168	3.0906	0.6137
6.5069	5.3823	6.7709	7.3498	5.6127	4.8716	6.4243	7.1758	5.2226	6.8820	5.8431
4.4000	4.2365	2.4815	2.3727	3.1043	3.4531	2.6979	1.8796	2.2629	3.7592	1.5278
6.9994	7.7813	6.3492	6.6454	7.0845	6.8746	5.8901	7.5459	8.3368	6.7534	6.8806
-6.3603	-7.2849	-8.0325	-6.8239	-6.8454	-7.6668	-8.1573	-6.3864	-7.9993	-9.0311	-7.9461
-6.3603	-7.2849	-4.5374	-6.8239	-6.8454	-7.4808	-8.1573	-2.2628	-4.0686	-4.8500	-7.9461
-6.3603	-3.2536	-2.1962	-4.8313	-6.8454	-4.9457	-4.1233	-0.7685	-1.2416	-4.2363	-4.3118
-6.3603	-1.9843	-8.0325	-6.8239	-1.3091	-7.6668	-8.1573	-6.3864	-7.9993	-9.0311	-7.9461
-6.3603	-7.1763	-8.0325	-6.6453	-6.8454	-7.6668	-7.9383	-6.3864	-7.9993	-9.0311	-7.0876
-0.0384	1.2554	-0.5769	0.7689	0.3662	0.2134	-1.5611	-0.3645	-0.4447	0.8742	-0.6299
5.7093	3.7201	4.3046	7.0317	4.5518	4.8244	3.8855	6.4123	4.9411	5.1474	4.2013
5.3018	6.5196	8.6129	5.2729	4.9879	4.7778	5.8831	4.8029	4.7532	6.7635	4.1667
9.8489	5.6199	10.9985	12.3242	10.4681	9.2841	9.9917	11.1951	10.9652	11.0381	9.7188
8.0319	6.9399	5.3783	10.7643	6.2863	6.8531	8.4203	10.5972	9.9649	5.7454	6.0691
5.1847	-0.3476	0.5768	0.0052	-0.5003	0.8266	-1.9937	3.0168	0.1564	-1.3055	0.9957
7.5414	7.8031	7.7654	7.2245	6.5505	6.1595	6.0585	8.3571	6.6138	6.1926	4.9754

Table S4. Normalised Ct values for plates A and B.

54	55	56	57	58	59	60	61	62	63	64	65
0.7231	-5.1137	-7.5934	1.7389	-0.2210	-3.1624	-0.4862	-1.9508	-6.2827	1.0966	-0.9378	1.1988
-2.7602	-11.5556	-9.0646	-2.0557	-4.7661	-7.3094	-4.6570	-4.2693	-9.9830	-3.1251	-4.0446	-3.3000
-5.8343	1.7686	-7.6886	4.7192	4.0917	-3.5041	4.7909	4.8654	-8.5881	5.2236	5.5341	2.2993
6.0129	1.9304	3.2568	4.7280	2.0821	3.4497	2.8820	3.1436	3.2524	3.7754	4.2758	5.3325
5.2058	5.3444	4.2698	4.7553	5.2166	6.6386	5.1472	4.9333	4.3752	5.3210	5.7424	5.1874
-0.4154	0.3710	2.6392	-0.0696	1.2403	3.7932	0.1170	-0.1833	0.5999	1.1434	0.5596	0.0308
3.1369	5.7721	8.2319	4.4359	5.6372	-9.7756	4.5597	4.1970	5.2473	5.5011	5.3436	5.1617
5.4414	4.9092	3.6417	5.4256	6.5078	6.6791	6.3586	5.7679	6.6052	6.3679	5.3977	5.7553
3.9878	4.0438	4.2274	5.2712	4.6720	4.4337	4.9587	4.0550	4.9945	4.7713	5.6246	3.8998
9.5484	3.9735	6.7576	8.2462	5.4631	6.2710	5.3790	7.8037	6.8615	7.5983	6.9266	7.1772
6.1766	3.1017	5.0203	4.4817	4.1148	6.4386	4.0412	4.9192	3.0006	5.2970	4.7047	4.6450
0.6862	0.2632	0.0382	0.2033	3.0593	1.2506	-0.5817	0.6811	0.1166	0.3994	0.2290	0.3628
4.4807	3.4439	6.2919	2.7537	3.5061	5.6912	4.6993	3.8550	4.7712	3.8472	3.4749	2.9226
5.2257	5.1665	6.3563	5.7912	5.3133	8.2233	5.0460	6.4418	6.3204	4.7397	5.4582	4.5431
5.1102	3.1230	5.0438	4.9112	3.7511	7.1103	2.9921	4.3572	4.0292	3.7489	3.8710	4.0958
4.0199	2.4729	5.8447	3.2453	3.6532	6.0842	3.8292	4.1736	1.7841	4.7606	2.7787	4.4547
1.5661	0.2810	0.5885	0.8422	2.8222	0.0755	0.9714	1.4376	0.9243	1.4184	0.7039	1.7932
2.6058	-0.7862	-1.4679	1.0757	-0.8742	-0.6982	-0.3044	1.4592	0.1306	0.3268	0.7125	1.5799
5.3470	3.2856	5.5403	4.9694	4.9513	4.4166	3.9091	5.9110	6.0117	4.3376	4.6879	4.2196
4.3670	1.0263	0.5255	3.7029	3.4984	2.9571	2.5513	3.9679	3.5715	3.1333	2.7526	2.2718
-0.6385	-1.0936	0.5334	-0.3927	-0.3210	1.3332	-0.8983	-0.1192	-0.3284	-0.3718	-0.1093	0.0558
6.3255	5.7727	7.9698	5.4337	5.1894	7.2848	4.6344	6.1220	6.7447	5.1908	4.9446	5.8749
5.6757	4.5232	4.9661	4.4386	3.9327	6.7304	4.1419	5.7426	5.3413	4.2153	4.5715	5.0712
3.7805	3.0779	5.7774	3.1112	3.1367	5.6032	2.6179	4.2701	3.9592	3.1999	3.0494	3.7170
2.2721	1.0050	3.9849	2.0394	2.2849	3.8626	1.3826	2.1777	3.4337	2.5097	1.6224	2.0090
4.8866	1.8585	1.1613	3.3439	0.5011	0.4830	0.8652	3.5332	2.8983	0.2614	2.9806	3.9343
-10.9872	-8.9510	-8.4943	-11.6198	-11.4335	-9.7756	-12.0755	-11.4543	-11.7313	-12.6768	-13.5070	-10.2632
6.6646	6.9630	5.3022	5.4613	4.4340	5.5067	7.5038	7.0605	7.0725	6.0281	7.3069	6.3383
-10.9872	-10.1984	-8.3444	-9.5145	-8.1945	-9.7756	-10.2881	-10.0176	-8.8797	-7.9574	-9.3957	-8.6373
1.9629	-0.2632	2.5455	1.0470	-1.7186	2.4738	-1.2901	-0.0353	-0.1166	0.0031	0.1684	1.3656
-4.0933	1.5747	-2.6765	-3.6206	0.2709	3.2367	2.2546	-1.3053	1.2309	-1.0934	-0.4674	-4.8457
-2.7570	1.5055	-2.1494	-1.8918	0.2299	3.1050	2.8035	-0.9262	0.7954	-0.0371	-0.0199	-4.0746
6.2230	4.4936	6.4553	4.8222	3.0042	7.7051	2.7349	4.7808	4.3149	4.3375	3.8625	5.8506
2.5074	4.0783	5.1833	3.6596	3.9335	5.9826	4.1688	3.5197	4.3799	4.4288	4.1697	4.0789
-0.3968	-2.5463	-0.6656	-1.3158	-3.6300	1.1729	-3.2777	-1.8019	-2.4939	-1.8292	-2.3898	-0.3568
-5.6999	-3.2271	-0.1874	-2.1286	0.2210	-9.7756	-1.0702	0.1528	-2.1334	-0.0031	-1.5191	-1.7482
3.6722	-8.7845	4.4540	0.0696	1.6759	-9.7756	2.8579	-0.9020	3.0192	-2.4421	0.5461	1.9872
-10.9872	-8.5518	-9.0646	-2.3921	-7.8447	-0.6581	1.7894	-1.6330	-8.0062	-8.6582	0.0199	-11.4457
-2.7754	1.9078	4.2259	2.4020	2.0641	5.1698	-1.2764	-2.2456	2.6255	0.0788	2.6816	-2.1902
-10.9872	-11.5556	-9.0646	-8.7990	-11.4335	-9.7756	-7.0663	-9.5128	-11.7313	-12.6768	-8.9312	-11.4457
-3.3737	-3.7348	-3.3426	-3.4286	-4.5616	-3.6576	-4.1006	-3.6619	-0.1682	-2.8150	-2.6514	-2.6783
-5.7787	-5.2530	-4.6290	-5.4310	-5.7056	-4.0716	-5.3347	-4.5437	-0.6250	-4.2726	-4.9507	-4.4167
-10.2977	-8.0429	-9.0646	-10.7748	-8.7885	-8.2302	-8.6998	-7.3995	-7.8124	-7.6808	-9.7979	-8.0875
-1.5005	3.6312	5.4899	-0.0826	3.2229	5.1848	2.4635	-1.1359	4.7131	1.2263	1.8354	-1.9115
2.6448	2.1782	4.7371	0.4687	1.4677	4.1680	1.9131	3.0356	3.4667	0.9405	1.1509	2.1055
1.2291	-2.8188	-0.0382	-2.2738	-1.9715	0.4392	-3.3209	1.0139	-1.9201	-1.0280	-1.1171	-1.7419
-8.4231	0.4496	-3.9937	-3.3706	-1.3763	-7.8357	-0.1170	-3.2024	-6.4955	-4.2779	-1.8421	-6.0016
-8.1282	0.9089	-3.9790	-3.4632	-2.0507	-6.2541	-0.9156	-4.0142	-7.1242	-3.8447	-1.4073	-5.3895
-9.0451	-2.6785	-5.8316	-5.4635	-3.7879	-3.6157	1.6120	-6.4177	-6.1574	-6.0571	-5.0346	-11.4457
4.0839	1.7373	4.3878	2.8818	2.5343	4.6616	1.8595	4.0177	3.0870	3.2965	1.9984	3.1976
4.0809	2.9627	5.0559	3.5194	2.8611	5.7267	1.7961	2.8829	3.9284	4.2515	1.6294	3.1711
0.2274	-1.5604	0.8215	-1.2890	-0.6276	-0.4978	-2.8433	0.7149	-2.6948	-0.4817	-0.8257	-3.3087
2.6541	4.2983	6.6263	2.6057	4.8412	6.4475	5.8285	4.7510	6.8722	3.0894	2.4787	0.3001
-7.9578	-10.1632	-9.0646	-10.6582	-7.5253	-8.2990	-8.7218	-11.4543	-10.1232	-8.8956	-11.1689	-8.1575
0.9962	-3.6333	-2.6052	0.9891	-1.8360	-4.0590	2.3770	0.5596	-2.5828	-0.0517	-0.9383	2.0589
3.6257	3.5660	3.1104	3.8979	5.3904	5.9993	3.4570	4.7323	4.4187	2.7928	3.5195	2.5302
-3.0280	-5.4116	-2.9563	-2.9935	-2.8773	-2.3649	-4.7487	-1.9075	-3.9203	-2.3674	-4.3150	-3.1057
1.2624	-4.6207	-2.4743	-0.0869	-1.4158	-0.0755	-3.6325	0.0353	-2.8617	-0.6613	-3.1180	-0.0566
-7.8625	-6.1896	-7.5529	-6.2471	-4.2367	-7.6624	-3.3142	-7.6445	-3.2445	-5.0687	-7.8038	-6.8143
-7.8625	-6.1896	-7.5529	-8.0345	-3.9987	-7.6624	-1.6125	-7.6445	-3.8370	-4.4901	-4.8156	-5.7563
4.7435	8.1252	6.0212	7.2945	5.8196	5.1969	8.3676	6.7620	7.4052	6.3367	8.6220	5.4872
8.7800	11.4363	11.8960	10.6110	10.0089	10.2960	11.8233	9.7077	13.1166	10.4952	10.6026	9.3800
8.5427	11.1674	11.0721	9.6483	8.1923	9.4263	10.6771	8.6070	11.6460	8.7378	9.9087	8.7305
2.6246	6.2313	6.9611	6.6814	4.6429	5.8069	6.7091	5.0715	7.9612	4.9577	5.3254	4.0406
-5.1818	-0.7428	-3.1496	-0.6415	-1.3236	-3.5227	-2.7478	-2.1263	-1.9843	-2.6502	-1.1848	-2.0589
-7.8625	-6.1896	-7.5529	-5.8308	-8.3045	-7.6624	-7.7267	-7.6445	-7.0960	-8.0824	-5.9749	-8.6070
-7.1957	-1.2449	-4.0027	-6.3761	-5.6353	-5.2848	-7.7267	-5.8251	-5.6308	-6.0270	-7.0177	-5.2411

Table S4. Normalised Ct values for plates A and B.

2.2239	1.9779	0.7599	2.9099	1.1319	0.8349	1.9315	2.9987	3.6728	3.0095	2.5138	2.0547
0.6364	-6.1896	-7.0695	-2.9645	-5.2909	-4.6220	-4.3254	-2.0982	-5.3469	-3.1052	-1.7082	0.9767
-4.0035	-1.8244	-3.1694	-2.7902	-3.8797	-3.8123	-1.3276	-4.4917	-3.0918	-3.4359	-3.6927	-3.0522
3.1339	0.9278	1.0132	2.1893	0.6336	3.3019	-0.2260	0.7816	0.7143	0.6818	-0.4796	2.9393
3.7088	3.2759	2.8028	3.4732	2.5661	2.7628	4.0660	3.1535	4.4181	3.1338	2.7605	2.6965
3.6021	4.1220	5.6722	8.4800	4.4009	6.5105	5.5973	5.2217	5.2403	5.2793	5.8819	5.0434
7.2359	5.6411	6.1200	7.9194	4.3347	6.3997	5.8193	7.5017	6.7756	7.0315	6.8929	5.8163
-4.8494	0.0318	-2.1817	3.6603	1.7232	-2.8344	0.0689	-1.7957	0.3213	-0.7824	0.7456	-2.5345
3.4542	8.3003	6.8000	-8.4700	6.1194	7.6517	7.4418	-7.6445	2.3148	5.5914	5.6147	5.4678
3.9585	7.6162	4.7067	4.9710	4.9945	6.4608	5.2006	4.2150	3.8892	4.9675	5.1240	4.9376
-2.2629	3.0327	2.5304	3.8400	2.7310	1.8895	1.9079	0.3221	1.9368	1.1081	1.4540	-0.0935
0.0717	0.2304	-1.4459	0.8827	-1.2350	-1.0547	-0.0689	-1.2835	0.1235	-0.0076	-1.2853	0.2288
-1.2589	-0.0318	-1.9895	1.2950	-1.4274	-0.9915	0.5410	0.2875	-0.5006	-0.5536	-0.0908	-0.2569
3.0590	1.3957	1.3364	3.0277	0.7894	2.2000	1.1190	3.4508	1.9150	2.7517	2.1580	1.9336
-6.2511	-6.1896	-3.7287	-4.5792	-3.1053	-2.3370	-1.7486	-4.6493	-6.5894	-8.0824	-4.4740	-5.1693
-4.0214	1.5171	1.5473	-1.4146	-0.0100	0.9065	-3.6956	2.9385	-3.1862	0.0076	0.7763	1.4091
-7.8625	-6.1896	-7.5529	-1.7743	-6.0056	-5.9488	-6.4076	-2.1908	-7.0960	-6.3372	-6.4312	-8.6070
5.3804	2.8934	1.9422	5.9095	0.5318	0.9014	4.0405	5.1978	5.2180	6.3349	3.0262	2.7016
3.6994	5.6791	5.9257	4.5200	4.1956	4.7612	6.6752	4.7525	7.2465	5.2549	4.9830	4.5295
-2.4540	-3.0391	0.1859	-1.3276	-3.2022	-1.8696	-1.4086	-0.7519	-0.6381	-1.5119	-0.0115	-1.6124
0.7155	0.0442	0.6921	-0.7914	-0.6805	-0.1047	0.7253	-0.4793	0.7204	0.3241	0.0115	-0.6368
-7.8625	-1.7262	-3.5555	-5.2463	-8.6839	-5.0676	-2.6132	-6.1649	-5.0877	-3.2667	-6.1534	-6.1405
-5.1802	-1.7192	-0.0705	-3.5642	-2.4545	-0.9816	-1.4899	-3.8914	-0.0108	-2.2973	-3.2734	-2.8537
9.0695	8.9847	9.3327	10.0386	7.9014	10.3571	6.6227	8.6835	10.2124	9.3611	8.2378	7.2982
8.8029	8.7642	10.2731	9.8124	7.6914	9.8948	7.3169	8.9345	10.5970	8.4400	8.9712	6.5054
-7.3721	-6.1896	-7.5529	-4.6540	-5.5206	2.0766	0.9048	-7.6445	-4.8717	-8.0824	-6.5741	-8.6070
-0.0327	-0.9009	-0.5281	-0.0737	0.5395	0.1047	1.8181	0.7471	0.0108	0.1397	0.1371	2.1168
-7.8625	-6.1896	-7.5529	-7.1727	-6.9700	-7.6624	-3.0382	-6.0580	-4.3514	-7.6013	-6.1528	-8.6070
4.2060	3.8401	4.3513	5.6973	2.9765	5.4998	3.5842	4.3707	4.9876	5.0208	4.0000	4.9401
-7.8625	-6.1896	-7.5529	-8.4700	-5.5035	-4.4059	-5.3464	-7.6445	-7.0960	-5.6141	-8.3773	-8.6070
-7.8625	-5.9275	-6.0122	-8.4700	-7.8264	-7.6624	-7.7267	-7.6445	-7.0960	-8.0824	-8.3773	-8.6070
7.6947	9.3788	9.1720	8.8924	6.8455	8.0142	8.1902	8.5917	10.2621	7.9074	9.0227	8.3754
-7.8625	-6.1896	-7.5529	-6.5575	-8.6839	-7.6624	-3.5445	-7.6445	-5.7110	-6.3200	-7.1657	-8.6070
4.2881	5.1550	4.9794	5.2353	2.5086	4.7535	4.0138	4.7417	5.4541	3.9932	4.1214	4.6035
-7.8625	-6.1896	-3.6760	-7.5332	-7.2416	-4.6631	-5.7648	-4.9628	-7.0960	-4.4377	-7.9272	-6.3712
1.3307	2.1306	2.2389	2.5420	0.0100	2.6606	0.5891	3.0874	1.7294	2.0544	1.6495	2.0412
4.5550	5.0943	5.8558	5.8810	3.4081	4.2308	4.1193	5.6825	4.8841	4.0676	3.6473	4.5702
0.0327	0.4419	0.0705	0.0737	-1.9573	-0.7910	-1.5751	1.3839	-0.4215	-1.3594	-0.6160	-1.8944
4.5665	6.1227	6.0716	5.8695	3.4835	5.3660	4.6291	5.4286	6.7298	4.1033	5.4604	3.5716
2.4991	-6.1896	6.4311	3.0585	4.3249	7.7650	4.1123	-0.1673	8.1240	4.0986	4.5966	3.0569
1.5073	0.3202	-2.4327	4.1511	0.4282	0.2836	-0.5225	1.0367	-0.4859	1.4148	1.8600	0.8547
5.5246	4.8185	3.3349	7.5010	4.4850	3.3663	5.6870	7.9017	4.3352	6.5020	7.3721	5.5499
2.5281	3.8159	2.9246	4.8476	1.0729	3.1484	3.5498	3.5402	4.3206	3.1003	2.7892	2.4252
5.5126	8.2972	6.0555	8.4631	6.8237	6.2468	6.8119	8.0200	8.5385	6.8964	6.9789	5.5445
-7.8625	-6.1896	-7.5529	-8.4700	-8.6839	-7.6624	-7.7267	-7.6445	-7.0960	-8.0824	-8.3773	-8.6070
-7.8625	-6.1896	-7.5529	-6.4544	-6.1382	-7.6624	-3.0764	-7.6445	-7.0960	-7.2193	-5.7588	-8.6070
-7.8625	-6.1896	-0.3379	-7.0635	-3.4947	-2.0121	-3.0966	-3.4382	-3.0649	-3.0764	-3.0082	-4.8926
-7.8625	-6.1896	-7.0332	-8.4700	-8.1953	-7.6624	-7.7267	-7.6445	-7.0960	-6.6863	-7.8048	-1.6320
-7.8625	-6.1896	-6.6390	-8.4700	-8.6839	-7.6624	-7.7267	-7.6445	-7.0960	-8.0824	-8.3773	-8.6070
-7.4380	-0.9626	-2.3279	-4.2249	0.1025	-0.9976	-1.9240	1.1208	-0.4964	0.9045	-3.9152	-1.6332
4.9480	4.0898	4.0845	5.8540	3.6572	4.6702	6.3765	5.4520	6.0872	4.9510	4.9396	3.4881
5.8258	3.8607	6.9564	6.1662	4.7336	7.6395	4.9135	4.9463	3.8748	5.1593	6.3249	5.1411
10.4942	9.1857	7.6395	12.0149	9.0902	6.5979	11.2569	10.3547	3.6653	11.3442	12.0674	9.8760
9.4229	7.4798	6.5536	11.3213	4.8701	5.9930	9.5700	9.1863	8.0121	6.5992	11.3836	5.7045
2.9799	-1.2903	1.4677	2.8064	0.2952	1.7768	2.7758	0.1673	3.4900	-0.5228	2.1764	0.0935
6.3852	5.9045	7.5410	8.9967	4.8844	7.3445	6.7627	6.4778	6.3946	6.6466	7.4673	6.1869

Table S4. Normalised Ct values for plates A and B.

66	67	68	69	70	71	72	73	74	75	76	77
-5.5063	-8.6090	-0.5600	-4.8864	-7.3465	-1.2975	-3.3983	-2.4630	-5.6374	-3.8356	-6.8913	-4.3218
-10.5252	-10.0188	-4.2656	-7.6071	-9.9746	-4.5698	-7.5911	-6.9548	-9.0610	-7.7317	-11.5216	-8.1906
-6.9522	-6.0037	5.2201	4.8983	-7.9482	4.6517	0.3050	4.7193	-6.3678	0.6104	-6.4068	-7.0484
2.6290	3.4948	3.1845	3.8612	4.2289	3.5731	2.3960	3.1214	3.2429	2.3258	2.1871	2.8371
4.6882	5.0324	4.8995	4.8211	4.6901	4.5433	3.9039	4.9185	4.3912	4.2258	4.4960	4.6763
1.3957	0.7602	-0.0596	1.5129	-0.0646	-0.0183	-1.4154	0.2181	-0.7880	-0.5730	-0.8539	0.4749
5.6191	5.8892	4.9605	5.5640	4.7986	4.2754	3.3236	5.1278	3.7844	3.4912	4.1896	4.7384
6.6600	6.2276	6.2642	6.3496	5.3377	6.2276	5.1719	5.4857	4.4635	4.5149	4.6858	5.2438
5.2653	5.6655	5.3444	5.9759	5.6399	4.9640	4.2144	4.0590	3.6137	3.7749	3.9992	4.0324
5.5648	6.0569	5.9524	6.0928	5.9910	5.7953	7.4362	5.6997	7.6394	5.5675	5.4239	6.5073
3.5546	4.9243	4.8063	5.4636	3.9409	4.6805	5.5564	4.6132	6.2489	4.5675	5.2992	4.6681
-0.1946	0.3651	-0.2964	0.6370	0.7586	-0.6758	0.0558	0.1886	0.0352	0.0423	0.1307	-0.2680
4.9012	4.8761	4.6986	4.4582	4.0895	4.0484	3.4634	3.7133	3.7959	2.8820	4.0949	3.8648
3.8307	6.3744	6.2317	5.4090	5.3989	5.7984	4.4186	5.2377	4.7081	4.2118	5.0869	4.6286
3.2450	4.9298	4.1500	4.5646	5.5521	4.1414	2.9395	3.2180	3.3344	2.7618	3.3326	3.1880
1.4346	3.8920	4.1797	4.6733	2.9420	3.8467	3.5892	4.1932	4.0751	2.9838	4.0464	3.5977
0.6489	1.3505	0.7125	1.1293	0.9477	0.0183	0.5443	1.0848	0.8054	1.7092	0.8210	0.4504
-0.2706	-0.0240	0.2380	0.1518	-0.3797	-1.0355	-0.9863	-0.3778	0.3026	-0.0207	-0.2597	0.0669
5.0782	5.8359	3.8083	4.9569	3.7517	3.1896	4.2414	3.7292	3.6849	4.9506	3.4226	4.2439
2.5955	4.4342	1.6003	3.3811	2.3282	2.3925	2.7445	2.7692	1.7756	3.2117	1.3818	3.0053
0.1946	0.5551	0.1123	0.0104	0.0256	-0.5609	-0.5084	-0.0881	0.4485	-0.3220	0.7508	0.5739
5.3287	6.1678	5.8863	6.1258	6.6809	4.9219	5.1557	5.4827	5.4374	5.0704	5.6600	5.7017
4.6583	4.6575	5.0363	5.1711	5.1580	3.8548	4.3601	4.3233	4.8689	4.3161	4.7524	4.9188
2.2264	4.3166	3.5819	3.5997	4.4650	1.9905	3.4178	2.7234	3.1514	2.8896	4.0693	3.1022
2.1989	3.3373	2.7358	3.1358	2.8200	3.3539	2.6060	2.1695	2.3735	1.9546	1.7671	1.5434
2.6290	2.3455	0.4817	0.2297	-0.2815	1.0377	1.0704	1.4925	0.1368	1.2561	0.7610	-0.1814
-9.8328	-8.6836	-11.8029	-12.2019	-9.3612	-11.3073	-12.2467	-9.7770	-12.6309	-6.5276	-11.5216	-12.1538
6.0905	6.7443	6.6577	6.8134	6.6290	5.7284	5.1360	6.9031	5.0303	6.0791	6.2420	6.6076
-3.7003	-10.5782	-11.8029	-10.1562	-4.5107	-11.3073	-5.0461	-11.3045	-11.7711	-7.3862	-9.9945	-9.2923
0.4253	0.0240	0.0596	0.4566	1.1385	-1.0767	-0.5357	-0.3748	-0.0352	-0.4932	-0.1307	-0.4546
-4.1471	-4.1672	0.8948	1.9955	0.3168	1.5859	0.4456	0.3972	0.6577	0.8074	1.2135	1.2078
-3.9921	-3.3714	0.2271	1.8844	0.0520	2.8168	-0.0558	0.7302	0.7559	0.9938	1.2930	0.7211
3.7721	4.8863	3.2239	4.5006	4.9637	2.5601	4.3383	3.2859	4.3560	4.3609	4.0707	4.4983
5.1950	5.2327	3.8059	5.0470	4.4022	3.6061	3.6499	4.3861	3.3936	3.7096	3.9998	3.8452
-2.5716	-2.0042	-3.9027	-1.9678	-1.2309	-3.7042	-1.8158	-3.1273	-1.4564	-1.6821	-1.1749	-1.6864
-2.8495	1.5617	-1.5980	-0.9528	-4.1476	-0.3350	0.2950	-4.5397	-1.8037	-0.1939	-2.8897	-2.8463
2.6742	2.6529	0.7120	-0.2626	-0.1919	1.9421	1.3213	3.0557	1.4671	2.8134	1.2082	1.7689
-11.3759	-10.5782	-0.5086	-9.2627	1.3196	1.8149	0.6075	0.7376	-0.0652	0.0207	0.6120	1.0460
-3.5967	6.5832	-0.8979	2.1591	4.4236	0.2947	2.0749	-0.7283	3.9886	1.4690	4.0582	2.9099
-11.3759	-8.6482	-10.3776	-12.2019	-6.9975	-9.3077	-5.2973	-10.9802	-8.1981	-4.2958	-7.8129	-10.5491
2.6447	-2.4740	-3.3342	-2.5085	1.4604	-3.3110	-2.8877	-3.1701	-2.6305	-3.6240	-2.5093	-4.0201
1.8498	-3.5328	-4.6354	-3.9942	1.4191	-4.5482	-4.8082	-4.5014	-4.3380	-5.7886	-4.0505	-5.1004
-8.1028	-7.7174	-7.9822	-9.7821	-9.7107	-8.0174	-8.6189	-8.1815	-8.7811	-8.7439	-6.8309	-8.5605
4.8232	6.0876	0.8649	-0.7901	5.1002	2.2026	2.3462	0.0881	2.3629	2.8773	4.0147	3.0101
4.5031	4.0497	1.7634	1.2934	2.2779	0.6605	1.4096	1.1938	1.5121	2.1103	2.2354	2.9496
-2.0847	-1.0849	-2.6550	-1.4507	-0.5578	-3.3123	-4.8896	-2.9337	-3.2367	-3.3141	-1.8702	-2.5494
-6.4754	-5.0774	-2.6686	-2.5143	-4.1973	-1.0337	-1.5238	-0.5962	-4.6208	-3.6318	-4.7917	-6.5572
-6.8067	-6.1000	-3.0600	-2.7486	-4.3510	-1.6953	-1.7624	-0.9467	-4.5516	-3.9752	-4.6465	-6.8163
-6.1522	-5.8525	-1.7704	-4.8535	-0.6614	2.1118	-6.4191	0.4219	-6.1467	-5.2953	-4.9045	-4.7705
2.6740	3.7344	3.0622	2.8645	2.6556	2.0071	2.5689	2.4767	2.6757	2.4648	2.6306	3.0404
3.6163	3.5697	4.1098	4.3706	4.3204	3.0386	3.5590	3.3122	3.2054	2.8939	3.4218	2.5152
-2.0630	0.6187	-4.7460	-0.0104	-3.5488	-4.2661	-4.5802	-3.3427	-2.9640	-1.4719	0.3420	-3.4820
3.4823	7.3147	5.5286	4.6094	6.0349	5.2497	3.1752	4.8974	4.5139	3.3819	4.9766	4.0430
-8.5912	-9.7439	-6.9375	-9.1740	-11.0761	-11.2014	-7.5929	-11.3235	-9.3325	-9.5502	-4.0300	-10.3898
4.7944	-1.8527	1.2993	-3.1783	-3.4074	0.2039	-1.4464	-1.1570	-3.0246	2.1757	-2.1837	-2.6535
4.0903	6.2777	2.5495	4.6316	2.1987	4.4670	3.5131	2.2678	3.5033	5.8328	3.1567	4.9210
0.9928	-2.8290	-7.4370	-1.7996	-5.5730	-5.1709	-0.8525	-7.4268	-3.9853	-1.9879	-4.0741	0.7609
-3.0440	-3.0770	-1.1911	-0.8700	-4.1830	-1.7211	-2.2118	-2.6850	-3.3581	-0.5403	-4.0401	-1.5898
-5.9606	-1.4027	-6.3215	-7.4286	-4.6968	-5.9468	-3.2618	-5.8662	-4.8818	-0.4272	-4.6132	-6.8622
-5.6177	-1.4058	-7.0186	-4.3945	-5.8934	-5.9468	-2.0665	-4.8656	-3.5386	0.0143	-4.9694	-4.4104
8.9160	6.3898	6.8421	5.8490	7.6293	6.8572	5.3508	9.4550	5.6739	4.9027	5.6645	6.7177
11.3314	10.9191	12.4231	11.7274	13.7267	13.2371	9.6645	11.9362	11.5027	9.1377	11.5863	11.1453
11.1882	10.0811	11.6592	10.6570	12.3569	10.9644	8.7645	10.9567	10.5448	8.6912	10.2952	10.3212
5.9662	4.9018	7.1550	7.3524	9.6122	8.1172	3.8806	5.9959	5.3804	3.7690	5.5139	5.8526
2.9820	-4.0751	-2.7225	-1.5289	3.3411	-4.2163	1.9071	-3.1515	-2.3562	-0.1414	-2.0518	-1.3689
-3.1969	-8.6268	-7.0186	-7.4286	-2.1229	-5.9468	-7.1309	-8.4976	-6.7825	-7.4726	-5.7892	-6.8622
-6.5449	-8.0323	-6.3492	-4.7075	-7.2265	-4.8379	-4.1228	-7.9108	-6.7825	-4.0229	-5.7892	-6.8622

Table S4. Normalised Ct values for plates A and B.

1.7103	1.8258	1.5875	2.8857	2.1196	2.1902	2.4658	1.7409	3.2669	1.8244	2.5936	3.3339
-3.3709	-5.6107	-3.2835	-5.9476	-3.2046	-3.9898	-0.7604	-4.6282	-2.5135	-2.3950	-5.7698	-3.7903
-2.0075	-4.6986	-0.8475	-0.3753	-1.6665	-1.4741	-3.1103	-2.7376	-2.5845	-2.9328	-5.7892	-5.1813
2.7993	0.8093	-0.5418	-1.1506	0.1902	0.0691	-0.0990	-0.7362	1.7962	1.2731	3.0881	1.3263
3.4746	2.2599	4.2777	3.7536	4.4405	4.7438	3.3691	3.8653	2.5522	3.3003	2.7531	3.7911
4.4486	3.5113	6.7710	6.6607	7.0047	6.6718	3.9031	5.3085	6.0753	3.6722	5.2558	5.8008
5.2995	5.7811	6.9743	6.8528	5.8536	6.0310	6.7468	6.4226	7.2759	5.5431	7.5469	7.6875
-2.9035	0.6130	-0.8648	2.5955	-0.6408	0.3081	0.2520	-1.6780	-0.9666	-3.3153	-0.7165	-1.5313
5.5857	7.3331	0.5418	7.4764	6.5812	5.9964	6.5825	7.8107	9.1260	6.6335	9.5119	7.0815
3.0777	6.1113	5.9991	6.2011	6.0736	4.8974	5.6281	6.3953	7.9263	5.5633	8.6006	4.3911
0.6312	2.2026	2.3060	3.4434	2.4685	1.5300	1.8968	2.0016	2.8347	0.3104	3.4364	1.9911
1.0784	-1.1117	0.7739	-0.9716	0.6832	-0.6804	0.0990	0.6507	0.1619	-0.0143	-0.0221	0.0549
-0.6036	-2.0018	0.8160	-1.5403	0.1326	-1.2091	-1.8252	-0.0970	-1.5215	-0.5775	0.1511	0.1288
1.3892	0.5080	2.5398	2.5123	2.2976	1.2432	1.7098	1.6344	2.3464	1.3799	2.3895	3.0288
-8.0591	-4.2522	-1.3369	-5.3384	-5.3806	-1.8894	-2.4166	-4.4657	-1.2992	-2.0147	-5.7892	-3.5111
0.9857	1.8395	1.7954	3.0848	-0.1326	2.4747	1.3683	0.4125	0.4865	-0.2977	-0.4577	-0.5469
-4.9939	-5.3574	-7.0186	-7.4286	-9.0964	-5.9468	-7.2501	-5.6079	-3.4316	-7.4726	-4.5519	-5.3552
3.0755	1.1916	3.9854	3.5924	2.3514	3.1367	2.2636	5.0777	2.2968	3.2752	1.7402	3.4739
6.0123	5.2444	6.6249	5.8085	6.9456	6.6084	4.8472	6.2141	6.5981	4.2128	5.9673	6.0093
-3.2868	-1.7591	-1.1041	-1.4306	-0.6030	-0.1995	-1.5070	0.0970	-1.3742	-1.8539	-2.8687	-0.0549
0.3009	0.1473	0.9478	0.4292	0.6157	1.7270	-0.2203	0.2813	-0.5270	0.2640	-1.1106	-0.1897
1.7706	-4.2933	-4.8320	-3.9759	-6.5575	-3.4544	-5.3163	-4.1135	-6.7825	-2.7239	-4.6589	-5.1951
-0.5416	-1.4295	-2.2151	-0.5006	-1.9353	-0.1844	-1.5867	-1.2877	-2.6813	-3.4058	-1.0241	-1.6204
6.3740	8.7588	9.1471	10.4637	9.7532	7.8192	8.0930	7.8755	10.1957	7.4793	9.7066	9.1671
7.5993	8.6875	9.0037	10.0409	9.5741	8.0524	7.0767	8.2442	8.9595	6.8776	9.5197	9.6904
-8.0591	-4.3268	-1.0162	-7.4286	-8.1934	-1.9064	-7.2501	-2.5242	-4.7862	-0.8576	-1.7296	-4.6790
-1.8054	-0.5799	0.6287	0.7712	-0.5264	1.8348	-0.7537	1.5763	0.6802	0.8902	1.1209	1.0735
-8.0591	-2.3679	-7.0186	-7.4286	-5.6015	-5.9468	-3.6745	-7.8454	-3.5957	-1.2464	-4.8777	-6.8622
4.2973	3.8197	4.9667	6.0614	4.6928	3.8358	5.7208	5.1703	4.8949	4.5798	4.3603	5.9711
-8.0591	-8.2140	-7.0186	-7.4286	-9.0964	-5.9468	-5.5000	-7.9155	-6.7825	-7.4726	-5.7892	-6.3482
-8.0591	-8.1267	-7.0186	-6.8160	-9.0964	-5.9468	-7.2501	-8.4976	-6.7825	-7.4726	-5.7892	-6.8622
7.3263	9.8930	9.0351	9.4408	9.3517	9.3134	9.2698	8.9650	9.4560	8.6746	10.5399	9.5057
-8.0591	-8.6268	-7.0186	-7.4286	-6.3956	-4.7678	-3.8884	-8.4976	-5.0730	-2.3686	-5.7892	-6.8622
4.6000	5.2595	4.7745	5.5923	5.1453	4.6185	5.7500	4.9225	5.1549	4.5559	5.2656	6.3062
-6.1692	-8.6268	-5.0862	-6.9718	-9.0964	-4.2314	-7.2501	-7.2130	-6.6868	-7.4726	-5.7892	-5.7337
-2.2722	0.9824	1.0686	2.2374	0.8440	1.3536	2.8472	1.1083	2.3330	1.2050	1.7788	2.3266
2.2602	4.8317	4.9071	5.1509	4.6849	4.5703	4.2644	4.2166	4.9592	3.3353	5.4993	5.1406
-1.3178	-0.2896	-0.7363	-0.5574	-0.6037	-0.9763	-0.7082	-0.6117	-1.4466	-1.8184	-0.7738	-0.4655
5.7360	5.2350	5.5608	4.9379	5.5389	5.0374	5.3782	5.4881	6.1941	4.5384	6.0773	5.9205
2.5898	5.6726	6.9416	5.4095	8.3246	4.3504	3.7275	5.3767	6.4377	3.1699	6.6765	4.9421
-2.1717	-0.1473	-0.9571	-0.4822	-1.2346	-2.8013	1.2220	-0.5714	1.2200	1.7991	0.9999	2.7531
3.9588	4.5184	6.7348	6.0621	4.9298	5.8167	6.3514	6.6216	6.1220	5.5973	5.7249	7.1309
2.2717	3.2901	4.4790	3.0337	4.4937	3.5194	3.7649	3.9621	4.0379	2.6417	3.8425	4.8385
6.5983	7.6594	7.3125	7.2163	8.2924	7.5168	7.6669	6.5949	7.3444	8.1774	8.0939	8.8767
-8.0591	-8.6268	-7.0186	-7.4286	-9.0964	-5.9468	-7.2501	-8.4976	-6.7825	-7.4726	-5.7892	-6.8622
-8.0591	-1.7525	-7.0186	-7.4286	-6.0192	-5.9468	-4.6887	-6.5068	-5.0209	-2.3339	-3.9871	-6.8622
-7.1827	-1.7208	-5.3965	-4.5479	-5.5746	-4.1000	-3.6014	-4.9585	-5.4651	-0.4532	-5.4638	-4.2647
-1.1370	-0.8218	-7.0186	-0.3570	-9.0964	-5.9468	-7.2501	-8.0476	-6.7825	-7.4726	-5.7892	-6.8622
-8.0591	-8.6268	-7.0186	-7.4286	-9.0964	-5.0979	-7.2501	-8.4976	-6.7825	-7.4726	-5.7892	-6.8622
-0.3009	0.7551	-0.9363	0.4438	-5.5516	-0.1089	-3.0448	-1.5728	-0.1619	-4.0387	0.0221	-1.5274
3.6102	4.2643	6.1352	5.7915	5.5848	5.9635	4.2431	6.0294	5.4779	4.0026	5.3905	5.7854
3.8689	4.3184	5.2387	4.7607	5.7853	4.9891	2.7580	5.3853	4.2911	3.6174	4.0522	4.1418
3.6452	7.8712	10.5629	10.8275	8.9299	11.3627	7.8895	11.2961	9.8518	8.6859	7.9432	9.8199
6.3934	6.0577	9.7489	8.0443	9.9413	7.0276	8.2490	10.0769	9.7648	8.3805	8.6661	9.5434
0.7390	0.4219	1.1723	0.1127	1.4044	2.5022	0.9102	2.9484	1.9969	2.2039	1.1144	2.1385
6.1981	5.6842	6.7747	7.3446	7.1943	6.6008	5.6830	6.7549	6.8061	5.8621	5.3842	6.3898

Table S4. Normalised Ct values for plates A and B.

78	79	80	81	82	83	84	85	86	87	88	89
-2.9568	-3.6506	-6.9314	-5.9151	-5.0355	2.1116	-6.6214	-6.9865	-5.4302	3.9585	-2.9056	-2.8325
-4.9204	-6.0096	-8.8485	-10.5114	-7.2890	-2.7249	-8.7693	-10.0447	-9.3152	-0.1378	-7.5646	-6.3452
-2.8639	4.7118	-2.7989	-2.3348	-6.4397	1.4414	-8.6401	-3.0877	-3.7238	-6.3388	-6.7428	3.4730
4.7036	2.2046	3.3137	1.4344	3.6351	2.3296	4.4871	3.2570	4.0819	4.6620	3.5316	3.0878
7.2803	4.1896	4.1952	3.6018	4.1779	4.7180	4.5096	3.5439	4.5974	6.2706	5.6445	4.2353
0.7659	0.2690	-0.3561	0.8774	1.3182	0.5556	0.7768	0.1879	-0.1589	3.2753	1.7912	0.2328
4.9491	4.3544	5.2338	6.3667	5.9298	5.1196	4.8229	4.8736	5.0120	7.7190	6.8207	5.5618
4.9079	5.9942	4.7689	6.6737	6.2525	6.1464	5.7832	1.5391	5.6735	8.9726	7.2364	5.5681
6.7545	5.3672	1.1300	4.3335	5.5158	4.5910	5.9215	3.9066	3.3772	5.9254	4.8914	3.3794
8.6973	5.3489	3.9851	3.3560	6.9856	6.0892	7.2232	7.3062	7.1970	6.6568	6.2401	5.2337
4.2286	4.7630	4.2269	2.9528	5.0098	2.4711	4.9618	4.4117	5.2265	5.0205	6.1874	5.0134
2.7532	-0.0766	-0.4437	0.3333	-0.7132	-0.2323	0.1735	0.4737	-0.4094	1.0863	1.1716	0.4222
7.5465	4.1536	3.1276	3.6441	5.3513	4.1839	4.4822	3.0994	4.0011	5.4578	4.9983	3.6478
7.2758	4.8906	6.3701	6.3798	6.3194	5.7137	6.3391	5.6164	4.8990	4.9352	5.1725	5.3255
5.8342	3.8478	4.3471	4.0566	5.3031	3.6359	4.7393	3.6782	2.5801	5.1177	2.8667	3.7384
-1.1261	3.9501	4.9736	3.9897	2.9742	1.3053	3.6684	4.0319	4.2693	6.1683	4.0309	4.6426
-0.7685	0.8067	0.6742	1.4242	2.0205	0.6476	2.1299	0.8556	0.8801	2.5396	1.1782	0.0769
2.9385	0.0766	-3.5097	-1.9982	-0.0242	-0.9308	0.4596	-1.6380	-0.0265	1.1898	1.0325	-1.0463
5.3676	5.0240	3.7924	2.5342	6.1277	4.0968	4.5017	5.4927	3.6797	4.0625	4.0835	3.3657
4.6764	4.0377	2.5101	0.6407	4.5562	2.3267	3.4575	3.6273	2.3505	1.7027	2.1871	2.0003
-1.9518	-0.1049	-1.0684	-1.9337	0.0242	-0.3799	-0.5969	-0.3488	0.2820	0.1378	0.6993	-0.0769
8.0017	5.7254	4.9476	4.9185	6.3768	6.1387	5.8129	5.4381	5.1841	5.7384	4.8957	5.3552
7.8252	4.8565	4.6284	4.1159	4.6238	4.5262	5.0474	4.2932	4.2266	5.3542	4.5677	4.0031
4.6338	3.4378	2.5634	3.2756	4.0560	4.1278	3.8683	2.5493	2.8848	3.8050	2.8331	3.1191
0.6580	2.8294	2.9141	2.8072	3.9463	2.6469	2.9006	2.6659	2.0707	2.9855	0.8309	1.8627
-1.3711	-2.1308	0.9368	0.0526	-0.4205	2.2533	2.0698	-2.3819	0.8595	3.7330	3.4519	-1.8051
-7.9733	-10.7663	-11.3739	-11.9492	-7.2890	-10.6505	-9.3528	-9.8571	-10.1898	-10.3515	-5.6395	-12.0852
7.8670	6.6056	5.5991	8.0896	6.5402	7.4333	7.3361	6.7927	6.2581	7.2359	8.3490	6.3774
-7.9733	-11.6535	-7.7228	-11.9492	-7.2890	-10.0390	-11.0301	-8.9209	-12.2515	-10.3515	-11.8708	-11.7559
1.7315	-0.3960	0.7384	0.3116	0.2104	-0.1431	1.8618	0.1490	0.6780	0.7054	-0.0433	1.6743
3.6384	1.2608	6.7739	2.0958	0.8854	1.5053	-2.0264	5.5721	1.3377	-5.9658	-2.8872	0.9445
1.0767	0.5122	6.8605	3.1027	1.6750	0.7507	-0.7430	7.0442	1.1561	-5.0238	-2.9428	1.1448
6.3898	3.3877	5.1872	4.8684	4.6347	2.9322	4.7003	4.1707	4.1495	5.8661	1.2926	4.5631
2.5217	3.6562	3.8262	5.9863	4.4137	4.0348	4.6790	3.9733	4.3218	7.8494	6.1922	4.4992
-1.9641	-2.9860	-1.6446	-1.1558	-2.5855	-3.2462	-1.8540	-2.9730	-1.5116	0.2011	-1.9061	-0.7332
-7.9733	-3.2223	0.8779	-1.8673	-1.0719	-1.5781	-0.3136	-3.1112	-0.1378	0.5416	-1.4132	-0.1261
1.2545	0.8226	2.4697	4.8358	3.3543	-1.7944	4.3144	2.3457	1.5272	-0.6547	3.4705	-1.6001
-7.9733	0.8118	6.6582	2.8573	-7.2890	1.0223	-1.1489	5.7414	0.7103	-10.3515	-2.6274	0.6230
8.0229	1.2470	0.8173	6.2388	6.4100	2.8356	6.6932	-0.9523	4.6224	-3.5941	-1.1036	2.1445
-7.9733	-3.3711	-0.2948	-11.9492	-7.2890	-10.0502	-6.3023	0.5931	-9.2053	-10.3515	-10.3662	-11.9826
-4.2165	-4.5701	-3.4666	-4.0316	0.3637	-3.7341	0.5260	-2.2707	-0.7721	-3.9594	-1.6801	-3.0759
-2.0559	-5.5279	-3.9127	-5.6752	-1.2646	-4.8255	-0.3519	-2.2108	-1.3033	-4.5586	-2.9576	-5.2172
-7.9733	-8.0054	-8.4305	-8.4136	-7.2890	-8.4665	-8.4788	-6.3763	-6.9478	-6.2356	-7.8260	-8.0887
6.1996	3.9362	-0.5815	4.4502	5.7575	2.4333	5.4909	2.7533	3.9673	-2.2810	1.5832	1.2224
4.0531	1.4039	1.7240	1.5958	3.6850	1.2163	2.8050	2.4298	1.6853	3.3008	3.3512	1.2016
0.4749	-0.3126	-2.6128	-1.8973	0.6102	-3.0778	-1.4308	-2.5673	-2.3916	1.6702	-1.3533	-1.1987
-2.4278	-4.1349	-8.1413	-5.2270	-7.2890	-0.1705	-7.1700	-5.8276	-2.7399	-6.7509	-5.3539	-3.7568
-2.3978	-4.6140	-6.6810	-4.9440	-7.2890	-0.2211	-5.2332	-5.5987	-2.4475	-3.7182	-5.5133	-3.4056
-4.0856	-4.6193	-8.5572	-2.5501	-3.2005	-5.4177	-3.6239	-9.3473	-2.8747	-8.4673	-8.1773	-9.3909
5.0687	2.6779	2.4583	2.2380	3.7170	2.2164	2.5181	2.2129	2.0026	3.9253	2.9763	2.3396
2.2030	3.8372	4.6368	2.5544	2.8546	3.4885	4.2831	4.0870	3.8451	5.1921	3.1256	3.8256
-0.0324	-1.6845	2.1591	-1.6682	-3.7908	-2.4360	-0.1735	-0.1807	-2.1226	0.5987	3.3204	-2.2846
8.4948	4.2317	4.5008	5.1686	8.2426	4.4998	7.2569	5.7611	5.1301	-0.3703	2.0592	3.9725
-7.9703	-7.4462	-10.8963	-11.5551	-7.2890	-11.4263	-9.6461	-9.5249	-9.9282	-8.7536	-10.5664	-10.7434
0.8522	-0.8503	-0.0342	-4.8877	-2.4685	-1.4610	-0.7299	1.1231	-2.2397	-0.1988	1.8685	-4.0467
7.4496	3.2713	4.0349	4.9261	6.6434	1.6434	5.0152	2.2010	3.9067	2.2635	4.2449	2.9316
-0.5090	-2.7336	-4.2529	-0.0526	-3.0149	-2.1763	-4.4989	-7.6356	-4.2126	-4.9626	-4.6029	-3.6615
0.0324	-3.0405	-0.8466	-3.8068	-2.6863	-3.6564	-2.8480	-3.8044	-5.2767	-1.1596	-0.7078	-4.4842
-4.3418	0.2526	2.9653	-6.6835	-5.4577	-7.1561	-4.7929	3.0191	-5.6086	-6.9243	-8.1715	-4.9491
-1.7098	-0.1093	0.6304	-6.6835	-5.4577	-6.2896	-3.5554	2.4970	-5.4216	-6.9243	-6.1722	-6.4502
8.9007	7.0102	2.3903	5.5344	6.2749	8.2394	7.9481	1.5865	5.5875	5.7761	5.4318	8.0683
9.6282	10.6853	10.7762	11.0955	12.1191	11.5507	12.8371	10.5954	11.6253	7.6322	8.5981	11.5753
11.9584	9.6998	8.7698	9.9871	11.1357	10.4605	11.8188	9.6321	10.3750	7.3867	7.9142	10.6691
4.0787	5.5596	5.3533	6.5413	6.3370	6.4769	7.6547	4.5639	6.9863	2.0770	3.0011	6.9540
0.8962	-2.2455	-2.7618	-5.1123	-5.4577	-1.3623	-2.3601	-3.8838	-3.5091	-0.5561	-4.6368	-2.7893
-4.3418	-6.8176	-8.1355	-6.6835	-5.4577	-7.8752	-7.9253	-9.0335	-7.7801	-6.9243	-8.1715	-6.4502
-4.3418	-3.6542	-7.2638	-5.7784	-5.4577	-9.0980	-3.3819	-4.3540	-3.1977	-6.7136	-6.3087	-5.7815

Table S4. Normalised Ct values for plates A and B.

2.0872	1.6720	-0.2545	2.3387	2.2764	1.9746	2.5376	-0.0970	3.1832	2.6110	1.7781	2.6316
-4.3418	-6.3314	-8.7488	-6.6835	-5.4577	-3.8288	-4.2078	-9.0335	-3.1615	-2.5895	-3.5994	-5.3752
-4.3418	-0.3961	-6.3838	-5.4098	-4.6929	0.4917	-1.5421	-4.7754	-3.2232	-1.9290	-2.6499	-3.8718
1.8206	-1.2930	2.1745	0.6536	0.7033	0.3981	2.3581	0.9455	1.9927	2.2658	2.0954	1.3770
3.2080	2.9728	2.1642	4.1302	5.1941	3.7963	4.4601	0.1518	3.1217	5.3077	-2.8339	3.3406
6.4401	5.7326	7.0256	5.6750	4.7387	6.1896	5.8714	2.8368	5.7725	6.0719	4.3331	5.1935
9.3240	5.2404	5.0202	6.7087	6.3161	6.2634	7.0007	4.2072	6.1607	7.9492	6.5157	6.5415
0.1032	3.0076	-1.5920	0.4571	-2.8700	0.8669	-1.4337	-4.5234	-0.5446	-3.4431	-2.1865	-1.3809
10.1189	-6.8176	5.3389	4.5836	7.2532	-9.0980	8.3143	6.2310	8.1442	5.9381	7.1392	5.9900
7.4951	6.3995	4.0329	3.5763	4.7712	6.5143	7.6322	5.3598	6.7427	5.0797	6.3059	6.7866
-1.2757	3.6396	2.8697	1.5106	2.0812	2.6616	3.5615	0.0970	2.9282	-0.0074	0.6088	0.9527
0.9398	-0.3745	-2.6456	-0.2544	0.1843	-0.5035	-0.1326	-2.5339	-0.8084	-0.1297	1.0021	-0.3457
1.4973	-0.6146	-3.1158	-0.1148	-0.1560	-0.1911	-0.9147	-3.6490	-1.9010	0.0634	0.0366	-0.6474
3.9590	1.7424	0.2545	1.4499	1.2907	1.7412	2.1154	0.1372	1.6801	3.4979	2.9473	2.9126
-4.3418	-2.3292	-3.0040	-4.6915	1.9554	-3.2506	-2.8575	-2.1467	3.1419	-3.6083	-6.6018	-6.4502
4.4787	3.5172	-5.0894	-0.4341	4.6804	-7.4367	-1.3560	-3.3739	0.1191	1.5856	-4.9008	0.1509
-4.3418	-6.8176	-8.7488	-4.8135	-5.4577	-6.8062	-7.9253	-4.9465	-5.0867	-6.9132	-8.1715	-5.6036
4.6358	0.6930	-0.8649	0.9898	4.7966	3.2506	3.1024	1.8587	2.4007	2.6958	2.8667	2.0584
6.8265	5.8152	4.2032	6.5307	3.3851	6.6448	7.0889	5.3452	6.6028	1.4273	4.1278	6.9501
-0.1032	0.0040	0.2842	-1.9579	-0.6202	0.9572	-0.5277	-5.1175	0.5839	-2.5939	-0.0366	-0.4918
-1.8280	-0.9501	-1.9298	0.1148	0.1560	0.1911	1.5813	-4.0845	-0.1720	-0.3920	0.8039	-0.0509
-4.3418	-3.0118	-0.8877	-4.8002	-3.1893	-0.5754	-4.3192	-4.9281	-3.7686	-2.9325	-7.7713	-5.2190
-1.7273	-1.6696	-2.6983	-0.6057	-0.8444	-0.5126	-0.8900	-3.5228	-1.1535	-2.0029	-4.8826	0.0509
9.7142	9.5642	8.5128	10.0652	7.2921	8.6019	9.5708	9.0577	8.9576	11.8306	9.6824	11.3529
10.5044	9.2155	8.1690	10.7145	8.2777	9.0957	10.1158	8.6225	9.3786	11.7453	9.1566	10.9304
-4.3418	-1.2316	6.2509	2.6884	-5.4577	-4.6259	-7.9253	5.1958	-0.1766	-3.3959	-8.1715	-3.5542
2.2852	0.9607	-1.5366	-0.2621	-0.2978	1.7019	2.5453	-2.8834	-0.1191	5.6529	0.1389	0.4973
-1.6788	-1.2000	0.5240	-6.6835	-5.4577	-8.4358	-4.9800	1.4315	-4.9073	-6.9243	-8.1531	-6.4502
3.5656	4.1172	3.1441	5.0920	2.3017	3.7579	5.4147	3.4239	4.0164	5.5539	3.9162	6.0307
-4.3418	-6.8176	-8.5061	-6.6835	-5.4577	-9.0980	-7.9253	-9.0335	-5.5506	-6.6281	-7.3421	-6.4502
-4.3418	-4.4217	-7.7849	-5.9719	-5.4577	-9.0980	-7.9253	-9.0335	-7.5556	-6.9243	-7.6250	-6.4502
8.2608	8.6479	7.4448	10.5167	-5.4577	9.9587	10.7669	7.0766	9.7534	9.2656	9.2459	9.9988
-4.3418	-1.2733	-1.1462	-6.6835	-5.4577	-5.9636	-4.7084	0.8232	-6.4151	-6.9243	-8.1715	-6.4502
6.7849	4.5741	3.4104	4.8752	5.4677	4.6801	5.6925	3.6565	4.3759	4.4980	5.2221	5.7845
-4.3418	-4.0925	-6.6091	-5.9709	-5.4577	-4.2925	-7.0707	-8.4683	-5.2126	-6.2217	-6.2349	-6.4502
-0.1874	1.0272	-1.1270	1.5845	0.6671	1.1425	1.1213	-1.3440	1.7381	1.3721	0.8724	2.1873
5.9130	5.3556	3.2348	4.7100	5.3123	5.0717	5.5490	2.6281	3.7031	6.1915	4.8543	5.9332
0.2595	-0.6960	-2.4251	-0.3733	0.2031	-0.9114	0.1009	-3.5566	-2.0418	0.0074	-1.9175	0.1331
5.6267	4.9755	2.9478	5.6380	4.8023	4.5309	6.1244	2.7142	4.8522	5.3325	4.7144	6.5230
7.1272	7.7769	4.3787	8.0086	5.9528	6.3100	7.0471	3.1604	7.4572	5.8336	2.9042	8.5383
-1.4532	1.5999	1.7443	3.0756	-1.3842	-0.6159	1.1498	-4.4158	1.0823	2.5267	1.1943	0.9745
6.2677	4.0769	2.1665	5.2717	4.1117	5.9322	4.2433	2.3964	3.8538	7.2191	6.3646	6.8263
5.8585	3.2023	1.8359	2.8411	3.6067	3.0077	4.8781	1.0696	3.9726	2.8393	2.2757	4.6911
9.3655	7.2527	5.2629	8.3859	-5.4577	7.5626	8.4200	5.2887	7.0681	7.9658	7.0727	9.0604
-4.3418	-6.8176	-8.7488	-6.6835	-5.4577	-9.0980	-7.9253	-9.0335	-7.7801	-6.7923	-8.1715	-5.1036
-4.3418	-0.0040	3.1599	-6.6835	-5.4577	-9.0980	-6.1571	2.0765	-4.2045	-6.9243	-8.1715	-6.4502
-2.5829	-1.4433	-0.4827	-6.6835	-5.4577	-3.4383	-3.4230	1.1802	-3.2881	-3.1550	-5.5990	-4.6150
-4.3418	-5.0928	-8.7488	-6.6835	3.3031	-7.7412	-7.9253	-9.0335	-6.7516	0.4360	-8.1715	-6.3161
-4.3418	-6.8176	-8.7488	-6.6835	-5.4577	-9.0980	-7.9253	-9.0335	-6.8683	-6.6009	-8.1715	-6.4502
-2.5516	-0.5424	-1.6884	-0.2008	-0.3499	-1.2879	-4.1170	-3.7283	-0.2100	-0.3593	-1.1429	-0.8720
6.7790	5.3314	2.7982	5.8693	4.1592	5.4653	6.9700	3.0683	5.7210	4.0320	4.3934	6.3556
2.6917	4.6667	6.4802	5.8484	4.4201	5.5394	6.0955	5.2269	4.9125	6.9583	5.7126	6.0228
6.8038	10.3902	7.7777	8.6411	7.8092	11.0838	10.4984	6.0576	8.6026	8.8260	10.2110	10.4324
8.5489	9.9004	5.9501	6.5067	6.7786	10.9921	11.1966	7.0786	7.3736	6.9905	6.8428	7.4617
-1.4790	1.2681	0.4039	2.8634	1.3494	1.0412	4.4822	-1.0487	2.3134	-3.0574	2.5066	-0.3386
7.3313	6.1373	5.3635	6.3958	6.0794	7.1053	8.2279	4.4293	6.6265	6.2860	5.6968	7.0948

Table S4. Normalised Ct values for plates A and B.

90	91	92	93	94	95	96	97	98	99	100
-2.4016	-4.6210	-5.8554	-4.5489	-3.3605	-4.2530	-5.8005	0.3544	-2.8633	-0.2602	2.9674
-7.6786	-7.0915	-9.0095	-7.7997	-5.8918	-7.8671	-9.1379	-4.0821	-5.8259	-4.0554	-1.2956
5.8841	0.9408	-3.4059	-3.0663	-3.1395	-4.8273	-3.7110	4.6316	-8.7260	4.1386	5.7527
4.1865	3.6340	4.2093	3.2642	2.7879	3.3173	2.5781	3.6086	2.7033	3.9936	4.4370
5.9820	5.1576	5.0628	4.1604	5.1133	4.9198	3.8218	4.5371	3.9403	5.1523	5.9325
0.5613	-0.1541	0.4767	-0.4360	-0.7520	-0.3630	-0.1211	-0.2020	1.8093	0.2602	0.9610
4.8029	4.4468	6.1576	3.8175	4.6045	4.7607	4.1396	4.4523	5.7637	5.2105	6.8991
3.7101	4.3205	3.7894	6.0404	6.4007	6.3415	6.1068	5.4948	7.7761	6.1750	5.8000
2.8662	4.5011	3.1412	3.9536	4.1678	4.9443	3.5308	4.3129	6.3920	4.6384	4.8672
7.4380	4.7725	7.8699	8.4429	5.0576	5.2640	6.3753	8.4540	6.6309	8.2790	5.8679
5.4900	4.0597	5.7031	4.4023	5.4820	5.3753	2.9936	5.4621	5.7121	5.8374	2.3280
0.8821	-0.2361	1.2531	-0.0954	0.8086	0.5279	0.1588	0.4919	-0.1025	0.7525	-0.1776
3.7321	3.7158	5.7273	4.2089	5.3999	4.4260	4.3761	2.9903	4.2853	4.1377	4.0188
5.9237	5.7243	5.5586	5.8400	3.8380	5.6726	5.6827	4.6933	5.8799	5.8629	4.7335
4.0398	4.1887	3.7329	4.4426	3.5925	4.2058	3.5578	3.6801	4.2902	4.4942	4.1578
4.5443	2.1111	5.7854	3.6638	3.6612	4.9033	2.6479	4.0885	5.0197	4.2748	4.6903
1.0262	0.3078	-0.4310	0.7713	1.5851	0.8729	0.3654	0.7327	1.4335	0.9617	1.9678
1.9329	-0.0512	-0.0840	-0.4561	0.5099	0.2317	-0.6332	0.8446	-0.2426	1.2554	0.8400
4.8171	3.6090	4.2895	5.7921	3.6666	3.2919	5.2785	4.5570	4.5950	3.8642	5.9630
2.9290	3.4974	-0.1160	4.3807	3.2531	1.7643	3.5930	3.2958	3.4730	3.0195	3.0320
0.8306	-0.5125	0.8246	0.1597	0.4183	0.6751	-0.3308	0.2113	0.1006	0.7039	-1.2218
6.3638	4.5941	6.3956	5.6791	5.0059	6.3052	5.7610	4.9542	6.2414	5.5935	5.6581
5.5810	4.7422	3.8883	4.3958	4.4738	5.0582	4.9536	4.4740	5.0493	4.8376	3.8268
4.3216	2.8132	3.5938	2.5780	3.0875	3.8356	2.6656	2.7502	3.5916	3.3344	4.3040
1.5376	0.9689	3.2073	3.1255	1.6459	2.2471	2.5964	0.5342	4.3041	2.2307	0.7173
-9.8511	-0.0236	1.7167	-0.4715	-2.8673	1.1252	1.9863	3.8246	-0.6129	2.3620	3.8453
-9.8511	-11.8712	-10.1363	-11.7585	-5.8949	-11.7791	-6.4983	-10.7697	-5.9267	-6.2423	-10.2431
7.1991	6.5502	4.0234	5.8727	5.6447	6.3584	6.4362	5.7118	7.8235	6.2321	7.0161
-8.8631	-9.4134	-9.3311	-8.3176	-7.1635	-11.6866	-8.1719	-9.8862	-10.0161	-11.8827	-10.2431
0.1426	0.0796	0.4455	0.7419	0.5927	0.5841	0.0412	0.6730	0.0813	0.7972	-1.2166
-2.6513	0.4678	1.0153	6.2650	-0.6509	4.2628	5.3129	-2.8434	-0.5509	-1.2395	0.7324
-3.1663	0.0236	0.8397	6.8827	-0.4183	5.1447	5.9075	-1.2802	-0.4271	-0.5207	1.6718
5.1733	4.4973	4.9007	4.2754	3.9790	4.2068	3.9044	5.3324	3.5469	4.9349	5.1479
3.7342	3.8231	3.8289	3.4147	4.0581	4.1529	3.2908	4.1320	5.2236	4.2061	3.6623
-1.1035	-1.9307	-1.3780	-2.9133	-1.4836	-1.2623	-3.1759	-1.2230	-2.0201	-1.6524	-3.7025
-0.4518	-0.2398	-1.1744	-0.6734	0.7402	-3.9364	-4.1305	-1.8885	-0.9821	-1.2825	0.1776
0.1258	3.3406	0.5029	2.5114	-0.9323	2.6531	2.8254	-0.0875	3.8844	-1.1462	-0.7341
-3.9481	1.1464	-6.2326	-5.1697	-1.4417	3.9290	-4.2591	-2.5189	-0.0162	-0.8452	-10.2431
0.7894	3.0115	3.7651	0.3786	1.7237	-6.0467	1.5711	-0.8890	3.0325	0.7576	2.2366
-7.8809	-2.9648	-10.1363	-4.8035	-2.2225	-11.0304	-5.4018	-10.6053	-10.5720	-9.7217	-10.2431
-2.7089	-3.0547	-3.1942	0.0502	-2.4268	-2.8956	-1.1746	-3.0425	-3.0177	-2.4813	-3.0956
-4.1541	-5.3525	-5.3251	-0.0502	-3.0614	-3.7782	-1.4232	-4.2243	-4.5150	-4.1356	-4.4592
-8.0828	-9.1982	-7.6533	-6.1522	-5.7211	-8.1132	-5.2121	-8.4296	-7.0324	-8.0128	-7.6475
-0.3391	3.0066	4.4042	3.2376	3.4910	2.0813	4.1933	-1.0363	5.0924	1.2844	1.0477
2.2714	2.2175	3.0011	2.1560	1.6775	0.9548	2.3608	2.0564	2.4370	2.4210	1.9523
0.0740	-3.9580	-0.6095	-2.0848	-1.8543	-3.2171	-1.6327	-0.7283	-2.7138	-0.9789	-2.0335
-6.0954	-4.5356	-1.5483	-6.5563	-5.0734	-9.1303	-5.2356	-4.0245	-0.0130	-5.3172	-3.6689
-5.7428	-4.5783	-2.1816	-5.0282	-5.6271	-7.7962	-5.7000	-4.4530	0.4883	-5.4413	-3.9548
-8.7731	-4.6542	-6.5485	-7.0650	-5.4961	-7.6952	-6.0690	-7.9178	-5.5724	-5.3385	-6.4727
3.7502	2.2554	3.7826	2.7951	3.6907	2.5897	2.7262	3.2472	2.9534	3.4559	2.9903
3.0836	3.4231	4.7564	4.4104	4.3585	3.9625	4.0978	2.9706	4.3458	4.3139	2.5411
-9.8511	-2.5388	0.0840	0.5587	3.5886	-1.2524	0.9190	-1.4437	0.2059	-1.9774	-0.9126
-9.8511	3.4572	5.8907	5.3832	2.5962	4.5145	5.6270	4.0087	3.5195	2.8932	3.8920
-9.8511	-10.9897	-7.6244	-9.8068	-7.2558	-10.3827	-11.3514	-10.7813	-8.6678	-10.8881	-10.2431
-0.0740	1.0531	-1.1677	4.3667	4.1743	-0.2317	2.2092	2.0116	0.0130	-0.4634	-5.2336
3.6933	4.9191	3.9921	5.9767	4.3290	0.6434	3.0221	2.8064	2.6664	3.1567	3.7570
-2.3777	-0.7130	0.8432	-3.1140	-1.9368	-8.4797	-4.7409	-0.8244	-4.4175	-1.2098	-4.3517
1.5255	-1.4837	-2.3555	-0.6967	-2.5807	-4.1790	-0.9890	0.6974	-2.7388	-1.5396	-2.4297
-5.3968	-1.3464	-6.1465	2.5914	-0.5862	-7.0267	3.6395	-5.8848	-4.5571	-7.7675	-7.1222
-5.3968	-0.7912	-4.6929	2.3439	-0.2313	-7.0267	3.5684	-4.8497	-5.0461	-6.4106	-7.1222
7.5344	6.0395	5.9923	1.7342	3.1064	6.5530	3.6218	6.9383	4.5358	7.0211	10.0897
9.8652	6.8201	11.2773	10.2554	6.7517	13.8784	11.4874	9.8049	10.3100	8.9015	11.2553
9.3379	6.2406	9.9743	8.3672	6.6504	12.5270	10.3918	8.8905	9.0839	7.5425	11.2264
3.7706	2.7136	5.8732	4.7939	1.1639	8.1583	5.7236	4.2822	5.5876	3.6375	5.6349
0.2681	-0.7051	-1.5031	-2.1137	-0.4214	-1.9376	-3.2742	-1.6623	-4.4472	-3.9886	-2.3876
-5.3968	-7.7511	-6.1465	-8.2790	-5.8804	-7.0267	-8.5448	-7.1846	-7.0756	-7.7675	-7.0977
-5.3968	-6.1970	-2.0598	-6.7717	-2.4284	-6.1019	-8.5448	-7.1846	-6.1848	-6.0649	-1.3673

Table S4. Normalised Ct values for plates A and B.

2.7202	3.3484	2.8792	0.7709	1.8552	3.4389	-0.1304	3.6709	2.0881	2.9576	3.0800
-0.9909	-3.1347	-2.8534	-7.3847	-5.8804	-5.9696	-8.5448	2.6923	-3.5396	-0.1039	-4.7091
-5.3968	-0.0391	-3.3439	-4.0951	-5.1608	-1.5056	-3.7124	-4.4864	-0.1994	-1.8994	-7.1222
1.4363	1.3637	3.8152	1.2099	0.3174	1.0880	1.7203	3.1423	1.7464	2.7790	-0.1526
3.0584	4.0405	3.5205	1.7316	1.4521	4.5532	1.7589	3.8458	4.2065	1.8430	1.7299
5.1021	5.8179	5.9252	2.9697	3.0356	5.6464	4.2798	5.9265	4.3976	5.5614	5.6004
7.7637	5.3127	7.6889	3.8303	6.5188	7.7254	4.6744	8.0028	5.8359	6.3775	7.4977
2.6155	-2.5542	-0.8212	-2.1915	-0.0006	-0.8125	0.1304	-0.2874	-2.5676	-1.8958	0.6883
-5.3968	4.8502	8.5008	6.4214	7.3158	7.1557	7.2076	5.9177	6.8150	6.3193	-7.1222
5.3366	3.9355	7.2169	5.3127	6.1538	6.3788	5.6429	5.0604	4.9834	5.4834	6.3152
2.5041	0.2657	2.8029	0.7223	2.5114	1.4050	2.7979	0.9838	1.4640	0.4526	0.0280
0.7093	-0.6440	-0.1510	-2.6396	-1.5431	-0.5832	-1.5530	-0.5563	0.6601	0.3268	-2.6164
0.8538	-0.6913	-0.5791	-3.0542	-2.0477	-1.0568	-2.0551	-0.8859	0.6160	-0.3989	-0.9031
3.2103	1.4480	3.1694	-0.7223	0.9260	3.5945	-0.9912	2.9123	2.0540	1.9957	1.0824
-3.3576	-7.7626	-6.1465	-1.9560	-5.8804	-7.0267	-0.3947	-3.5522	-0.7952	-6.5772	-6.4967
-2.6364	0.7798	-0.2314	-2.4435	-0.1833	0.0123	-4.0615	-1.5338	1.4450	0.2990	1.8458
-5.3968	-9.7143	-6.1465	-9.6251	-5.8804	-6.6237	-8.5448	-6.3281	-4.5205	-6.6048	-7.1222
-5.3968	2.7729	4.0191	1.8996	2.8552	1.4449	2.1005	7.6898	1.0372	1.5984	2.8964
4.2307	0.4848	7.0772	5.1519	2.1963	8.4381	5.9487	4.8948	4.6125	3.0077	6.2922
-0.2681	-2.0745	-0.4501	-4.0271	0.1504	-0.4456	-0.1721	0.4052	-1.1924	-0.0291	-0.0280
-1.4021	0.7825	0.4929	-1.3772	0.4327	-0.0123	-2.2852	0.0580	0.1080	-1.4669	-2.3847
-2.9893	-5.5009	-2.8424	-4.4836	-5.6763	-4.4331	-1.9064	-1.2552	-5.5836	-3.6597	-4.3382
-2.6611	-3.6758	-0.6466	-2.9720	-2.9807	-1.1242	-2.8645	-3.3612	-0.5586	-2.4258	-4.7078
9.7977	6.5608	10.6843	6.9563	6.9137	11.6656	7.6810	10.9994	7.5020	8.6652	10.9694
9.6074	6.9009	10.0060	6.3915	6.0839	11.2684	7.7132	10.2456	8.3746	8.0165	9.9485
-3.5199	1.6958	-4.3812	4.3282	-3.2745	3.1313	4.9080	-2.4923	-7.0756	-7.7675	-5.3691
0.8699	-1.0498	-0.0452	-1.6959	1.4672	-1.0346	-0.5386	1.1863	-0.7130	0.6563	2.2199
-3.1989	-1.3541	-6.1465	1.9398	-3.3452	-7.0267	2.6404	-6.2410	-7.0756	-7.7675	-7.1222
4.8383	3.7036	6.4270	2.3253	4.0231	5.8698	2.6880	6.5350	3.6946	5.3998	3.6102
-5.3968	-9.7143	-5.1251	-8.5124	-5.8804	-7.0267	-8.5448	-7.1846	-5.5870	-6.6683	-7.1222
-5.3968	-9.7143	-6.1465	-9.6251	-5.8804	-7.0267	-8.5448	-7.1846	-6.9177	-6.9650	-7.1222
8.9437	7.8508	10.1095	6.3114	-1.5410	9.7352	7.0410	9.3739	10.0769	8.9635	11.6232
-5.3968	-4.5992	-6.1465	0.7295	0.2186	-7.0267	2.1289	-7.1846	-7.0756	-5.8273	-7.1222
5.0151	4.3775	6.2162	2.2294	4.8583	6.1506	3.9610	6.5513	5.0326	5.7870	5.2802
-5.2439	-7.5226	-6.1465	-9.6251	-5.8804	-6.6070	-7.6058	-5.4699	-4.5907	-4.9825	-7.1222
2.0766	0.0391	3.7335	-1.8451	0.0006	2.0674	-0.5541	3.9951	1.2620	2.8815	2.4314
5.4974	2.8215	5.3979	2.1929	3.2403	6.0219	3.6791	6.3786	4.9320	5.5271	5.5492
-0.5972	-1.9398	0.6721	-4.4652	-1.1130	0.9202	-3.8118	-2.2621	-1.1913	-0.8723	1.0549
4.6999	3.7084	6.5090	1.4076	4.1728	6.2080	3.3503	6.5478	4.2790	5.7037	5.5343
1.6524	3.7343	7.7397	3.5267	2.9721	9.3680	4.4187	1.4122	5.7839	3.8019	3.1881
2.1658	1.2744	2.9618	-2.3655	0.3216	3.4969	-1.7563	3.6791	-0.3370	1.4145	1.7382
8.3418	5.3144	6.3011	1.7808	5.7540	7.0004	2.7974	7.4256	5.2062	7.0541	7.5819
4.4515	3.0332	4.7126	1.3395	2.7576	4.5770	2.3526	4.4264	2.0996	4.1693	3.3030
7.5735	7.3938	8.2223	6.3865	5.9909	8.5623	5.5436	8.2524	6.6147	6.8002	7.4533
-5.3968	-9.7143	-6.1465	-9.6251	-5.8804	-7.0267	-8.5448	-7.1846	-7.0756	-7.7675	-7.1222
-5.3968	-1.5270	-6.1465	3.0567	-1.6551	-7.0267	2.8059	-7.1846	-7.0756	-7.7675	-7.1222
-3.1310	-1.6728	-3.5114	1.3824	-1.1495	-4.8648	2.2249	-4.0433	-4.2566	-4.6457	-1.2627
-5.3968	-9.7143	-6.1465	-3.6743	-2.8353	-7.0267	-8.5448	-7.1846	-6.7177	-7.7675	1.0736
-5.3968	-9.7143	-6.1465	-9.6251	-5.8804	-7.0267	-8.5448	-5.7329	-6.3929	-5.9170	-7.1222
-5.3968	-5.5984	0.0452	-4.5620	0.3160	-0.4589	-4.2091	-1.2358	-0.1080	-0.2034	2.7386
4.6225	4.4413	6.3273	3.0804	3.2683	6.4059	3.1933	5.8530	5.0721	5.9939	3.6549
5.8195	5.7901	5.2905	4.6201	4.0824	5.0637	4.2357	4.9221	5.0803	5.6683	5.3702
11.1305	9.6108	9.8551	5.6783	6.6982	9.6529	6.7858	11.0161	10.1246	10.8918	8.0776
10.1818	9.8195	10.0105	4.9001	7.2347	10.4133	7.2926	9.7918	6.8795	6.9176	5.2217
0.7449	3.0936	0.2499	-1.1242	-3.8088	2.5286	-1.0005	-0.0580	2.9427	0.0291	-2.5769
6.6065	6.7252	7.4147	4.1785	5.6253	6.8757	4.6199	6.9681	6.0744	6.7967	5.9911

Table S4. Normalised Ct values for plates A and B.

NBM40107	NBM5	KG1	NB4
-3.0335	-3.6925	-6.8973	4.0083
12.3607	-6.0049	-10.3397	-0.1376
0.6025	-2.0163	-10.3397	-9.4989
3.3909	3.1921	2.2117	4.7084
9.1099	4.9091	5.1092	7.4139
0.3904	-1.0421	2.0143	2.2006
6.7995	4.3912	8.1300	9.2692
8.2220	6.1418	8.1286	8.9203
3.1555	3.9502	6.3449	7.1183
4.6096	6.8598	5.8793	6.4071
4.9287	4.7090	5.4530	6.9631
2.6096	0.2011	1.4616	2.6175
4.1454	5.4103	2.5402	7.9119
14.9812	6.4007	1.7590	4.2456
6.6096	4.8827	2.1269	4.8653
-2.1534	2.8366	3.9009	5.3039
-0.9300	0.6270	0.5111	2.0416
-0.4853	1.1779	-2.0932	-0.7658
4.6096	6.5122	3.6861	6.3486
3.5304	5.2295	3.9093	6.1554
3.1591	-0.3001	-1.6535	1.2898
3.5201	5.3082	5.2462	6.3939
3.9024	4.6771	4.0540	5.5186
-0.3904	3.3876	2.8623	4.0118
3.2122	2.2032	-0.7068	1.1836
-2.3048	-2.0467	-8.0965	-0.8128
8.0710	-10.4276	-10.3397	-9.4989
3.9635	5.8629	6.5431	8.0547
-10.3904	-4.4010	-10.3397	0.7416
-0.3904	-0.3435	1.0769	1.2461
-2.0843	-1.3890	2.6659	-3.9934
-2.1698	-1.1685	2.8467	-3.4136
9.6096	4.5949	5.3531	7.9839
4.2559	3.8840	6.7055	8.0797
-2.1200	-2.0003	-1.2235	2.7145
-1.0910	2.0288	-4.9853	-0.7731
3.2598	0.7212	3.6606	-0.2919
-1.3904	-0.9787	1.5741	-5.7837
0.8045	2.8527	1.3835	-2.3529
-3.1048	-8.8479	-10.3397	-9.4989
-2.3762	-3.1892	-3.0211	0.1376
-2.6166	-4.3683	-3.5095	-1.7289
-3.3904	-8.1511	-10.3397	0.7552
1.8223	2.7439	5.0116	-2.7322
-0.9312	2.0058	3.1760	4.5397
-2.9489	-0.3266	-4.4652	-2.4330
-4.4974	-3.3523	1.5897	-7.0320
-2.7259	-0.2011	3.0219	-2.6391
-1.8049	-6.0819	-9.9189	-9.4989
3.8832	3.6247	2.0428	4.2104
1.2502	2.6845	1.8443	4.2597
2.4522	2.0054	-3.0572	2.6033
4.8958	5.3804	4.0854	5.2219
9.1145	-6.2188	-10.3397	-9.1448
-1.3904	2.2147	-0.5111	3.6207
4.4247	7.9637	-0.7804	-6.3177
1.3472	2.9033	0.9153	-9.4989
-5.3904	0.6138	-2.0263	-2.3757
-3.2389	-5.7383	-6.3726	-6.3075
-4.6090	-5.1537	-6.3726	-6.3075
1.2016	3.2262	5.9246	6.5302
7.1702	6.2278	8.7812	10.5648
6.1649	5.7762	9.0549	10.7297
-3.0126	0.9858	1.4252	3.7098
2.7200	1.9282	-5.2499	8.3444
-8.2800	-4.5082	-6.3726	1.2125
-3.1465	-1.3525	0.5171	-0.3385

Table S4. Normalised Ct values for plates A and B.

3.2106	2.6021	0.7258	2.0662
-1.5828	-4.6052	-2.8984	-6.3075
-2.5410	-3.7278	-3.7742	-0.8693
0.7200	0.2439	0.6876	5.7596
-1.0929	3.9952	6.6637	4.9777
-1.2800	3.5908	1.8038	3.7281
4.6538	4.8852	5.1895	6.7806
5.0228	2.2907	7.8405	-1.8167
6.1337	5.1912	9.5966	9.2207
7.2333	2.6407	2.1382	7.4073
5.9854	2.6912	8.5595	1.4297
-1.7248	-1.7263	-6.3726	-2.1467
-3.2800	-3.4646	-6.3726	-6.3075
0.8526	0.4332	-0.4575	1.0905
-2.6910	-5.5566	-1.5311	-6.3075
-3.8321	-5.5386	-6.3726	-6.3075
-2.4828	-8.0700	-6.3726	-6.3075
2.7200	2.0704	9.0563	8.6472
5.3131	1.6637	4.1576	6.2500
-2.2868	0.1416	7.8295	-2.7106
1.7200	0.8168	0.2390	1.1241
-2.3463	-4.4514	1.2109	0.6541
-2.7164	-5.0383	-1.8262	-1.8157
7.7200	4.9812	9.1521	11.2675
6.2731	4.3083	7.9991	10.6043
-2.0473	-7.1920	-6.3726	-6.3075
0.1941	0.1018	-2.2813	-1.5563
-2.2985	-8.0700	-6.3726	-6.3075
8.3928	1.7747	6.0796	6.9198
5.0402	-8.0700	-6.3726	-6.3075
2.2007	-1.6418	-0.2390	-0.6602
6.3675	6.6101	8.9321	8.1293
-3.4867	-6.3038	-6.3726	-6.3075
1.0859	2.7705	4.6382	6.7971
26.0663	-6.4759	-6.3726	-6.3075
-5.2800	-0.1018	1.8723	1.7525
1.7200	2.1237	3.2998	2.4438
-1.2800	-3.5385	-0.4107	0.3385
-0.2295	2.8913	5.6697	6.1461
13.2635	2.6195	3.3787	5.1585
2.5679	1.3977	-0.2899	-2.1209
3.7200	3.7441	5.4899	8.2362
-0.1941	0.8001	0.4991	1.7255
7.7200	7.7139	9.9175	8.6780
5.4633	-6.9714	-6.3726	-6.3075
-4.3186	-5.9481	-6.3726	-6.3075
-1.0074	-6.7846	-6.3726	-6.3075
-8.2800	-7.7954	-6.3726	-6.3075
1.7200	-8.0700	-3.2000	-6.3075
-5.1440	-0.8836	-1.2478	-0.7832
0.4417	3.0101	3.2742	4.2647
3.7379	3.8775	3.3864	4.4430
8.7200	5.9424	8.4890	6.6430
6.5224	6.2038	8.3705	7.5354
-3.5246	-3.0637	2.1589	-4.2125
7.7200	4.0312	3.0863	3.7264
