# Supplementary material for: Distinctive Patterns of MicroRNA Expression Associated with Karyotype in Acute Myeloid Leukaemia
Source: PLoS One. 2008 May 14;3(5):e2141. doi: 10.1371/journal.pone.0002141 (PMC2373886; doi:10.1371/journal.pone.0002141)
Supplement: Table S5 — List of the 94 miRNAs that passed the 5% FDR filter in the ANOVA test. The miRNAs are reported alongside their accession number and sequence (Sanger miRBase database v5.0, Sept 2004, http://microrna.sanger.ac.uk/. n/a refers to miRNAs removed from the Registry). (0.04 MB PDF) [file pone.0002141.s005.pdf]

Table S5. List of the 94 miRNAs that passed the 5% FDR filter in the ANOVA test.

Mature miRNA	Accession number	Mature miRNA Sequence
hsa-let-7b	MIMAT0000063	ugagguaguagguugugugguu
hsa-let-7d	MIMAT0000065	agagguaguagguugcauagu
hsa-let-7i	MIMAT0000415	ugagguaguaguuugugcu
hsa-miR-100	MIMAT0000098	aacccguagauccgaacuugug
hsa-miR-103	MIMAT0000101	agcagcauuguacagggcuauga
hsa-miR-106a	MIMAT0000103	aaaagugcuuacagugcagguagc
hsa-miR-107	MIMAT0000104	agcagcauuguacagggcuauca
hsa-miR-10a	MIMAT0000253	uacccuguagauccgaauuugug
hsa-miR-125b	MIMAT0000423	ucccugagacccuaacuuguga
hsa-miR-126	MIMAT0000445	ucguaccgugaguaauaaugc
hsa-miR-127	MIMAT0000446	ucggauccgucugagcuuggcu
hsa-miR-128a	MIMAT0000424	ucacagugaaccggucucuuuu
hsa-miR-128b	MIMAT0000676	ucacagugaaccggucucuuuc
hsa-miR-129	MIMAT0000242	cuuuuugcggucugggcuugc
hsa-miR-130a	MIMAT0000425	cagugcaauguuaaaagggc
hsa-miR-130b	MIMAT0000691	cagugcaaugaugaaagggcau
hsa-miR-132	MIMAT0000426	uaacagucuacagccauggucg
hsa-miR-133a	MIMAT0000427	uugguccccuucaaccagcugu
hsa-miR-133b	MIMAT0000770	uugguccccuucaaccagcua
hsa-miR-135a	MIMAT0000428	uauggcuuuuuauuccuauguga
hsa-miR-140	MIMAT0000431	agugguuuuacccuaugguag
hsa-miR-145	MIMAT0000437	guccaguuuucccaggaaucccuu
hsa-miR-146	MIMAT0000449	ugagaacugaauuccauggguu
hsa-miR-148a	MIMAT0000243	ucagugcacuacagaacuuugu
hsa-miR-150	MIMAT0000451	ucucccaacccuuguaccagug
hsa-miR-154	MIMAT0000452	uagguuauccguguugccuucg
hsa-miR-154*	MIMAT0000453	aaucauacacgguugaccuauu
hsa-miR-155	MIMAT0000646	uuaaugcuaaucgugauaggggg
hsa-miR-17-3p	MIMAT0000071	acugcagugaaggcacuugu
hsa-miR-17-5p	MIMAT0000070	caaagugcuuacagugcagguagu
hsa-miR-181b	MIMAT0000257	aacauucauugcugucgguggguu
hsa-miR-181c	MIMAT0000258	aacauucaaccugucggugagu
hsa-miR-182	MIMAT0000259	uuuggcaaugguagaacucaca
hsa-miR-183	MIMAT0000261	uauggcacugguagaauucacug
hsa-miR-184	MIMAT0000454	uggacggagaacugauaagggu
hsa-miR-185	MIMAT0000455	uggagagaaaggcaguuc
hsa-miR-187	MIMAT0000262	ucgugucuuguguugcagccg
hsa-miR-190	MIMAT0000458	ugauauguuugauauauuaggu
hsa-miR-193	MIMAT0000459	aacuggccuacaaagucccag
hsa-miR-194	MIMAT0000460	uguaacagcaacuccaugugga
hsa-miR-195	MIMAT0000461	uagcagcacagaaauauuggc
hsa-miR-197	MIMAT0000227	uucaccaccuucuccacccagc
hsa-miR-199a	MIMAT0000231	cccaguguucagacuaccuguuc
hsa-miR-199a*	MIMAT0000232	uacaguagucugcacauugguu
hsa-miR-199b	MIMAT0000263	cccaguguuuagacuaucuguuc
hsa-miR-199-s	n/a	cccaguguucagacuaccuguu
hsa-miR-19a	MIMAT0000073	ugugcaaaucuaugcaaaacuga
hsa-miR-20	MIMAT0000075	uaaagugcuuauagugcaggua
hsa-miR-200a	MIMAT0000682	uaacacugucugguaacgaugu
hsa-miR-200b	MIMAT0000318	cucuaauacugccugguaaugaug
hsa-miR-200c	MIMAT0000617	aauacugccggguaaugaugga
hsa-miR-203	MIMAT0000264	gugaaauguuuaggaccacuag
hsa-miR-204	MIMAT0000265	uucccuuugucauccuaugccu
hsa-miR-210	MIMAT0000267	cugugcgugugacagcggcug
hsa-miR-214	MIMAT0000271	acagcaggcacagacaggcag
hsa-miR-215	MIMAT0000272	augaccuaugaauugacagac
hsa-miR-218	MIMAT0000275	uugugcuugaucuaaccaugu

Table S5. List of the 94 miRNAs that passed the 5% FDR filter in the ANOVA test.

hsa-miR-219	MIMAT0000276	ugauuguccaaacgcaauucu
hsa-miR-222	MIMAT0000279	agcuacaucuggcuacugggucuc
hsa-miR-224	MIMAT0000281	caagucacuagugguuccguuua
hsa-miR-25	MIMAT0000081	cauugcacuugucucggucuga
hsa-miR-26b	MIMAT0000083	uucaaguaauucaggauaggu
hsa-miR-27b	MIMAT0000419	uucacaguggcuaaguucug
hsa-miR-28	MIMAT0000085	aaggagcucacagucuauugag
hsa-miR-296	MIMAT0000690	agggcccccccucaauccugu
hsa-miR-299	MIMAT0002890	ugguuuaccgucccacauacau
hsa-miR-29b	MIMAT0000100	uagcaccauuugaaaucagu
hsa-miR-301	MIMAT0000688	cagugcaauaguauugucaaagc
hsa-miR-302d	MIMAT0000718	uaagugcuuccauguuugagugu
hsa-miR-30b	MIMAT0000420	uguaaacauccuacacucagc
hsa-miR-30c	MIMAT0000244	uguaaacauccuacacucucagc
hsa-miR-30d	MIMAT0000245	uguaaacauccccgacuggaag
hsa-miR-30e	MIMAT0000692	uguaaacauccuugacugga
hsa-miR-320	MIMAT0000510	aaaagcuggguugagagggcgaa
hsa-miR-323	MIMAT0000755	gcacauuacacggucgaccucu
hsa-miR-324-5p	MIMAT0000761	cgcauccccuagggcauuggugu
hsa-miR-326	MIMAT0000756	ccucugggcccuucccuccag
hsa-miR-328	MIMAT0000752	cuggcccucucugcccuuccgu
hsa-miR-330	MIMAT0000751	gcaaagcacacggccugcagaga
hsa-miR-331	MIMAT0000760	gccccugggccuauccuagaa
hsa-miR-335	MIMAT0000765	ucaagagcaauaacgaaaaaugu
hsa-miR-338	MIMAT0000763	uccagcaucagugauuuuguuga
hsa-miR-339	MIMAT0000764	ucccuguccuccaggagcuca
hsa-miR-340	MIMAT0000750	uccgucucaguuacuuuauagcc
hsa-miR-34a	MIMAT0000255	uggcagugucuuagcugguugu
hsa-miR-367	MIMAT0000719	aauugcacuuuagcaaugguga
hsa-miR-368	MIMAT0000720	acauagaggaaauuccacguuu
hsa-miR-370	MIMAT0000722	gccugcugggguggaaccugg
hsa-miR-372	MIMAT0000724	aaagugcugcgacauuugagcgu
hsa-miR-374	MIMAT0000727	uuauaauacaaccugauaagug
hsa-miR-9	MIMAT0000441	ucuuugguuaucuagcuguauga
hsa-miR-9*	MIMAT0000442	uaaagcuagauaaccgaaagu
hsa-miR-98	MIMAT0000096	ugagguaguaaguuguauuguu
hsa-miR-99a	MIMAT0000097	aacccguagauccgaucuugug
