# Supplementary material for: Distinctive Patterns of MicroRNA Expression Associated with Karyotype in Acute Myeloid Leukaemia
Source: PLoS One. 2008 May 14;3(5):e2141. doi: 10.1371/journal.pone.0002141 (PMC2373886; doi:10.1371/journal.pone.0002141)
Supplement: Protocol S1 — Locked nucleic acid fluorescent in situ hybridisation (LNA-FISH) detailed protocol. (0.05 MB DOC) [file pone.0002141.s006.doc]

Protocol S1. Locked nucleic acid fluorescent in situ hybridisation (LNA-FISH) detailed protocol.

One hundred pmol of LNA detection probe was labelled with digoxigenin (DIG) using a DIG 3’end labelling kit (P/N 03353583910, Roche) according to manufacturer’s instructions. Labelled probes were purified using Sephadex G25 Microspin columns (Amersham), and reaction efficiency and approximate concentration were determined by dot-blot on nitrocellulose membrane. Cell suspensions from thawed BM or PB were washed twice in 1X phosphate-buffered saline (PBS) and finally resuspended to a concentration of 0.5 million cells/ml. One hundred μl of this suspension was pipetted into a cytospin column and spun at 300 rpm for 5 minutes on poly-*l*-lysine (Sigma Aldrich) coated glass slides, using a Shandon Cytospin 3 Cytocentrifuge (Fisher-Scientific UK Ltd). Slides were air-dried before proceeding to the next step. Hybridisation mixture containing ~30ng/μl of labelled probe in 200μl of hybridisation buffer (50% deionised formamide, 0.3M NaCl, 20mM Tris HCl, pH8.0, 5mM EDTA, 10mM NaPO_4_, pH8.0, 10% dextran sulfate, 1X Denhardt’s solution and 0.5mg/ml yeast RNA) was heated to 65°C and then applied to the slides. Slides were covered with RNase-free coverslips (H18200, Invitrogen) and incubated for 17-18 hours at a temperature 20-22°C below the melting temperature (Tm) of the miRCURY LNA probe used in a humidified HYBrite™ (Abbott Laboratories Ltd). Slides were washed once, for 5 minutes, in 50% formamide/2X SSC at the same temperature that hybridisation had been performed. This was followed by a further wash at room temperature using 1X PBS/0.1%Tween 20 for 5 minutes. Slides were then incubated for 1 hour in blocking solution (0.5% blocking reagent (1096176, Roche), 10% heat-inactivated sheep serum (16070-096, Invitrogen), 0.1% Tween-20 and PBS). Anti-DIG fluorescein isothiocynate (FITC) conjugated antibody, diluted to a final concentration of 1μg/ml in 1 X PBS/0.5% bovine serum albumin (w/v), was applied to the slide and placed in a humidified chamber, for 1 hour at 37°C. Slides were then washed in 1X PBS/0.1%Tween 20 at 37°C, drained and counterstained with 4’-6’Diamidino-2-phenylindole (DAPI), before the addition of ProLong® Gold antifade reagent (Invitrogen).
